# Supplementary figures and images for: High-order radiomics features based on T2 FLAIR MRI predict multiple glioma immunohistochemical features: A more precise and personalized gliomas management
Source: PLoS One. 2020 Jan 22;15(1):e0227703. doi: 10.1371/journal.pone.0227703 (PMC6975558; doi:10.1371/journal.pone.0227703)

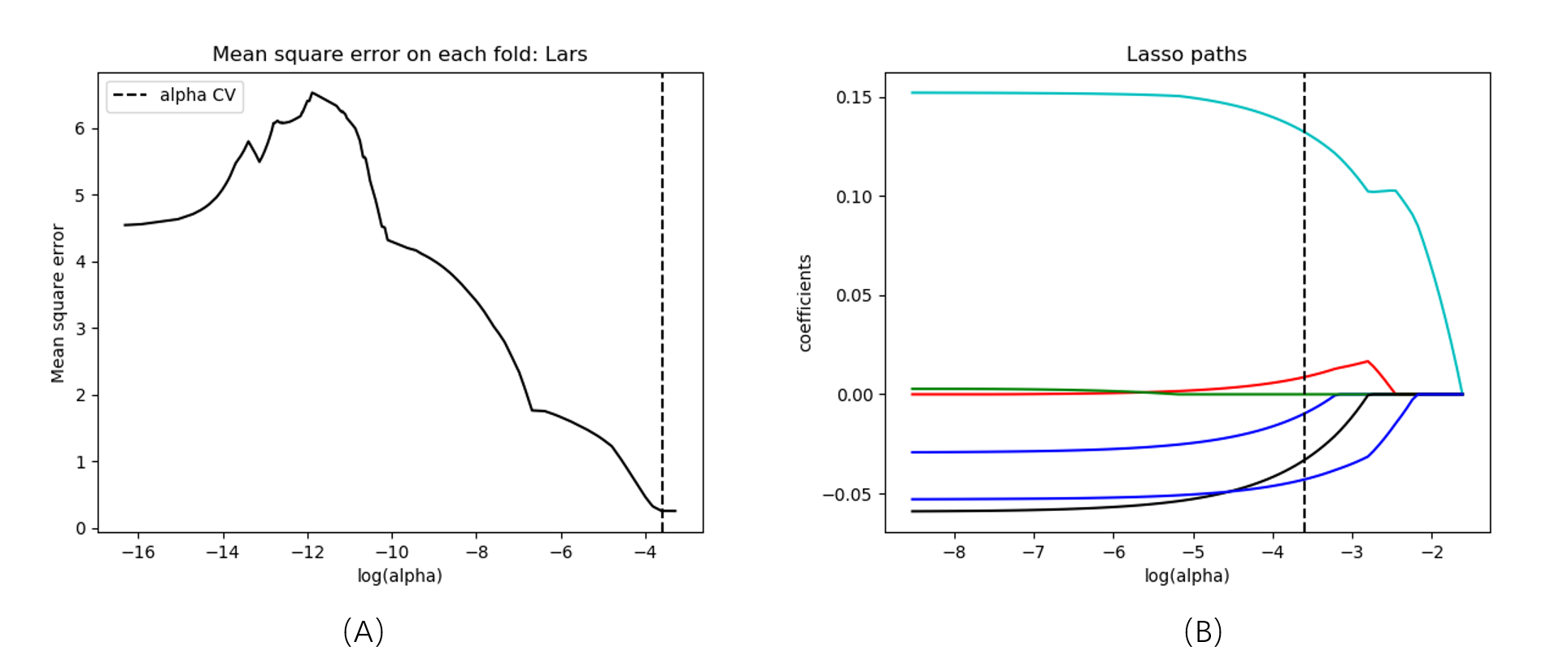

Supplement: S1 Fig — (A) The lasso model first choose the optimal log (ɑ) according to the minimum mean square error among the 10-fold cross validation.(B) The lasso model compresses the unimportant feature coefficients to zero according to the optimal log (ɑ) value. (TIF) [file pone.0227703.s001.tif]

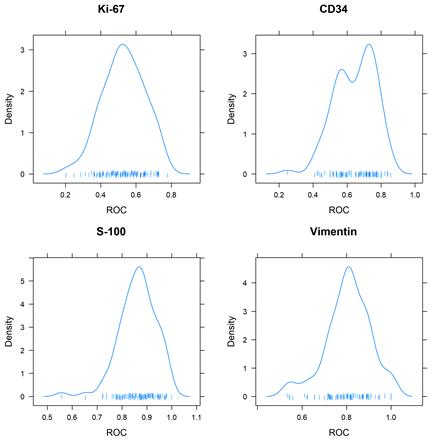

Supplement: S2 Fig — (TIF) [file pone.0227703.s002.tif]

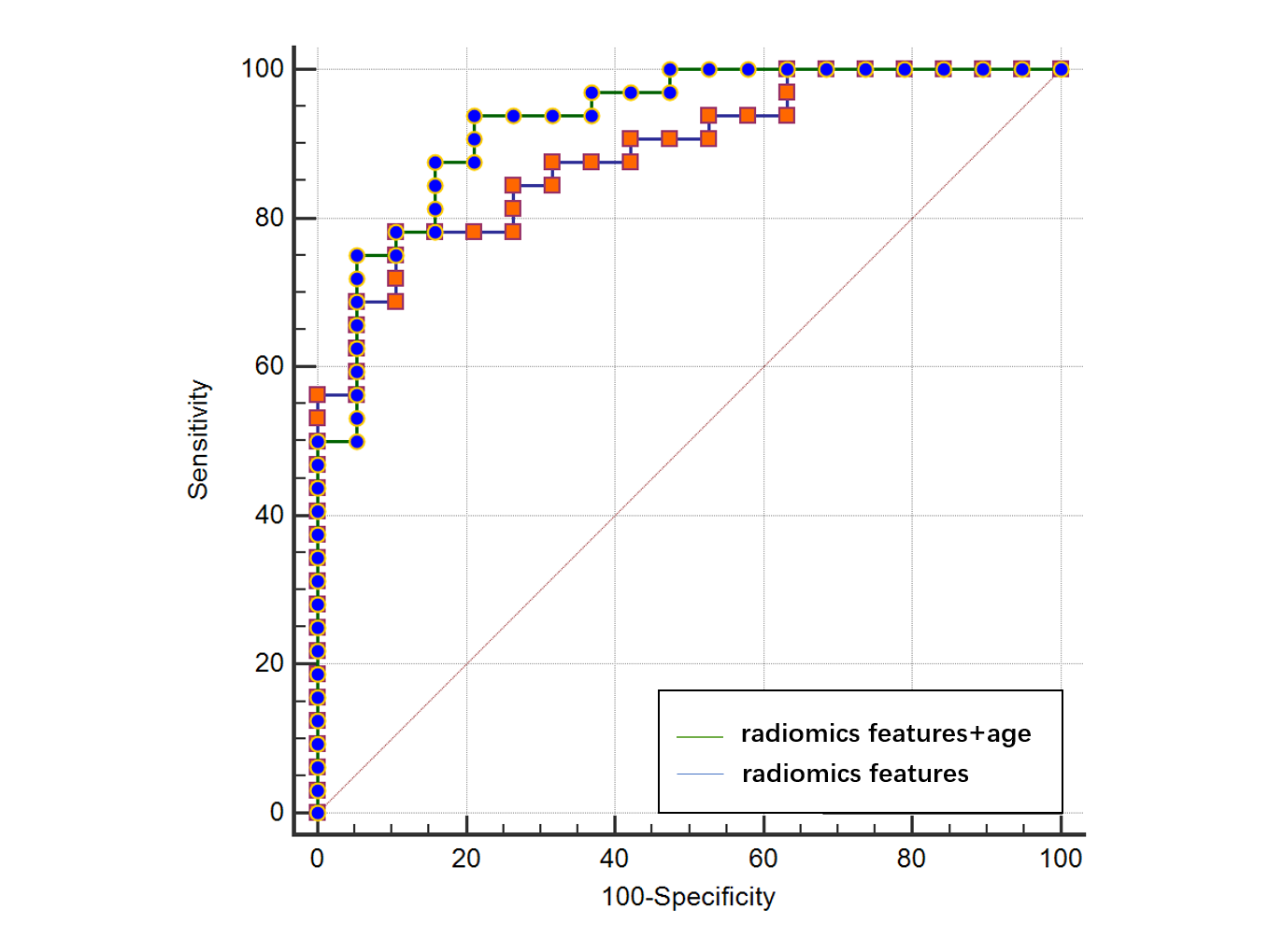

Supplement: S3 Fig — (TIF) [file pone.0227703.s003.tif]

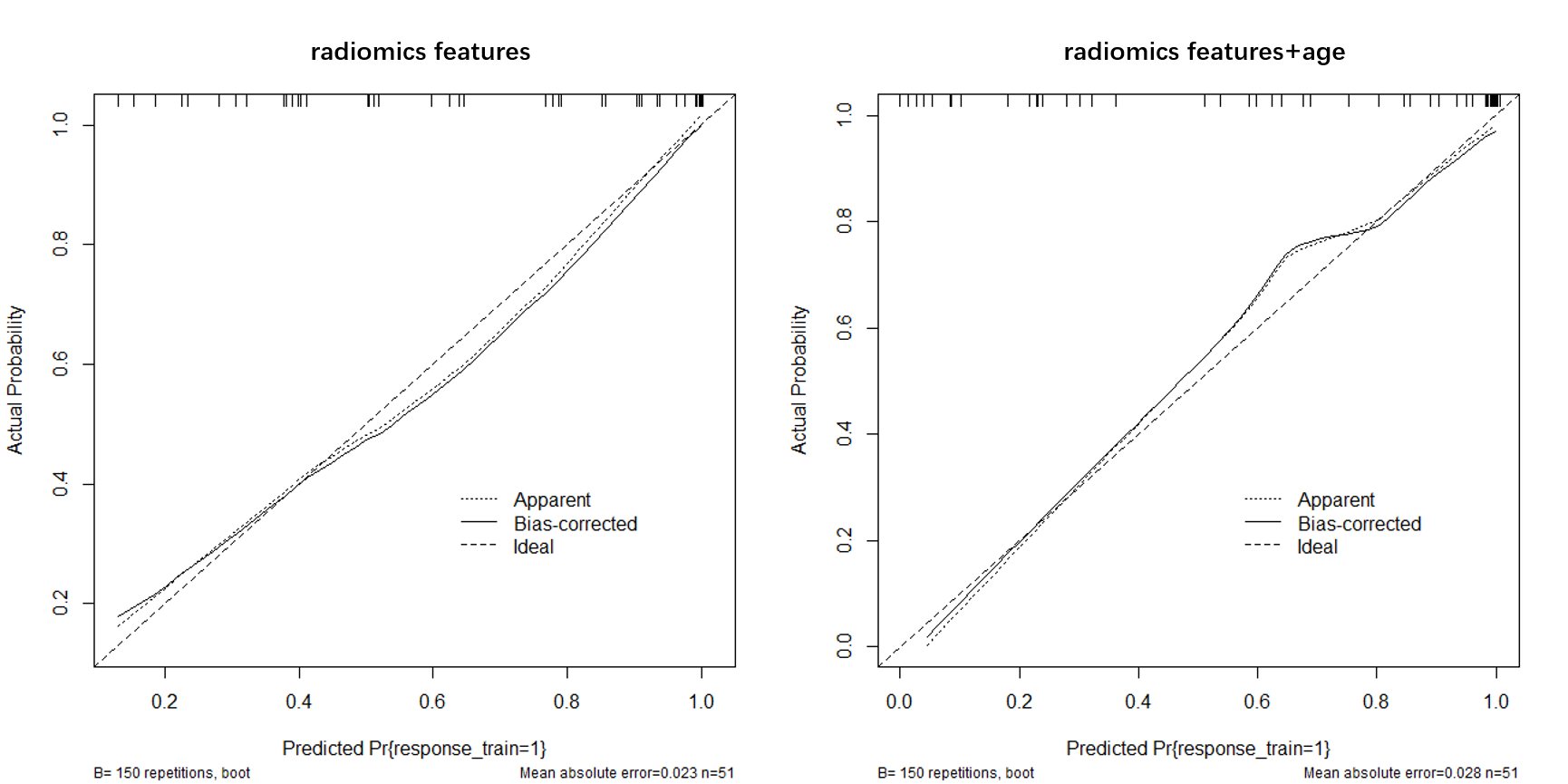

Supplement: S4 Fig — (TIF) [file pone.0227703.s004.tif]

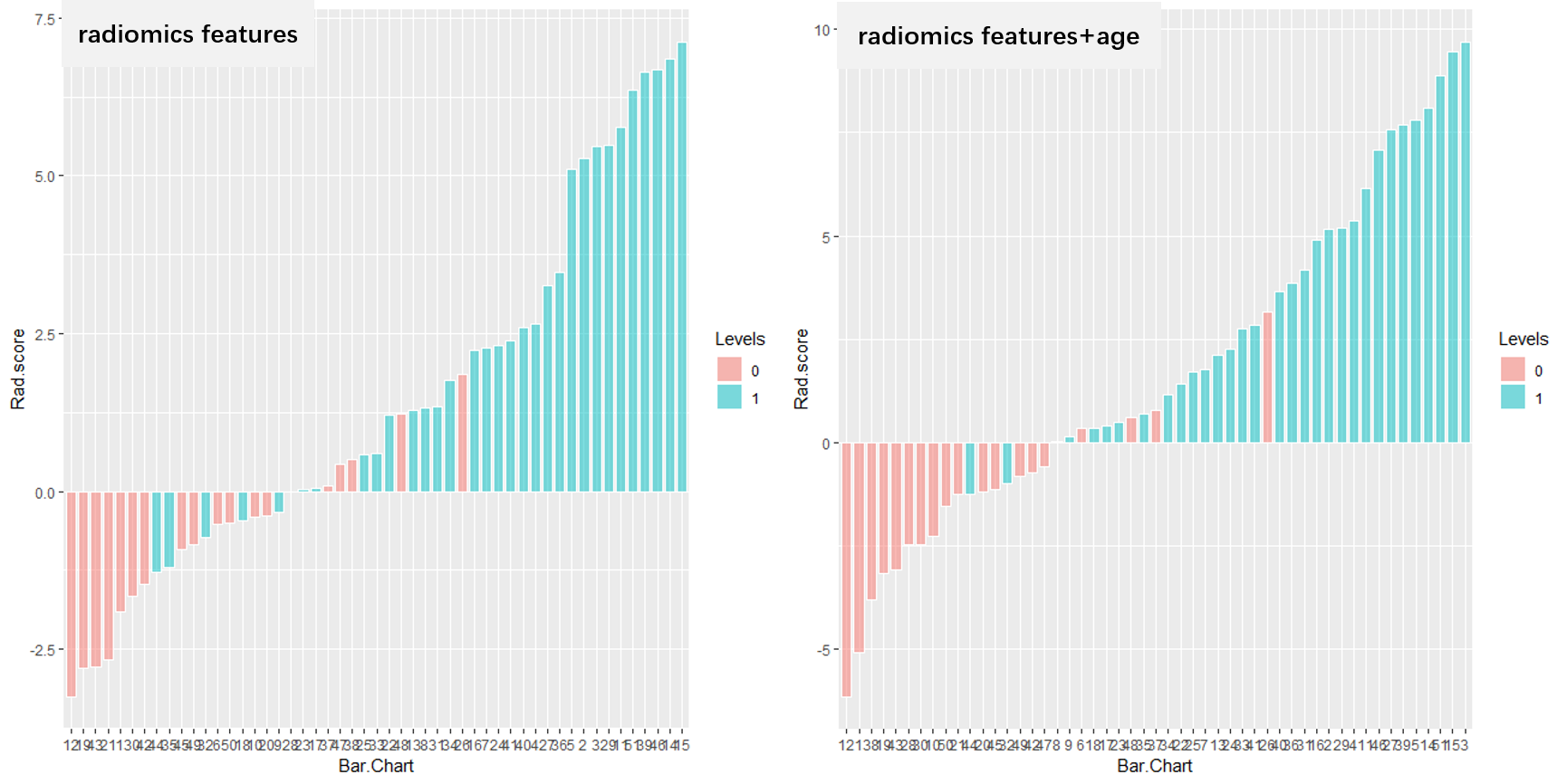

Supplement: S5 Fig — (TIF) [file pone.0227703.s005.tif]
