# Supplementary material for: High-order radiomics features based on T2 FLAIR MRI predict multiple glioma immunohistochemical features: A more precise and personalized gliomas management
Source: PLoS One. 2020 Jan 22;15(1):e0227703. doi: 10.1371/journal.pone.0227703 (PMC6975558; doi:10.1371/journal.pone.0227703)
Supplement: S1 Formula — (DOCX) [file pone.0227703.s006.docx]

Radscore$=$0.153$+$0.209 kurtosis

$+$0.708ClusterProminence_AllDirection_offset1_SD

$-$0.478HaralickCorrelation_AllDirection_offset7

$+$0.058Inertia_AllDirection_offset1_SD

$+$0.712SizeZoneVariability
