# Supplementary material for: High-order radiomics features based on T2 FLAIR MRI predict multiple glioma immunohistochemical features: A more precise and personalized gliomas management
Source: PLoS One. 2020 Jan 22;15(1):e0227703. doi: 10.1371/journal.pone.0227703 (PMC6975558; doi:10.1371/journal.pone.0227703)
Supplement: S2 Formula — (DOCX) [file pone.0227703.s007.docx]

Radscore$=$1.715$-$2.152 MinIntensity

$-$2.088 ClusterProminence_AllDirection_offset1_SD

$+$0.432 Correlation_angle90_offset1

$+$0.196 GLCMEntropy_AllDirection_offset4_SD

$+$3.578 LongRunLowGreyLevelEmphasis_angle45_offset1
