# Supplementary material for: High-order radiomics features based on T2 FLAIR MRI predict multiple glioma immunohistochemical features: A more precise and personalized gliomas management
Source: PLoS One. 2020 Jan 22;15(1):e0227703. doi: 10.1371/journal.pone.0227703 (PMC6975558; doi:10.1371/journal.pone.0227703)
Supplement: S5 Formula — (DOCX) [file pone.0227703.s010.docx]

**Positive Risk Probability**$\boldsymbol{=}\frac{\boldsymbol{1}}{\boldsymbol{1+}\boldsymbol{e}^{\boldsymbol{(-Radscore)}}}$
