# Supplementary material for: High-order radiomics features based on T2 FLAIR MRI predict multiple glioma immunohistochemical features: A more precise and personalized gliomas management
Source: PLoS One. 2020 Jan 22;15(1):e0227703. doi: 10.1371/journal.pone.0227703 (PMC6975558; doi:10.1371/journal.pone.0227703)
Supplement: S2 Table — (DOCX) [file pone.0227703.s012.docx]

The primary radiomics features extracted in this study

| **Feature type** | **Feature name** |
| --- | --- |
| **Histogram features** | Minimum Intensity |
|  | Maximum Intensity |
|  | Median Intensity |
|  | Mean Intensity |
|  | Standard Deviation |
|  | Variance |
|  | Voxel Count |
|  | Voxel Value Sum |
|  | Range |
|  | Root Mean Square |
|  | Mean Deviation |
|  | Relative Deviation |
|  | Skewness |
|  | Kurtosis |
|  | Uniformity |
|  | Energy |
|  | Energy |
|  | Frequency Size |
|  | Percentiles^&^ |
|  | Quantiles^$^ |
| **GLCM features** | Cluster Shade^#^ |
|  | Inverse Difference Moment^#^ |
|  | Cluster Prominence^#^ |
|  | Correlation^#^ |
|  | Entropy of GLCM^#^ |
|  | Energy of GLCM^#^ |
|  | Inertia of GLCM^#^ |
|  | Haralick Correlation^#^ |
|  | Angular Second Moment |
|  | Contrast |
|  | Hara Variance |
|  | Inverse Difference Moment |
|  | Sum Average |
|  | Sum Variance |
|  | Sum Entropy |
|  | Haralick Entropy |
|  | Difference Variance |
|  | Difference Entropy |
| **GLRLM features** | Short Run Emphasis* |
|  | Long Run Emphasis* |
|  | Gray Level Non-uniformity* |
|  | Run Length Non-uniformity* |
|  | Low Gray Level Run Emphasis* |
|  | High Gray Level Run Emphasis* |
|  | Short Run Low Gray Level Emphasis* |
|  | Short Run High Gray Level Emphasis* |
|  | Long Run Low Gray Level Emphasis* |
|  | Long Run High Gray Level Emphasis* |
| **GLSZM features** | Small Zone Emphasis |
|  | Large Zone Emphasis |
|  | Zone Percentage |
|  | Low Gray Level Zone Emphasis |
|  | High Gray Level Zone Emphasis |
|  | Small Zone Low Gray Level Emphasis |
|  | Small Zone High Gray Level Emphasis |
|  | Large Zone Low Gray Level Emphasis |
|  | Large Zone High Gray Level Emphasis |
|  | Gray Level Variance |
|  | Zone-Size Variance |
| **Form Factor features** | Sphericity |
|  | Surface Area |
|  | Compactness 1 |
|  | Compactness 2 |
|  | Maximum 3D Diameter |
|  | Spherical Disproportion |
|  | Surface to Volume ratio |
|  | VolumeCC |
|  | VolumeMM |

**Note:** ^&^ There are 19 percentiles from Percentile5 to Percentile95 with the interval of 5.

^$^ Quantiles include Quantile0.025, Quantile0.25, Quantile0.5, Quantile0.75, and Quantile0.975.

^#^ Offsets of GLCM features extracted in this study include 1, 4, and 7, and the rotation angles are 0°, 45°, 90°, and 135°. By varying the displacement vector between each pair of pixels, we have 18 parameters related to each of GLCM features. Take Cluster Shade as an example, its feature set includes the following 18 features: ClusterShade_AllDirection_offset1, ClusterShade_AllDirection_offset1_SD, ClusterShade_angle0_offset1, ClusterShade_angle45_offset1, ClusterShade_angle90_offset1, ClusterShade_angle145_offset1, ClusterShade_AllDirection_offset4, ClusterShade_AllDirection_offset4_SD, ClusterShade_angle0_offset4, ClusterShade_angle45_offset4, ClusterShade_angle90_offset4, ClusterShade_angle145_offset4, ClusterShade_AllDirection_offset7, ClusterShade_AllDirection_offset7_SD, ClusterShade_angle0_offset7, ClusterShade_angle45_offset7, ClusterShade_angle90_offset7, and ClusterShade_angle145_offset7.

* The rotation angles of GLRLM features extracted in this study include 0°, 45°, 90°, and 135°. By varying the displacement vector between each pair of pixels, we have 6 parameters related to each of GLRLM features. Take Short Run Emphasis as an example, its feature set includes the following 6 features: ShortRunEmphasis_AllDirection_offset0, ShortRunEmphasis_AllDirection_SD_offset0, ShortRunEmphasis_angle0_offset0, ShortRunEmphasis_angle45_offset0, ShortRunEmphasis_angle90_offset0, and ShortRunEmphasis_angle145_offset0.

GLCM: gray level co-occurrence matrix, GLRLM: gray level run length matrix, GLSZM: gray level size zone matrix.
