# Supplementary material for: High-order radiomics features based on T2 FLAIR MRI predict multiple glioma immunohistochemical features: A more precise and personalized gliomas management
Source: PLoS One. 2020 Jan 22;15(1):e0227703. doi: 10.1371/journal.pone.0227703 (PMC6975558; doi:10.1371/journal.pone.0227703)
Supplement: S3 Table — (DOCX) [file pone.0227703.s013.docx]

The accurate score of Bootstrap validation method

| Boot ROC | 1st Qu | Median | Mean | 3rd Qu |
| --- | --- | --- | --- | --- |
| Ki67 | 0.456 | 0.534 | 0.530 | 0.615 |
| CD34 | 0.561 | 0.667 | 0.646 | 0.736 |
| S-100 | 0.823 | 0.871 | 0.863 | 0.913 |
| Vimentin | 0.757 | 0.815 | 0.807 | 0.875 |

The accurate score of 3-fold cross validation method

| CV3 ROC | 1st Qu | Median | Mean | 3rd Qu |
| --- | --- | --- | --- | --- |
| Ki67 | 0.440 | 0.493 | 0.486 | 0.551 |
| CD34 | 0.594 | 0.667 | 0.639 | 0.698 |
| S-100 | 0.752 | 0.757 | 0.800 | 0.823 |
| Vimentin | 0.792 | 0.813 | 0.813 | 0.833 |

The accurate score of 5-fold cross validation method.

| CV5 ROC | 1st Qu | Median | Mean | 3rd Qu |
| --- | --- | --- | --- | --- |
| Ki67 | 0.430 | 0.480 | 0.474 | 0.540 |
| CD34 | 0.563 | 0.733 | 0.693 | 0.750 |
| S-100 | 0.875 | 0.950 | 0.923 | 0.958 |
| Vimentin | 0.750 | 0.867 | 0.798 | 0.875 |
