# Supplementary material for: High-order radiomics features based on T2 FLAIR MRI predict multiple glioma immunohistochemical features: A more precise and personalized gliomas management
Source: PLoS One. 2020 Jan 22;15(1):e0227703. doi: 10.1371/journal.pone.0227703 (PMC6975558; doi:10.1371/journal.pone.0227703)
Supplement: S1 File — (PDF) [file pone.0227703.s019.pdf]

河北医科大学第二医院科研伦理委员会
Research Ethics Committee of the second hospital of Hebei Medical University

科研伦理委员会伦理审查决议

Approval Letter of Research Ethics Committee

项目名称<br>Study Title	High-order radiomics features based on T2 FLAIR MRI predict glioma immunohistochemical subtype: a more precise and personalized gliomas management		
主要研究者<br>Principal investigator	刘怀军<br>Huaijun Liu	科室<br>Department	医学影像科
送审日期<br>Date Submitted	2019-04-02	审查决议编号<br>Approval Letter No.	2019-P037
会议地点<br>Meeting Location	/	会议时间<br>Meeting Date	/
审查方式<br>Type of Review	□会议审查 Meeting review<br>■快速审查 Quick review<br>□紧急会议审查 Emergency meeting review		
送审资料<br>Document(s) Reviewed	试验方案 Research Proposal<br>知情同意书 Informed Consent Form<br>主要研究者简历 Curriculum Vitae of Principal Investigator(s)<br>招募受试者的文本内容 Recruitment of subjects<br>安全措施及应急预案 Safety measures and emergency plans		
项目来源<br>Issued BY	□纵向课题 ■院级课题、自选课题 □横向课题(医院间)<br>□横向课题（企业间） □伦理备案		
审查结论<br>Evaluation	根据该研究的试验设计，经伦理委员会审查，受试者的健康、权利和隐私得到充分保护，对受试者的潜在风险和伤害可控制到最小。<br>Based on the study designed, after the review of Ethics Committee, the health, rights and privacy of subjects are fully protected; potential risk and harm to subjects are controllable to minimal.<br>同意（Approved）在我院实施。<br>主任委员（签字）<br>Signature Chair: 马景学印<br>河北医科大学第二医院 科研伦理委员会<br>日期：2019 年 4 月 10 日		

地址：河北省石家庄市新华区和平西路 215 号　邮编：050051　电话/传真：0311-66002811
Address: No.215 Heping West Road, Xinhua District, Shijiazhuang ,Hebei, 050051,Tel/Fax:0311-66002811

本伦理委员会组成及操作符合药物临床试验质量管理规范和伦理委员会药物临床试验伦理审查工作指导原则及相关法律法规。

The composition and operation of the Ethics Committee conform to the Criterions for the Quality Control of Clinical Trial of Drugs, the guiding principles for ethical review of drug clinical trials and related laws and regulations.

请遵循 GCP 原则、遵循伦理委员会批准的方案开展临床研究，保护受试者的健康与权利。研究过程中若变更主要研究者，对临床研究方案、知情同意书、招募材料等的任何修改，请申请人提交修正案审查申请。发生严重不良事件，及时提交严重不良事件报告。发生违反试验方案情况须及时报告本伦理委员会。

In order to protect the health and rights of the subjects, the applicant should follow the GCP principles and programs approved by the Ethical Committee during the clinical studies. If any changes are made to the primary investigator(s), the clinical research protocol, informed consent, recruitment materials, etc., the applicant is requested to submit an amendment for review. In the event of a serious adverse event, a serious adverse event report should be submitted in time. The violation of the protocol shall be promptly reported to the Ethics Committee.

请按照伦理委员会规定的年度/定期跟踪审查频率，在截止日期前 1 个月提交研究进展报告；申请人暂停或提前终止临床研究，请及时提交暂停/终止研究报告。完成临床研究，请申请人提交结题报告。

The applicant should submit the study progress report one month before the deadline according to the frequency of the annual / periodic follow-up examination. If the applicant suspends or prematurely terminates the clinical research, the suspension / termination report should be submitted in time. The applicant should submit the final report when they complete the clinical study.

本试验年度/定期跟踪审查频率为一年，本批件有效期为一年。

The annual / periodic follow-up examination frequency is one year and the validity of this document is one year.

地址：河北省石家庄市新华区和平西路 215 号　　邮编：050051　　电话/传真：0311-66002811
Address: No.215 Heping West Road, Xinhua District, Shijiazhuang ,Hebei, 050051,Tel/Fax:0311-66002811
