# Supplementary material for: High-order radiomics features based on T2 FLAIR MRI predict multiple glioma immunohistochemical features: A more precise and personalized gliomas management
Source: PLoS One. 2020 Jan 22;15(1):e0227703. doi: 10.1371/journal.pone.0227703 (PMC6975558; doi:10.1371/journal.pone.0227703)
Supplement: S3 File — (ZIP) [file pone.0227703.s021.zip › statistical analysis/CD34/H-L Test.doc]

GET DATA /TYPE=XLSX
  /FILE='C:\project\hebeishengerglioma\数据分析\CD34\3.xlsx'
  /SHEET=name '3'
  /CELLRANGE=full
  /READNAMES=on
  /ASSUMEDSTRWIDTH=32767.
EXECUTE.
DATASET NAME 数据集1 WINDOW=FRONT.
LOGISTIC REGRESSION VARIABLES Label
  /METHOD=ENTER GreyLevelNonuniformity_angle135_offset4 LowGreyLevelRunEmphasis_angle135_offset1 ShortRunEmphasis_angle135_offset7
  /PRINT=GOODFIT
  /CRITERIA=PIN(0.95) POUT(0.99) ITERATE(20) CUT(0.5).


羅吉斯迴歸


附註	
已建立輸出	19-JUN-2019 16:07:49	
備註		
輸入	作用中資料集	数据集1	
	過濾器	<無>	
	粗細	<無>	
	分割檔案	<無>	
	工作資料檔案中的 N 列	42	
遺漏值處理	遺漏的定義	將使用者定義的遺漏值視為遺漏	
語法	LOGISTIC REGRESSION VARIABLES Label
  /METHOD=ENTER GreyLevelNonuniformity_angle135_offset4 LowGreyLevelRunEmphasis_angle135_offset1 ShortRunEmphasis_angle135_offset7
  /PRINT=GOODFIT
  /CRITERIA=PIN(0.95) POUT(0.99) ITERATE(20) CUT(0.5).	
資源	處理器時間	00:00:00.02	
	經歷時間	00:00:00.02	


[数据集1] 


觀察值處理摘要	
未加權的觀察值a	N	百分比	
選取的觀察值	包含在分析中	42	100.0	
	遺漏觀察值	0	.0	
	總計	42	100.0	
未選取的觀察值	0	.0	
總計	42	100.0	

a. 如果加權有效，請參閱分類表以取得觀察值的總數。	


應變數編碼	
原始值	內部值	
0	0	
1	1	


區塊 0：開始區塊


分類表a,b	
	觀察值	預測值	
		Label	正確百分比	
		0	1		
步驟 0	Label	0	24	0	100.0	
		1	18	0	.0	
	整體百分比			57.1	

a. 常數包含在模型中。	
b. 分割值為 .500	


方程式中的變數	
	B	S.E.	Wald	df	顯著性	Exp(B)	
步驟 0	常數	-.288	.312	.851	1	.356	.750	


未在方程式中的變數	
	分數	df	顯著性	
步驟 0	變數	GreyLevelNonuniformity_angle135_offset4	6.747	1	.009	
		LowGreyLevelRunEmphasis_angle135_offset1	.786	1	.375	
		ShortRunEmphasis_angle135_offset7	4.936	1	.026	
	整體統計資料	7.807	3	.050	


區塊 1：方法 = 輸入


模型係數的 Omnibus 測試	
	卡方	df	顯著性	
步驟 1	步驟	8.710	3	.033	
	區塊	8.710	3	.033	
	模型	8.710	3	.033	


模型摘要	
步驟	-2 對數概似	Cox & Snell R 平方	Nagelkerke R 平方	
1	48.654a	.187	.251	

a. 估計在疊代號 4 處終止，因為參數估計的變更小於 .001。	


Hosmer 與 Lemeshow 測試	
步驟	卡方	df	顯著性	
1	9.214	8	.325	


適用於 Hosmer 與 Lemeshow 測試的列聯表格	
	Label = 0	Label = 1	總計	
	觀察值	期望	觀察值	期望		
步驟 1	1	4	3.664	0	.336	4	
	2	4	3.393	0	.607	4	
	3	2	3.083	2	.917	4	
	4	2	2.700	2	1.300	4	
	5	3	2.423	1	1.577	4	
	6	3	2.140	1	1.860	4	
	7	2	1.934	2	2.066	4	
	8	1	1.625	3	2.375	4	
	9	0	1.524	4	2.476	4	
	10	3	1.515	3	4.485	6	


分類表a	
	觀察值	預測值	
		Label	正確百分比	
		0	1		
步驟 1	Label	0	18	6	75.0	
		1	8	10	55.6	
	整體百分比			66.7	

a. 分割值為 .500	


方程式中的變數	
	B	S.E.	Wald	df	顯著性	
步驟 1a	GreyLevelNonuniformity_angle135_offset4	.806	.491	2.696	1	.101	
	LowGreyLevelRunEmphasis_angle135_offset1	-.406	.394	1.064	1	.302	
	ShortRunEmphasis_angle135_offset7	-.273	.478	.327	1	.567	
	常數	-.386	.357	1.171	1	.279	

方程式中的變數	
	Exp(B)	
步驟 1a	GreyLevelNonuniformity_angle135_offset4	2.239	
	LowGreyLevelRunEmphasis_angle135_offset1	.666	
	ShortRunEmphasis_angle135_offset7	.761	
	常數	.680	

a. 步驟 1 上輸入的變數：[%1:, 1:	
