# Supplementary material for: High-order radiomics features based on T2 FLAIR MRI predict multiple glioma immunohistochemical features: A more precise and personalized gliomas management
Source: PLoS One. 2020 Jan 22;15(1):e0227703. doi: 10.1371/journal.pone.0227703 (PMC6975558; doi:10.1371/journal.pone.0227703)
Supplement: S3 File — (ZIP) [file pone.0227703.s021.zip › statistical analysis/CD34/SPSS clinical Difference test.doc]

GET DATA /TYPE=XLSX
  /FILE='C:\project\hebeishengerglioma\数据分析\CD34\临床.xlsx'
  /SHEET=name 'Sheet1'
  /CELLRANGE=full
  /READNAMES=on
  /ASSUMEDSTRWIDTH=32767.
EXECUTE.
DATASET NAME 数据集1 WINDOW=FRONT.
EXAMINE VARIABLES=年龄 BY CD34
  /PLOT BOXPLOT STEMLEAF
  /COMPARE GROUPS
  /STATISTICS DESCRIPTIVES
  /CINTERVAL 95
  /MISSING LISTWISE
  /NOTOTAL.


探索


附註	
已建立輸出	19-JUN-2019 15:35:53	
備註		
輸入	作用中資料集	数据集1	
	過濾器	<無>	
	粗細	<無>	
	分割檔案	<無>	
	工作資料檔案中的 N 列	42	
遺漏值處理	遺漏的定義	應變數的使用者定義遺漏值視為遺漏。	
	已使用觀察值	統計資料是根據所使用任何應變數或係數沒有遺漏值的觀察值。	
語法	EXAMINE VARIABLES=年龄 BY CD34
  /PLOT BOXPLOT STEMLEAF
  /COMPARE GROUPS
  /STATISTICS DESCRIPTIVES
  /CINTERVAL 95
  /MISSING LISTWISE
  /NOTOTAL.	
資源	處理器時間	00:00:00.61	
	經歷時間	00:00:00.52	


[数据集1] 


CD34


觀察值處理摘要	
	CD34	觀察值	
		有效	遺漏	總計	
		N	百分比	N	百分比	N	百分比	
年龄	.0	24	100.0%	0	0.0%	24	100.0%	
	1.0	18	100.0%	0	0.0%	18	100.0%	


描述性統計資料	
	CD34	統計資料	標準錯誤	
年龄	.0	平均數	48.083	2.8609	
		95% 平均數的信賴區間	下限	42.165		
			上限	54.001		
		5% 修整的平均值	48.972		
		中位數	49.500		
		變異數	196.428		
		標準偏差	14.0153		
		最小值	7.0		
		最大值	71.0		
		範圍	64.0		
		內四分位距	16.8		
		偏斜度	-1.063	.472	
		峰度	1.942	.918	
	1.0	平均數	51.667	2.8029	
		95% 平均數的信賴區間	下限	45.753		
			上限	57.580		
		5% 修整的平均值	51.852		
		中位數	51.000		
		變異數	141.412		
		標準偏差	11.8917		
		最小值	30.0		
		最大值	70.0		
		範圍	40.0		
		內四分位距	16.3		
		偏斜度	-.158	.536	
		峰度	-.583	1.038	


年龄


莖葉圖


年龄 Stem-and-Leaf Plot for
CD34= .0

 Frequency    Stem &  Leaf

     1.00 Extremes    (=<7)
     1.00        2 .  7
     3.00        3 .  146
     7.00        4 .  2347899
     7.00        5 .  0111699
     4.00        6 .  0234
     1.00        7 .  1

 Stem width:      10.0
 Each leaf:       1 case(s)


年龄 Stem-and-Leaf Plot for
CD34= 1.0

 Frequency    Stem &  Leaf

     3.00        3 .  027
     4.00        4 .  3568
     6.00        5 .  002368
     4.00        6 .  0389
     1.00        7 .  0

 Stem width:      10.0
 Each leaf:       1 case(s)


Ò©êØÐÐ`6£î¥f¸îÝ»W«cd#322fù½Àh «Àj f¿Êå'´õ			/½ôReU¾*¡Uj$zñâE¹1«z§Wª÷ý½Àh «Àj ¦ªqªº|ûí·Õ;ÀRÊaÔoÝº¥))Õ²ª+qîÜ¹VçH2@V,×¯_ooo×®ùý~í`jZj^^^äX¶¥¥E®NLLHåÆééén·[)ËÖ¯_?3«_|ñÚûIn5Z-..VÇàÇU`uÉÌÌlhhÐ®=6ê@ü2ü°ªÃ"Jd²Yd²Y¬²Y¬²Y¬@VY¬@VÀ?þ8££ADàIEND®B`


ZNelÿ_kQQîÜ¹#kêëë±he¡g,<~ü8âb2vËzùÞ&''å«·oßV¡R÷7nÜX\|îÜ9Ûd3¼ÿþûó|oKºÄ^¾*ßÚãÇGl¦ìI²²¬>ùÛ]VV¦­KÖÜ½w¶ÉÈh±X¶mÛ¦­TËzmçêR M6I³ãââ6oÞ,+?úè#YàÀm,,Ô_eëÖ­²^þQY¯ÅrvÿþýÚU233Õå/_¾¬ª&®¤«««K¾¤¶K¿¥ò¥ß¡ªõúõëåêÒ]¹ rvÇÉz¬ÊY#@VAVUø?FçÉêÃÕý'NÈ©Ì|ê¬LwßWDç)«|õÓO?ÕN§jtHêtéÒ¥7äwÞÑÎÊ÷fµZU¥;&_;*·sùeÁívÏ¼)u±'OêWªMðÏú¯/õ*ú¬jS»¬ùüóÏeA=LVAVUF&ÅüüüYFU-ttt¨Ç~þæP¥z¬µ¿¿ÿÖ­[ª^×ÕR¡>Õ=n¬£¿Ì'|¢-ÇÇÇkËÙÙÙ!eïäÞ½ú["!!¡¤¤D.óþûï«Çµc«7oÞeÃ1ën±ÙlòÕ#Êrû²2--íõÂ;¦iÛ¢²ªeõûü<dxHÿä4Ê¬)óú.C§zV;°ÚØØ¨]Er+UÛ´i,KKdùÎ;²òøñã²üÍ7ßH=«=i¬®[·N¾+W®è¬Ïêk²aÃíyÅr@.¦%¤dÊP(áààà¬»E*>ëÓ æyb×R¯¢è2Cë/¬¦Uý¦UU`9þ¼zÒ¬Ój+UDå48M´¾ªóý.Ò¼c¨*3ÅÅÅfU®qòÌ33/Y__¯n0bZõx<ÚedPuFÊ¤;ë¿>ëú(®¢¶WfÙÌÌLm[dZU~¿¬¬«æ´gÖÌÌªOõ¾qãK¾úê+õÔßiúy7âÑ¹¦UíyO²¼÷î§¿9:«¸®|úÑÕ3Í(µ|áÂ5­ª¬ÊÎ;åtÇ3¯¢.?³ë×¯×¾Í"¯2kVÕ÷ £scc£ªÜ¿ÑNUU`ÕxòäÓéÔ9íh¥ö °þø¥P¯EQYÍÊÊîÖÕÕi]ùæoÔÍª¡p×®]óL«ú¬º¨''G¼odR·Ú·¤^$Ë'O³ÇVÕäúêþýû#÷¤¿¯Aê®=+ÿDii©ÜoP«mWDVs¹²*wMd+l6ºÙþþ~9UÇ¼É*È*°h-ÔÿA`Uõhí;wÔdJëééQGRåìG4ßïÒBYøð¡z²qÄµ´é3âF"¦måúõëRâ§º£²¬®,ë	Æêm2å»ººÔ$ªèßéi9WUmôW+É*È*°F,&«·oßVzXõá4Uõ.HógUlU". YÒÞfH-ý[ÎÌªÔHùP(¤Þb)¢âê½eA¾ùCÍõM2¬Ï|oùß[qIWÑgÕï÷'%%9µCÔIíÛ·¬¬kAccã<YUôÅ~xáÂ4-¥*Tú·O5«ú×­joa(7bµZÕíëßÈw®iUæcù&åøüóÏ#Vï¥^Æ:ëû)ªÃÃê0sÇÏÈÈ³ÙlõõõêXìü¶ø«Dduæ·÷ÞïUU`-PÏ¡+«<èïï×ÎJ"ÆÄÅ¼Ëölá§5©'+ÍúÜí	MÚYKUÐ·ÔÞ1??_?>jgUg	Ð¬&''eU!/ÿ*j¸ß¼yóoµ¿ÿ~þO¬«ØW_µaÃýR÷jÚÅ§ñå¡£V¯3ÕØU@V «UÈ* «UÈ* «UÈ*dUÈ*dU¿uæÌ¤¤$íìéÓ§SRRØ-YVÔýû÷KJJ,K||¼Ífï½÷=zôtúºYïp8:::ÔåoÜ¸±ûöuëÖ%$$ìÞ½[®®ÝÔäääÆå*Q|;wîõòÍ§ÉÂãÇçü`0>|X¶°°/@V#ÑJ6:::55ÕØØ(5Ú±cUK¯×'A³»ví¦¦É<vk'NÐ®µx7oÞëeee²^¾±úúzYxÿý÷g^¦¿¿ÿ«¯¾¯^¸pANÏ?çÎYØ²eË¹sç¶NãgUà¹;tèä§¥¥eÖáOë,é/ V³¬vVòEVUÿæº¢úÒÃ<x ÙÙÙ3/#´Ì²Ú­©ïVJ¬Ö:ujÃü¬²<wN§S4ë#«úÎÉÀ*Ë©©©ú?~¨³§OÞ·o_Yõù|*³^Qz©­-ódU¦ÕË/k_MJJ5ÆÈ*ðÉ¬9W#:'Ë			ÚY»Ý.kd<9R­ÉÌÌìïï×_Ë0yfÖY¿§¿Am8'«W¯^½yóæ¹sç´¬ÊÈËÏ «ÀËUÒ¦¦¦êêêdåÞ½åìÅÝn÷<uxyY/8p@R*W®.))uëÖåææò³È*ðÜmÞ¼Y¹¿sêA`«Õª¿:¶ªFØmÛ¶=6âZÏdZâA`u¨Xj¥ÖU¬+A=ÏöÌ3ówN¯WQYU/]|>Õììl~±È§,©¬Þ¾bbB­Ñ?]YlÚ´)--M"$<yò¤DÈëõê;'ë>_\Üõë×NCAðÚµkR/¹À|°:.5«ÚÙ?üPê§ÍõY³ª¿)µEÈ*ðÜÝ¿¿¨¨H²/nóÍ7úTuéÒ%uù7onß¾=!!A®"Ãî"Î¨³úèÑ£ùç¤å¥¥¥ê­*ffuæ±U¹ÇpöìYí¬ü ²`QºººÁ Öã/ªå	9+ã8» «UÈ* «UÈ*dõ¥óÿøÉÉÉV^´ïï===«;«ÈÍÍ½	Àö£ýèßþíßVVwìØñ5/Úüc²Y%«²JVd¬@V «d@VÉ*¬UÈ*YU² «d²Y%«²JVdõ%ÌêÕ«W333ãããNg__YÕè¥¥¥]¹rEN:µyóæÿýßÿ½0ÃÛo¿MVduiÖ­[÷í·ßfóÖ[oñ³ÕÅêéé9pà,<á¯þê¯Vdu±>|èóù=zÄ±UY]»wïîß¿ÿÞ½<@V%nß¾ýþýû¼À@V+55UÿÔ$² «¼¬UÈ*d¬È*Y¬@VÉ*¬UXÓ&&&Ø	d°p¸¼¼|Ï=ì²XP(ät:Ødýê÷ûF£ÏçaU@Á Ãá!µµµ½AVQ(//!µ  `llBVQêííUC*GRÉ*à©I%«T²JVà¹*--ÍËË+,,çiGÚêñxRÉ*`mmm2zJ/ÛÛÛÕjåB2¤Z,ÚÚZvYÌ>&&&655ik¥ÒýÅ*++Íf3C*YÌG½/RÄÊ#Ghp¹!¬ÎjVVVÄJ¿ß¯²ªT·Û=00À¾"«¨;;;µ5cccV«µ±±Q©@£!«Åjnn²ÖÖÖµ¶¶:Î-[¶ÈÓÛÛËþ!«d&æçç§§§ËPYYÉJVÉ*D/0¤U²Ë544k4ËËËRÉ*Yå©!¬U`H%«d^´ÚÚZRÓÓÓRÉ*Yèx<Áà÷ûÇÇÇÙ!d¬@ôCªÅb!Uÿ «dftt!¬Ux6CªÕjµÙlmmmì²JV ú!ÕçóÉZPPÀJVÉ*DO½ßorr²þUAVÉ*,ÍØØXIIÑh!UÙ!d¬@ÚÛÛív»ÙlfH%«d¢7>>^TT$Cj~~þðð0;¬URKK©I%«d;¤úý~R½^ïÈÈ;¬UR0LOO7Íì²JV`YCªÉdr»Ý^¸´´Ôn·[,ËÅûBU²¿ÕÝÝít:eH=räÈë&z½^õ<&keÌe7U² ÖI#ËËËFãbT¥®®.''G_ßÚÚZ	-;¬U1MFÌ¬¬,ijUUÕâ?Üï÷6[,ö'Y%«bzH5LN§³»»I×-++«®®Ö¯KLLd¯U² B!Ë%Cª2/õêííí6mttTZÏÇ%«d@Ì©2hÊêñxy$uV%%%éééuuuØââb©,¯p%«d@Ì©9992¤ÅIKSSSAAA^^ª¼µ!Y%«b¤Ôl6gee-õH*È*Yßp»Ý2¤VVV.HY%«b×#GL&C*Y%«°,¡PÈãñT²JV zUUUf³Ùn·wvv²7È*Y(©!µ´´t||BVÉ*D©¡¡Áb±0¤U²Ë2::êõzRÉ*YåjkkKNN~~Cª4'=U²`íöù|F£ñ9©Öiw($«sºÿ~jj*Y°z555=×!µ½½]j*£°,Ãá²²²ôôta&«³èêêr8¬XÆÆÆ÷TË%åÖ¯ñz½´²úkÒË7ojYúñ)))o¿ý6?K/`0hµZWàé¾%âjmmmqq1?²:;VKgÈÈÈ «^*£££êHê=VàÁX)÷ÐÐ~MEEEYY?²º@VyÀË¯¥¥E:g69²2ÏË#//Oû·å;?²JV¬b2JádHu»ÝËùìñ¥ÃUi¹L¨EEE¥¶¶Y%«V±`0¾CjÖÖÖªªª@ °EYgL"ZQQ±òC*È*o`­ioow8/pHY%«Öp8ì÷ûM&C*È*,KwwwNNC*È*,DTRÊ²ËN§4µ¢¢"bH-++ËËËÛ³gOCCû¬UO 0ÍCâ:3·ÉÉÉÅÅÅmmmuuurÞ;¬UsHÍÉÉ1ååå³¾¡Ûí®ªªÒÎÙíöÖÖVvY%«ð;¤2¤JVg½@8Q5â1áêêêÒÒRöY%«ðÛ!Õår©!u§ûÊüjµZ#.PSSSRRÂ>$«d~M;:×ªg·Ûõx*KYÙd¬uCCC¹¹¹aþ!U¯­­-99¹ªªj`` ³³3//ÏãñÃav&Y%«RÍéééKýìñP(äóùdº9µ¢¢¦U² ¦¨!Õï÷¯Àg¬UkVmm­ÅbbHÈ*üÎêñxdH-**bHY%«;¤¦¦¦¶µµ±7@VÉ*èÔ]»vÉj2^íµÄÄÄââb>d¬X²äääÌÌÌááaUÙ¼iìU²`±ÆÆÆdHÍÏÏß¸q£þ`j8¶Z­ò7½²JV,¬µµ5y,477ïÙ³'âRÓd¬XìêóùFFFdüís¹5¼Àd¬O»RõèÄÄÝnÚÚÚZÍ&f¬U³ÃEEEF£QRõB¡t4//¯¼¼­Vkww7;d¬HyÔb±È:×eÆÇÇªªªdeNY%«æR=ÏÌ! «dÀbAÃ1ÿU²`ÿñÿýï|'%%¥¾¾²JVDé¿üeÜ´¿û»¿ûì³Ï¬Vkyy9»d¬X	)¨©ßÿþ÷ÔÊááaÅÒÛÛËþY%««»»Ûáp$$$üþïÿ~8Ö©¤¤äÈ#ì"U²`Q¤f³9''ç_ÿõ_­VkÄçÏøý~²²JV,,IMM&SUUª©Óé¬««Ó.066&¡ì+U²`!Õb±deeéßI*+¥²mmmRÙââböÈ*Y0§ááa·Ûm4ý~ÄTUÖüüüôôôÜÜ/I²JVÌI&QGÏïY%«¢166&S©©2ª!UÿÁãY%«¥¥¥%==Ýf³½úê«¯¼òÊ~ð>^d¬Fbbâ?ýÓ?åææ­[·JVSAVÉ*h¸¿ýÛ¿²ÚíöÎÎNYãõzõ9U²`QF£©%%%ÚZ[[Ëf@VÉ*¥ijj²Z­ÕÏ>ûL¿¾²²²¬¬ý²JV,ÊÈÈ×ë!µ¨¨H~¯eYá©zË$õh0@VÉ*´¶¶&&&J;ÛÚÚäìøø¸kZMMMUU¬¯¨¨`/¬UI´¸¸Øh4îÙ³gttT[/£j]]]IIIii©D²JV, ¥¥%y,°7@VÉ*ß½^¯ÃáÈÍÍÿõ0ããã2ÊêóùôC*@VÉ*_knn¹³¶¶vhh¨µµuÏ¯Z­ÖÄÄÄöÈ*YibbB2©²®zú®ü¸ßï!UÚöÈ*Y0P(±RzäÈílzzºÙlÖ¯È*Y0KVNgÄÊÒÒRUÐp8,5LgxxÝ²JVÌg®Á ü±Ûí%ðÑâ «dÀ¢477KYõOYúë¿þku$Õív°@VÉ*%Ù4???===77÷ïÿþï:Ê²JVDI"ZQQa2²²²RAVÉ*ÈêñxN§ÏçëííÕI:#C*GRAVÉ*y½^ËÕÙÙ944TYYÔªªª$¨2¤F´ «dÀ,G8ÖÖTWWËäÚÝÝ-­5Leee© «dÀ¢D¼ÃÃ×Ó¯I	UÃTU²`iYñT;äïÁ`!U?Âd¬XþAàÚÚZSSSS·lÙÂVAVûúú¬Vk|||FFÆ¥KÈ*ð2ðz½o¾ùæ~ô#RÝn÷Æµ§,dõ¥ÎªÏçkllÏ?ÿ|÷îÝ²Ð3ÃýÙU`%UWW÷»ßý½ßû½?ú£?_Òîînö	°:²455%©©©ß~û­a6o½õ?K`JG9¬Ö¬ÆÇÇG,ÿ¿~úÓ2­+ X,ÍÖÖÖÆÞVeVãââ´å­/Äøø¸Çã!µ¤¤DÙ!Àeõúõëi>¯¥¥E~	µ5jåáÃeýµk×s7nTË2YV^]]]rr²©ú°Yø©C¡¿¾	Ál6ß½W­³îÝ»·¾¾^äTªLVRóóóFcaaáèè(;Xé¬ª|öõõÅÅÅ;wN­Y·n,ËúââbY¡3;;·ÖÕÕ"·fµZ¯^½JVç¡¿¿ÿç?ÿ¹üùýþ±±1µ²©©É6­½½]¼°¬ª²ýÙ'Nhg7mÚÁÛA/cÇ½òÊ+¯½öÛí¶X,ßýîwåîoAAº+¬UÀÈjGGGKKËñãÇå²±±QNeîÜ¹Sý¢ª±uÿþýdxIæÔ¸¸¸_üâÚþðRÙ7Ê´Êþ^pV?~,§=vÞºuKN/_¾,§gÏÕ.c³Ù233É*ð2øùÏ.sªZ/,,4ßùÎwäw¼ø¬**«ÃêAàM6É«==ixIüéþ©Ûí`0n±XÔSkjjØ9ÀËÕíØêÉ'VÐ/~ñhQQ©^¯wddäúõë2­ö÷÷³s7«Rý´ZPP@VÁ?ÿó?KD_yå_þòrVúøiiiìàåÍêùóçÃá°:ì:88(w<x@VK~+ý~¿©.Ël6ÇÅÅÉØ*µÛí¿úÕ¯Ø?ÀKÕ>yòäÞ½êÉJ3_r£,e%«Àt´¶¶V­ù÷ÿwXyìx¹²úÍ7ß8qBÂ¢oªükg:¤'LV8¤ºÝn¹Ë^ê¬*Úc¼ò,½páþ«2ÎÊÊóçÏU`õööÊÉ&&&Ø!ÀêÈê#«À$¢åååjH`d¬©@LfÕ`0|øájA½«~WWÓé,,,<wî*¯[VC*°²zøðaµ%gÏåÛ·oËrKKÙl&«Às244TZZçõzøÃ2¤«8«===ê¤¦jÁçóio»çÎÕ¤¤$²<ÉÉÉ2¡þìg?KHH9õã?f·«5«Ú'oØ°Aeuß¾²Fp6­Uà9±ÙlÇËÍÍ!µ²²²··×b±ð	äÀjÍªö ðÑ£GeÁl6oÙ²EËªL®²ÿÎ;ïUàHIIß»	ªZ'÷eÙ9ÀêÎê¬Ó*ÏÏÐÐÜåWdHÕIÝ³gOss3ûXSÓªJì½÷È*ð<ÔÖÖÊ¯[vvö«¯¾´õcccE¿ÀZÈê_~)+xx¶FFF<Ñh,//!5X­ÖöövùÔÔív°ÕÕË/«Ç¥¦²P\,ËrgùÝwß=uêºÓ*ðTùýr8ÚTÑÔÔät:M&Ýn¯¨¨à¥5À*Îêää¤ëùóç<yråÊm~ÝµkYÓÊÖfVúT8Oe9ÊòéÓ§å4UàhhhHNNR¬Á¬Þ½W*áÔÖôõõÉsçÎÉòèè¨zX*0¿öövõ1ã6­¨¨èÚµk>!¬NNNöôôÌ¦Ú[·n©ù¬¨MMMRÐááá?ù?ã7RXVùà²Ûímmm_O¿N¦  @î¦¦¦=z=U²,Íøø¸ÍfûzúqàäääôôôÎÎÎ^0ÄPVzzvNóMkùµ¬^½*ËdXP8þ?ø¿ø¿0~¿_*++«ªªJKKÙ9@¬dõÉ'êÝög¾5Ó*°$Á`Ðd2mØ°ATµfllLæ×ÖÖVv+YçÈ*°H2KSò¼ñÆùùù555V«UNÙ?Y%«ÀbÉ/¤ÝnOLLlhhøzú¡à@  -++ÓÆV1Õ©©)íí "²ªÞ¬s©2¤z½Þv@VgPÕ«§,©e²ÌÔÞÞ.CªüÔÕÕ±7²:_VyH-..KKKûË¿üËêêêÑÑQv@VÉ*°dÁ`PTÉd6ßï½ÚÚZÏ'kÙ9Y%«ÀbMLLTTTHPß|óÍ¤¤$ù=Ô¾TYYË.È*Y¥»»ÛétÊ¾øâÂÂBýWÃápbb¢²£²º@Vå:YCª©n·``@ÖTWWEf³qõ¬vttx½^õß¬¬,íÙ¿Úr½'«å!UZYY©¬[[[ÃáÐÊjµ²»XÏêãÇy«`.R5¤ÎüX7Y¹gÏ±±1YBÞöëYålYÉïXNNÙl²ÎzÄt||h³ÙäT.ÆNÈêoÉ3gÎ¨÷T²Ûí7oVo±d0.^¼HV;&&&ü~¿ÑhÌÊÊ1tÁËê@VÿÏ+W$¢ò7")));;[=wIâÇ´RN§4µ¢¢Xd5ú§»øøx9]·nÅb9zôèñãÇÕg¯JhÓÒÒÈ*baHÍÉÉéîîfd5Ê¬Þ¾[²Ú××7wwîÜIV±æÔ²²2T¬.+«ÌmÛ¶Éz6ãÞ½¦;wÎl6<xprròÖ­[<µªªªJþ«¿öÚkv»]¬a·du¹ÇV;::$±úrlkÛÀÀËå!õÍ7ßüÁ~¿~jNNNÄ( «QfuçÎ¾iê!öïßOV±TPõo$Ëe1O@Vç|(øý÷ßÓ3gÎÈß6;wNÎÖÕÕ-xä¬b5©òÛï÷777ïÙ³'â2²¦©©Õè³úé§ªeÍ©ÿÄ¬b-©éééê!_ÑÙÙ9óÃg<O[[» «ÑgUkgJJJVVþK§N"«X3Cjyy¹©Úúp8ÜÒÒ¢­imm5¼o>@VÍ´ª½ÃáPGXûûûÉ*Vµ@  TIæÌ¯ÊÀXTTT[[[RR"MeTÈêr³zæÌ¬NMMÉú­[·ò%¬^###yyyò?Yz©R#WUUIY+++ØoYfoµ¿~ýúÝ»wóVûXêêêdµZ­íííì/&«|ÖÒZXX8ÏdX@ss³©ÉÉÉ²ÀÞ@V(É`Z\l4µÏ²D£½½Ýn·ËÊ;9 «---~¿¿´´ôÆORóòòx½)µÕû÷ï§¦¦UÌTTTd³Ùª««@VVV~~þò?-ÊØÐÐÀ°Ö²ÚÕÕåp8YEæææôôtmÃ¹¹¹Qß $Y®n2<ÏÈÈÀÌªôòæÍZV§¦¦þ|M6½ýöÛü,cMaaa]]]Ä ér¹¢»µîîn§Ói6kkkùìqk6«ÚÛ6iYýé¯¿þ:YAùùùo844äp8¢RÝn÷ÀÀ;@e¡è×TVVÎüP¶E©555©ÖfV¿AV1ÑÑQ»Ý^VV666«««?¤ÊUdHu¹Æ8¦U²_¿£~~~â4Ç³ø:Ê%srrdH­ªªbH@VÉ*¢$-// r$@ìf·À3!CªÔÔd21¤ «dÑ©2¤:N¤ «dÑëííu82¤r$Y%«Xòòr£Ñèv»»»»ÙÈ*YEB¡Ëå!µ²²!Y%«:êp8ÿJV «d³©F£±¼¼!Y%«^ `H@VÉêj566VZZUTTô?ôhh(77×`00¤ «duµ65==½°°°wZqq±Íf!TªTùf:;;ù¹ «duU*))¦ê×HYef]ÉïA*®T¿ß?>>ÎY%««UNNNÄAefÍÊÊZ±o ¶¶Öb±0¤ «dud5âA¡Phe²*CªÇã!UcTd¬®/êAàäädÍÖÖÖÆOY%«kÄÊ?eInÜçóÉZPPÀ¬Õ5XVY³¦ÉúªTÑÚÚÊ@VÉ*¢·§jHev²JV%MRU²g6¤ú|¾ò.@V±F´··«!µ¡¡½¬UD)FTd¬b¹CªÝn·X,µµµìd¬b¹CªÇãaH@VÉ*¢C*²JV±Ü!µ´´TT·ÛÍ¬UD¯··×étÍæ@ Àg «dQ9rDöøÀÀ;Y%«R(ÊÉÉ1Láp¬UDIûìñöövö²JV¥¡¡¡ÜÜÑXZZÊ¬UD¯®®.11Ñf³uww³7U²(äåå¿ßÏg «dÑ;rä©ééémmmìd¬ÆõAlV«UBèv»åÆrnmttÔçó©ÏgH@VÉjlp¹¯wxxXkjj,K0îÖ¬Óxº/²JVcQ]]dUÿVGRVY³ÔÛ*<Rd`eÇ «d5ùý~éhÄü*ën¤µµ599Ùf³É»Y%«±«¬¬¬ªªJ¿FfÍÄÄÄE^].÷ìÙÃ¬ÕX×ÞÞ.¦þ3d|>ßb®ÛÜÜ,¶Z­MMMìId¬â×ÊËË¥555n·;''gÁ¹sll¬¸¸!È*fYËÊÊJJJ¤¬¾¿ Ì¦2àÊÚÐÐÀ®²(IqeH5©@V±,mmmÃb±0¤YEôÆÆÆÔÓÕ»F°C¬"Jííív»]Ôºº:öUDi||¼¸¸Ød21¤YÅ²Èÿ5¤ý[È*6¤Æ¼¼<T «XÖêp8Ìfsuu5C*UD)WTTÈêv»Ù!@V¥`0h·ÛeH­ªªbH²(IDeH5Lg``dQÿYYY2¤öU²½ªª*	jNNN(boY%«Qêííu¹©¼¼|ÁÏ«UÌ©²²Rêt:R¬ÕèåææÆÒÒÒññqvU²¥êêj³Ù,Cjww7È*Y~Hu¹¤óT «d5JÑÄÄD¤@LgõêÕ«ñññÒ¾¾>²º!Õï÷3¤@Lg5--íÊ+²pêÔ©Í7ËÂ¯føÉO~BVçR[[k±XÒÓÓåÇÌÞXÏªÞºuë¾ýö[ÃlÞzë-~FGG½^/O÷²:ÈÂµvíÚÅ´¡µµ599YTîd5ÒÃ>ß£G8¶º ¼¼<Ryã$ «OµvÕÙ»wïîß¿ÿÞ½<xAMMM2¤Úl¶ÎÎNöÕHÁ`pûöí÷ïßç6óÁ´  Àd2p$ÈêìRSSõOM"«sðù|©@Vy;Y%«UÈ*YU² «d²Y%«²JV «U² «d@VÉ*d²JVd¬@V «d@VÉ*¬UÈ*d¬È*YU²Y%«²JVd¬@V «d@VÉ*¬UÈ*YU² «d²Y%«²JVd¬@VÉ*¬UY%«UÈ*YU² «d²JVd¬È*Y¬@VÉ*¬UY%«UÈ*YU²Y¬UY%«²JVXcYÌÎÎÏÈÈèêê"«²=©éÙ³geáÊ+IIId@V.8Î©©©3lÞ¼ùí·ßæg	 «òäÉúõëÃ©S§$«oÏð½ï¬ÈêtttlÜ¸dui¿±>>>¬ÈjôÒÒÒeáêÕ«Û·o'«²½¾¾¾S·mÛvïÞ=² «¼¬UÈ*d¬È*Y¬@VÉ*¬UY%«UÈ*YU²Y¬ê¼úê«©×_Ýn·oIo¼ñÆ÷¿ÿýÝöM6Åæ¶§¥¥Åì¶§§§Ë¶Ëiln¾l»üôcvÛå·þÙÞ¦ôhÕguddägÍív;/bÒ;ï¼Û¾oß¾ØÜöÒÒRÁðÙgÅà¶:tH¶½²²2·=È¶<x06ÿÛËïaaá³½Íÿþïÿ^ÝYþæoþæ­·ÞÍmÿø¹KÛ^__¿~ýúØÜöÎÎNùóú?ÿó?1¸íÿùÿ)Û><<Ûþõ×_Ë¶ÿË¿üKlþ·É²±±qeþ-²JVÉ*Y%«d¬U²JVÉ*Y%«d¬U²JVÉ*Y%«d¬U²JVÉ*Y%«du!|ðÁÏ~ö³ØÜö;vÄæ¶9s&3336·ýÊ+¯¿þú£GbpÛõ«_É¶ÿ×ýWn»Üm¿zõjlþ·ÿã?þãÖÖV²À*CV «ÕîêÕ«ñññN§³¯¯OÖÈ©Õj5.]ZÃÛ>88­¶´««+¦¶]éèè0þïÿ|ìlû½÷:1µí?Þ¿BBÂ¦MÁ`¬ý×ÿÜãââbjóåÇíp8^È»ËjZZÚ+WdáÔ©S7oÏ§!öùçïÞ½o»üg:öìÓé'­$%%ÅÔ¶'OÈ=*-«±³í---ýØÙöCòÉ'SSSòGÖn·ÇÚÿyl²loLm¾ü»û¶,ÈijjêJnL?¼nÝ:µ÷å·N&''ÕÞ_ó. Ãz¬mûÇ|øða-«±³íÒTuwJÿ'F¶]îJ^¿~=6·]3::ºmÛ¶XÛ|¹u÷î]YSujÅ¶=v³ÚÓÓsàÀY×Vê×êÄ¶~ýzIë1µír5++K~©´¬ÆÎ¶§¥¥mß¾]¶QöÀ7bjÛeë;&w åk¬ý¾+^¯WþÜÅÚû¾¾>ù¹Ëï»ªÍ_±mÑ¬>|øÐçó©î©CJBBB,l~GGÇÆcjÛå/ËåËNmRkbðç.wÛÕ£±³í²¥uuu² ÷'ä^EþÜoÞ¼¹eËýÍÏÎÎVTH_ÕX±mÅ¬Êýû÷ß»wOÀLNNªGTlbº³;Ûnø]1ûsWMbgÛõ[kÿçCÉ¼®ß!1²ù3gÓÛöËj0Ü¾ûýû÷µ5÷î­¯¯:ýv2Â®ámOKK|:ýthÙ	1µíú¾ÆàÏýÖ­[êe¬ýÜKJJN>ýtúið.+ÿÏoÝº5Åà;PÕ»k×®ÉäºÛsYMMMZºººRRRâââ¬VëÚ~g¯¾¾¾¹ã¶mÛ65¬ÇÎ¶ÏÌjìlOOz¥ÛíVOâmðà×ëUÇoÞ¼ÿçÔótbí¿ýÈÈz=¡ÊòJn;oY¬@VY¬@V «¬@V «¬@VÌââÅáñãÇrúñÇË6ddd´´´ø|>9õówÞ`På2õyïï½÷:ûèÑ#9«>¸YÖ'O;wîÃ?È>ZN?úè#9µX,Á`PÎ/¾øB;ëp8²³³¦ÉÕÏ=+Ø¹s§¶æÓO?5<Ð®ÒÕÕ%k´OùPY=sæ?¬k©9I¬^¸páéo>¤]?­j½û¶½sçnÛ¶M­ÔÞ°ººßïX#õÈëõÊ~ùå²fÇ²|òäIYþüóÏùYdXõÒÒÒ¾úê«õë×Ká$õõõGÎÉÄ©â©S§Ô²ÕjTÅU®%³©,È0¡FÕÆÆFYsíÚ5íêj6«5>5RYö?@Vµõk08qBÍ¦êðçiô4RR?­j×RßÞßß/¡PHNÕÇzËBvv¶þÂªÓr;²¬J|üøqY£Ý¸t@VµàüùóRDu5OLL¨æÍ:­ª³/_NMMUåJeå7RRRvïÞ-úÛçwÔãÆêº^¯7¢Ðr;ü ²¬Uâª¦®,çççÏUigOO¬¹÷®:,ªXUÿÞºu+âÆåôâÅÚ«æ`mZa@Vµ@%=zôèúõëëêêÔìo·Û[~Cÿ °Lª×¯_S¹Ø¹iêIIj!))IJ955%7.Ëú0Ï:­êGadXÅ>øà69UÓçÈÈöõõ©5ò%³Ù¬=ÛHQMâFÜ¤ôÞ½êIÂO<ÙN¦U¬kV8¾ÿ¾zªg©#ªV«5âZê®÷îÕê¨odZZÚ,V²ÄõêÑÑQ9­¯¯×ÖÇÅÅmÝºUJ©²*_Ðª5ö÷÷Ë©Ì¬ê^Uçü»À´U ¨ª9U¾ûî»ê`)®dOå0â<x ))Õ²ªÞVâÒ¥KKVçI2@V¬wîÜ9þ¼vöîÝ»Á`P3õ²Ôüü|ý,æÌ9;11!9oÚ´Éãñ¨/ÃaùÒffõË/¿TÏ~ËGL«¥¥¥ê=%øqd-7onllÔÎ^¸p!âøeüXÕÛ"JI²Y¬²Y¬@VY¬@VY¬@V «¬@V «àéÓÿü·eí©<IEND®B`


糾正常態 Q-Q 圖


O<	z»o½õzûöYG+++5gtttâÇ²fÍûÆÆFÍà"õõõ·ØÓÓsùòå	n%::Ú~tÿþý/Âî³ËåZ¿~½|æYâß@V1÷ÿò"".Þ©3777ë0??_G­äÎ=öLñW­Zå4EªãñãÇ=ÏxjxxXçôz½¾ÿ¾¦«««íYU¬»aåjddDGÕ=4«¥¥¥Ö[ZZÌÌøøø²²2ë©´w÷;¦3tvvÚ³:Þ/¾øB'éAµ···Ö£ÓMöÙg,ÝI=êc233µ<']S¿ÈÃuôîÇÜ«+V8jzíÚ5sRVVÿk «W®cÇ ââbÇ«¶Þ¼yóÈ#qqqæå¾««ËÌ?qâ5løUbëØ0å«ªªz~GVuÔ1~-))Ñüo¾ùÆ~)H3Í¸Pæ~êðí·ßÖ95ñé§Zg~ôèÆÊ&;::t¨ß=&hÔu=z,111ºN3§¿¿_­Ý±c¹i~üñÇã½±0£sM´ÑÄãÇg»qãæk9«ñ:õîÝ»&TæÞ¨èEEEyÐÑ¹è¹`ì?ð"æîy,©¿²²ùÂÈì#0EkâfEGG[-yúý`Í·F±\¯ÚÉÉÉjfu®éÐ¦M¬!ÎwiãÆÖI&Ìf´g®ÓívÞVOO/`ÎsçÎSåÈÈH½3pYe5g;pàÀÓ±¹¥©ýºÑÜÜ5+uW­·#oVÍzñâEÝækûüóÏåÊ:³ 5Ä×Q=@ëá/]ºÔ_×fª¦®Ò¥A¶N2Äþ¬é$ó`L­-Z¤«»ZnÐÑ	ÖùO÷"¬êÍV­æè!UUÌ¿¹NUÇ¼P~õÕWöh©S3,3Á ¬:õË/¿´¦½^¯y]Ök·ý<|òÉx£U3^´¿Ê;nÑ´ÓL÷ööa«@ò4~Ò3!!!èÝ3Y=LåDmË-«éaÚ³ª±£Yÿ©A°	¶Î ªÖÍ¾Ù<ÒæææI÷ÞÏ:ªå¬o*µÿ~ª7C&·zk¢ÇnßflgÎvèÐ!ûLóôéMLÐ[îEìYµÖ@XÑ¬&« «4RTl3Y5c8ÇÎÍºß§ßoÞ3ë';::LcÄqYëåÕÚ'ÈZol^íçùâ/&ÎªÆ|yyyOÇÖ»Ú;úôûÕÚ&Ì0gpì/£Ú¸eªdöÀµÒUC[AfÐlÔÛ)DõØ:ÕLM¡;fUÀÍz£ ¥ª;l¿«º?^ë<ª»YWlm[íîîÖtzzzÐ§8))I§:¶:+1óÜ¹skÆXÅld5ÓfcÁÄk²9Oý3EÑLcó"¨A§YÑjmXµ·[³­æ'''kZ¯¿VK4³¢¢ÂlãTëëî@ëÈªÆº¡½ÁYu¬¶ÅºÝ#GhÂ±eQsRRRÌyt÷McU¨Ì½µ¬£fÏ#³ÅWDÃ¾æ¹±±ÑÚeIÃYO£FeØdÕ+E­®®nâå0ÞCkki½ÑÙÌ(ÙlÈÔ P#ÂÀuÚ*t7¨	vRîE¬ZDAýVAV1¿=ÖÈVLiL9ZÆX»=[ãj6¤MôW¡N8¶¡æ¢¢¢©VÍ´n1è~°zÇ ÞkteFH:af^ë¦#uëÚì~),,tlôÕuªc%uÅ<Þ­ûxËÁ±2hÍÊg-Çh5;;Û:ýA×¨êÐ[:1Yf=¥KZE£U3QRRBVAV1µ20«öÏw3wuuéU^§N2»þf±wkÇ­Zû=izíÚµÖ0ÔÚþU]D÷V×¯[×hâÛ2ÔDeeå£Us¶¶63Ì2£R³êÒ´AÑ2cq«÷ýýýª Yc¬~+lë!8öæ5Fzß¬©Ì±o]¶R¢Ì´1k´j%'''ð³³FBB9`;-ZdÝË/4«æ>hè]mYmn6UU¹'Ox½^kdã¬ÀöµÕdÕçó©»fÏXóZl¸Å¤Þÿý	F«ö¬fff:Î`vN¶¯³UV].×¡CÌÓ¨ÈìõjÎi%ÇÁ|BÆsÒÑª¹*ÓÕÚË÷ìÙ££7oÖ­Oª+ Û©é®ufj¿ûØ×Ê¤YòÖyÌ§4­Çh6;Î 77æÔ7:öú½2o­ÕMè±è=yâ¬çÈÕ©¼¬jÑéQhQ«5OYÎddáÏñAÏñF«C[³¶öþýûf#lZ[[­mÖ¾²3ËªÆfgcståÊf"%þïÀº!Z½Û¯A&ó^ÁÚ=xÒÑªWieÆ¬Ö|³·¤¤Dï*ÌGHíCµ/@³sß+ó1ÙÆÆÆÀ%`+`_ 5ÆÍ*ñSÛÖM3ª6÷D6ë®î`lÖÒëÝ®ùòåËf$jö~f	ÌªµÃÌ$« «×¦U%ÄÌªÈGcÌ+¯µGÏYµo[µoª4Ì÷YÌÐ×'k½±Yª!£ÙÜkvu¼Ö[t¥ªª*=p3Ô[3~5oÌ¸Íìãøèãq9>âbîXàÇI_ÓUëÛ'ó!óìïHÌ®UÐQVV6Þ74mØ°!ð»~OäÌ.bÏªÞÄÅÅ¥§§'×ñ×¯_OVAV1ÛxY5/¦XÉRóânÿú¤ ù±nÕzêJ<¹~ûß^ÁÒÊ­`9¾àÉþ¡ °±©µîWg3+~õÖÁ¾wqGGytºéÀ=ÍêYÇ&ÞÈÈHkðÄ£UÓlÖµ¯62-úÝfS·ùÌx­¢¢"##Ãår%%%éQm±?MS¿#«woË-ddóÙït¼¬>|øP±êÕÜ1´Ê·,Y;:dvVº¿£IÀ9æ;¾1Ñ¼ÖfÕ|Ã°Ù¿×|ìõéØG?í+~5òÖy4bËÎÎ6÷GãZRã0³:ÚþIÈsóæÍÒÒR³vÚZíxfç,ë¨¥)¨ãumVþsssíÃGë¨ÉðÄ¿`§û¯G7Þ·*N÷"fEEZZÚ¤_µoä «G4hëÂñµÃí¯ö¯e[uó¨MÔ§88³2xêi|qi>=ñÃ4ÃV¬@V «¬@V «¬@V «U@V «U@V «U/®ººº¸¸8ëè'X,Y¿ÁÁÁâââèèh·Û´eËááá§c¿¡mh~zzúÌù»ºº²²².¹víZ]Üºªøøx]df÷D·»yóæ¨1xüøñ¸ÿööì±Ý°aO%@VçL%KÓÛÛ;::Z]]­D­Y³ÆÊªe^^ËåRPuôý÷ßWSÛÇèÙÙÙÖµõÕWÖ¥f ´´TÕ¨ªªÒÄ§~xS§NéÔsçÎéðìÙ³÷ïß×Ä+VáiÈ*ð|©IµµµAGV [[[5]XXh?2lÆ²ÖQåùdUiÔe=zôðáCM,[¶,ð<4k,kÝCsÇTb3çøñã111<­Y¯×«,]Ýj¤¬NLL´¡¢¢B3ÌÑ'N¬_¿þdU½´.«	+dU£Õ/Z§ÆÅÅ1²ÆãUÐHMGFFZGSRR4GÃS.Í¥KvttØ/ÌwÆårÙ/k'Èêµk×º»»¬¬jÈËÓU`dÕÞ¹ÑÑÑÊÊJÍÌÏÏ×ÑÆÆÆ·Þz+ðRÓ2Å¬ê¤M6)¥¸rå9sqq±&.jÕ*V¬ÏGZZÙ9qVÍJ`Çc?Ù¶j°o¿ýv½ãRÓ­Î`%°Ù*¬ú|>3¬dxnÌÎ·uuugÕb1Y5V>Ç³lÙ2Óxâ.K&«wïÞ2sì& «@H=|ø099955UeR&:¤2åååÙ³ªù~¿ßåruvv>ÛªÑá74á³Ï>/ÆSýþþ"þ¹&ªÆ÷ Yµ_¹óÈ*ð|ªUn·[UÌ¾ùæûTAõù|ÍÍÍæüÝÝÝYYYº»q§Õááá]³²½yófó­YÜ¶ª7õõõÖ'YyN²`J._¾ÜÒÒbõ¸±±ÑLé¨FÞ,"¬@V «¬@V «Õ¹ê7¿ùÍâÅ=Ä«¯¾ÚÚÚ¶YÝ´iÓªU«º7ß|óòåËáÕ5kÖü!ñÖ[oUÈ*YU² «d²JVd¬È*YU²Y%«²JVd¬@V «d@VÉ*¬¾Ymkkóx<n·;##£¹¹Ù~RYÕIøýþêêjM8p`íÚµöjkk7nÜhýî»ïÎX½z5YÕ?ÕÄÈÈHbb¢ý$5µ¾¾Þ:úí·ßFó_ü§@VÿÛí:-©©©YYYéóùººº4çqú§b´ «är¹¬éÈÈÈ çéëëóz½l[ÕIÄÇÇÀïlã¬Èêÿ_UU¥	úý~ÇJà;wîÑjVVYÕIèÎ%$$¸ÇsíÚµ?Þ¹±Ó´¶¶¦§§»Ýn=¬È*_ «d²Y%«²JVd¬@VÉ*¬UY%«UÈ*YU² «d²JVd¬È*Y¬@VÉ*¬UY%«U² «d@VÉ*d²JVd¬È*Y¬U`Nêíí­©©Ùµk×É'X  «dÀ555-^¼xÝºu[·n]¾|¹×ë½wïd¬aK#ÈâââèèhïôéÓ³8Nõx<µµµÖ¢¢¢¼¼<9È*YÂzí¸~ýº¦ÀØØØ'OÎÊ5×ÔÔèÊís4TÕõ±ØAVÉ*4Nµoò<sæ³rå»wï.--uÌLJJêééaÉ¬U OY- §O^¾|¹lÚÞÞ¾xñbF« «dO,**²ÏQóbcc<xðÃ¯åóùM¤ÕÔÌÌLakkkß÷ÝôôtÖÔÔð,¬U L~=::ZÁ³ælß¾]CÌÙº~åyÝºuêtRRuå'OÔÄáÃoÝº¥é'd¬aBSYKKK5§A¤7ë·ÒÛÛ;4FMmjj²æë¶të·oßæY%«@¸téÒ¶mÛÊËËéçJ5,öz½j¹ýC8Y%«@ÒøR¹ÕáìfÕçó9fúý~²²JV°õàÁ¥.66V¯)/~÷Ýwgk;ÞJàg±Ú «dx!¨£ëÖ­3;®Zµj¶®<p¥]»v±ÌAVÉ*¾þúk¥ÎþÑRMkÎ¥Kfë&Ìl,YÍl@VÉ*Î4,((pÌÔ£G²p@VÉ*éÑ¨4ðs«cß U²`JÌ*ßòòrkÎáÃøA7U²`&ÚÛÛÕÑììì­[·æææz<«W¯²X@VÉ*zðàAMMÍ®]»=j£ïºuëÒÓÓõB³ûv¾.d¬¡ØØXUöúõëMMM>ÏñýY%«¦ÊëõVVVÚÇ²g?r³÷îEýèG?úÓ?ýÓ×_ýøËd¬áIüÔÍ7ïÝ»wV®ÿ£>RPõ«_üîw¿KIIY°`eY%«@ØfUcSGVKJJf+«.ë×¿þµNttô|ÀY%«@xº¸¥¥å_sgg§²êùáfdd°ØAVÉ*»,-_¾|¶vYø?ùÇ*ßÿüçý×ÍbY%«@Øjoo÷ûýéééÛ¶mÅØ$$$üÕ_ýuTÙÖøuÿþýsb±téõ×_ÿ³?û³_~Y/Á|iY%«³ßýîw/½ôÒ+¯¼òájª¦êpNÜó'Oj¨­õÙ³gûÛßÆ¡¬d¬xÎ¢>ø ##C+ãTyçwìs4lÕP²JV`Ú·ëÍÁo¼Á!«d¦ÍåruvvÚçüíßþ­;1È*Y©JII±h``ÀívoÛ¶%CVÉ*ùîöíÛ»wï>úô÷sîèèxé¥~úÓþú×¿þÕ¯~µ`Á×_%IVÉ*ù®¦¦&66vÃ[·nMOO_µjS¹àþðwÞy'!!Áãñ|øáS¼È*Yjµµµ%%%7oVóéµ··«©Ö/h¨«Ûå)Y4æSØ4jÜ;fÉ%Ïî^wíÚ¥~;BÂ² lÝº533ÓZ¡ª	¯×[^^þn®¸¸øðáÃ,àYÔÔ¦¦&û£Gæåå=£ÓX£aûóçÏk¬Ì² ,_¾üúõëö9gÎÉÍÍF7wïÞ=Ç£¸õÌ·nÝRSí¿Ø²JVÌa6l(--µÏÑhÒ1gvµ··½Þ%Kèe4::zëÖ­< «ÂÄíÛ·/^¼mÛ¶c¹ÞÞÞg»§^ºtI·ÈS² ¬¨p¹¹¹æ7a²³³ë²JVd¬È*Y¬UY%«²JV`Ö]½zµ¼¼|ïÞ½Ö×÷¬UíÛ·ÇÆÆx<M<»	Y%«ÂÙùóç¬OÙÞ»wÏëõjØtD[TT«CµduÚÚÚôÞÍívgdd477Oñ$²0£Z½Þ¾E~ÔÈÚç9sfÕªU³UVVêo×®]Ê°ÝÖÖÖ²ôÈê´ùýþêêjM8p`íÚµS<¬"GEEeffjL³|ùò[·n±LÂFê¨â%KØçôööêo ¥¥ÅÞèèhëð@V§*..nttT###ôí·ßþ4ÀË/¿¼páÂ$`Îúó?ÿó^zéÕW_5GõJêr¹þâ/þ"ðñññ:Uç×¿Ëmn=ÅbóãÿØñÂ¥'7ð¥L?ùÉOX/W^yåÎªÛí:xÒwß·/Ào¾ùÚk¯mæ¬ÈÈÈ>úÈ>Gÿ·¿üå/góù|jêÏþó5kÖ$$$¤¦¦þÛ¿ýKoN0k#rrrÌSöw÷w:º~ýzûyþþïÿ>--ÍqAÇóÏÿüÏ,ÀÍ%K^è¬ê¹5­×)ÄJ`Í&UEÔ1Sÿ·Û·o·Ï9yò¤þ­_kXµjÕ¶mÛXsÅÕ«W½^ollìâÅõT:~ÝZ	lÿñK.éü¬f%ð´ÅÇÇ5½âIdaC/=rss=j³aÃÃÛçèu933¥7·´ïÔ½÷jxZSSsëÖ-ý(À?Õ)ÉÏÏ¯ªªÒý~ÿO"«x1ÕÖÖlÞ¼Y/S¼Î¬¡§õj«|êµU£XGh÷yIOOgËïÿûüÇÔKÄÔùü÷óî»ïj8ÍnÀdutç^J®]»öÇ;1ÞId/¬ÂÂÂ¤¤¤Ý»wû|>µp*ù×ytNMÖ­[gvüÀbiiiQQcd£°Ìõæ[/n·;**JZ,Ìë¬òu?póçÕ«W=ÚÔÔt%¡®VÁØµké´eÑÑÑºÈ<_æJ©õöâ?þã?ô.ü'?ù	 «dsÞ*++ísZZZfqó§"ªk[<&è>/óPZZÚücû;vüèG?bÉ¬UÌy¡Ùü9ñ>/óM\ÜßüÍß8fjüÊY%«óJKKìs¶oßÎæÏgê7ÞX´hÎ¿üË¿0ZY%«½½½)))ë½÷vïÞ½xñâ¯¿þ%óì´¶¶jlª¸Ý***ÔÔwÞy%²JVnß¾;&;;»½½eò¬©âr¹Ø	È*Yðüö·¿U_ÿûß³(@VÉ*^æÕ«WÙY%«øAÚÛÛ>ß%Kô¯Åí «d3¤ÆÆÆîÝ»×|ÍùHÌîÝ»Y2xq|ýõ×555MMM|í>Y%«xÑíÚµËñ©óçÏ;~_x^¿Øòùj-½­¬¬ÔÐÓ§OOå:É*YÅ|Q\ìøeY°`K/ ?ÃðÜ÷8sæLtt´Jo~X½·¾³¬U0ZÝåø­â1ý8U÷Êþë:j?Y%«@×ô£Ö²dðÜMñ'îC¬²²RãTÇýÔàu^m÷%«ÀD<ÞnëM·&t8·áÅ¤ºuË>';;ûùVwïÞ]ZZê©üÏ«õÀdÞh?¾¦¦ïEÂ¥¤¤$33³§§ç¿Æv_Ò85ð'îCìÌ3^¯×þÖóêÕ«ºWóêy!«0W/X°Àì¼|ùòë×¯¿QY·n©»ª;vðàA²JV`nPÀZZZnß¾ýlÐýÉÍÍVP5N-//oÏYÌ25~¾®¬@VÉ*¬UY%«U² «d@VÉ*d²JVd¬È*Y¬UY%«²JVd¬ðBgõÑ£G/^¬¯¯xüø1YÕPG÷ïß²~ýú'Nèè©S§tÛÐÐPYY©é³gÏUYRVsrrÌtmmmRRÖutt´¿¿_g¸páYg¤¥¥eïÞ½ååå_ý5K#²ºeËèèhRËÊÊ.Aªf677kâ/¾Ðôýû÷É*Ìº¿ßRZZZTTuðàAËÏêªU«ÌôÝ»wMA5sÇ°ê=¦»ººÈ*Ìºââb½¦+®æèõë×5ÈijjbÉIV5íóùÌÄñãÇ5ÑØØ¨é;wîUu§*¥ö9¥cX2s;«RVVöùç	3³£££¡¡ÁÌ|ôèYY§±éÐÐÎáÃX2s;««&:;;Ï=Ë¶UxFôò[SScW^^Î«Y­««S5£¢¢jÇh:&&ÆL¨¯())1ÃÙ¬?Î?æÌMkØºûöôôô°dæjVûûûGGGÍÇiÌ.ÁÖ§k®Â×AÀ³¦LJJÇãQ_/_~ëÖ-ÉÎª1<<l¤===ÖÌ»wïjÛíîîî&«ðLÝ»wAjdõæÍÊgAAAÐSÍ¶ÕÄÄD² «HNN^¹råÄçéìì<uêYU~ÁÐfµµµ5gLí÷Ìô±cÇ"""4MVdurO<1ûÛ?Ìºoß>F«²:È*¬U^¬*¥üq`VGFFÈ*¬Î|ªéèèh³Ë&«²:ó¬²@VÉ*d¬È*YÕéeuhh¬Èêä.göøõù|ÖÞ¿ÖtUUù8² «xüø1_µ «üd¬Â;«uuuæSRRÒÒÒÌ×FDD466UY+W®(¢½½½qqqË-3û«pVduz­ÉÌÌ¬­­u»Ý:pattô¾û***Ìï+´©©©d@V'q÷î]eµ­­mâîæääUY|¨úöÛok¢½½ÝëõæççihhúäOFFFîÜ¹ÃJ`YÞ÷B(±ö²m@VPVsrrücÌw-mÜ¸¬Èê´Wúé§:¬««ihhÐÑÊÊÊI·¼UYufõË/¿4ÓIIIK.µ¤¸UYFV­v&$$ø|>ûIÇ'«²:ÑªõuéééfkGGYÕidµ®®ÎÕÑÑQÍ_¹r%»,Èê-Z´híÚµ|Õ>¬ò6QVÛÚÚ<ÛíÎÈÈhnn¶ÔßßaCVáUEnÃÖÑÍiiiÁUùýþêêjM8pÀ±2¹¶¶Öþýß÷Ý¾o¾ù&YÌá¬öõõ)¢­­­öÊ7T\Üèè¨ù¹ÖÄÄDûIjj½uôÛo¿MðòË/¯^½§0÷²ªòFGGÏÒ?~Üüz¹h¡ ;)·ÛtZRSS³²²4Óçóuuu±VY5¡9qâ5H5?f¾~ýzûÈõý÷ßúuºk:22r¼ñ±×ë%«°ÊªãK4m¾øþýûö3ìÛ·oÒ+±ÖÇÇÇk¼kÂï"ã¬Â'«ÉÉÉxüø±5³¢¢bêW_UU¥	úý~ÇJ`óÃs­feeU@fUµ3¿[nF«ffBBuýû÷OýuçtYËåñx®]»f][[[ÓÓÓÝn·ÊJVa>Z5íééÑ´¢&vîÜÉ×AÈê³*O<±fîÙ³¬ÈêÌ³ôÇmÈ*¬NcÛªýë¬OàLº'0YÕ [ýê«¯Ìàêêê?þØñy² «Á)ªfgg§9úÍ7ßØO=rä1YU~gõÐ¡CöoÛ·6»UY¶o¾ùÆþmÀÃÃÃ:zäÈÀÃd@VÇÝ¼ºlLCCÇãQÕÆ¨©ùùù¨®®ÖôÊ+É*¬Ni`ÇNLbÛ*¬ÎÄàààÛc46Ý¹s§ùUóIV3T-++#«²:ß3·vSRÕ§UYÜÅ×±¾hIÃÖïiÎ±cÇ4Óþãäd@V'§¡ê²eËøÜ*¬þÐØÜ¹sÇú18² «3g>¥zîÜ¹û`³Ëõ¥Ád@V§ÔÔªªªÑ1sáÂÍéîîf´ «Ó000 ^¹rÅ>ÓUë'ÍÉ*¬ëñãÇÊçÈÈ²cí¦ùÖîÁBVduª_ahÿ®%vYU~ «-Z±b?@mmmDDÄ±cÇÈ*¬NýëõÒ¤¤$ëË"tR]]YÕidU)­óÉ',P>qâNêêê"«²:;£Õúúz² «ÓÈjZZùó-[¶DEEúé§®««ÓI7nÜ «²:¬Þ¿_ï½÷5'++ëÉ'§NbO`Yªúúzk§ßøøx³UÓ111Ø¿¿.YÕ)×5BÍËË³¦srrøÜ*¬Î|U0YÕgµµµ5gÌx+Í´n¬ÈêT¿xxx¸°°P½÷®Þ¼yóÜ¹s!Û¤JVáUQV+++ÍnÀö/ÜWq:¤ù>¬Èêä«ªªÆ;Ã£GTÖõë×UYål «U² «d@VÉ*d¬È*YU²Y¬UY%«²JV «d@VÉ*¬UÈ*d¬È*YU²Y%«²JVd¬@V «d@VÉ*¬UÈ*YU² «d²Y%«²JVd¬@VÉ*¬UY%«UÈ*YU² «d²JVdÕnpp0111p~[[Çãq»ÝÍÍÍd@V'¡;äþøýþêêjM8p`íÚµø¿É*¬þ¢ØÝÝ4«qqq£££ÑpöÛo¿æ¿øO3¬ÚîM°¬ºÝnûôwß÷¬^½Ñ*¬NUËeMGFF²m@VGÔ8«ñññ###f%°¦É*¬Î|´_UU¥	úý~² «3Éª9ªûàr¹<Ïµk×È*¬òu²JV «U² «d@VÉ*d¬È*YU²Y¬UY%«²JV «d@VÉ*¬UÈ*d¬È*YU²Y%«²JV'244tðàÁuëÖåååmß¾``¿` «durss/_~òäÉÓ§O¿ûî»>ïÁüY%«Óvøða5ÕÞQYKJJø#²JV§mÝºu555ö9.]Ò?b «duÚü~ÿ3gìs®_¿îõzù#²JV§mÛ¶m°Úç:æÈ*Y,Y²¤¨¨èÞ½»víÕ?b «du&nß¾­áéâÅÔìììöövþ¬UY%«UfY%«²JVd¬@VÉ*¬UY%«UÈ*YU² «d²JVd¬È*Y¬@VÉ*¬UY%«U² «d@VÉ*d²JVd¬È*Y¬UY%«²JV «U² «d@VÉ*d¬È*YU²Y¬UY%«²JV «d@VÉ*¬UY%«U² «d@V£ÁÁÁÄÄÄÀùýýý6d@V'¡;´µµµ7nd´ «S¥(vwwÍªZ__oÝ --mõêÕ<Í²j»7Á²åv»>_WW²º:À«¯¾JVduò¬Zúúú¼^/+d5xD!MU$«²úVß¹sÇV³²²È*¬Î$«æhkkkzzºÛíÖcPYÉ*¬òu²JV «U² «d@VÉ*d¬È*YU²Y¬UY%«²JV «6ÅÅÅ/¿ürÚ¬JNNNMMMCiÿå_þ%Ë!~ö³½öÚk,PJÃr%ýëOv¯SÑ	ç¬öôôUÿþïÿ±yóæ#!ýæçç³BI¯5ÙÙÙ,PZ±bÅo¼Ár¥ÜÜÇ3»×yôèÑÿüÏÿÛ¬ÎºÿþïÿVVX¡ôÊ+¯:tåJÿú¯ÿÊr¥øøå/Ér¥íÛ·gddæ¶È*Y%«d¬U²JVÉ*YY%« «dd¬U²JVÉ*È*YY%«du¦_íµ+W®°(BiÅ'N`9Ò|ðßüåJzóÑG±BéÀ999d9¬@V «sB[[Çãq»ÝÍÍÍ,gçÚµkK.Õ¢öz½Zì,ü¹páBDDð¡ñøñã7FFF&''·´´°ÌC@Ë9==Ý,aó![æd5¿ß_]]ýtl+÷ÚµkY ÏNjjªÙ/ìøñãiii,üÐxòäÞÍXYe?keee_|ñÅèè¨^ëSRRXæ!w÷î]Mè0111Ë¬>ô ó| .Â;wîÙ³ÇÊ*ËüYÓð¨³³PÒÛ¾¾>MèÐ¼	Ù2'«A¸Ýî ÓxvZ[[7mÚÄÂ½y÷ù|z±²Ê2ÁKÊþýûõÆQ¯ï,óhkkÓ×¹õòÊeNVp¹tdd$äYôèßïfá@^^ÞÅÿçÿû¬²ÌCðRYY©®®.½§aÀ²eËÌõuÅ¡d5øøø³®@Ó,gª¯¯oãÆýýý,üÐøßXæ¡yIqXæ!XCð¼9Y"??¿ªªJ:Ô(òì´´´dee²ðK_Yæ¡Q\l¾>ìæÍ,óÐUK[7nÜÐÈ5Ë¬qùòåËåñx®]»Æyv#'~è³Ê2Ö>|§1Ïçëîîf@OOjªe®CMrUÈ*d²È*d²Yd²Yd²`r?ÖáÎ;5'&&&##£¶¶Öï÷ëPóËÊÊÆ	0¿©2óç[¶l1GuÔü(²'O444|þùçÜ't¸cÇFGG·´´ØÃyäÈëhzzú²eËÆèâõõõ:CNN5çË/¿ÔZ¹|ù²æX?úa²ZWWÇSU ¬hzáÂENÕá¹sç~ÿCåöÑªUÙ»wïêèýû÷uøöÛoöß»5/))qÌQuCyyyºÂcÇiÎ5k4èÐ!M8pç «ÀzêÔ©E©pÊdUUÕ¾ûÔ98íQ<~ü¸öx<¸êRjBÑ3T­®®Ö7nX7cÓ7o*áfÎ£G4Geùd¯à¯¾úÊMÍæÏ1;vìP#M)í£UëRæ7Ì;::4ÑÞÞ®CóóÚX¶lýÌ¦ÓºMWTThuåê:O@VppöìÙÑl@2Í:Z5G/^¼hÖå*ÀÙÕÕ°víZMØ¯_WþÞïõÆæ²yyyBëzx"²ÏUqµï©«éÜÜÜ YU;[[[5§¯¯Ïlµ6¬Õ¿wîÜqÍ7uØØØhsÍ8Ø­j°Ë³U ­¤ûöí[´hQee¥;ºÝîÚïÙWkôiêØÙÙ©C­aÙ)ÉLÄÅÅ©£££ºrMÛÃt´j «ÀöÙg°éÐ>zztØÖÖfæè¤¨¨(ko#Ã4UÅuRÚßßov~òäI`;­d[æó-:4-¦ÕÕÕÇq)ó	×üü|«öF¦¦¦y`´U`>0~éííÕaUU5ßår­R¥4YÕ©­¸pávttèPcV³¦WCÕq_&­dÌGHÍ8Õ~üñÇf°«ì:¾hðáÃ&J©UóµÍÍÍÓ­Þ¼ys$dÀqÿþý³gÏZGûúúZZZ¬/s0KÍÍÍµeëêêtthhH9Ô³³³ÍI:)&&&0«Ç3?éüÑêæÍÍwJðtd_ÒÒÒª««­£çÎs|¿¿«ùZDU!)@V «UÈ* «UÈ* «UÈ*dUÈ*dUÈ*dyéÿ³ÓwGIEND®B`


dãÖ¾þúkãZË744$7²ØËËËe¾übÕÕÕ2ñÅ_.ÓßßçÎ¹ôþýûrzïÞ½/^ÈÄGÔÔÔ´sk¬«®¢¢BòS__tðgt®§§G¦:d^@2¬cYã¬ä9¬jÿ»¢^ôúõëW¯^ÉÄ;´e[ÓßVJ¬sjkk#""ø[dXun·[tËª¹s2`é¸¸8ó/_EEEz¶®®îÀ!dÕëõj2^QziÌ	#KdUF«>4.cdxÏd¬¹X-é°°0ãlbb¢Ìá©#uNZZZ¿ùZ¶`³½Ôn·oÐ/Õîîî¡¡¡¦¦&#«2äåoU`eÕ´ùùyÏ'3ålkkkVVÖu|û?ð»eU.úì³Ï$¥2ñèÑ#]¸¤¤D&ÂÃÃ³³³ù[dXuÉÉÉºçréÎéF`Ëe^@÷­êv×®]k½ÑjuW±Ô4==]çU¬kA³mhhXºs:´¼_E³ªo]~>WÕ;vhøÅ2YÒ¬NOOëóáÊÈ*°Z^½z$L^½zU"äñxÌù^¯×n·?yòäÍÂ>T>~üXê%|ùåË©ãJ³j=qâLT/Xì6A³j¾)½GÈ*°ê¦¦¦:$Yr8ÒWéÖ·ß~kJPÓÓÓÛÛÛuù¡¡¡Ý»wÉUd°»ÌAgÈY)**'-/--ÕªÌjà¾UyÅÐØØh¼?4@V,KgggGGÑãÖÖV³2gd²Yd²Y¬nL¿ùÍobbb¼ÿøÇ===dõÍg=À;øùÏþ»ßý¬~Õ½÷þ	wðË_þ¬UY%«²JVd¬@VÉ*¬UY%«²JVÉ*¬UY%«²JV «d@VÉ*¬UYý ³ÚÛÛër¹Gjjjà---6¬ÈêÛy½Þ¨ªªÊÏÏ·77¦YýßÿýßûöìÙCVdõÏ¢¢¢æççeÂï÷ÇÅÅY.=úô¹sç4«ß÷-?þ¬~Ïáp£££ééé]c#ðlû·c´ «f·Ûé°°0óEçáÃßß+ö­ÈêrDGGûý~Ý,ÓqgþYÕ·(,,¬®®	9õz½Aa´ «ËÒÙÙk·Û].WwwwÐUYåã 6==]___YYéóùÉ*Yh||<==Ýív8Îææf²JV¡(**ÊUÏJS¥¬ëjÌJVÉ*|0bbbÆÆÆÌs¤²>¬UÀÊHPããã-3;vòäI²JV¡VÌs²²²nÝºEVÉ*`Å*++Ýn÷³gÏþ´pH°U·oß>99IVÉ* %%%[¶lÉÌÌÏÈÈ°^É*Y¬ÌØØXGGYCÉ*Yl@d¬È*YU² «d²JVd¬È*YU²JVd¬È*YU²Y%«²JVd¬È*Y%«²JVd¬È*Y¬UY%«²JVd¬@VÉ*¬UY%«U² «d@VÉ*¬UÈ*YU² «d²JVd¬È*YU²Y%«²JVd¬@VÉ*¬UY%«²JV «d@VÉ*¬UÈ*Yhdd¤¼¼|ß¾7nÜ «d¢®®®CÝ½WR\<==MVÉ*`ÅÜnwee¥qvll,11qÍÆ¬'«½½½.Ëáp¤¦¦¶··/êîîNKKd]Ëbd6ªññqªZÆ¦gÏ---%«+ãõzkjjd¢ªª*??ß|QRRÒ£Gd¢¶¶699¬ÀÎª±,Y½téY]±¨¨¨ùùyðûýqqq-.ý2@llì=xDÀÎívû|>ãìäädzz:WÌáp6ëééQ©dµ4@jj*Y ££#22òäÉ2Ç!K+f·Ûé°°°À^¿~íõzgffØ[__<á§¤¤dff?~¬kö£7NV£££ý~¿niË¥/_¾<xðàÄÄGÈêÛVWWËÊóE»wïâ6²º,±±±v»ÝåruwwÿùnØ¾¿#qqq6² «|¬UY%«U² «d@VÉ*d¬È*YU²ëÐÀÀÀ+WÎ?ÿàÁÖY%«:Ï·uëÖâââòòòÄÄÄ¼¼¼µüHz²JV`ãèëëèY	jNNÎ×7Y%«°¡TVVÊ Õ<g``@Æ¬¬²JV`ÅJJJ®b¹eËÖY%«°b.]òx<æ9ÍÍÍn·5CVÉ*¬ØØØX||ü±cÇÆÇÇåìoÝºÅ!«dBñÍ7ßdeeEFFºÜ¾uBVÉ*¼ÉÉI¹²È*YU² «d²JVd¬È*YU²JVd¬È*YU²Y%«²JVd¬È*Y%«²JVd¬È*YÅ¦4>>^ZZèt:322Y'È*YE(¦§§333=Ï³gÏäì7bbb:::B»©cÇ¥¤¤lÝºÕívû|>V/@VÉ*6dUhÌ¹víÌ	á¦òòò²²²úúúdúÁ2ü=yò$k «dHYYÙ+W,N§Ó¹ÒÛinnNNNs¤¯[¶lg%CuuuåääDFFÆÄÄx½ÞááaÖ	Y%«ØhÊËË+++ÍsFFFäo¥·söìY¹)ËLõÊ?!+¢­­M^®Ék8yÝ666VZZêr¹t×È*YÅÆñàÁ:Ç%%%2Xéíø|¾ââbËÌíÛ·°¡¯±,ÛEÊÊÊX3d¬b£9yò¤õøñã×®]Û·oÛíëJoD2Æ5Må9T²jÞkÍLªCss³<ÞX3d¬bÉêáÃ/]º466ÚÜºuK:eü!y.**r¹Þ0~ÿûßËß·­­Í¼û|E-|oÜ¸!VÖ-Y%«À¢úúúdì+y®¬¬äAyyy111ÀÌÌÌøøø®®®n§¤¤ÄãñVyõÂ»°È*Y°¹fggGt_»vÍårp·sÁ+WÎ=+WyùÅ>²JVl.N§óo¾1ÏÙ·oß7BøÊðT­ê»wï²nÉ*Y°¹ÅÇÇ[f;vú «d¬Edd¤åP£¼¼¼ÐF« «dÀftßjÈ¬UZàÀ¼o¬U²àtuuÝ¸q£­­y&«d¬bc)//ß·o¢nß¾Í	téÒ%ý>9µ|ò3@VÉ*ðÿõõõÅÄÄ>|¸¹¹ÙçóÅÇÇslªEYYÔT7ØÊiFFFII	«ë.«¯_¿~øðacc£Íf%«À_EVVyø%#W§Ó©_õ?ýðÍæÏvÖUÄ7`eU:zñâÅÄÄÄÔÕÕÉÙ;wîÈi^^^SS¼^é÷îU`µMNNÊPÕò=2;þ<+GÝ½7ðãvÙZuÕÜÜ®¯¯×i³ÎÏÏOLLÈ---dXmããã.ËÕòòò³gÏ²rTÐ/9òÜÅÊÁ:ÊêÑ£GN§R+**ÂÃÃe*3ÛÛÛeâÌ32ýâÅ²¬ÄÄÄ[·nÇ¯òJWZÂ1^yÈUGG1§««KæðýX_YÍÎÎÖéÑÑQ-¨Ì<uêXåá+ÓO>%«ÀÚÆbbb*++ÚÚÚä³¨¨Ïm7º<x §2ÍG#aýfU¦ÓÓÓu¢¶¶V&Z[[eúùóçdX^¯7%%%33S²ASÉõðáÃ9992õUQQQqâÄ	ÐýýýMMM:óõë×dx+ùÏ±ÒB`SgU^[F®2ñäÉ÷î±ox«gÏÉÿÛí.**¦LX>ÆÀ¦ÈjCCTsëÖ­õd:""B'¤¯2QVV¦ÃÙuøfV²uBÆ¦Û·oczòäIÃØtY×·Óè!ÁÆ»k=z´üÛéííu¹#55µ½½éBCV±nÉ?¤O-9mmm¬`seUÍÌÌètxxØ9::*s$lCCCo½¯×[SS#UUUùùùK_Úÿú¯ÿñøk;qâÄ?ÿó?[fÊÿüÏÿü+þVÿýßÿý³ý,222::úÿñûÛßòÿ÷ÕÁÁAÉgQQQÐKußj\ÜÒ7¥£^¿ßoY8ð¢æ|÷Ýw6VÓé|oYMHHØ¹sçÒË<yòäÎ;K/#Ú ÓA/mÎÿðé§îÚµë`øÅ/~ñ/ÿò/½½½2ý?ÿó?¿þõ¯333þZ¿O||üöíÛ³ò+ýèG?ÊÍÍå/úùÏ¾î¾ÁÆn·ÓaaaK_Úö­b=÷x<111YYYûöí3üÚÿ yAlSVVær¹øKk±oµ§§'wwAýtúæÍ2Fé%n!::Úï÷ë6[^ú¢ÐæU¬ÒõðAzó7366fó_ÿõ_±±±üµÈêÜÜîÈ4¿õÂË¿ÂÂÂêêjSK/mY/""Âòßñw÷wÿðÿÀÖ"«A?#bEYíììÂv»Ýåruww7ô¢ÐæU`ùnß¾-ÿ>ÒÑüä'[¶léïïgÍFVù8`½§¤¤$©é~ô£?üá¬`í²:??/)=räH`Vu'YÕG¨ú>=dI§É*¬U6P<(///--½vít²JV®¤¤$>>þìÙ³W®ÈÈÈÉÉ¡¬ «d@(nß¾½ûvã£-$¨ûöí;~ü8kd¬X±âââk×®ç´µµeff²f@Vß)«ò¬P^^^ss³yÎ7ß|ÂY]TKKÇãÑ#~ÓÓÓ£éêêjý8²l6ååå6Ï9þ¼<3°f@V5;;ûæÃDVÕ622SYY©)Ý½×åruuu±f@V7 ²¬¾¾¾¬¬,«ku²JV¼ÉÉIËWâdu)ò?ÓÐÐ S¬[h³ÙZ[[É*¬®Ì£G$¢###QQQ;vìÐã¥[Vdueo­ÉÌÌ¬¯¯w8rît:/pùòeý>s	mRRYÕ·¬ööö.ÝÝÜÜ «oªîÚµK&úúúÜnwaaaÑ¦¦¦­[·~þùç~¿ÿùóçlÕ.$Öö­Ö®®®ÈÈÈ¯×;<<üd577×»@?kéàÁd°êt:¯2===22RZZo|ÄºËªô/¾Ó­[·FDD455ÉYÏ÷Ö=¯d°Ú²³³¥©æ9åååë7«_õNKÿÓÒÒÌI*ÅËSGdddVV<C±NðÞÅÄÄX¾ë·¹¹9//oýfÕhglllzzºù¢ÚÚZ² (y¦ËÌÌôx<Ï=iO8ÎÖÞ/ËeÙäëÖ-yà£Uãã RRRtk?Y¼"¬RV¾ï]QQQqq±ùõ/]º´~³ÚÐÐ`Éêüü¼Ìß¹s',XLYYe<ßÉ5÷kll,s<Þ*++:dÙ,¼²j¶mÛ¶üü|>jÀrËsyÎÈÈHdd$kïDÔçó¾Ç/SâlÈ*°<xð &&ÆüBCðUçøU²¬/ÇáéñãÇ¯ñ^ÞM|ÀYµÙlÅÅÅÆÙþþ~ÓÑÑAV,G[[[yyyIIurrMÕ/_JDzzÌVduü~¿¼Àt:EEEú^ÚÚZýöò¸¸8ã#ÍY² «Áé[hêêêAª~ùÌ#×ýû÷UY]Ö^UãCdZ?øÅæ.@VduÅYMHHÙÙYcæåËÉ*¬.+«IIIú½å:ZÕ±±±Æ/^$«²ºâÑªtxxX¦»»»uæéÓ§É*¬U177gÌ<wîYÕÐ³ôËmÈ*¬®`ßjcccà;p8@VCyßê×_­[ccckjj9by¿YÕà$RÍ'OèÙo¿ýÖ|éõë×ÍÉ*¬ò6¬yV¯^½jþ´c·+YÕûöÛoÍ<33#g¯_¿¸q¬Èê¢»Ww,hjjr¹ª¦ÒÔÂÂB¨©©é;wUY]ÖñÀ®^½Ê¾UYÅÔÔÔ®26=ú´~Áª¾Uªd@VWðæÆaJÒ*Ë¥ÓÓÓd@VßîáÃùZakÑdÎÍ7e¦Ýn'«²ºÜqªUwìØÁûVdõ]ß`óüùsãËàÈ*¬Nß¥zÿþý¦¦&ó`=dÉøÐ`² «ËjjuuõüÓÒÒ"s­ÈêLNNJA=zdiÉªñæd@V5;;+ùôûýÕÜÜxàèÐ!ãð`AVdu¹ahþ¬%YU¾uÕmÛ¶ôÑGÞõõõ6íæÍd@VËüñúÒøøxãÃ"ä¢² «+Èª¤´~Áç.§2]WW'=út3dulllrrGÕÕ­666®F].ÃáHMMmoo7_ÔÝÝ&¹ÝnYlµ³ÚÜÜ¼ûvùe"##322úúúxY§¬&''ë=ztëÖ­_|ñL744ÈE?^¬z½Þ¨ªªÊÏÏ7_¤o¥­­­_lU³*ú|¾éçÏyöì5 «¡gõÅrúÉ'svïÞ=77wçÎUÚ¥ïê1q\ÜbËbÿ'@BBÂ=ÞTVVçñP²ÆÆFã ßèèhÝÃ*Ó2qñâEîÏªÃá:mÖÓÓ#£RÉê§~úÓ¾¬ºÝîóìÛ·ÕPÌÎÎ#TÇcLçææ®ê1Gæïpõë×ÒûUÝ-5Ïñù|2å¡dõÍ;n^í¬Ú~ Ó22ÖoS¶,ùòåËNLL¬öÀgÏMOO×³###Û·o²òP²ºb===¹Û¬ÓYYYï=±ÕÕÕ2!§2*5_ÔÑÑ±÷î©©©µyMqqqLLLiiiIIÓé<yò$3 «ïôiÀ333ÊÙÁÁÁû÷ï¯Æ.UCgggll¬Ýnw¹×§ë@6..Îf²ï[mkkaëùóçÿûßó ²úF`ÉªÏçÓÃÍ¸/Å½zõªÌOOOçSdõítclP¯_¿²8p¬nTccc2L?|øðñãÇ¬ «|B×ÕÕår¹®]»VVVæt:ïÞ½Ëj@VÉ*B(CUãl[[[dd¤_Y3È*YÅÊlß¾zzÚ<3''§¾¾¬U¬L___ff¦efAAÁíÛ·Y9È*YÅÊLNN:Nó7öYæY%«X®K.¹ýøF©iVVVII	«Y%«Ñ­[·Ün÷-[ËÊÊ,»Z¬UÈ*YU² «d@VÉ*YU² «d@VÉ*d¬È*YU² «d¬È*YU² «d²JVd¬È*YU²JVd¬È*YU²Y%«²JVd¬È*Y¬UY%«²JV «d@VÉ*¬UY%«U² «d@VÉ*d¬È*YU² «d²JVd¬È*Y¬UY%«²JVd¬@VÉ*¬UY%«ÕuÕk×®x<cÇñx²ºFz].ÃáHMMmoo¥¥Åf³@YfddÜ¾»¹¹Yâ?<<ÌCÈêZðz½5552QUUo¹tnn.--ÍÈêüú×¿^WYqª4u||ÜSZZ*÷,Õµ5??/~¿?..ÎrééÓ§Ï;§Yýî»ïlÁ|üñÇëç#ÃS§çEFFò²ºGÐi1::.Ñ5F«ìß¿]Ve`z÷î]óééi§ÓÉCÈêZ°ÛíÆtXXù"ÇóðáÃïïÕ³oõøñãûöí3Ï9þ|ff&Y ««ÅØ~+ÓÑÑÑ~¿_7ËtÐÅ×VÇÇÇ322dÌÚÕÕ500 u¹§â!du-VWWËJËðt$ðäää±cÇdêv»$®<^¬®ÎÎÎØØX»Ý.£ºîîî ý°² «|¬U² «d@VÉ*¬UÈ*YU² «d@VÉ*Y%«²JVd¬@VÉ*¬UY%«²JV «d@VÉ*¬UÈ*YU² «d@VÉ*d¬È*YU²Y%«²JVd¬È*Y¬UY%«²JV «d@VÉ*¬UY%«U² «d@VÉ*d¬È*YU² «d²JVd¬È*YU²JVd¬È*YU²Y%«²JVd¬È*Y%«²JVd¬È*Y¬UY]¾ÞÞ^Ëåp8RSSÛÛÛÍÍÎÎ<x0,,,!!¡££¬Èê[x½Þ¨ªªÊÏÏ7_TQQqæÌùùyijbb"YÕ·pÊßï3_$ã×'Oge±Ï$''ïÙ³¬~ÏápÖ³/^¡j¿duOÿøÇd@VÿÌn·Óaaa|>L<ú4==À²í2í÷ûu#°L3µdÉ*¬QXXX]]-rêõzÍÔÕÕÉÄàà`ff&YÕ·èììµÛí.«»»ÛÎÊé«W¯<SÓÓÓÈ*¬òq²JVd¬@VÉ*¬UY%«U² «d@VÉ*¬UÈ*YU² «d²ú_÷·û·ÉïObbâOúÓd¬¾¿ÿû¿gU³ªYÕX'«ZRBV¿7<<|ý½JJJÊÎÎ¾Õ'ÿÿôOÿÄzXòÃzX?ùÉO>þøcÖÃß»wï¼Á7nüñ$«ïß®]»9ÂzX¿øÅ/þýßÿõ°233ÿã?þõ°222ÊËËYkàg?ûÙ±cÇÖàU²JVAVÉ*Y%«d¬¬UU²²JVAVÉ*È*Y%« «d¬®¾O?ýôÔ©S¬5PPPPYYÉzX¿úÕ¯~óß°ÖÀþýûÏ;ÇzXçÂd	Y¬@V7ÁÁÁ;v8ÔÔÔÎÎNÓÛÛër¹tN;«èýjii±Ùþü@eU¯		«zõÌÎÎ<x0,,,!!¡££U½y3±Ûík¶ªÉêÉß£±±Q&=z%^¯·¦¦F&ªªªòóóYEïÑÜÜUVõ*©¯¯çzóVõ*©¨¨8sæÌüü¼4511U½dõÊº]³UMVCwÿþ·Û-Wù'	¿ßÇyN>îÜ9#«¬êU"MÕVõê½.òä	«zÍìÚµk-W5YqµmÛ6y®¯­­³Ã¸È<w4::.ÿFVYÕ«$))i÷îÝ²Je?úU½zde^¼x1<<ªýýý¬êÕæñxzzzÖò	¬®¥¥%::Z&t«½cÍ¼Çÿ¾YØG¢sXÕ«íåËºU½JdÅú|>/ò"U½ª>úè#óÊ_UMVßõ§J~¿nXÐÐâý<:ÿ«zmèÓ«zW&O «­¢¢ââÅæ¿«¬®XRRÒàà LtwwïÞ½[&«««eBN½^/«h5úª¬êÕT?þ«<ªWUIII]]Ý÷dff²ªWÕÎ;ûúú³k³ªÉêõöö¦¦¦ÊËÌ]»vMLLÈÎÎÎØØX»Ýîr¹¤µ¬¢ÕË*«zôôô¤¤¤È£:++KÊÊª^=¯^½òx<ºhhU½ªÂÂÂô¥µ|!«UÈ*dUÈ*d²È*d²È*d@Z[[m6Ûìì¬>ZæDDD¤¦¦Ö××½^9ù>/Ølúå*KÐ¯?zô¨³úM¢Y°AÌÍÍ5558qB"WWW'§§NS§ÓÙÑÑaçõë×³)));vì(Z Wollrss9_õÌyõêqÎÎNc|fµ¡¡?È*E©---9I¬Þ¿ÿÍß	o­³/^¼Ó]»véÌ©©)Ëøµ¬¬Ì2Gê+?ÈãñÈÞ¼ySæìÝ»W¦¯^½*ÓUUUü-@V|ðîÜ¹³mÛ6)d²ººúÂÒ9q£X[[«Ó.K¤W¹MeB£©©©:T­©©9?6®®cÓÁÁAI¸ÎyýúµÌÊ²þAVl¬ÿjíë¯¿Ö±©îþXpêÔ)i¤Ò<Z5®¥_ßßß/rªß³-;vì0/¬Ûi-ñåËeqãÒuþ «6÷îùý~)¢î@Öæ­êÙÆÅÅé¶°Ì|úôilll~~¾Lo_nüO>ÑíÆz]Çc)´ÜUgÀ*q5©+ÓyyyA³*íìéé9/_¾ÔÝ¢ÆUÝüûüùsËÊikk«1ÌÕq°1ZÁ.Uî%½páÂ¶mÛ|>GbbbýÌeô©u|òäÊbMô $RÎÏÏËË´9ÌAG«æ¡0@V|À¾üòKêèsxxXNuuëVãh#¥MâZnMR:11¡	ÏÍÍ¶Ñ*@VkrrrjjJßß"§z$îéÔ:ÖÔÔ¸Ëµô®FÍLJJòÜÁh «Àf ï~Óêêjc¾Ýnß¹s§R³*Jhu¢¥¥EG¢ýýýr*cVÝÒ+CÕE;­dØô-¤:NÕÓ#Gè`)®dOshù ÁW¯^i %¥FVõc%ÚÛÛW:ZÉYðÁxñâÅ½÷³/_¾ìèè0>ÌAßgË644ÈÙééiÉ¡,£MNNÊEY½yó¦ý$Ë[F«¥¥¥úü9@Vl.ÉÉÉ555ÆÙû÷ï[>_¿«~,¢T!)@V «UÈ* «UÈ* «UÈ*dUÈ*dUÈ*dîÿq×¨ÚpZIEND®B`
