# Supplementary material for: High-order radiomics features based on T2 FLAIR MRI predict multiple glioma immunohistochemical features: A more precise and personalized gliomas management
Source: PLoS One. 2020 Jan 22;15(1):e0227703. doi: 10.1371/journal.pone.0227703 (PMC6975558; doi:10.1371/journal.pone.0227703)
Supplement: S3 File — (ZIP) [file pone.0227703.s021.zip › statistical analysis/high and low grade/radiomics+clinical/HL.doc]

GET DATA /TYPE=XLSX
  /FILE='C:\project\hebeishengerglioma\数Õu¤ÀªR\T2¤G级©M¤T¥|级¤ÀªR\R统计结ªG\组学+临§É统计\33.xlsx'
  /SHEET=name '33'
  /CELLRANGE=full
  /READNAMES=on
  /ASSUMEDSTRWIDTH=32767.
EXECUTE.
DATASET NAME 数Õu¶°1 WINDOW=FRONT.
LOGISTIC REGRESSION VARIABLES Label
  /METHOD=ENTER HaralickCorrelation_angle135_offset7 InverseDifferenceMoment_AllDirection_offset4_SD LowGreyLevelRunEmphasis_AllDirection_offset1_SD ShortRunEmphasis_AllDirection_offset7 age
  /SAVE=PRED
  /PRINT=GOODFIT CI(95)
  /CRITERIA=PIN(0.95) POUT(0.99) ITERATE(20) CUT(0.5).


Ã¹¦N´µ°jÂk


ªþµù	
¤w«Ø¥ß¿é¥X	16-MAY-2019 18:38:24	
³Æµù		
¿é¤J	§@¥Î¤¤¸ê®Æ¶°	数Õu¶°1	
	¹LÂo¾¹	<µL>	
	²Ê²Ó	<µL>	
	¤À³ÎÀÉ®×	<µL>	
	¤u§@¸ê®ÆÀÉ®×¤¤ªº N ¦C	51	
¿òº|­È³B²z	¿òº|ªº©w¸q	±N¨Ï¥ÎªÌ©w¸qªº¿òº|­Èµø¬°¿òº|	
»yªk	LOGISTIC REGRESSION VARIABLES Label
  /METHOD=ENTER HaralickCorrelation_angle135_offset7 InverseDifferenceMoment_AllDirection_offset4_SD LowGreyLevelRunEmphasis_AllDirection_offset1_SD ShortRunEmphasis_AllDirection_offset7 age
  /SAVE=PRED
  /PRINT=GOODFIT CI(95)
  /CRITERIA=PIN(0.95) POUT(0.99) ITERATE(20) CUT(0.5).	
¸ê·½	³B²z¾¹®É¶¡	00:00:00.02	
	¸g¾ú®É¶¡	00:00:00.02	
¤w«Ø¥ß©Î­×§ïÅÜ¼Æ	PRE_1	¹w´ú¾÷²v	


[数Õu¶°1] 


Æ[¹î­È³B²zºK­n	
¥¼¥[ÅvªºÆ[¹î­Èa	N	¦Ê¤À¤ñ	
¿ï¨úªºÆ[¹î­È	¥]§t¦b¤ÀªR¤¤	51	100.0	
	¿òº|Æ[¹î­È	0	.0	
	Á`­p	51	100.0	
¥¼¿ï¨úªºÆ[¹î­È	0	.0	
Á`­p	51	100.0	

a. ¦pªG¥[Åv¦³®Ä¡A½Ð°Ñ¾\¤ÀÃþªí¥H¨ú±oÆ[¹î­ÈªºÁ`¼Æ¡C	


À³ÅÜ¼Æ½s½X	
­ì©l­È	¤º³¡­È	
0	0	
1	1	


°Ï¶ô 0¡G¶}©l°Ï¶ô


¤ÀÃþªía,b	
	Æ[¹î­È	¹w´ú­È	
		Label	¥¿½T¦Ê¤À¤ñ	
		0	1		
¨BÆJ 0	Label	0	0	19	.0	
		1	0	32	100.0	
	¾ãÅé¦Ê¤À¤ñ			62.7	

a. ±`¼Æ¥]§t¦b¼Ò«¬¤¤¡C	
b. ¤À³Î­È¬° .500	


¤èµ{¦¡¤¤ªºÅÜ¼Æ	
	B	S.E.	Wald	df	ÅãµÛ©Ê	Exp(B)	
¨BÆJ 0	±`¼Æ	.521	.290	3.240	1	.072	1.684	


¥¼¦b¤èµ{¦¡¤¤ªºÅÜ¼Æ	
	¤À¼Æ	df	ÅãµÛ©Ê	
¨BÆJ 0	ÅÜ¼Æ	HaralickCorrelation_angle135_offset7	9.164	1	.002	
		InverseDifferenceMoment_AllDirection_offset4_SD	9.020	1	.003	
		LowGreyLevelRunEmphasis_AllDirection_offset1_SD	.415	1	.519	
		ShortRunEmphasis_AllDirection_offset7	9.407	1	.002	
		age	5.865	1	.015	
	¾ãÅé²Î­p¸ê®Æ	25.159	5	.000	


°Ï¶ô 1¡G¤èªk = ¿é¤J


¼Ò«¬«Y¼Æªº Omnibus ´ú¸Õ	
	¥d¤è	df	ÅãµÛ©Ê	
¨BÆJ 1	¨BÆJ	35.137	5	.000	
	°Ï¶ô	35.137	5	.000	
	¼Ò«¬	35.137	5	.000	


¼Ò«¬ºK­n	
¨BÆJ	-2 ¹ï¼Æ·§¦ü	Cox & Snell R ¥­¤è	Nagelkerke R ¥­¤è	
1	32.213a	.498	.679	

a. ¦ô­p¦bÅ|¥N¸¹ 7 ³B²×¤î¡A¦]¬°°Ñ¼Æ¦ô­pªºÅÜ§ó¤p©ó .001¡C	


Hosmer »P Lemeshow ´ú¸Õ	
¨BÆJ	¥d¤è	df	ÅãµÛ©Ê	
1	3.477	8	.901	


¾A¥Î©ó Hosmer »P Lemeshow ´ú¸Õªº¦CÁpªí®æ	
	Label = 0	Label = 1	Á`­p	
	Æ[¹î­È	´Á±æ	Æ[¹î­È	´Á±æ		
¨BÆJ 1	1	5	4.885	0	.115	5	
	2	5	4.349	0	.651	5	
	3	3	3.713	2	1.287	5	
	4	3	2.682	2	2.318	5	
	5	1	1.877	4	3.123	5	
	6	1	1.048	4	3.952	5	
	7	1	.357	4	4.643	5	
	8	0	.074	5	4.926	5	
	9	0	.014	5	4.986	5	
	10	0	.001	6	5.999	6	


¤ÀÃþªía	
	Æ[¹î­È	¹w´ú­È	
		Label	¥¿½T¦Ê¤À¤ñ	
		0	1		
¨BÆJ 1	Label	0	15	4	78.9	
		1	2	30	93.8	
	¾ãÅé¦Ê¤À¤ñ			88.2	

a. ¤À³Î­È¬° .500	


¤èµ{¦¡¤¤ªºÅÜ¼Æ	
	B	S.E.	Wald	df	ÅãµÛ©Ê	
						
¨BÆJ 1a	HaralickCorrelation_angle135_offset7	-.589	.472	1.559	1	.212	
	InverseDifferenceMoment_AllDirection_offset4_SD	-1.611	.669	5.798	1	.016	
	LowGreyLevelRunEmphasis_AllDirection_offset1_SD	.771	1.343	.329	1	.566	
	ShortRunEmphasis_AllDirection_offset7	-2.505	1.222	4.203	1	.040	
	age	.119	.055	4.720	1	.030	
	±`¼Æ	-4.042	2.569	2.476	1	.116	

¤èµ{¦¡¤¤ªºÅÜ¼Æ	
	Exp(B)	95% EXP(B) ¤§«H¿à°Ï¶¡	
		¤U­­	¤W­­	
¨BÆJ 1a	HaralickCorrelation_angle135_offset7	.555	.220	1.399	
	InverseDifferenceMoment_AllDirection_offset4_SD	.200	.054	.741	
	LowGreyLevelRunEmphasis_AllDirection_offset1_SD	2.162	.155	30.084	
	ShortRunEmphasis_AllDirection_offset7	.082	.007	.896	
	age	1.126	1.012	1.254	
	±`¼Æ	.018			

a. ¨BÆJ 1 ¤W¿é¤JªºÅÜ¼Æ¡G[%1:, 1:	
