# Supplementary material for: High-order radiomics features based on T2 FLAIR MRI predict multiple glioma immunohistochemical features: A more precise and personalized gliomas management
Source: PLoS One. 2020 Jan 22;15(1):e0227703. doi: 10.1371/journal.pone.0227703 (PMC6975558; doi:10.1371/journal.pone.0227703)
Supplement: S3 File — (ZIP) [file pone.0227703.s021.zip › statistical analysis/high and low grade/spss age.doc]

EXAMINE VARIABLES=年龄 BY 病理分级
  /PLOT BOXPLOT NPPLOT
  /COMPARE GROUPS
  /STATISTICS DESCRIPTIVES
  /CINTERVAL 95
  /MISSING LISTWISE
  /NOTOTAL.


探索


附註	
已建立輸出	07-MAY-2019 10:37:41	
備註		
輸入	作用中資料集	数据集1	
	過濾器	<無>	
	粗細	<無>	
	分割檔案	<無>	
	工作資料檔案中的 N 列	83	
遺漏值處理	遺漏的定義	應變數的使用者定義遺漏值視為遺漏。	
	已使用觀察值	統計資料是根據所使用任何應變數或係數沒有遺漏值的觀察值。	
語法	EXAMINE VARIABLES=年龄 BY 病理分级
  /PLOT BOXPLOT NPPLOT
  /COMPARE GROUPS
  /STATISTICS DESCRIPTIVES
  /CINTERVAL 95
  /MISSING LISTWISE
  /NOTOTAL.	
資源	處理器時間	00:00:01.47	
	經歷時間	00:00:01.64	


病理分级


觀察值處理摘要	
	病理分级	觀察值	
		有效	遺漏	總計	
		N	百分比	N	百分比	N	百分比	
年龄	.0	19	100.0%	0	0.0%	19	100.0%	
	1.0	32	100.0%	0	0.0%	32	100.0%	


描述性統計資料	
	病理分级	統計資料	標準錯誤	
年龄	.0	平均數	43.000	2.5740	
		95% 平均數的信賴區間	下限	37.592		
			上限	48.408		
		5% 修整的平均值	42.944		
		中位數	43.000		
		變異數	125.889		
		標準偏差	11.2200		
		最小值	27.0		
		最大值	60.0		
		範圍	33.0		
		內四分位距	17.0		
		偏斜度	.082	.524	
		峰度	-1.413	1.014	
	1.0	平均數	52.281	2.3837	
		95% 平均數的信賴區間	下限	47.420		
			上限	57.143		
		5% 修整的平均值	53.201		
		中位數	51.500		
		變異數	181.822		
		標準偏差	13.4841		
		最小值	7.0		
		最大值	72.0		
		範圍	65.0		
		內四分位距	16.5		
		偏斜度	-1.122	.414	
		峰度	2.781	.809	


常態檢定	
	病理分级	Kolmogorov-Smirnova	Shapiro-Wilk	
		統計資料	df	顯著性	統計資料	df	顯著性	
年龄	.0	.155	19	.200*	.924	19	.135	
	1.0	.113	32	.200*	.925	32	.028	

*. 這是 true 顯著的下限。	
a. Lilliefors 顯著更正	


年龄


常態 Q-Q 圖


2ddXz$<òwqq±¶GæX²gdd$BÛdÊh±X¶lÙ¢íT+À²_ÅF¸»hýúõÒì6ÈÎÊþ½÷jÓµÜÜ]Þ÷]Ù/TökÓb¹§ÝeÓ¦MêöW®	®¤«££C>¤¾/ýw*S¿BUë5kÖÈÝ¥»òKlÈÕëä³½KXVåÙ#ßYYà3J#dõþýûê?ý'NÈ¥ÌùÔUeJöíÛ§ÍD#U>úõ×_kÛ.K5F:¤¿Íþýûõº|ùò3¿]»viWåk³ÛíªRGÊ/^¯WåVr._¡l¸Ýî©¥nvòäIýNõ-Kø§ýì³½>«Ú¬]ö?~Ô0YY)îØ±#lgXV%¥aeU.]Rk¿O~~©R­µöööÞºuKÕ+ì¾Z*ÔË¢OtëÆ*0úÛ|õÕWÚvll¬¶½yóæ°!e:(_ÉØØþäSÄÅÅÈm>ûì3µV¬½¶zóæMÙv:ÓKRR|4ìey|Ù</wQY½xñâ´ïE½Èª¶Õäg¬/é··Ëæ[ê?ttª5XíÕºº:í.[¨*Üúõëe[Z"ÛwîÜÇí~øAâzþüyíh¦Y/àêÕ«úë³ú´û>m W,¿ÈÍÔQBêLÊ°¿¿ÚaOT»fm.shýÕlU¿Ù*È*°Ä´´´¨£¦­i¥¨OíO_Á zQ0ÒÏÁ ÍUeÆçó=3«rß°!§ÞòÔ©SêÃf«Úmd¢<í|Z)3Ýi?û´û£¸ú~eÒ,ßÎ¦M´ïEf«j£¨¨¬¬KÌiGÖLÍªêo|ãÆ)l|ûí·êÐßôóÝ°Ñ§ÍVµãd÷îÝO~~uVý5ì¾òèDÿ¬ÙlD©í/ªÙªzUÙ¾» ðÁSï»nÝ:uû©íföhfxi³ª¾:×ÕÕ© Êï7ÚÓddX2=zär¹´Éöj¥¶¬ýR¨¿EQYMMMîÖÔÔh]ùáÔÃªIáo~ó³UVÓÓÓÃn N;odRµÚ¤þ"H¶O<)WÃ^[U7_ÔGóòòÂÒÿ®Fê®­ÇÊ§(,,ßÔ7«_aYÉ]UùÕD¾¤¤$õ°½½½r©^ó&« «ÀR¢µP/ò"°ÊªZ­½sç:àHfi]]]êT¹zðàÁH?KÏÊêýû÷ÕÁÆa÷Òfa6ÛV¥ÄOt¯nöôôÈ¶:v±ZÙY¦<rGG*ú3=Íå.S³ªMýÕN²²,3ÉêíÛ·ÕO-«Þ¤*¢Î9«ú×Ve#ì%í4CúéOm85«R#ýíý~¿:ÅRXÅÕ¹eC¾ø<íM2YznÈçVÕ]ôY-**Z»v­ÓéT¢N&õÑGUU`9¨««Uõ¾øâ/.^¼¨¦¥TJú¤i³ªÿ»Uíò v»]=¾þD¾O­ÊüX¾Hù?¶¬Î¥þuÚó)ªÕÀ<íë<vìØÆcbbN:¥^ü­Íü.aYúåòÉ'ddXÔ1´OËê½÷zµ«R¦°iâLÎ²¤-vX:XiÚc´´«*ª a§îïï×Î¸cÇýôQ»ª2<íMkbbBæ£jyîwQû6<óTûyyy<'AV%ìÛo¿MHHxÑ uM;ó4¾8Ôµú»áÈß¦¶dUÈ*d²È*d²È*d²Yd²YdÆ£GÃ±cÇæñÑÔ§ËFûöý666®]»V»zîÜ¹uëÖñ4È*0×øíÚµ+//ïÒ¥K			ë×¯¿4éøñãò!uÍ. äCréóù¼ÔÎ'N=ò7ß|#ûÁ Êêµk×¦ÿù4FFF.é444ÈþèÞäüÁæI²ñðáÃ§þ×`0:tH577§@V(ÚÓÓ311!94F¾±ÜLçüùójãæÍ0Ù¸û¶>:ìö²óàÁêjWW~^¨¹sçÜìòåËÚ]ÔW"µSEBqq±Ü·®®îÔ©S²ñÙgM½Ì¡¿ýö[ùèÅå²¥¥E%o½õ|ïNâU`?k³rÙÑÑ±cÒÙòäÉ°ÛkY,É¶ÚÐÒ¨¿å|¶gêû²äRæ©êªt1ºoJÒ(w¿ÿþ½÷dC&ÙSo#bËj_T_ÿÏ=+³vYfgppp||Ú´WUÊ§eXËªLR6[ÄÊ~øAß&IðÔù¤jeXM+Õ£É<òêÕ«²q÷îÝè¾)é¥6Ó-²*³Õ+W®hõ´1@VH¤pêUÌË%±Q¢G½ÚØØ¨nùÝwß©=B¶åòÜ¹súÙªÌðÔ-%Ò$þ~4)oºïõë×Õ­4L-ùÊ¤PÒfU8Éª þùçò¡#G¨SEø¦bbbôYfV¥î7oÞÔVÂ%«2ååéU`úûûÕK¤r©&ccc7nÜÐ¾üøquã©¯­h³U±Ìze¿L1åL¾p«&Ú´oÚÃdF«½ªMÒ+¬nÝ1É3Ìª|hïÞ½j­[¾ý'?<%ñññ<C²Ìz¶ª^VTKÁê°6¨©§~ªUJeUQ!éSö/¾øBÛ4µ³¦¦FeXªcÕW"áb¶Å"°zAWj*_ÚCV²Dõãa0|òÉ'*«jþ*Å'O>|XÍeÃn,Y)±×ë2é¿þúë°G«zÙ2¬ ÊãÇU eB,US/ÐÊ§BêP#í³%ócõ®á!K*«ò5¨9·ìÉÌÌä¹U ¬ÞºuKeUæ©j§ÌU2¯_¿®ö;vLöìÛ·ïÉÏkÂg«ò¡ýû÷ë¯jKÊ<¸Z+V¯rRMâ´Ú,VæÇ²qjÒÓþÀfÚ¬ê*;;ç@VÙ"ÊÔS6ÆÆÆôglðûýêh^uj$ºÂÂBÙ¾qãg~¶ªtÒ¯ñÊÕÝ»w«) <Ô¹sç´SChå;ºùäªZõ8rép8$ÛQdUî%é¸¸¸øøxù§i_[oS¾#í/Yyzd»wïjó¨CÔTQÀÚÃèsùäG7oÞLHHÐ¯_¹reêù"D(z2ù2ªL¥¸^I©6INNÖ®ÎûïÚÉÕ±Íj[ÍÚ×¯_ÏÓ «UÈ*dUÈ*dõ¹ù¯ÿú/ÍfàyûÅ/~¡þ´	guïÞ½7xÞÞ~ûíÿùÿYòYýàþÀóö«_ý¬@VÉ*¬UY%«UÈ*YU² «d²JVd¬È*Y¬@VÉ*¬UY³zíÚµM6ÅÆÆº² «ÑKNN¾zõªl=vÃ?ýôÓÅ)¶mÛFVduvâããüñGÃtÞÿþ-du¦ºººöîÝ+§ø·û7f«²:S÷ïß÷x<<àµUY¼¼¼±±1Õ9iooßºuëÝ»wùY«ÄÄDý¡Id@V9¬UÈ*d¬È*Y¬@VÉ*¬UY%«UÈ*YU²Y¬UXYÌªª*£ÑØÜÜÌPU@ôÁ Çã1>/1 d¥ÎÎNÍf6Ï9ÃhU@ôÊËË%¨ééé¼ªJVÑ¡¸¸x||!«(µ¶¶Ú&5551d¬XéËËË«««fß£Ñèv»eÂÊHU²`EÏÊÊr8ÅÅÅùùù¥¶¶v÷Õ~¥¬,üU²kÊDSû3ÎÎN«Õ*Ï¼£¶ðË_¦U²æt:ÃÚikiié3cÌÂ/Y%«Îb±­ßVWWççç?íö,üU²OÚÐÐ ßS^^>íÕÂoRR¿d¬À4êëëív»ôR¶C¡PYYÃáSo©~½^ïèè(ãFVÉ*L¯¶¶Væ W«Õ144vµðk2ª««.²JVàÙd:íiñÕÂoJJJww7£DVÉ*Di||_²JV`øý~§ÓÉÂ/Y%«0WR³ÙÌÂ/Y%«0'¡PÈëõ~É*Y9aá¬U,üU²ó_²JV`~°ðKVÉ*ÌµðÆÂ/Y%«=má7¢!«dæD-üòäd¬À_Þ¬U~É*YùÑ××çr¹Xø%«dæªººÚb±°ðKVÉ*ÌÉèèh~~¾Á`ðù|Ó¾*È*Yÿ£SRRdZ__ÏhU²Ñ+++3L,üU²s2::m4KJJ8Ç/Y%«=¿ß¯~²JV zUUUêTÁ`Ñ «d¢4<<m08â¬U^wwwJJJS=øýþ§Ó)ÙN±DVÉ*LïèÑ£f³933óiGü¶··[­Öòòò¾¾¾¶¶¶ÔÔTÏÇ¸U²%ª#~#L@].WMMvuttÔn·Ëß Y%«ðg­­­6M)Ñ7ÊÍÂ¢[XXXYYÉU²R[[k2dª:44ùjnÕ¢¢"²JVÉ*ü)^¯WêaGM»y²JV,ê¿6mVQ;d)--CÈ*Y°Òªß(Îñë÷û=ÓéLOO¾ò6d¬X¹FGG333F#E$«dæ¤­­Í1?!«dæ¤¤¤D&©YYYÃÃÃY%«¥`0¸cÇi*	CVÉ*ÌIwwwRRÓédá¬UÍfªòænd¬@ô:â·ªªÑ «d¢×ÞÞn³ÙNggg'£AVÉ*Di||¼¸¸X&©ÙÙÙ£££Y%«¥ááá¬¬,i*ï.²sÒÐÐ`µZZ[[U^ii©LR½^/¿ «½¾¾¾ÌÌLÉ4ï§z¬$U²`immµZ­v»~ø-//·ÙlIIIòà999¡P¡&«dÀr&©+**2ó~ªË%à?Nåñx¤¬8Y%«­@ àv»M&SYYÙü®ÓZ,¡¡!¿e6ì÷ûv²JV,CõõõêTÝÝÝóþàéééa;½^ï3gy²:ÏîÞ½HV</21-((0gÞÜMæ©#lÑÜÜÌøÕùÔÑÑ!¿Ê³¬x.ÒÒÒFãÑ£Gô¹²²2íjSSL9S?YgÒË7ojYýñÇßbõêÕÛ¶mãßÀ¼khh0Í)))íííý¹dÂ*³·Û]RR"Ób«ÕÊ;ÊÕ¢eõ§~:<ÅÛo¿MVÌ/í¿999vªù¤õõõ2g­©©Ñ¾²ºPYeÀ"ðûýiii2O­®®æÌ «½¦¦&µð»Gü¬U+LLFcAAçøYèõõõ¥¦¦Ê<µ¦¦ÑÀ2Ï*§° $¥Åår±ð²JVDO[øÍÏÏ_ S=¬U+vÄo=£²JVD¯¦¦F*YUoU² ¡PH;âwÞÿ,UfÀååå>¯¢¢SU²`ëîîv:v»½¡¡aÞÍ«4µººÚëõÊgád¬X¶jkk%n·```Þgª¾ÖUUURVÎÓDVÉ*åFÚo2JKK¨sÍÍÍa;9[Y%«ùoN-ü.èÛ655åääíLOOïììä¬UËDUUÙlÎÌÌú,þ0%¿ß/ó U²`9y½^£ÑXRR²8/pÉ´X¦ÔÚÚêp8***ø «dÀ×ÙÙb³ÙÚÚÚóóJÂåóÊ$UúZ]]Í?Y%«<é-##c¡~²`9ÙÙÙ¡¬¬¿lY%«¢'ÿÙívÍÖÚÚÊh¬UÑé©ÉdZ#~²`9Þ±c4_U²`N:;;&qÖU²`NFcvv6§Y%«¢222¤©Geád¬^[[c¿ «dÀÊ$Õãñð&á «d@ô@ff¦Édª¬¬dád¬^½Ífs8íííÈ*Y%«#~õú~C²JVÌÈÀÀËå2LaïSUUõÊ+¯ÄÄÄ¼ôÒKqqquuuÈ*YIss³ÝnOIIéîîÖïolljU~~~(«¿ûÝï¤¯ê]N²JVLCã×ëõN=ÕÃo¼öSÿæo¦¥¥1h «d@8é¨:â÷io.jiiÑï©¨¨xõÕW:U²à¯´¶¶N»ð«päÈýßÿþ÷2eô@VÉ*¿())1Ó.üêíÜ¹sÍ5ÚQÁ²!ó×üü|d¬øuß¿adF÷á$¹Wrr²:|	 «dXéZ[[m6[äß©Þ¤U²¬t3È*YÉl~²JVL¯¶¶6_¬U%y½^ÁÀÂ/È*Y0'~¿ßét²ð²JVÌ¤Ôl6³ð²JVÌ	¿ «dÀü`ád¬,ü¬Uó_U²`~¨_ÍÖÔÔÄh¬UÑS¿n·;0 «d@´ßññqd¬¶ðÛÜÜÌh¬UÑ+++cáç¬fNòx<¡¿¿_Û£v:tHö_¿~¬Ëz#Xø­ªªúðÃûÛßÖ××3ÎXYxüøñÂ`_]GFFÔ¹ºÿ~f«À2£Þ|Þ~C¡Ðë¯¿ûÎ;ïlÜ¸qÕªU¹¹¹6V>zzbbb. öÄÇÇË¶ì÷ù|²ñÚk¯mÞ¼¬ËzòXøÇ´X,ÃÃÃêjKKµ®®1ÇËª*«Ð_=qâvuýúõò»'Y´[øUâââ$¥ú=2mï½÷y¬¬¬^ºt©¡¡áØ±còÃ&¿WÊ¥ìËíÛ·Ë<Õëõªik^^Y®ZøÕ¹i(ÒïÉÍÍýÇüG++«>ËH;oÝº%W®ËóçÏk·IJJÚ´iY¨òòòZøÕ[½zõ§~ªßóÆoìÜ¹ñÇÊÊª¢²ºÿ~ùeS-¯_¿^f±ÚáÁÌV¥htt4''gqNõPTTSQQñÇÉÃ$¨±±±ü+`åfõÒ¥KÚk«'Od¶,iò;±Ãá°X,mmmóe¶*)¸®ZµJ&¯öy%UI©~¶êõzÉ*°ªß¾¾¾EþÔ2Cýþûïù'YýKV[ZZB¡zÙµ¿¿?Ü»w¬KB0ÌÌÌ¦q_`±³zÿþýG©¦þÉ:0XÊJVüïb³ÙÚÛÛà9dõ~8qâsÝºuú¦:NíêÔqÂdxL&ªÊÑOVm7IA/^¼¨ÿ¨LgegKKY^LÃÃÃÚÂ/£<ÿ¬ò6ÀÒÕÜÜlÄ·Y0'êT,ü/VVÃ_|¡6ÔYõ;::Wnnîù»UàE£Îñk2¤¬ñ¼pY=tèÚHMMóçÏËöíÛ·e»¡¡Ál6UàÅÑÔÔ¤ÎñÛÚÚÊh/PV»ººÔ	¤¦jÃãñh§Ý¿sçÊêÚµkÉ*ð"©zs·¬¬,~.«Ú;'$$¨¬~ôÑG²G½q6[%«À ¸Ýnijee%¿ÀUmøðáÃ²a6ßzë--«2sØØØ]»vUàùR¿v»S=K «ÓÎVY^21-,,Ijvvö¾¹@Vj¶ª;66FVçkhh(==]Ê¿ÀÎêéÓ§eg~~>ÀÀùðÃ­V«Édzýõ×ëëë§½ì·ÙlC~ì1`	dõÊ+j½Wj*>O¶-Ë¾ûÎ=«nÃl_¡PèÕW_]³fÍ#GÚÚÚ~ýë_¿ôÒKøÃô·©ü^+TÇ3::Ê K#«*®---=ºzõª6ýÍo~CV ½ÖöìÜ¹S*«]íëëKKKã_`éeU¨wé©l···Ëö¹sçäÒï÷U`!H2?üðCýIìªU«Ôö3gÌf³Ãáà_`éeuddD*áÔöôôôÈ.Èv0TKÄÚÌ¬s÷Î;ïÈôT¿gppP²Ô¿999ú¹,¥Õ®®®©3W©©¶çÖ­[jþJVùRQQ!íìííÕvÝºuN§Ód2UVV2DÀR­ò6ÀsñË_þòå_ÎÏÏÄnÜ¸1&&fõêÕ)))~¿ÁÈêý²Ýnÿ)._¾LV§ùøãÇÚI!;;;1,ÀÒÎjWW×öII?SÛê¯Weæ(7®««ãÇïÞ½û§~:<ÅÛo¿MV!sSµð[UUÅ¿ÀrÈê£GÔÙö§":ò·zÀÄÄÄü1iÕ«WoÛ¶K¬pÕÕÕf³Ùåruww3À2Éj3.E'66vÚmM(òz½ò³æóùXøÈj$111Úv\YÂ¨_§ÊlÑaV?~¬",«êL³òÚk¯©É¥lU@O-ü¦¦¦rÄ/°g«úªs«CÔö¬jÏ=§N¹|Ú±Nd+¿ÀÊÍê;::Ö­[c·Û¯]»FV?þ¼ð+¿¤ÖÔÔ0Yåt@ôÔÂozzz__£U²D#æää¼òÊ+ò3µnÝºË/3&Y%«@4FGG×¯_ÿ·û·ó7SWWWUUe±XxG¬>'«ÀlíÚµËh4fdd©=RÖôôtFXÎY½téRvv¶:â755U;úWÛ>uê}ÚYÈ*0vÄï¿ÿû¿ëÏG(Û2ae|åÕò6À<RGüZ­Ö_ÿú×gÎÑ(Ê~XÎYåáy¤øÍÊÊÊ¶ôuttTûh~~¾ÇãaÕP(ÔØØ¨Îäp86lØ Nd0¾ûî;²D¦~M&Syy¹¶ðëóù***ªªª222ÒÒÒ$·°Rf«W¯^ÊýÚµk7oÞ¬]ø1["S¿v»½µµ5ìC²§¸¸¸  @ÊÊUÉgzzzCCCll¬ÇÇ[,Ã;vL½÷ª699¬Si¿@VÜ¾[²ÚÓÓ¹»Û·o'«^ ÈÌÌ4¥¥¥¼9@VÿÌ-[¶Èßïw¹{öìñNºpáü¾ÿþ[·n±ç.ÊSÿyy²µµÕf³9ÎÎNþi²:ýß°JbõåµU¼ d.èñx,Ûíeee?Ç¯§¤¤D&©ê_þu²)«Û·o÷LRçÈËË#«xî|>_FFJi(ÊÏÏ«Ïå+	ò©¥©,üd5ÒRðg&f³9!!áÂrµ¦¦æ¯¼U,(©©ÕjÕOO¥gC~ù+Q¿)))ÝÝÝü»dõYýúë¯ÕvRRÒ¦Mô¸U</n·;l§×ë­­­]Ì/C-üîØ±ãù®?X2YÕÚ¹nÝºÔÔTýÎ=KVñ¼A»ÝöGiiimmmó¨_ÉÄÂ/@V£­j§p:êÖÞÞ^²çHf«>OKZEEÃáXY£ÄÛ1÷tÈêì²ÚØØÕÇËþwßCð|IAÓÓÓ¥m^¯×5iÎ½ /..6999ñÕè­Y³f÷îÝj/yÞ9sFf°$äÒÔªª*F «¼½¦¦&øÈ*YæJæÁ2Iõz½ú÷t@VÉ*¤çø*æÐÐÛí6ÍGå_¬U,íibQQÕjMJJK2.rØjkkåó¦¤¤øý~þ9²JV±´y½Þ/j³ÆâââE+ºÏç3ÙÙÙê «dK^__Ýn×¿,Ë"¼º)(--M>Güd¬b¨¯¯ÏÉÉ	Û)Ö>ëoss³Ífs:¼9@VÉ*¶¶¶©gýu82]¸OZVVf2$ç,üd¬bY	B2klhhÐöÔÖÖ¦¤¤,ÐQKCCCf³¹¦¦Á@V±<'¬V«ÕçóUWWçççËvggçB|"õæn.S= «XÎdY^^.M-++[;µsüææærªdt:--Íl6ËlÑ@Vè©sü¦¦¦.è1PÈ*YÅ2Õ»±ð¬Ñr¹ñ¬s¥Îñ®NdvßP(Ä «@ü~jjªÍf;sæ£¬Ñ«¯¯·X,ñ¬s¼^¯Ñh7j@VÉ*§ÞÞÞù@¿ßït:­V«þ¬Â@V±ÿý÷ã¥^9åªU«Þï½yyØêêj³Ùìv»¬bEB«W¯~ã7åjKKË+¯¼²qãÆ9>¦×ë5%%%,ü «duùôÓOM&þÏ]þ÷ÿWæ¬Q/«_ÍÖÜÜÌð «dueÙ¹sç;ï¼¶Ób±TUUEñh,ü «duEûøãßxãý¹Fy*ÏêqXø@VÉ*þtôïK/½ôé§jd®¹zõêY_d¬âÏ9²jÕ*³JP-ËË/¿ÜÒÒ2ó»³ð¬UÏYÿûßË?ýÇüý÷ßÏð^,ü «dó_d¬b~°ð¬UÌááa~U²yÐÚÚjÄÂ/²JV1'2=5_d¬"zÒÑÉT]]Íh «dÑS¿)))ÝÝÝ²JV=µðëõzGGGd¬"J,ü «dó_d¬b~°ð¬UÌ~U²º´¶¶JöæýG,ü «du)((HJJª¨¨¨ªªJKKËÌÌÇ²²ð¬Õ¤¾¾^æÁ`P] feeÎýYø@VÉê½¶¶¶ôôô9>lss3¿È*Y]qvìØvvû§Ó9Ç,++IªÇãaáY%«+Kqq±ÏçÓï©¬¬"F÷h@ ++Ëh4ÊðænÈ*Y]qÁ Íf+//Wljj²ÛíQ<Tkk«Ü×áp´··3°È*Y]¡ü~¿Ûí¸Ú'IYgûdõÊ$5''gxx!@VÉêJ¢+¢vÄoee%Ã¬UDO-;ò2È*YEÆÇÇ>Á`àTÈ*YÅÔÔTÉTSSÃh «dÑkjj²Ùl,ü «dsÅ9~U²y000i6Yø@VÜÝ»wÉêrU__o±XNg__£¬.¬ù×`øË×óÿMOV¯ÑI³½×øøxaa¡ZøB#²ºà¤7oÞÔ²úã?¦óþûïóoù··§¦¦ÚívÍ&¿Íüäéééf³¹ªªa@VÕ~úéÿM±mÛ6f«Ïßï·Z­555ê¬¿µµµe&gýmjj[¦¥¥±ð¬>Ï¬òÚê%''§¼¼çïQ|>ÑhK~ÕE¨BV_p.+lºùUev+5Í2¯eôUf«ø+­­­ú=mmméééÓÞXRjµZYø@VÉ*¦WQQª,RÍÒÒÒ°iGüÊ%ï@¬r:<UAAÍf+äóùÂÂ944¤ømhh`¸U²gèìì¬ÔÖÖö¡µð;00À@ «dQ9knn®Á``áY%«¿ßïr¹ìvSS£¬UDïèÑ£f³9==_d¬"zãããêT%%%,ü «dÑóûý©©©V«5ì/Y¬UÌNee¥ÉdÊÈÈ²JV¥P(TPP`0***Xø²èuww§¤¤Øíö©±d¬b*++Íf³ÛíUD)åääpÄ/UÌzs7ÍÖÞÞÎhYEôÊËËÍfsVVÖðð0£dQ÷z½F£±²²_ «<ERRR¿@V1'êT;vì`áÈ*¢'õx<ÒÔòòr~¬"zÝÝÝIII6MUD¯¬¬Ìl6ËT_ «t4##Ãh4²ðds¢øµÛíñdsRVV&ÔììlÞÜÈ*YÞðð°ÔÔd2UTT0@VÉjôÚÛÛ­VkJJJww7£d¬F¯¼¼©2Uå_ «d5z@ ==Ýh4VUU1@VÉê¬õööB!Ùhnn¶ÙlN§³³³a²JVg'777fÒªU«âããeÃãñpÄ/U²MSFãþð@ *ecX¬ÕhÈÜTÚÔÔ¤-ü¾òÊ+¥¥¥U²:;ÕÂÂB°jçøu»ÝU²:k×¯_72=­¬¬Ôv&''ÿîw¿cp¬ÕYhkk³Z­2Oý§ú'mgKKËªU«äñ²JVgª´´Ôd2y<Ë/¿üòË¯¾úªÌPßï=ijnn.ãd¬ÎÈÐÐÛí6Í555jÏðððoûÛ7¾óÎ;Ga¬Õ©­­µZ­N§süY%«Ñ/((0ùùùãÈ*YÞÀÀ@zzºÙl>sæOk «d5z555V«Õår±ðd¬Fott4''Ç`0ø|>uY%«Ñ9Ã¡?â@VÉj4Î9#Au»Ý,üY%«ÑBê¿,üY%«s"sS§ÓiµZkkkyúY%«Ñ«ªª2Í©©©CCC<w¬Õ(B¡üü|~¬Õ¹êììt:6­¹¹§,U²½ÊÊJÉx¾Y%«QÍÊÊ2²ÍÈ*YRJJÝnokkãid¬ÎIFFÛíà9d¬ÎÕðððøø8OP «d@VÉ*d¬È*YU²Y¬UY%«²JV «d@VÉ*¬UÈ*d¬È*YU²Y¬UY%«UÈ*YU² «deÕk×®mÚ´)66ÖårõôôUY^rròÕ«WeãìÙ³6l «²:?âãã?~¼wÉí¶mÛø·ÕêêêJV·Mñ_ü¬ÈêLÝ¿ßãñ<xðE`YÃÏÔÕ¼¼¼±±1Õ9iooßºuëÝ»wùY«ÄÄDYUN «d²Y%«²JV «U² «d@VÉ*d²JVd¬@V «:«W¯Þ0¯þþïÿÞáplÀÌ8&13.y13ôw÷w×¬ëþáZ¿~ý¼ôhÉg5|3ßÜn·Óéü3óæoþó?ÿ3ã0Cò·eËÆa·oßÎ8ÌPBBÇãaf(...77w~³¶¶öÿþïÿvVÂüÇ¼ÿþûO03òcÃ8ÌÐ¿üË¿03´iÓ¦Ï?ÿq¡×_ý¿ÿû¿e]]Ýâ|.²JVÉ*Y%«d¬U²JVÉ*Y%« «d¬UU²JVÉ*È*Y%«d¬Õgãý×å97CûöíûÏÿüOÆa>úè£03´sçÎ#G03ô«_ýêÔ©SÃýò¿ljj"«,1d²Yî®]»¶iÓ¦ØØXËÕÓÓ#äÒn·Ë7^¾|ç^ÿæÍÕàttt0qéÒ%áÏ?YWccc+²æååÅÅÅ­_¿¾½½ázFÛtbbbs¸V¯^½*gÏÝ°aÃÉ£[ÕbÇß½7OG=yþ?^6dÐÖ®]Ëp=Ó£Gä÷6-«WÒ	ý+|õÕW?¦:kdd|s¸Vô"p||¼-ä*<§uñâEß3ôå_:tHË*Ã4UýÒ¦a¸"ÿ;88ÈpÍJ0Ü²eË"×ÊÍjWW×Þ½e#66VÛ©ß6ýZ³ftBæ÷Wd·oßNMM]-«WÉÉÉ[·naA»qãÃÈ#Gd2 SÕÞÞ^k&²³³å¿úEþaY½ÿ¾Çãyðàl«ew%..'â´.]ºôÚk¯1ü¾råÊÉ×uÔk&FFFÔZÃNMMlÈ¯ òÃõL7oÞ|ë­·ô¸8ÃeX?ÃyyycccêªÔbbbB­¨rài¿,3øqúk×Ì©ÿæ®ôÂãL8p@E®Õööö­[·Þ½WÛ³gÏu²¹),ÏE½äääþþþ'GPË¸1ï+Ï®<»nÝº¥~ÓåÙõLçÎ2y|~zz:ÃõLï¾û®ßï_üÿêWÃæëÖ­±Ûíz===7n_·lÙ¢æ÷×¬²ÊpEÐÕÕåt:åÙåv»¥¬Wd÷îÝËÎÎV/Eß¼yáÉ:Fi9d²Yd²Y¬²Y¬²Y0ï¾ûÎ`0<|øP.¿üòKÙ°qãÆÇ#²ÿÀOý7ÔûD ÞýO>QW<x WÕ «À2ñèÑ£.|ñÅ¹sçÎÉåÁåÒb±´··ëÃùÍ7ßhWNçæÍ½äîçÏlß¾]Ûóõ×_Ë÷îiwéèè=Ú»|¨¬666òOU`YIê¥K$rX¹¼xñâß>]?[Õ*ûöm¹zçÎ¹Ü²eÚ©¿au÷¢¢¢°=R_ùDÙÙÙò§O=|ðl<yR¶?Î¿@V%/99ùÛo¿]³fN2yêÔ©ÃKçdÆ©âÙ³gÕ¶Ýn)©«ÜKæ¦²!Ñ7ª©j]]ì¹~ýºvw57íïï«=÷ïß=RYÆ «Àòú5N8¡æ¦êåÏIFªRêg«Ú½Ô;«÷ööÊßïKõVØ²±yófýU§åqd[øØ±c²Gpé:ÿY	)¢z5«æM;[UW¯¨Ör%À²óÆëÖ­Û½·lè_|×®]jÝXÝ7;;;¬Ðò8üCdX>V«þH]ÙÞ±cÇ´YvvuuÉõ²¨öÂªZþ½uëVØ÷÷÷Ëåwß§MsÕ<X­Êd¬ËzôðáÃkÖ¬©©©QsÇØØXÃÑð3ý"°Ì>UåRnva:(Im¬]»VJùøñcypÙÖyÚÙª~*¬KØç®Â&jöä²§§GíÍfíh#E5UöhÒ±±1uð£G¦¶Ù*@Ve+Ý½Wý#Ô+ªuuuv»=ì^ê/ìÙ£ÕQßÈäääiþ_`¶U`%PýåòÔ©SÚþwßWJ©²*ÐªK.©hoo¯U­ôÊTõ©ÿ/0[È*°¨?!UóTu¹oß>µ,Åì©hðÞ½*R-«ê´/_ílµ¿¿?B²`É¸sçNKKvudd¤½½];ú³Ô;vèç²ru||(7^¿~ff¦úP(%$$LÍêéÓ§ÕÑOrû°Ùjaa¡:§ÿYV6ÔÕÕiW/^¼v"~þMXÕi¥ÊLI²Y¬²Y¬@VY¬@VY¬@V «¬@V «àÉÿ3PGÓ´L°IEND®B`
