# Supplementary material for: High-order radiomics features based on T2 FLAIR MRI predict multiple glioma immunohistochemical features: A more precise and personalized gliomas management
Source: PLoS One. 2020 Jan 22;15(1):e0227703. doi: 10.1371/journal.pone.0227703 (PMC6975558; doi:10.1371/journal.pone.0227703)
Supplement: S3 File — (ZIP) [file pone.0227703.s021.zip › statistical analysis/high and low grade/spss sex.doc]

CROSSTABS
  /TABLES=病理分级 BY 性别
  /FORMAT=AVALUE TABLES
  /STATISTICS=CHISQ CORR
  /CELLS=COUNT COLUMN
  /COUNT ROUND CELL
  /METHOD=EXACT TIMER(5).


交叉表


附註	
已建立輸出	07-MAY-2019 10:45:48	
備註		
輸入	作用中資料集	数据集1	
	過濾器	<無>	
	粗細	<無>	
	分割檔案	<無>	
	工作資料檔案中的 N 列	83	
遺漏值處理	遺漏的定義	使用者定義的遺漏值會被視為遺漏。	
	已使用觀察值	每一個表格的統計資料都以每一個表格中，所有變數指定範圍中具有有效資料的所有觀察值為基礎。	
語法	CROSSTABS
  /TABLES=病理分级 BY 性别
  /FORMAT=AVALUE TABLES
  /STATISTICS=CHISQ CORR
  /CELLS=COUNT COLUMN
  /COUNT ROUND CELL
  /METHOD=EXACT TIMER(5).	
資源	處理器時間	00:00:00.03	
	經歷時間	00:00:00.16	
	要求的維度	2	
	可用的資料格	131029	
	精確統計資料的時間	0:00:00.02	


觀察值處理摘要	
	觀察值	
	有效	遺漏	總計	
	N	百分比	N	百分比	N	百分比	
病理分级 * 性别	51	61.4%	32	38.6%	83	100.0%	


病理分级*性别 交叉列表	
	性别	總計	
	.0	1.0		
病理分级	.0	計數	9	10	19	
		性别 內的 %	42.9%	33.3%	37.3%	
	1.0	計數	12	20	32	
		性别 內的 %	57.1%	66.7%	62.7%	
總計	計數	21	30	51	
	性别 內的 %	100.0%	100.0%	100.0%	


卡方測試	
	數值	df	漸近顯著性 （2 端）	精確顯著性（2 端）	精確顯著性（1 端）	
皮爾森 (Pearson) 卡方	.479a	1	.489	.563	.344	
持續更正b	.158	1	.691			
概似比	.477	1	.490	.563	.344	
費雪 (Fisher) 確切檢定				.563	.344	
線性對線性關聯	.470c	1	.493	.563	.344	
有效觀察值個數	51					

卡方測試	
	點機率	
皮爾森 (Pearson) 卡方		
持續更正b		
概似比		
費雪 (Fisher) 確切檢定		
線性對線性關聯	.182	
有效觀察值個數		

a. 0 資料格 (0.0%) 預期計數小於 5。預期的計數下限為 7.82。	
b. 只針對 2x2 表格進行計算	
c. 標準化統計資料為 .686。	


對稱的測量	
	數值	漸近標準錯誤a	大約 Tb	大約 顯著性	精確顯著性	
間隔對間隔	皮爾森 R	.097	.140	.682	.499c	.563	
序數對序數	Spearman 相關性	.097	.140	.682	.499c	.563	
有效觀察值個數	51					

a. 未使用虛無假設。	
b. 正在使用具有虛無假設的漸近標準誤。	
c. 基於一般近似值。	
