# Supplementary material for: High-order radiomics features based on T2 FLAIR MRI predict multiple glioma immunohistochemical features: A more precise and personalized gliomas management
Source: PLoS One. 2020 Jan 22;15(1):e0227703. doi: 10.1371/journal.pone.0227703 (PMC6975558; doi:10.1371/journal.pone.0227703)
Supplement: S3 File — (ZIP) [file pone.0227703.s021.zip › statistical analysis/ki67/HL.doc]

GET DATA /TYPE=XLSX
  /FILE='C:\project\hebeishengerglioma\dataanalysis\T2 ki67\nosmote\3.xlsx'
  /SHEET=name '3'
  /CELLRANGE=full
  /READNAMES=on
  /ASSUMEDSTRWIDTH=32767.
EXECUTE.
DATASET NAME 数据集1 WINDOW=FRONT.
LOGISTIC REGRESSION VARIABLES label
  /METHOD=ENTER kurtosis ClusterProminence_AllDirection_offset1_SD HaralickCorrelation_AllDirection_offset7 Inertia_AllDirection_offset1_SD SizeZoneVariability
  /PRINT=GOODFIT CI(95)
  /CRITERIA=PIN(0.99) POUT(0.99) ITERATE(20) CUT(0.5).


羅吉斯迴歸


附註	
已建立輸出	01-AUG-2019 17:38:18	
備註		
輸入	作用中資料集	数据集1	
	過濾器	<無>	
	粗細	<無>	
	分割檔案	<無>	
	工作資料檔案中的 N 列	50	
遺漏值處理	遺漏的定義	將使用者定義的遺漏值視為遺漏	
語法	LOGISTIC REGRESSION VARIABLES label
  /METHOD=ENTER kurtosis ClusterProminence_AllDirection_offset1_SD HaralickCorrelation_AllDirection_offset7 Inertia_AllDirection_offset1_SD SizeZoneVariability
  /PRINT=GOODFIT CI(95)
  /CRITERIA=PIN(0.99) POUT(0.99) ITERATE(20) CUT(0.5).	
資源	處理器時間	00:00:00.02	
	經歷時間	00:00:00.02	


[数据集1] 


觀察值處理摘要	
未加權的觀察值a	N	百分比	
選取的觀察值	包含在分析中	50	100.0	
	遺漏觀察值	0	.0	
	總計	50	100.0	
未選取的觀察值	0	.0	
總計	50	100.0	

a. 如果加權有效，請參閱分類表以取得觀察值的總數。	


應變數編碼	
原始值	內部值	
0	0	
1	1	


區塊 0：開始區塊


分類表a,b	
	觀察值	預測值	
		label	正確百分比	
		0	1		
步驟 0	label	0	0	24	.0	
		1	0	26	100.0	
	整體百分比			52.0	

a. 常數包含在模型中。	
b. 分割值為 .500	


方程式中的變數	
	B	S.E.	Wald	df	顯著性	Exp(B)	
步驟 0	常數	.080	.283	.080	1	.777	1.083	


未在方程式中的變數	
	分數	df	顯著性	
步驟 0	變數	kurtosis	3.008	1	.083	
		ClusterProminence_AllDirection_offset1_SD	1.153	1	.283	
		HaralickCorrelation_AllDirection_offset7	1.361	1	.243	
		Inertia_AllDirection_offset1_SD	.127	1	.722	
		SizeZoneVariability	2.594	1	.107	
	整體統計資料	7.704	5	.173	


區塊 1：方法 = 輸入


模型係數的 Omnibus 測試	
	卡方	df	顯著性	
步驟 1	步驟	8.726	5	.121	
	區塊	8.726	5	.121	
	模型	8.726	5	.121	


模型摘要	
步驟	-2 對數概似	Cox & Snell R 平方	Nagelkerke R 平方	
1	60.509a	.160	.214	

a. 估計在疊代號 4 處終止，因為參數估計的變更小於 .001。	


Hosmer 與 Lemeshow 測試	
步驟	卡方	df	顯著性	
1	2.975	8	.936	


適用於 Hosmer 與 Lemeshow 測試的列聯表格	
	label = 0	label = 1	總計	
	觀察值	期望	觀察值	期望		
步驟 1	1	4	3.750	1	1.250	5	
	2	3	3.528	2	1.472	5	
	3	3	3.256	2	1.744	5	
	4	3	2.925	2	2.075	5	
	5	3	2.710	2	2.290	5	
	6	2	2.438	3	2.562	5	
	7	2	1.996	3	3.004	5	
	8	3	1.635	2	3.365	5	
	9	1	1.215	4	3.785	5	
	10	0	.547	5	4.453	5	


分類表a	
	觀察值	預測值	
		label	正確百分比	
		0	1		
步驟 1	label	0	17	7	70.8	
		1	10	16	61.5	
	整體百分比			66.0	

a. 分割值為 .500	


方程式中的變數	
	B	S.E.	Wald	df	顯著性	
						
步驟 1a	kurtosis	.209	.567	.136	1	.712	
	ClusterProminence_AllDirection_offset1_SD	.708	.445	2.524	1	.112	
	HaralickCorrelation_AllDirection_offset7	-.478	.526	.825	1	.364	
	Inertia_AllDirection_offset1_SD	.058	.339	.029	1	.865	
	SizeZoneVariability	.712	.398	3.204	1	.073	
	常數	.153	.317	.233	1	.629	

方程式中的變數	
	Exp(B)	95% EXP(B) 之信賴區間	
		下限	上限	
步驟 1a	kurtosis	1.233	.406	3.746	
	ClusterProminence_AllDirection_offset1_SD	2.029	.848	4.859	
	HaralickCorrelation_AllDirection_offset7	.620	.221	1.738	
	Inertia_AllDirection_offset1_SD	1.059	.545	2.057	
	SizeZoneVariability	2.039	.935	4.449	
	常數	1.165			

a. 步驟 1 上輸入的變數：[%1:, 1:	
