# Supplementary material for: High-order radiomics features based on T2 FLAIR MRI predict multiple glioma immunohistochemical features: A more precise and personalized gliomas management
Source: PLoS One. 2020 Jan 22;15(1):e0227703. doi: 10.1371/journal.pone.0227703 (PMC6975558; doi:10.1371/journal.pone.0227703)
Supplement: S3 File — (ZIP) [file pone.0227703.s021.zip › statistical analysis/ki67/spss age.doc]

EXAMINE VARIABLES=年龄 BY label
  /PLOT BOXPLOT NPPLOT
  /COMPARE GROUPS
  /STATISTICS DESCRIPTIVES
  /CINTERVAL 95
  /MISSING LISTWISE
  /NOTOTAL.


探索


附註	
已建立輸出	01-AUG-2019 16:39:03	
備註		
輸入	作用中資料集	数据集1	
	過濾器	<無>	
	粗細	<無>	
	分割檔案	<無>	
	工作資料檔案中的 N 列	82	
遺漏值處理	遺漏的定義	應變數的使用者定義遺漏值視為遺漏。	
	已使用觀察值	統計資料是根據所使用任何應變數或係數沒有遺漏值的觀察值。	
語法	EXAMINE VARIABLES=年龄 BY label
  /PLOT BOXPLOT NPPLOT
  /COMPARE GROUPS
  /STATISTICS DESCRIPTIVES
  /CINTERVAL 95
  /MISSING LISTWISE
  /NOTOTAL.	
資源	處理器時間	00:00:01.03	
	經歷時間	00:00:01.06	


label


觀察值處理摘要	
	label	觀察值	
		有效	遺漏	總計	
		N	百分比	N	百分比	N	百分比	
年龄	.0	24	100.0%	0	0.0%	24	100.0%	
	1.0	26	100.0%	0	0.0%	26	100.0%	


描述性統計資料	
	label	統計資料	標準錯誤	
年龄	.0	平均數	45.958	2.5162	
		95% 平均數的信賴區間	下限	40.753		
			上限	51.164		
		5% 修整的平均值	45.741		
		中位數	49.000		
		變異數	151.955		
		標準偏差	12.3270		
		最小值	27.0		
		最大值	69.0		
		範圍	42.0		
		內四分位距	20.5		
		偏斜度	.111	.472	
		峰度	-.847	.918	
	1.0	平均數	52.038	2.7030	
		95% 平均數的信賴區間	下限	46.472		
			上限	57.605		
		5% 修整的平均值	53.124		
		中位數	52.500		
		變異數	189.958		
		標準偏差	13.7825		
		最小值	7.0		
		最大值	72.0		
		範圍	65.0		
		內四分位距	16.5		
		偏斜度	-1.305	.456	
		峰度	3.393	.887	


常態檢定	
	label	Kolmogorov-Smirnova	Shapiro-Wilk	
		統計資料	df	顯著性	統計資料	df	顯著性	
年龄	.0	.128	24	.200*	.954	24	.328	
	1.0	.126	26	.200*	.913	26	.031	

*. 這是 true 顯著的下限。	
a. Lilliefors 顯著更正	


年龄


常態 Q-Q 圖


÷ZòäIy+cëüQJ¬EE¹uë¬©¯¯lÕD+Ý3dáÁ±[ÖË×699)½yó¦úï¡±±qóæÍEEEmmm*·³ÉöfÙ¿ÿ<_Û¢®"±Ê ¶åèÑ£)¬¬«GþvikdÆ5cccó´MFF³Ù¼mÛ6m¥ºXÖkSì<W%&&J³ÃÂÂRRRdå¡Cdýï½§kú«¼úê«²^>©¬×ÆbywïÞ½ÚUÒÒÒÔå/^¼¨ª&®¤«³³S>¤¶K¿¥ò¡Ù_¡ªuTTº+ÿÈ¼;Ïýä½J@VåfY#@VAVUø=«ódõÞ½êþ_|!oeæSïÊ)aøà´It²ÊG?ûì3mÙáp¨ÆHôÙ·o¾@.ììÚµKW¾6Å¢*õùçËGåüü|[É¹|²àr¹fßºØñãÇõ+Õ&Køçüì½>«ÚÔ.k;&ê`²²¬22)æææ¬Èª¤4 ¬jáÜ¹sê¾ßx¨RÝ×ÚßßãÆU¯ëj©P>ÔÝo¬£¿Ì§~ª-kËCÊ8(_ÉíÛ·õ· bÃÅÅÅrýû÷«ûµÇV¯]»&Ëv»ÎÝbµZå£(ËíËÊ¤¤¤e¹ÊêÙ³gwÌÐ¶E=ÈªÕìóßóU`þÉ[¯×+³¦Ì[êºê>XíÕÆÆFí*[¨*¢,KKdùÖ­[²òèÑ£²üÃ?H>­=i¬FFFÊpéÒ%õYÔuµFÖW,ÿÈÅÔ³Ô2ÊD8888çnÏù4¨yØµØ«hºÌÐú«iU¿idXeÎ9£4ç´@ÆJQyë!ÚK_|>zPp¾ß%AðªÊLQQÑc³*×x²¥¥eö%ëëëÕL«ÙÙÙÚedPsFÊ¤;çgsWQÛ+C³lNZZ¶-2­ªÒÒR²²¬JjÓY3;«j<Õ_ø»ï¾bÉÂ7ß|£ú:C?ïÜ#ú¨iUÞ,¿ýöÛÿðè¬öøkÀuåÐßþY=É$RËgÏUÓªzUÙ¹s§¼Ý±cÇìëÆÇÇ«ËÏngTTöhx9³ª¾UPåÿíÁi²²¬SSSCæ´G+µ;õ_õZÕôôtén]]Ö~øAÝ¬ßzë­y¦UVNgÀÔÛ T_­ö%©WÉòñãÇåÝÇVÕäõÑ½÷<ïIÿ¿B©»v¬|ù¿Am¬¶]Y]ÈUUù×D¶Âjµªíïï·ê1o²²¬&Zõæ¿XeUÝ[ëÖ-õ#Òº»»Õ#©òî¡Cæû]zïÝ»§lp-mú¸i[¹zõªø¡îÑÍÞÞ^YVOWû|¾G=ÁXÝ³-S¦Ürgg§DýrÙYÕFµ¬¬kÄB²zóæM5ð©»UïÍPQGA?«úÇVe!à%í0Cúkém8;«R#ýåûúúÔ!*®(òÅ8pàQGhaö±æ?¶â¢®¢Ïjiiill¬ÝnW;DLêwÞ!« «ÀZÐØØ8OVÕññÇ=V%MK©þðIsfUÿºUír#EÝ¾þ@¾Ve>/R¾cÇÜ	¬¥^Æ:çñÕÃÃê0ú:=fµZëëëÕc±óoÚÂ¯ÕÙ_Þo~ó²²¬ê9´ÊêÝ»wûûûµw¥LcâB²¤=[8àiMêÉJs>÷GBö®¥*hÀáµã æææêÇGí]á9_4§ÉÉIGÕÈK¿îSRR¨ý½÷ò3	²¬bß|óMttôJÿÔ½váiÔÔêuÃóo¦[²È*d²Yd²Yd²Y¬²Y¬²ºØØXíÝ'OÆÇÇ³[²<ëßK¿ÌR.¯¹ÿ~IIi,<xð`Oqøðaý»|7²Õ?*+++666Ö××ËÂþýûg_¦¿¿ÿo¾=VÞ9sæÖ­[²°uëÖ¶¶¶WgðmÈ*@VJå÷îÝ»÷®,dddÌ¾Ì¹sçdÕ>Qww·*±ZsâÄèèh¾­YVoÞ¼váÂýedLHHp:~¿_­þíÙ³G./+--ZbVåv´+ÊÏy²*ÓêÅµÆÆÆÎcdxÚY I¥dÁçóÉJÅ¢¿óäÉ²°wï^µþ>weeKK,8p à6sç	ÓgUêþØ¬^¾|ùÚµkmmmZVeäåÛUàÙgUôõõ¥§§ËJþ22NNNÊBTTZ¿yófyWVNOOËBbbâ§ÕfU>ôÞïIJeáÒ¥KêÂÅÅÅ² £³Ü|[²<û¬?~wíÚÕßß¯_°¬ÕNUPá ¦Õ înnnV5Ô²U`EduÃ²<=cvV'gÈÙlVë£££Õåç.##C=eI,ð)K*«7oÞPk²³³ù¶dxöYµX,²<88XWW7;«eeeê-EEEjaa¡zîÕ«We!---¸¬jÿøãÕs£æyÍYÕßT^^ßV¬Ï>«mmm±±±QQQ¥¥¥³³úí·ßª'Ý¹sG­×Þ cî;nÝºµÄ¬ÊæççË­EFFÊ-Ë»sfuöc«wïÞ=ú´öJV¾­Y° ^¯Wë±Ä^-OLLè6¬@V «¬@V «Õêÿùãââ,<k/¼ðBww÷êÎêï½ugí¿øÅ¿ÿû¿¯ú¬îØ±ãµ?ÿó?'«U² «d@VÉ*d²JVd¬È*Y¬UY%«²JV «U² «d@VW`V322ÂÃÃSSS;;;É*¬OjzúôiY¸téRllìÿþïÿåÍ7ß$«²ºOÃñã?æòÆoð½ÕÇv8qBÞ0ËßüÍß0­Èê";wnóæÍ<¶ «Ë#<<¬ÈjðeáòåËÛ·o'«²¼ÞÞÞÔÔTS·mÛvûöm² «@VÉ*d²JVd¬@V «d@VÉ*¬UÈ*d¬È*Y¬@VÉ*¬UY%«UÈ*YU² «d²JVd¬È*Y¬@VÉ*¬UY%«U² «d@VÉ*d²JVd¬È*Y¬UY%«²JV «U² «d@VÉ*d²JVd¬@V «d@VÉ*¬UÈ*d¬È*Y¬@VÉ*¬UXü~ÿÁÇÇÇÙd°$6Íd2?½AVAòù|!77whhBVÁ©q3jkk'&&Ø!dÑÑÑüü|£Ñ(Cª¬ì²Ò©S§bbbdHmmmeo¬¬^¾|9---<<ÜápôööUxrrrCaa¡¬ìµÕ¤¤¤K.ÉÂ'RRRÈ*<iMMMf³999¹¹¹½±Ö²ª9==ýç³ÄÇÇ¿ùæ|/`FFFdH5EEE¼2ugµ»»[¦RÉjÉ,©©©dbbbÂãñL&ÃÑÕÕÅYãY½wïÛí¾ÿ>wÀR].©ååå¼~fígulllïÞ½·oßæÀ°ìdH5ÍN§s``½±ö³êõz·oß~çÎ^`ËK:*5¦VUU1¤JV:dÎï÷WTTFËÅZYåp°¼¼^orr²©!¬UDT©yyy.¬U^WWÝnihh`oU²AòûýEEEjHaU²Aêèè!Õjµ:u½AVÉ*i||¼  @ÔÂÂBDHVÉ*OfSëÎéFVÉ*ÏçóÍR'&&8¿Y%«°8MMMqqqÃëõª5###yyy²R&WÅRWWÇ^"«dcxxXò)CjYYßïW+e!99¹¤¤D­ÒZî&«dÃãñÄÄÄÌ>§[MMMNN~Msssff&¬UHÍÍÍR5ÅÅÅRÖ&ýFVÉ*:xð 4ÒårõôôÌyòòò[,vY%«ðGN§SZ]]=Ïáòåï\þð¿ì@²JVàw$¢2.ünUUURV[kkksrr¯´!«d~G:ùØ!uöÌ*Y-**ª©©ápKd¬ÀïDXZZ*Aånd¬À¨sN7²JV`I&&&´sºñ(Y%«¼ææf5¤rN7²JV x>/77×`0ìÞ½[Ù!d¬@<Ùl¶Ùl¼¬U:È©x¬UÞÄÄ:¡Ýn?þ<;¬UROOÔÔh4/ü  «dTu$ÂÌÌÌG.d¬Àãy½^R#""<ÈJVÉ*Éï÷2¤U²KÕÑÑa³ÙdHõx<©d¬@FGGeHÍÎÎ^È9Ý@VÉ*Ì­µµ5..Îl6×ÖÖ²7È*YàÔüü|Áàv»GFFÐgñù|eee999»wïæD7d¬X¤pq3hê¤©6MÚÞÞ.Èn·±óÉ*Y°vÈ`*ãéRõùCU²`í¨­­5Í2¤>¶Y,³±VUUð «dÀê644­ÿÔN<.ýöûýú5555ÅÅÅ|;È*Y°y<ÉdµZÛÛÛæçÍÊÊª¬¬ÔÞp:RV¾#d¬X´sº>ýsºõôôÈÀZ^^>00àõzsrrdb_AVÉ*Õa%ÓMºîv»åkºWTTÐT²JV¬>2&JÆ8§È*,vN79È*sº¬À2àn «°<äÏ¥©&sº¬À2©999CCCìUXêZYYÉ²óñù|òwPÞÎù¡TUx¼ññq·Ûçr¹bbb¤ú£ø655É,KCCC*È*<:é·:Ö ßï/,,ÌÊÊRCª:§[^^ÞS,@Vàÿèéé±Ùlú1TeÍ¡CÔ*Ó*	däÔ©Sùùùú52¶¾ôÒK2¤=ýÃå¬U«üís:Ú»2¡>÷Üsþ9;d¬Xu¯Çãñù|¹¹¹2¤îÜ¹óÅ_|jçY%«Ö¾¾¾üä'áááqqq¿øÅ/dZíêêb·¬U644³aÃRÿéþ©¡¡9d¬FUUÉdr82°²7@VÉ* uuueffÆ¿ßÏY%«111QYY)CªÓé?|ìU² HêÄãf³¹ªª#¬UAÿ»¿û»°°°6lÚ´Éårñt_tVïÜ¹@V7¤&&&®_¿þõ×_9µººÚb±°sYíìì´ÛíÃ¿îYþò/ÿ¬ -,,4)))»víÒ¨¼¼|÷îÝì"bV¥×®]Ó²úã?æòÆoð½ immµÙl2ÖÕÕýÕ_ýU»þ£2ª:öB1«¿ÿjtÓêÿ7Ë¯ýk¦UÏçËËË?¹¹¹ÃÃÃ²FÓsÑÈ_½ôôtöÈ*­Ì¦ÍÍÍúv»]Fényy9»« «W¯^Íáv»åÇZr888¨­Q+>,ë¯BV,LÕª¨¨ÈjµVUUÕÔÔ8NùÄ °:²:999==­µÐd2©5òî¾ûV,»ÚÚZ³ÙÜÚÚú¨ËtttKYi*VMVUÃÂÂÚÚÚÔÈÈHYVgÍ7gddUK700àr¹äODaa!ÊÇÚÌªª >²üÅ_hï&&&¦¦¦r8KTUU!C*wÀÍê¹sç=*)mll·²FXæÔüü|5¶îÝ»¬Z__Óé¦êI*))ÉÉÉ)((ça`ÕdõÁòöþýûÒÎ7nÈÛ/ÊÛÓ§Ok±Z­iiid@&&&ÊËËM&SzzzÀ9ÝäÝ¢¢¢öööÅRYYÉÃêÎª¢²ºoß>¿ß¯îNLL)Vz0Ó* ¨×JS«««g.ßåré;:<<l69*ÖNVÏ;§=¶züøq¦UAÿÑËÊÊ"""NçóÈ¨ð_Àì=¬Á¬JJõÓj~~>Y°ð!Õn·Ë*Ãè£Îé6>>.gVJ«ªªØXkY=sæü©v¹÷.YðXËââb£Ñ¥D8Í¦?ö¯üÙ	X¬¾¬Þ»wojjêöíÛêÉJ³_r£,e%«æ×ÚÚ*hLLLmmíBN<ÞÜÜ,njjdggçæærÆr¬î¬þðÃ_|ñ3>>^ßT»Ý®½àÀõ<a²àQCª:§[NNü¾ð+:uÊápDDDÈZRR2û(À*Ëª¢ÝÇë÷û¥ gÏÕTÆYYyæÌ²`Î4Ê§?ÒYÊÈ*°6È`¯Õ¨T¯×[]]íñxzzzØ? «dÀBÕÖÖÆÄÄÈ*)ý~æ	Gn·Ûf³L¦öB4«òÏæÇ¬ÔQõ;;;GAAA[[[BB¯[ ñù|999F£±´´T; qq±ËåÒ^:00`6Ï?ÏîBfõðáÃj!==]N>-Ë7oÞåææfùÇ¬ø^wN·dÊpØ²ì1VV»»»Õ¤¦jÁívkÝ¿uëÊjll,YBÜÈÈHvv¶©ååå³O*­xm¸¨¨ýÐÊªvÆòèèhÕwÞyGÖ¨×hÓ*YB¥Óáp<ê½N§³©©I¿&//Ïãñ°ëZYÕî>rä,L¦­[·jYÉUÂÃÃwíÚEVÐÔÓÓ#É?UUUó«¡££#&&F#I.vðàA»ÝÎëPºYsZåN` ©sºÆììì¡¡¡Ç^^þVØl6Å"ÍÌÌUPVUboß¾MVÐäõzeÜkhhXÔGGGRAV³úõ×_ËÊÂÂBîBßï/--!Õív/êHYxñâEu¯ÔTdÙl6ðÁ'NPaZBpHmmmeo¬.Úää¤ë3g¦¦¦.]º¤Í¯o½õYBjHUËÏÏÏ×òÕESgñTåUY>yò¤¼íëë#«@ÙÔf³Y­VTÕ%eullL*áÔÖôööÊ¶¶6Yöù|ê.bmr%«À#©:ª<ÕduIYìîî=¹JMµ57nÜPó+YÖ¸¸¸ÙG"È*g°°###n·[.!d¬Û»ï¾òÚk¯Jpöj·Û9+*°üÚß9Ã=£ùÔ²zõª,U`åî¹çÖ­[°iÓ&ùåýõ×g©Úáòç9!@VÌêÔÔ:ÚþìCC0­«Õj^^¿~]½èÐ!ùu·Ú<ÙlÎÌÌdHTVç9âYVS=ª_óÓþ411Q²²²"""<ÈU'¿¼k^íµM6IJM&Ëå8Í8'ÕééiípYUÇ`"«Àª°~ýú÷ß_¿Fji6=C*ðô¦UJÕ1ÕSÔ2YVí~`è/¼ ¿Â.xÆYåN``JII_a[3~õ«_±O² HêpùÕ?û³?»rå; «ôÇ²JVüÑèè(çtVGV'&&È*°µ··sN7`EdõÜ¹syyyê¿éééÚ³µåúúzy7<<¬+sH-**!sº+"«<àPûÀ*ÕÐÐ TVÙÀÈ*g°V£ááá¼¼¼²²2T`%fÕï÷·´´¨c*Éÿ¿)))êKáÛo¿%«ÀÊQSSc±XËVô´zéÒ%¨ÏçÍÈÈPÏ]ø1­+:§üb0¤+7«ò[êt:ÃÃÃå­:è#G=ªÎ½*¡MJJ"«À3T[[+¿6íüùóì`åfõæÍÕÞÞÞù»»sçN²<ééé&©²²!XéYdnÛ¶MäW×ápìÙ³'F[[üïÛ·orròÆÜ	<ÓXeYÕ¿U«/(­ÏvHÍÌÌ4ÕÕÕÓX­YÝ¹s§:.ÄÞ½É*ðË-qe«/«RÐýû÷ËÛùenkkwëêêûÈ+Y×ëµZ­òkèñxu±±1¹ÌºuëÔ9à~ùË_²ßÕÏ>ûL-Ë¯kZZþCW²<i~¿¿¤¤$"""++khhhKÊeF£:]ùûï¿/Ý²e;XYYÕÚ®ÿÐ'È*ðD555ÙíöÚÚÚùI÷Ýw¥£×¯_×ÖH_å÷ô?þã?ØÀJVµÃAÈ/¹zµ¿¿¬OÈèèh^^ü¢åää<öò©©©			+ÃÂÂ>üðCv&°²ÚÒÒÕééiYÿê«¯ò%à	ÿVë¢N<þË_þÒl6¬ùõ«¯¾b+%«zQQQo¿ý6Ú¨¡¡!RFcIIÉ¢N<.ÿËÿ»úÙtëÖ­Õ±±1ö*°³Êl'­¶¶Öd2%''wN·)ëOúÓ×^Mþ¨,²ááaÅòòr¿ßôí=ztË-?ùÉORRRþõ_ÿU²Ð211QQQãp8¼^/; «dROOÝn7¥¥¥. «dßïJPeHåHY]´ÞÞ^ÅzáÂ²PÖÕÕe³Ù"""*++9@Váv»eáØ±cz¡YE(©êpùN§sºd5x±±±ÓÓÓ²099 ËÿoÄÄÄ7ß|ï%Öªöööääd³Ù¼À!U.ÓÜÜ,®««[ÔkX¬ý¬ë%«¿eË-dkÒøøx~~¾ÑhÌÎÎ^à:22âp8L·n·;&&¦««=	ÕßÓ7lØÀÀ­­­ET:þHªµ´´T·¡¡Ájµòla¬þÞæÍ'''ÕÀ²LV"CjAA©2nú|¾_Q.,h°Óé<þ< «¿³gÏúúzY·ò'¬bÍ;uêÔb¯éêê¬ÌÏÏâ¦¬Í¬vvvÆÇÇY,Ë/U¬aÃÃÃ¹¹¹AFÕÓm¶ÑÑQ³Ù¬L®6M~ÉÙ½Yåp!uuuRDI`ssóRn§¨¨(++KUZ\,ïò"W¬ULÝn·Ñh,,,s¤ r;RhËe±X¤©ztY%«XÅ<R;::ñfeZõz½ÃÃÃìa¬Uììluâñ¥ÓY%«Xûúûû¿úê+y;çG<h22339V@VÉ*0ë×¯¿øâëÖ­3Íë×¯ÿ?ù«W¯jíëës:2¤s¬Uà1¤£/½ôÄõûÇ8_~ùåèèèïgL¤©Ó «dx¼S§Në+eY³ÿ~»Ý¾ðÃå «dø¾´´Ôf³é×HV7mÚ´~ýúÎéU²,ÂW_µqãFí]ùíJOO7öìaçd¬£îòýÕ¯~5>>^\l4ä­þYKÈ*Yª¥¥%,,lýúõÒ×M6Ércc#» «dX4u$BÁð³ý,++ëoÿöoS²JV`444ÄÍàDlY%«ÀÔììlRóóóõçe@VÉ*°PeeeÔÈÈH«ÕÚÚÚÊ>È*YQ\l±XeHÝ¼yó_üÅ_pdÆ©S§âââþyRÛÛÛ%¨999ì¬U`qúúúd<!µ´´T;×ëu:ì¬U`¡´ÃåGEEUUUé?444d·ÛÙEY%«ÀôôôH8Õ9Ý>üðÃüü|ýG%·»wïf/d¬R%¥ÔÌÌL«¬ñù|6­¬¬lttT>ZSScµZÕU²Nê%?©ÃÃÃOÿS½^R#""dÕ?×WÊ*«Åb1ÍN§S¾<¾MÈ*VÁ¤XTT$éIQ&By;44ôt>µßï/--Õ©@V±ºIS³²²Ô¡Ô±ÉÉÉOá¢6MTÇÃRU¬RÓÃº¦¦¦'úIeHÍÎÎæÄãÈ*Ö®®.hÀJX<ø>ckkk\Ùl®­­eÿ «XS|>Åbñûýún·»®®nÙ?vN7y+Ëì|dkPVVVQQöè¦:ùÚ²%sº «d5$HA³³³­VëîÝ»N§,,ïKY´!sº «duM®«««ªªjmmxòmWWÌçÏ×Á»,jkkÍf³©ÓY%«kJSSSLLLAAAyy¹ÝnÏÊÊxHuy©2¤ «duMéëë¦j÷îÊ¨[RRò>Çã1Lênì|d¬®5eeeú56íIô[æ`£Ñ¨?§U²º¦×ÔÔ¬XöxËmÚív¯×Ë>@VÉêU]]§_ÓÞÞ¾§/Ù733SÓ# «duµZ­Ò<uÇ¬LÉÉÉËòRuaÉÎáòU²*FFFrsscbb¤¯eY(?ÜçÊÊJTd¬Ïç[Û)))1.Ãå «dÁkmm×l6?CYE¨/..!Õív/ËÔd¡;¤&''ÇÄÄ<Ñ³±YÅÇáòU²¢®_¿þúë¯[,_|±  `éþUçtlnnf÷ «d5ô÷÷?÷Üs/½ôÒoûÛ¿ÿû¿xùå¾5Ï·÷n5¤òH*²JVCÍfKMMÕÞQ5<<¼¢¢"ª­­!Õáptuu±cU²ÂÂÂd`Õ¯ïÝÏþóEÝÈðð°ËåRG"|¢'²mýúõ!üõ¯ýÊ+¯,üª««Íf3C*²JVñtt´tT¿F°æQÔ9Ý8²JVñ;ëÖ­ÊÌÚßßÿòË/oÜ¸q!/ñx<Y%«,«Ì¬ëf¼ôÒKµÎÖÓÓi2ª««RU² IDËÊÊdHÍÊÊb «dAòz½v»=..Îãñ0¤ «dAòûý¥¥¥F£< «dKÒÑÑa³ÙdHmmmeo «dA-,,!UÞr¸|d¬"x2ªÃå···³7U² ©sºÉZ\<>>Î@VÉêÚ7::Z]]]TTTQQÑÓÓ³«ÎéÜÑÑÁN@VÉjHèêê²X,ùùùµµµ¥¥¥f³yéÏ'ÊÎÎ!Un!YzîÜ¹@V!«Õ*£ªöîùóç¥¬KyéÇã1LéééòÃÇî@VÎÎN»Ýn0üñë¹>Ë_ÿõ_Õ'g`` 999à2h677qkN§SÔÊÊJò¬>mÒËk×®iYýñÇsyã7ø^>!*+wïÞêÔ©EÝDôàÁ2¤Ê­Ém²cÕgF?­^å­·ÞbZrü~¿ÙlÖptt4`ÍcõôôØíö)+C*²º²Êc«OÇã±X,êÉºRSËU\¼ð*«#ÊÊáòÕgQ¬®MMMCÆMÍ&Ä©/£-Ë@VV<RÕsrrÙ!È*YEZ[[ÕáòØÈ*@FGGóóóåß ·Û=22Â@VÉ*¤DÈ9ÝU²ß¨©©©®®^ÔAze0ÍÎÎ!UFUTd¬âwêêêL&SAAAYYÍfËÍÍõûý½Çã1ÍÚ+p¬Uüî©111ÚAz%¨2Ìs¡¡¡¬¬,Îéd*++eHÕ¯õQWG"?½dÿL555+#""f_rxx877WTÉðBî%²JVCNuuu^^~M»ÝnsHõ]]]ì4 «Ûèè¨Õj-//WvttØl6ýñzzzN'Ë²r¹11Ú©ß$¢F£133SâÊ²òûý2¹jïvuu¥§§ËÊáò¬"xÑ	ª°ì «Òùóç9§U,Éèè¨:§[vv6G"²àqN7 «dusN7 «du477[,Îéd¬.:¡:§þu5²Åihh±Z­©@VÉêTuâñÂÂBÎéd¬Ox<99sºY%«KRÕÇKKKR¬Õ MLL«sºq¸| «d5xò=VËçn@VÉêRTÎéd¬.×ëu8©@VÉêÊÊ9Ý¬Õ%éèè°Z­ÓÈ*Y]´«W¯þüç?ß¸qãóÏ?¶k×.Îéd¬ÙÔç.))éÔ©S~øaXXØºuë>ÌO6U²ºh¯¼ò4utt´¨¨(""ÂívoÞ¼ùõ×_ç'È*Y]´7þÃ?üCÜææfYSQQa±XøÉ²JVÇçóFuâqínÿøÿHV¬ÕÅ©­­5ÍáááçN` «du¡Ì©eeeýýýÚSÍfþùç¯_¿ÎO6U²úxUUU&)==]¾mjÍÕ«W_yåçgÈk «dõñCj^^Rý~??»@VÉjª««eHÍÊÊêêêâ§È*YR___ff¦4µªª#Y%«Aòûý.kxxV «d5H6ÃåY%«KâóùòóóFcNNçt²JV×ÚÚªDØÔÔÄO'U²$u¸|ReTM «d5HÍÍÍêpù²JV122c0R¬ÕàUUUÅÄÄØíö~¬ÕàDDDód¬.÷úY%«UÈ*YU² «d²Ê÷@VÉ*¬UÈ*d¬Èê]¾|9---<<ÜápôööUY^RRÒ¥KdáÄ)))d@VGddäôôô³Hnß|óM¾²ºPÝÝÝRPÉê³¼ðÂd@VêÞ½n·ûþýûÜ	 «cøõîØØØÞ½oß¾Í3duI¼^ïöíÛïÜ¹Ãldu©:d@V9¬UÈ*d¬È*Y¬@VÉ*¬UY%«UÈ*YU²Y¬êoÜ¸1eYmÙ²Åf³¥¤ýìgú§²ÛÛ²Û,Û.oCsóeÛå»²Û.¿õËÒ£UÕ/Ëå²Ûí_¤]»vÅÇÇæ¶¿óÎ;6lÍm/))1ÿò/ÿÛ~àÀÙöà¶<Ùöûöæ½ü¾,ïm644ü÷ÿ÷êÎêðþûï¿ñÆ¡¹í¿ýíoå_ÐÜöúúú¨¨¨ÐÜöóçÏË×ÿùÿ	ÁmÿÏÿüOÙöáááÜöï¿ÿ^¶ýßþíßBóÇ^&ËÆÆÆ§ó¹È*Y%«d¬U²JVÉ*Y%«d¬U²JVÉ*Y%«d¬U²JVÉ*Y%«d¬ÕÇùè£Þ÷ÝÐÜö;væ¶·´´¤¥¥æ¶_ºtiË-÷ïßÁm¿~ýºlûý×à¶Ë?R²í/_ÍûW^y¥µµ¬°ÊUÈ*dõ»|ùrZZZxx¸Ãáèíí5òÖb±ÈÔÔÔ.¬ámÌÈÈP[ÚÙÙRÛ®;wÎ`øýÏ|èlûíÛ·:!µí<Ø»wï½^o¨ýÌë¿ïaaa!µùòí¶ÛíÏäÏ]Èe5))éÒ¥K²pâÄYp»ÝêbÇûí·×ð¶ËÓéÓ§Î<i%666¤¶]LMMÉTZVCgÛ%-ú5¡³íøôÓO§§§å¬Ífµyl²loHm¾ü»yó¦,ÈÛ§¹í!'pdd¤Úûò['jï¯ygÏa=Ô¶ýO>9|ø°ÕÐÙviªúwJÿ'D¶]þ¼zõjhn»ÆçómÛ¶-Ô6_þy«þ£zjÛºYíîî~ï½÷d!<<©_^«[TT¤EõÚvù5==]~©´¬Î¶'%%mß¾]¶QöÀwßRÛ.[÷ùçË?Ðòµ¿¿?Ô~ß¼¼<ùsj?ö½½½òßwy«6ÿ©mfõÞ½n·[½tO=ä lØ°!6ÿÜ¹s7o©m¿,/^|8óhZßwù·]ÝK:Û.[ZWW'òÿüWß÷k×®mÝºU¿CBdó322ÔÒWµÚ¶bVåËÞ½oß¾­ÞÀLNNªTlBúg-t¶Ýðì÷]ý5	m×o]¨ýÌ+y]¿CBdógÏ¦OmÛC.«^¯wûöíwîÜÑÖìÙ³§¾¾þáÌíd]ÃÛ488øpæéÐ²BjÛõÁïû7Ô?¡ö/..>yòäÃ§Á;ÎüõÕWûúúBðÏL¨êÏÝ+WdrÛrYMHHZ:;;ãããÃÂÂ,ËÚ>²Wooojjªüã¶mÛ65¬Î¶ÏÎjèlww·z¥ËåROâm¿÷n^^zÚµk!ø3¿aÃõ<Pû±Q¯'·²ü4·ÃA@V «U@V «UÈ* «UÈ* «UsøöÛoÃäí'|"k¢££SSSÝn·¼õxäï¼Á Îå2u¾÷ßüæ7êÝû÷ïË»êÄ¥È*°FLMMµµµüñÇ¹'OÊÛCÉ[³ÙìõzõáüòË/µwívFFFþ¹úéÓ§å;wîÔÖ|öÙg²æîÝ»ÚU:;;evÕ¾YÖRÏ;'ÄÊÛ³gÏ>üÃIÚõÓªVÙ7oÊ»·nÝ·Û¶mS+õçVW/--X#õO'7øõ×_Ë;vÈòñãÇeùØ±c|/²¬zIIIß|óMTTN2Y__äÈéLú(8qB-[,IUZ2Ê£©©©jTmll5W®®®fÓÁÁAI¸ZsïÞ=Y#eÿdX[¿±Ã_|¡fSõðgôCI#U)õÓªv-uþöþþ~Yèëë·ê´Þ²¡¿°ê´Ü,«=zTÖh7.]çU`-8sæÌää¤Q=ê÷û'&&TóæVÕ»/^LHHP÷åJeåwßÿöÛoËþöåÆwíÚ¥î7V×ÍËË(´Üß¬kg`¸ê©+Ë¹¹¹sfUÚÙÝÝ-kÆÆÆÔÃ¢Ú«êîß7nÜøàà ¼ýöÛoµ1WÍÁÚ´*Ã.ß¬kzôÈ#QQQuuujv·ÙlÍ ¿X¦OUÇ«W¯Ê[¹XÛõ¤$µ+¥eçVõ£0²¬bô¼UÓçÈÈ¼íííUkäC&I¶¢*Å¸5IéíÛ·Õ§¦¦f·i «Àå÷ûïÜ¹£^ß"oÕ3Ô#ª%àZê®öìÑê¨odRRÒV²õêÏ'oëëëµõaaa¯¾úªReU>*¡UçÎSh¿¼UÝÓ+£ê#ÿ.0­dê%¤jNUo?øàu°W²§rp Á»wïª@JJµ¬ªÃJpa±Óêààà<IÈ*UãÖ­[gÎÑÞóz½ÚÁÔËRsssõ³lKK¼;11!9'&&fgg«ùý~ùPttôì¬~ýõ×êÙOrùiµ¤¤DSo@VÐÒØØ¨½öìÙñËø0°ªÃ"JI²Y¬²Y¬@VY¬@VY¬@V «¬@V «àáÃÿjM2IEND®B`


ôècÕ»¹chÎQ¬­2+Ü^ÆÑ$àóGL4ïõÁY5G6Û÷Ý^¿ÛõÓ¾âW#o]G#¶üü|óx4®U 53«£í¨@ÖÚÚZ³vÚZíxfã,ë¬¥)¨ãÇº7+ÿEEEöá£uÖdxáß°Óã×³ï¨K½YQ±~ýúEµoíòU¬!´%&&®ôgá8ì°ãûWûáGÙÖ`ÝÏ!ø+jõ0gö=ÃOãóÃ,L³ôÂOÓ[²Y¬²Y¬²Y¬@VY¬@VY¬@V¬---ÉÉÉÖÙ³gÏ¦¥¥±X²Dð¿Íï~ïr£°°0ä­¦¦¦ª««ãæhâÉ'üÝØÏódX[Y½sçN~~þ|·ª­­Õüæææ¦¦&M¼óÎ;Á×éïï?þ¼.½páN;::ÆÆÆ4±iÓ¦öööÍsx­²¬¬þÍw+sÑãÇ=z¤7_çâÅËZ÷ÖÓÓcJlæ9s&11× «@D³:::ºeËØØXËµaÃ®®.ûu4XLOO÷ù|f¾RWZZªë+i555333ËËªßï7Éy+Ý¹5_V>ÈªF«/_¶.MNNcdxYU$Mh¦Ûí¶_Gá<ö¬&vîÜiæïÚµKg5³¥¥Euuuûe©cÜèèhVüE³zãÆ;wî´··[YÕ× «@D³*µµµ^¯W3Õ3ûu4ÖD||¼¢³9;;«ÌÌÌåVJVuÑ[o½¥jâÚµkæÊUUUÐxZ£p^k¬ÍêñãÇ5ýÆoô÷÷Ûç;¦­°àY~Z£Õe¬¦¦ú|`æU¬ÎjLL¦gçguz&ÌüÄÄDsý0¹¼ÑêÆÍ&Kæ&K&«£££fN~~>¯5@VfÕívkz`` ±±18«µµµf'ÊÊJ3¿¢¢Âlp;88¨6<Å¬Zg÷îÝk6Z`YµßUqq1¯5@Vfµ½½=999>>¾¦¦&8«.]2Ûþ<|øÐÌ·Ô aîë¯¿>66ö,²ª¿RVV¦?«?§³!³üÝê£GÚÚÚ¬=Yy­² ,W¯^íîî¶z¬OfzrrÒ¾-²Y¬²Y¬@VWßýîw©©©n"â'?ùIOOÏªÍê[o½µeË;DÄ«¯¾zõêÕÕÕ×_ý¿ÿüçd²JVd¬È*Y¬UY%«²JVd¬@VÉ*¬UY%«UÈ*YU² «ÏaVÝn·ËåÊÉÉéêê²_4>>eCVdu~¿¿¹¹YGÝ¾»ý¢@ °sçNëì×_!È¶mÛÈ*¬þ^rròìì¬&¦§§ÓÓÓí©©mmmÖÙ¯¾ú**_þò¼Ì²ú?WÈiÉÎÎÞºu«fz½Þ¡¡!Íyäÿñ­ÈêïEGG[Ó111!¯sÿþýÜÜ[ÕE¤¤¤LOOÀïjó¬ÈêÿWZZÚÔÔ¤	úý~ÇJà»wïÑêÖ­[É*¬.B.---::Úívß¸qã÷nnwÇãr¹ôTV² «@VÉ*d²JVd¬È*Y¬UY%«²JV «U² «d@VÉ*d¬È*YU²Y¬UY%«²JV «d@VÉ*¬UAúúúJJJ<Þhêëë'''Y& «dÀrtww'%%544|öÙg~ú©×ë­¬¬d±¬UËÛØØhðàÛí¾råKd¬XE455Õ±Ö·ººúÃ?dá¬UKÎªÆ¦¬ÖÔÔUU²`9B®îîîfÉ¬UKæØd)//M@VÉ*åëëëóûýÇçó½ûî»ì`²JV «d@VÉ*°ºÝ¾»¢¢ÂëõæååÕÔÔ<xðe²JV,³©ååå7oÞ¼~ýzqqqnnîÈÈKd¬X²ò9ö9µµµ,U²`Éòòò4NµÏimm-**bÉ¬UKæóù®_¿nsîÜ¹ââbÈ*Y°döìÑØÔÚU¿>|%²JV,ÙÄÄ:4NÕtAAÇY%«éÁï¾û®Æ¬ÅÅÅ§ZMUqÙÙd¬ø®úúúô6äv»3224e¬UË122ÚÐÐ ÑªÎ~òÉ'êkkk+Kd¬X²ÚÚÚûó+Kd¬X²¢¢"P3ãââX2 «dÀÛêÝn7Kd¬X²sçÎ­[·Î~Xà²²2Ç²JV,Â?2SUUÑÐÐpìØ1½éÞ»w¯¯¯Os*++÷ïßÏ±øAVÉ*°RÝ¼y355U5äÌtvvÖÖÖª¯*ëÄÄDcccRRæ8qÂï÷geeþùç¼4 «dXy¶lÙòî»ïÚçDøGf>ûì³+W®XsêëëÙ6d¬+ªµ¾þXß­jüªÐr&U²¬<n·ûÞ½ö9þÃ@VÉ*°UVVZg#ÿ#3~úiVV9èÑÙÙiv¹¹rå[0¬U`ÅP´Ö­[ç÷û[[[¿¯Ñ_×Ógö_s3ÌÈÈ8sæf&$$h~jjªWU²DÚ~«&9~?|óýÈLÄè/ÖÖÖªîqqq§±±QË-&¥ÈýøE9U²D®LÒù|¾ÎÎÎÏ?ÿ}ÕPoÂ^h=xûðTÏ.++«»»d¬ Æyöo(?þøãzP@³õ>åYVVvêÔ)^hU²DBMMF¨©©©+ñ+Ióãqlrss­¬U Bjkk÷ïßo399´B÷þ,...))±¾L­¯¯WVícñç>ÍråöíÛüË$«dX:;;322ìû¢hðºrU¤O>/++«¬¬,wÎJ9¡>TVVêÞjÝn·^ÄHVÉ*°"UUU­[·îÄJluuujjj__ß~FðüñÇÝÝÝ+h`5ÕÚYÏ=zQØ¬U`ER4¼+((¨©©ayª©Æ©ï³õ«×CVÉ*,MÈm5`­¯¯ÿ.wûgJFÝÜn·YÑþîÑd¬XÓ&''?üðC¿ß_\¬.³ÁáXå[RRròäÉS§NUWW×ÔÔtvvÿtÕK÷©SÝü_üàÁuëÖUVVjDnújEVÉ*ÌKõù|ùùù9^¯7Íªõ«Zg?ùä?üÃ?üó?ÿsÝÛ±cÇÔ!Ç/ñÍGÂ-[¶Íuªäö¤G®O	±¸cY%«Vßþö·ú^^^þé§ÎC½Ñ«aª ²´ì/u'EEEöÑ¡Æ¬ö^ÎgxxØãñèV÷`kü×ý×º­uWç©¬ó=~wuÏqqqßïSzFú àx¹¹¹d¬Xa¾øâ%êG?úÑ_üÅ_üô§?á~õ«_9®£ti|©¦*Zz»×Ø.))ÉþéáÓ8¾5ÔY¥:Ûêa;w®¾¾¾±±Q£Ì¬¬,Ç¬ºhÑq§î!x¨·ïå=§EÉ±ùO¬UÏÅéå_¶¾àlnnVYuj¿NðÈeü¹¼¼<ÇîIêtNNNuuµ¯+8ÆmóÑß8qÂþc|!éÁ-[3×­[÷ý~©G®%l19'«dÀóå?øÁàà ­ùi|G%ÖTÍìþf«åååòýÓ?ýÓÿñ«"fèyêÔ©ÔÔÔpÊª®;f~)Hå¾yófÈôÈuÿ@À£;yvÕbÉÈÈØ¿ÿ±cÇÌ×Ï+èèWd¬øjtttpù~úÓÚçÁñÞ½ôG¤)eeeêáì¯bVÞ*!7çTUU¥¤¤8~NC·pÂIII&Àºy½|ê¥ª«Q¯.¹c«¬ºHA¨¢¢BÓÏÉÞ,zFZÔZ&*+M%«V¤^x¡¥¥Å>GÙsüg>£Þ~ô£ihhÎªjêZÎOIV9¼st«øPEìWÐÄÅÅóàQ=Z³¯§ùêWMµRÚÝÝ­ù!w¼QÝô×c0LVÉ*§Fÿ¯Hk¿û»¿Óøµ¿¿ßq5í46mllÔè°¤¤$99ù¯þê¯ìW0+W¨wçØç/é§÷Ôió­duuõ=ìi<ZPPÀ«LVÉ*¾yiÌÿÃ9íìRPjkk÷îÝë¹ý×ÕTcM"uµp¯*ºzlßcGÈòòòe<=6ÇØTw«O¼Äd¬¨/¾øâßÿýß;::ÂüJ/8&¨!clllzzº	[__®PUUÎ½íß¿_eÕÕ.sss÷s¶R;6­jmmÞîd¬x¾hªøÕÔÔñ¨*¸mÛ6tí;ÏhfBBB;®hÌª¬ª^öOÄ¬ÇpûömU­/AVÉ*ç×ðð°ÏçËÈÈÐ9æÑ¦M×)))9wîÆ»qqqEEEæHL¼Rd¬XIqÕ@sddD£RÇã¸´  À¾hdèÁ³/Y¶zÝn·ËåÊÉÉéêêó"²Bo£¦ÔÔÔð~ú½ÐÈµ±±Ñ:ýúuÕ]VgVý~ss³&=ºûö0/"«XªªªÌîNMMu! jÉ8qÂüòZkk+«6«ÉÉÉ³³³NOO_à¢¯¾úêå /½ôRlllðüÑ[ù/¾¨ºÖÄÄÄLäéUÐÂ×ÛÆ©iii,|?þñë¬ºÓÁýõ×¼úê«¯¼òÊàù³yóæ_üâö9ÿò/ÿ¢O¿þõ¯Y8ÀÊµnÝºç:«ÑÑÑÖ´>Èy+ñü«©©	ÞÂív/?¬Aú4¦w¹ÿøÿ`°xq)))ÓÓÓfM¯¦Ã¼¬âù×ØØg? Ï'|²¤£ßaûâ/þàþÀüèìË/¿üÂ/üö·¿e±ÕE655iB§~¿?ÌÈ*ªÏç³~Y,$%%ExwI¬hUSUVÆ¬duzpiiiÑÑÑúãÆß?¸¨¨ù."«XA<x`~®2..N#×Èï+MuüºÀüÉTTT°dÈ*¥|ñÅßÐñ_°pÈ*Y%ûÁ~ÐÑÑaðÏÿüÏ,²JV`Éì?:;11¡wóþðÏÉäkvv²+ÕÏ~ö³¨¨¨øøø_|ñ¥^r^¿ªiQQQRRRFFÛí>vìY%«°b*]³ù£³ÏzWRRbª×¯_WÚòUÀÓrssíc«¬¹ÚçU²Ëþýûkkk35`]S¿DVOÇÇsïÞ=F«d°###©©©ö¯©@VOMgggBBÆ¬öìñz½>o­íiCVOÓíÛ·Z[[íÉ*YU² «d²JVd¬È*Y¬UY%«²JVd¬À*111qóæÍ¾¾¾5u²JVàé;|øpRRÏçóx<n·ûÊ+,²JV`9>þøãSÍÙS§N©¬###,²JV`É¼^ogg§NYYYCCK¬UX²Ç'NTTT°dÈ*Y%óx<7oÞ´Ï©©©y÷ÝwY2d¬ÀíÙ³'//ÏúÙðë×¯'%%é%CVÉ*,§JiIIIAAABBBkk+Ë¬UX¾Û·o;w.°0Y%«²JV «U² «d@VáñãÇ/_nkkzòä	YÕåPG:µcÇ³gÏêìùóçuZTTÔÞÞÞØØ¨é² «aeµ°°ÐL3­1ëìììøø¸®pñâE² «aeu÷îÝ			¤ÖÕÕÅÆÆjª]]]xÿý÷5=66FVd5¬¬nÙ²ÅLjæï½§kww·¦È*¬.-«öz½fâÌ3¸té¦ïÞ½KVd5¬¬J]]ÝÞ½ÍÙßßßÞÞnf>~ü¬ÈjXYõù|«&§¦¦:::øn@VÃÒÒÒ¢jÆÅÅæh:11ÑL¨¯¨©©1ÃÙíÌJV+5«ããã³³³fw³I°µwÍµk×8¬.ÙÔÔ[3GGG5ÇårÝ¹s¬ÈjXÏ²²²ïVÓÓÓÉ*¬."33sóæÍ_gppðüùód@Vù"ÕÂ9þ9oéÓ§OGEEi¬ÀqøðaÇ§Ó²º4333f`ûÎ¬d´kPMMjÚÝÝ­éë×¯çååUUUÕï¬ÀÚtûöí¤¤¤k¦>ûì3²JVKÓÚÚZRRâ©9çÎ#«K0;;«îÚµ+8«ÓÓÓdÖO>ù¤  À1SsY]þUÓòMÌ4YµàÁqqqÖîînU`xx¬~§¬²Öì5))©¢¢âÄUUU©©©­­­«øùUÀ³uûöíµ¾¾þóÏ?_ÝO¬@VÉ*¬Êää$YÕÅ]¼x±¸¸Ølñëõz­­­é¦¦&óqd@VñäÉµ «üd¬VwV'&&ZZZÌq³²²Ö¯_o[uéÒ%² «KsíÚ5Etdd$99yãÆf`Ñ*¬.m×ÏNccc<xäÈóæmvv6YÕE*«½½½w·°°¬ÈêâCÕ×^M¹¹¹¥¥¥esÚÛÛãââÞ~ûíééé»wï²@Vv%Ö^P¾[ÕïÕÂÂBÿs¬¥;wUY]òªàwÞyG§---qqqííí:ÛØØ¸è7¯d@VYýàÌtFFÆì)®d@VU«iii^¯×~Ñ3gÈ*¬.g´jÂãñoXûûûÉ*¬.!«---¬ÎÎÎjþæÍÙd	@V)>>~ûöíj@VùVQVÝn·ËåÊÉÉéêê²_4>>eCV«3«¹u¶¿¿_sº»»qW~¿¿¹¹YGu¬LöãK|ýõ×¼úê«d°³zÿþE´§§Ç^ÙùFJNN5?×n¿HMmkk³Î~õÕWA^zé¥mÛ¶ñ2V^VU¾ÚÚÚ²²2³/Í3gÌ¯«Ö!íÙE¹Ó½uëVÍôz½CCC¬¬ª¬]hÎ=kRÍïØ±Ã>róÍ7Ã¿Ïèèhk:&&f¾ñqnn.Y¬ª¬:¢¤is(à±±1û<¸èXëSRR4Þ5CaMÏwùKV«'«>ÔÄ'O¬G	ÿKKK4¡S¿ßïX	l~xN£Õ­[·UÀ*Ìªjg~·ÜVÍÌ´´4ëÿõàtÛèèh·ÛãÆëNtÚÓÓãñxÊJV«|´j:<<¬iEMìÛ·ÃAÈê2³*333ÖÌUY]~VCþ¸YÕ%|·j?µÎ¢[UY±ßêGdÖ§¥¥577ïÚµË±¿YÕÐOUsppÐýòË/í<yÒ¾¬È*?@Ä³züøqûÑö­¯]É*¬.Ù_~i?ðÔÔÎ<y2xå0YÕy¿^Ý8§½½Ýív«jísÔÔÒÒRM477kzóæÍd@VÃÚØ±ÓñãÇùn@VãáÃ¯ÍÑØtß¾æVÍ¬f¨ZWWGVdu	¿gnm¦¤ª9.$«²º¸Ë/ochIÃÖ²oiÎéÓ§5Óþãäd@V§¡êÆÙo@V¿ë6wïÞµ~¬Èêò½T/ÐÞÞn_l6Y²LVd5¬¦655ÍÎÑ/jÎ;w­ÈêLLL¨ ×®]³ÏtdÕúIs² «ózòäò9==­¬ZÛ×ÕÕi~EEµy°UY÷öc-±É¬òÃp<Yß´i?H :ú4YÕpÙ¯¯fddXÐE---d@VU¥40çí·ßÕ©¦Ï=«È*¬>Ñj[[YÕ%duýúõæÌwïÞ÷Î;ïhº¥¥EÝºu¬Èê²:66¦Ó7ÞxÃ³uëÖóçÏ³%0¬«­­ÍÚè7%%Å|ÃªéÄÄDM:tÈD¬ÈjXÇZ²F¨ÅÅÅÖtaa!û­ÈêòWUY]~VzzçÌ·ØLë¯UY÷hÀSSS*èèè¨Îp!b_©UÀjÈª(«f3`û÷UÜãÇk¾×ë%«²º¸öööÌÌÌ¦¦¦ù®ðøñcuÇd@VùÈ*d¬È*YU²Y%«²JVd¬@V «d@VÉ*¬UÈ*YU² «d²Y%«²JVd¬@VÉ*¬UY%«UÈ*YU² «d²JVd¬È*Y¬@VÉ*¬UY%«U² «d@VÉ*d²JVd¬È*Y¬UYµøðazzzðüÞÞ^·Ûír¹rrrºººÈ*¬.BÎãñDEx<~¿¿¹¹YGÝ¾»&þo² «¿§(Þ¹s'dVggg51==­áìW_Ê/ùK^fYµ=PYu¹é¯¿þúÿÙ¶m£UY]<«ÑÑÑÖtLLß­Èjèg5%%ezzÚ¬Ö4YÕåVKKK4¡S¿ßOVdu9Y5gõ¸ÓÒÒ¢££Ýn÷7È*¬r8Y%«UÈ*YU² «d²JVd¬È*Y¬@VÉ*¬UY%«U² «d@VÉ*d²JVd¬È*Y¬UY%«²JV «U² «d@VÉ*d¬È*YU² «d²JVd¬È*Y¬UY%«²JVd¬@VÉ*¬UY%«UÈ*YU² «d²JVd¬È*Y¬@VÉ*¬UY%«U² «d@VÉ*d²JVd¬È*Y¬UY%«²JV «U² «d@VÉ*d¬È*YU²Y¬UY%«²JV#÷îÝ«­­õù|^¯·¢¢bddÁ@VÉêrLNNæåå]¿~½¯¯¯²²2##cxxÄ@VÉêíß¿???_qµæ¨¬³òÈ*Y]2¿ßìs4fõz½ü#²º>LOO?>>e³²zîÜ9û7oU «K çñxBVSC·;w®©À>Ï>§¬¬À@V@Q¼sçNÈ¬ª©mmmÖÙÙÙÙ·¬_¿~Û¶m«ã¥,((PY5fmmm-**ÒP¬.YÈ¬fggoÝºÕår©.CCCÊê¶ ?ùÉOVMVMY;æ÷ûëëë<xÀ¿` «O'«û÷ïçæær8YQÇVHgUbbbÈ*¬~§ÀwïÞ5£Õ­[·UY]NVÍÙÇãr¹ôTV² «@VÉ*d²JVd¬È*Y¬UY%«²JV «U² «d@VÉ*dÕ¦ªªê¥^ZÿTefffgg¯GiÿÙýË!þôOÿôW^a9DRÖC$é¹þ©?ÝûTtVsVO>UÿöoÿU]]¤¦¥¥¥,HÒM~~>Ë!6mÚô³ýåIEEEn·ûéÞç©S§þó?ÿsÕfõ©ûïÿþoeõÓO?eQDÒüããÇ³"Éçóýú×¿f9DÒßÿýßÿÍßüË!êëësrr"ó·È*Y%«d¬U²JVÉ*YY%« «dd¬U²JVÉ*È*YY%«du¹¦¦¦^yåk×®±("iÓ¦MgÏe9DÒßþíßþîw¿c9D>ÇüêW¿b9DÒÑ£GÉ*+Y¬@VWÞÞ^·Ûír¹rrrºººX ÏÎ76lØ E«ÅÎÂ/FEEñ>2<y²sçÎÌÌÌîînyh9<³Í!#¶ÌÉj~¿¿¹¹ù¹o¹·oßÎyv²³³ÍvagÎY¿~=?2fffôiÆÊ*ËüY«««ÿý÷gggõ^Å2äääÑÑQMè4===Ë¬~=ô@ÓÓÓæõ@ÄÆÆ²ð#cß¾°²Ê2Ö4<äM&ôñåþýûÐ©ù(±eNVCp¹§ñìôôô¼õÖ[,üÐw¯×«÷+«,ó¼¥:tHõþÞßßÏ2ÞÞ^-pý#×©Þ^"¹ÌÉjÑÑÑÖtLLäYüø±ßïbáG@qqñåËÿç?ÿ·YeGà-¥±±QCCCúLÃ275êë¦M"¹ÌÉj)))ÓÓÓf]¦Y ÏÔýû÷wîÜ9>>ÎÂ¨ÿe·Ç8e5ß×2'«!655iB§E±@îîî­[·>|øÿ½ôeUUUæða>e¡jikâÖ­[¹FrÕ®^½ív»oÜ¸ÁyvÒÓÓ#'~ä³Ê2Ö=zT\¬1×ë½sçË<US-sj:Ë¬@V «U@V «UÈ* «UÈ* «U»téRTTÔ'Otºoß>ÍILLÌÉÉ	~¿_§_WW7ï[@TùMß<ß½·9;55¥³æDU`iooß»w¯"wöìY¾÷Þ:MHHèîî¶óäÉÖYÇ³qãÆ²9ºy[[®PXXhÍùà4çÑ£GÖM®^½ª9Ö~¬¶´´ðdXU4H½xñ¢"§ÄêôÂß|ûCåöÑªUÙÑÑQÓék¯½ffÚïÖÜ¼¦¦Æ1GõÕ*..Ö>Zs^ýuM?~GåµÈ*°âegg?>>>^S&<¨ÎiÄiâ3gÌ´ÛíÖÔÄU·ÒØTæää¡jss³æÜºuËº¹(áfÎãÇ5GeùdX]ÿ£¢>úè#365_&Îyï½÷ÔHSJûhÕºùóþþ~MôõõéÔü¼¶&6nÜh¿²é´îGÓ¦ÄGÑëÎÕu^¬«AGGÇôô´h¾@4Í9Z5g/_¾nÖå*À944¶ûvMØï_wþÆoõÆæ¶ÅÅÅBë~x!²¬«âjßRWÓEEE!³ªvöôôhÎýû÷Í×¢Ö«fõïÝ»ww>00 ÓK.YÃ¶F«ìò*dXÌ·¤oll4cGËø%°F¦:ÕÕÚçÌDrr²J9;;«;×´=Ì!G«ö¡0²¬`¿ùÍoLØtjFÃÃÃ:ííí5stQ\µµaªâ:îM)7	ÏÌÌ·Ñ*@VUkbbâáÃfÿ-Ì7¦ÍÍÍn·Ûq+³kii©UG#³³³C¼M0ZÈ*°½_FFFtÚÔÔdÍÞ¼y³Ji²ªKZ3qñâE3íïï×©Æ¬fM¯ªó¾M0ZÈ*°]HÍ8ÕîÚµË¬Vq=CÇ=zd©ZY5èêêZêhu```$dÀ166ÖÑÑa½ÿ~ww·u0³[jQQ,ÛÒÒ¢³Ê¡®o.ÐEÁY=ú´ÙúI×wV«««Í1%x9²¬-ë×¯onn¶Î^¸pÁq ~VsXDU!)@V «UÈ* «UÈ* «UÈ*dUÈ*dUÈ*d5éÿ<§?¨ÞIEND®B`


tGYEVeu|eõ/ókKñÇÍÆÏþíoÿåDVUYUYEVeUVeUVeYUYUdInêÔ)©åáUYUYe|I§Ó²¬Êª¬Ê*×G&só7=ÔüÜÒûUYUYUÆ¯­"«²*«²¬Êª¬Êª¬Ê*²*«²*«²*«Èª¬Êª¬"« «Èª¬Êª¬"«²*«²*«²¬Êª¬Ê*²¬Ê*²*«²*«È*²*«²*«Èª¬Êª¬Êª¬"«²*«²¬¬"«²*«²¬Êª¬Êª¬Ê*²*«²*««QèûP.]ºÄ#¬"«²:¾²Zºoïü×¿>àDVUY_YÍdfüÇ¿ßQr·ÍÊ<ùä2 ²¬ÊêøÊª×VUYUYEVeYEVeUVeYUYUYUdUVeUVUUdUVeUVUYUYUYEVeUVeUVeYUYUddYUYUdUVeUVeUVUYUYEVUYEVeUVeë"Äiù²ÿl~niÉÈª¬Êª¬Ê*ãÎë¯¿¾~Ìò555ÝíødUVeUVÂáûÈ:Èª¬"« «È*²*«È*²*«²*«È*²*«²*« «²*«È*È*²*«²*«È*²*«²*«È*²*«²*« «²*«Èª¬"«Èª¬Êª¬"«Èª¬Êª¬¬ÊêÕ'G]]¬Êª¬¬ÊêoÍê3g÷aqöìÙ³²*«²²*«WjÃáäxöÙgsí¼(¼ùæajÅ²*«²²*«wìØ±pf|ñÅÅO¿öÚkUYYÕ«300NpI:äuÖBVÃ²*«²²*«WúJGÍf³õõõ'~__Í7ñÚµkeUVedUV/©zþüù$®555UYYÕëð+K²*«²²*«×3«ÅÿhuHVÃíïü«¬Êª¬"«^VÏ;7þü]»v3cÚ´iaÐÔÔ|ö7]|eUVedUV/SÖ/^=	,«²²*«×¬Êª¬¬Êª¬"« «Ë¬¶¶¶Êª¬Ê*Èª¬þavïÞÎ°dÜÝÝÍfã8®­­íèè(>l)YUYEV Y'ÇÊ+ìY²dÉ~ùùóçëêêYÍårmmmaÐÒÒÒÐÐP|äð©½Ã,]ºTVeUVUJ2«á´øþûïÃ6g2ì©¬¬¼ÂyõÕW7mÚTÈjyyyòO]óùü2uáÂÔH-ZäÁUYEV)É¬:t(l·nÝ¶kÖ¬	;wíÚUVVþcgÎ¹ì-ôööÖ××'µ?ÙÇqa¶x<âÔÉayæW«²*«È*¥Õ°ýðÃwnØ°¡¿¿?:;;G¿åËïÝ»7ùªdOEÙt:]|ð(S^[UYEV)á¬ëÂäýjwª	Û-[¶$ï½¶öY8&GæbaOEEE>OéãâGUYUdÒ¾Z-ü¥;vôöö2ÆÛ·o¿Õ«WyóQú¢¶¹øQ¦dUVeY¥´³zôèÑ#G$EìêêêîîÞ»wïÙ³gLþc£_­ÏjøªL&EQ6-<Ì8%«²*«È*!«á"µªªê»ï¾'ÇþýûÃöñÇïééYzÑ®]»§Cný9YUUY½äåÕ~ø!ye4Ä5ÏÞ	nÈ[±Êª¬Ê*Èª¬^Rhj¨æóÏ?ÆÛ¶m§ÈÆûúúBeÃ9sæ,X°À/UYYÕ+Íêï½WüâèòåËýú"YUYYÕ´´´Óbúôéo¿ýö'|Æ«W¯N¦:;;Ã§ÍÍÍ²*«²²*«WäØ±c*þ;ø§O.|úÙgfÄ·:UYUUY-²*«²¬"«²*«²¬"«²*«²²*«²¬¬"«²*«²¬"«²*«²²*«²¬¬"«²*«²¬"«²*«²¬"«²*«²²*«²¬¬"«²*«²¬"«²*«²²*«²¬¬"«qV««ÿÔÑ¾±ä>[ÚÐó"«Èª¬#öì	?n½5;3fL¿ñÆôÝx¸Ûÿû ²¬Êêd±~ýúðÇ: «Èª¬"« «²*«È*È*²*«²²¬Ê*²¬Êª¬Ê*²²¬"« «Èª¬Ê*È*²*«È*Èª¬Ê*²²¬Êª¬¬"«²¬2áJ9v,¯¬Ê*²Èª¬"«²*«² «²¬²*«È* «²¬Èª¬Ê*¬Ê*²Èª¬"«²*«² «²¬²*«È* «²¬Èª¬Ê*¬Ê*²Èª¬"«²*«² «²¬²*«È* «²¬Èª¬Ê*¬Ê*²Èª¬"«²Ê¸ÒÞÞ3MMMVUYUYdUV@V@VeYUdUV@VeYUdUVUYYUdUVUYYUdUVUY@VedUVUY@VedUVUY@VeYUUY@VeYUUUY@VeYUUY@Vëìì¬««ãxîÜ¹ÝÝÝaOØf³Ù°§¶¶¶£££øàQ¦dY=öþýûÃ`ûöísæÌ	×ÖÖ---ÅúùçÿgÊ*4«ÅÊÊÊÂ¶¼¼|`` òù|eeeñC¦.P1Ì7Ü°hÑ"6:«]]]«V­8;Ç£OyYýÕéÓ§s¹Ü3gÂ8¢Âþt:]|Ø(S²¬þâÄ+V¬øñÇO+**òù|òLo9Ê¬ «öìyè¡úûû[[[Ã lÃ%lñÁ£LÉ*²:XYY*ö»Éd¢(Êf³¿ÞÅKOÉ*²êÏA «²²²*«Èª¬ «²²*«Èª¬ «²²²*«Èª¬ «²²*«Èª¬ «²¬Ê*Èª¬ «²¬Ê*Èª¬ «²¬Ê*²*« «²¬Ê*²*« «²¬Ê*²*«Èª¬¬Ê*²*«Èª¬¬¬Ê*²*«Èª¬¬Ê*²*«Èª¬¬¬Ê*²*«¿ÁGJ¥ñW_Édâ8¾óÎ;?ýôS#«²pN<y÷Ýw²ºlÙ²-[¶Á¦Mxâ	ë#«²p^|ñÅ6²:cÆS§NA6µ>²*«Wª§§çî	-d5ãÂlñYUËxôÑG?ÿüó0(d5¢Âl:¶D²*«W*õÏÂo¾¹¿¿ÿcK$«²p-M¹óæÍa¶Ë-³2²*«×ÕÝ»wÏ93¢L&óå_ZYUdUV@VeYU`ryÿý÷ëëë§LR]]½nÝº'OZYUkñÖ[oUUUµ··ñÁÃÏåææfË"«²pÕúúú¦MjZØsüøñ°çÛo¿µ8²*«W'uÁCv>õÔS|ðÅUY¸:áª´¦¦fÈÎGydçÎGVeàªUUUmÛ¶­ðé¦MväÈ+#«²pÕBGgÎÙÔÔôÎ;ï¬Y³fÆü±eUY¸FÇýõ×W®ÒK/ÊZYUUUY@VeYUUY@VeYUdUV@VeYUdUV@VeYUdUVUYYUdUVUYYUdUVUY@VedUVUY@Veddu4ÝÝÝÙl6ãÚÚÚY@V¯].kkkY@V¯]yyùÀÀ@äóùÊÊÊ0næ®»îzøá=ØÈêeÄqY`[n¹EVÕË¢¨0N§Ó@V¯]EEE>OcY@V¯]ccckkkm.UdõZ¤R¿ÜÉpï3LEÙl¶³³SVUYUUUY@VeYUUY@VeYUUUY@VeYUUY@VeYUYmnn¾é¦æPÊþüç?ßvÛmÖ	ì;î5kÖìÙ³-EI¹øY=räÈVJÜO>Y^^nÀÞxãT*µvíZKQÒÞ÷Ý~úig	`Ó¦MÕÕÕÖ	¬¯¯/dõë¯¿¶¬"« «È*²²¬¬"«²*« « «È*¥fÛ¶m>ø u`ûé§n¿ýöZYddd®]www6ã¸¶¶¶££Ã0ô÷÷WVV:Ûeþ¹­­-ZZZ,¥nß¾555©TÊÙ.«ð(//|>?âÿàCiY¼xñáÃGÌª³]VaÌÅq<âJûGíHYu¶Ë*¹(ãt:mAÀYu¶Ë*¹|>?xñi±0¶ Là¬:ÛeÆm.³ Là¬:ÛeÆüçÎ¾û2LEÙl¶³³Ó²0!³êlU@V@V@V@VYYYddddUU`ì¾±/úíÇ¯YdUVAVYYÆ(«½½½<ð@YYYÇóæÍëêê*>¦­­-sÑÖ­[oaÇ³gÏ_2wîÜÏ?ÿR*2gÎâcmß¾=Ú½÷©!1¶Â «0I³ZpìØ±°3NóÂ/qØq]]]2uÿý÷O¿øâ0Þ·o_744È*È*Èê/<¸nÝºúúúâýÉøÇã'NqÇÉTHï«Õ©S§Ê*È*Èêà-[Âøå_>|øðð¬?>C¸¬¬,Õ(dddõ×KÏÞÞÞáY¹ã5kÖñÂ©äåØÝ»w_ö:U5kVá¢sÊ)aúôéÁ~eéO>I¾dçÎC¦/^,« « «]]]µµµq/Y²äèÑ£aÿÚµkÿùØL6mÈ?°	ûkjj¢(SÏ?ÿü©S§dddddUUU@V@V@V@VYYYddddUþ+fÐ5ø¸-IEND®B`


T-TEST GROUPS=label(0 1)
  /MISSING=ANALYSIS
  /VARIABLES=年龄
  /CRITERIA=CI(.95).


T 檢定


附註	
已建立輸出	01-AUG-2019 16:40:15	
備註		
輸入	作用中資料集	数据集1	
	過濾器	<無>	
	粗細	<無>	
	分割檔案	<無>	
	工作資料檔案中的 N 列	82	
遺漏值處理	遺漏的定義	使用者定義的遺漏值會被視為遺漏。	
	已使用觀察值	每一個分析的統計資量是根據觀察值，該觀察值對於該分析中的任何變數沒有遺漏或超出範圍的資料	
語法	T-TEST GROUPS=label(0 1)
  /MISSING=ANALYSIS
  /VARIABLES=年龄
  /CRITERIA=CI(.95).	
資源	處理器時間	00:00:00.00	
	經歷時間	00:00:00.00	


群組統計資料	
	label	N	平均數	標準偏差	標準錯誤平均值	
年龄	.0	24	45.958	12.3270	2.5162	
	1.0	26	52.038	13.7825	2.7030	


獨立樣本檢定	
	Levene 的變異數相等測試	針對平均值是否相等的 t 測試	
	F	顯著性	T	df	顯著性 （雙尾）	
						
年龄	採用相等變異數	.021	.884	-1.639	48	.108	
	不採用相等變異數			-1.646	47.957	.106	

獨立樣本檢定	
	針對平均值是否相等的 t 測試	
	平均差異	標準誤差	95% 差異數的信賴區間	
			下限	上限	
年龄	採用相等變異數	-6.0801	3.7097	-13.5390	1.3787	
	不採用相等變異數	-6.0801	3.6929	-13.5054	1.3451	
