# Supplementary material for: High-order radiomics features based on T2 FLAIR MRI predict multiple glioma immunohistochemical features: A more precise and personalized gliomas management
Source: PLoS One. 2020 Jan 22;15(1):e0227703. doi: 10.1371/journal.pone.0227703 (PMC6975558; doi:10.1371/journal.pone.0227703)
Supplement: S3 File — (ZIP) [file pone.0227703.s021.zip › statistical analysis/ki67/spss sex.doc]

CROSSTABS
  /TABLES=性别 BY label
  /FORMAT=AVALUE TABLES
  /STATISTICS=CHISQ
  /CELLS=COUNT EXPECTED ROW
  /COUNT ROUND CELL
  /METHOD=EXACT TIMER(5).


交叉表


附註	
已建立輸出	01-AUG-2019 16:43:25	
備註		
輸入	作用中資料集	数据集1	
	過濾器	<無>	
	粗細	<無>	
	分割檔案	<無>	
	工作資料檔案中的 N 列	82	
遺漏值處理	遺漏的定義	使用者定義的遺漏值會被視為遺漏。	
	已使用觀察值	每一個表格的統計資料都以每一個表格中，所有變數指定範圍中具有有效資料的所有觀察值為基礎。	
語法	CROSSTABS
  /TABLES=性别 BY label
  /FORMAT=AVALUE TABLES
  /STATISTICS=CHISQ
  /CELLS=COUNT EXPECTED ROW
  /COUNT ROUND CELL
  /METHOD=EXACT TIMER(5).	
資源	處理器時間	00:00:00.02	
	經歷時間	00:00:00.13	
	要求的維度	2	
	可用的資料格	131029	
	精確統計資料的時間	0:00:00.00	


觀察值處理摘要	
	觀察值	
	有效	遺漏	總計	
	N	百分比	N	百分比	N	百分比	
性别 * label	50	61.0%	32	39.0%	82	100.0%	


性别*label 交叉列表	
	label	總計	
	.0	1.0		
性别	男	計數	12	17	29	
		預期計數	13.9	15.1	29.0	
		性别 內的 %	41.4%	58.6%	100.0%	
	女	計數	12	9	21	
		預期計數	10.1	10.9	21.0	
		性别 內的 %	57.1%	42.9%	100.0%	
總計	計數	24	26	50	
	預期計數	24.0	26.0	50.0	
	性别 內的 %	48.0%	52.0%	100.0%	


卡方測試	
	數值	df	漸近顯著性 （2 端）	精確顯著性（2 端）	精確顯著性（1 端）	
皮爾森 (Pearson) 卡方	1.213a	1	.271	.390	.208	
持續更正b	.663	1	.415			
概似比	1.216	1	.270	.390	.208	
費雪 (Fisher) 確切檢定				.390	.208	
有效觀察值個數	50					

a. 0 資料格 (0.0%) 預期計數小於 5。預期的計數下限為 10.08。	
b. 只針對 2x2 表格進行計算	
