# Supplementary material for: High-order radiomics features based on T2 FLAIR MRI predict multiple glioma immunohistochemical features: A more precise and personalized gliomas management
Source: PLoS One. 2020 Jan 22;15(1):e0227703. doi: 10.1371/journal.pone.0227703 (PMC6975558; doi:10.1371/journal.pone.0227703)
Supplement: S3 File — (ZIP) [file pone.0227703.s021.zip › statistical analysis/s-100/HLtest.doc]

GET DATA /TYPE=XLSX
  /FILE='C:\project\hebeishengerglioma\数据分析\T2 s-100\3.xlsx'
  /SHEET=name '3'
  /CELLRANGE=full
  /READNAMES=on
  /ASSUMEDSTRWIDTH=32767.
EXECUTE.
DATASET NAME 数据集5 WINDOW=FRONT.
LOGISTIC REGRESSION VARIABLES Label
  /METHOD=ENTER MinIntensity ClusterProminence_AllDirection_offset1_SD Correlation_angle90_offset1 GLCMEntropy_AllDirection_offset4_SD LongRunLowGreyLevelEmphasis_angle45_offset1
  /SAVE=PRED
  /PRINT=GOODFIT
  /CRITERIA=PIN(0.95) POUT(0.99) ITERATE(20) CUT(0.5).


羅吉斯迴歸


附註	
已建立輸出	16-MAY-2019 14:26:11	
備註		
輸入	作用中資料集	数据集5	
	過濾器	<無>	
	粗細	<無>	
	分割檔案	<無>	
	工作資料檔案中的 N 列	49	
遺漏值處理	遺漏的定義	將使用者定義的遺漏值視為遺漏	
語法	LOGISTIC REGRESSION VARIABLES Label
  /METHOD=ENTER MinIntensity ClusterProminence_AllDirection_offset1_SD Correlation_angle90_offset1 GLCMEntropy_AllDirection_offset4_SD LongRunLowGreyLevelEmphasis_angle45_offset1
  /SAVE=PRED
  /PRINT=GOODFIT
  /CRITERIA=PIN(0.95) POUT(0.99) ITERATE(20) CUT(0.5).	
資源	處理器時間	00:00:00.02	
	經歷時間	00:00:00.01	
已建立或修改變數	PRE_1	預測機率	


[数据集5] 


觀察值處理摘要	
未加權的觀察值a	N	百分比	
選取的觀察值	包含在分析中	49	100.0	
	遺漏觀察值	0	.0	
	總計	49	100.0	
未選取的觀察值	0	.0	
總計	49	100.0	

a. 如果加權有效，請參閱分類表以取得觀察值的總數。	


應變數編碼	
原始值	內部值	
0	0	
1	1	


區塊 0：開始區塊


分類表a,b	
	觀察值	預測值	
		Label	正確百分比	
		0	1		
步驟 0	Label	0	0	21	.0	
		1	0	28	100.0	
	整體百分比			57.1	

a. 常數包含在模型中。	
b. 分割值為 .500	


方程式中的變數	
	B	S.E.	Wald	df	顯著性	Exp(B)	
步驟 0	常數	.288	.289	.993	1	.319	1.333	


未在方程式中的變數	
	分數	df	顯著性	
步驟 0	變數	MinIntensity	9.456	1	.002	
		ClusterProminence_AllDirection_offset1_SD	9.322	1	.002	
		Correlation_angle90_offset1	5.085	1	.024	
		GLCMEntropy_AllDirection_offset4_SD	3.145	1	.076	
		LongRunLowGreyLevelEmphasis_angle45_offset1	3.398	1	.065	
	整體統計資料	24.077	5	.000	


區塊 1：方法 = 輸入


模型係數的 Omnibus 測試	
	卡方	df	顯著性	
步驟 1	步驟	32.762	5	.000	
	區塊	32.762	5	.000	
	模型	32.762	5	.000	


模型摘要	
步驟	-2 對數概似	Cox & Snell R 平方	Nagelkerke R 平方	
1	34.163a	.488	.655	

a. 估計在疊代號 8 處終止，因為參數估計的變更小於 .001。	


Hosmer 與 Lemeshow 測試	
步驟	卡方	df	顯著性	
1	2.489	7	.928	


適用於 Hosmer 與 Lemeshow 測試的列聯表格	
	Label = 0	Label = 1	總計	
	觀察值	期望	觀察值	期望		
步驟 1	1	5	4.896	0	.104	5	
	2	4	4.618	1	.382	5	
	3	4	3.747	1	1.253	5	
	4	4	3.227	1	1.773	5	
	5	2	2.442	3	2.558	5	
	6	1	1.259	5	4.741	6	
	7	1	.610	5	5.390	6	
	8	0	.173	5	4.827	5	
	9	0	.027	7	6.973	7	


分類表a	
	觀察值	預測值	
		Label	正確百分比	
		0	1		
步驟 1	Label	0	19	2	90.5	
		1	3	25	89.3	
	整體百分比			89.8	

a. 分割值為 .500	


方程式中的變數	
	B	S.E.	Wald	df	顯著性	
步驟 1a	MinIntensity	-2.151	.717	9.015	1	.003	
	ClusterProminence_AllDirection_offset1_SD	-2.088	.806	6.710	1	.010	
	Correlation_angle90_offset1	.432	.588	.540	1	.462	
	GLCMEntropy_AllDirection_offset4_SD	.196	.595	.109	1	.742	
	LongRunLowGreyLevelEmphasis_angle45_offset1	3.578	3.978	.809	1	.368	
	常數	1.715	1.322	1.682	1	.195	

方程式中的變數	
	Exp(B)	
步驟 1a	MinIntensity	.116	
	ClusterProminence_AllDirection_offset1_SD	.124	
	Correlation_angle90_offset1	1.540	
	GLCMEntropy_AllDirection_offset4_SD	1.217	
	LongRunLowGreyLevelEmphasis_angle45_offset1	35.807	
	常數	5.554	

a. 步驟 1 上輸入的變數：[%1:, 1:	
