# Supplementary material for: High-order radiomics features based on T2 FLAIR MRI predict multiple glioma immunohistochemical features: A more precise and personalized gliomas management
Source: PLoS One. 2020 Jan 22;15(1):e0227703. doi: 10.1371/journal.pone.0227703 (PMC6975558; doi:10.1371/journal.pone.0227703)
Supplement: S3 File — (ZIP) [file pone.0227703.s021.zip › statistical analysis/s-100/spss clinical Difference test.doc]

GET DATA /TYPE=XLSX
  /FILE='C:\project\hebeishengerglioma\数据分析\T2 s-100\T2FLAIR2018.12免疫组化数据 - 副本(1).xlsx'
  /SHEET=name 'Sheet1'
  /CELLRANGE=full
  /READNAMES=on
  /ASSUMEDSTRWIDTH=32767.
EXECUTE.
DATASET NAME 数据集3 WINDOW=FRONT.
DATASET ACTIVATE 数据集3.
DATASET CLOSE 数据集2.
EXAMINE VARIABLES=年龄 BY S100
  /PLOT BOXPLOT HISTOGRAM NPPLOT
  /COMPARE GROUPS
  /STATISTICS DESCRIPTIVES
  /CINTERVAL 95
  /MISSING LISTWISE
  /NOTOTAL.


探索


附註	
已建立輸出	15-MAY-2019 19:04:59	
備註		
輸入	作用中資料集	数据集3	
	過濾器	<無>	
	粗細	<無>	
	分割檔案	<無>	
	工作資料檔案中的 N 列	75	
遺漏值處理	遺漏的定義	應變數的使用者定義遺漏值視為遺漏。	
	已使用觀察值	統計資料是根據所使用任何應變數或係數沒有遺漏值的觀察值。	
語法	EXAMINE VARIABLES=年龄 BY S100
  /PLOT BOXPLOT HISTOGRAM NPPLOT
  /COMPARE GROUPS
  /STATISTICS DESCRIPTIVES
  /CINTERVAL 95
  /MISSING LISTWISE
  /NOTOTAL.	
資源	處理器時間	00:00:01.62	
	經歷時間	00:00:01.76	


S-100


觀察值處理摘要	
	S-100	觀察值	
		有效	遺漏	總計	
		N	百分比	N	百分比	N	百分比	
年龄	1.0	7	100.0%	0	0.0%	7	100.0%	
	2.0	36	100.0%	0	0.0%	36	100.0%	


描述性統計資料	
	S-100	統計資料	標準錯誤	
年龄	1.0	平均數	45.571	5.3446	
		95% 平均數的信賴區間	下限	32.494		
			上限	58.649		
		5% 修整的平均值	45.579		
		中位數	50.000		
		變異數	199.952		
		標準偏差	14.1405		
		最小值	27.0		
		最大值	64.0		
		範圍	37.0		
		內四分位距	28.0		
		偏斜度	-.432	.794	
		峰度	-1.144	1.587	
	2.0	平均數	50.056	1.9078	
		95% 平均數的信賴區間	下限	46.183		
			上限	53.929		
		5% 修整的平均值	50.006		
		中位數	50.000		
		變異數	131.025		
		標準偏差	11.4466		
		最小值	30.0		
		最大值	71.0		
		範圍	41.0		
		內四分位距	16.8		
		偏斜度	-.101	.393	
		峰度	-.837	.768	


常態檢定	
	S-100	Kolmogorov-Smirnova	Shapiro-Wilk	
		統計資料	df	顯著性	統計資料	df	顯著性	
年龄	1.0	.194	7	.200*	.905	7	.362	
	2.0	.095	36	.200*	.964	36	.282	

*. 這是 true 顯著的下限。	
a. Lilliefors 顯著更正	


年龄


直方圖


±MW¥k×®vµzðàÁä-ÒÏJ^gÔ××,R·oß>í+'ªªîûVûMMMî	«ÒÓÚäää|?¥~ªâý­­­áâÛo¿]|Õâª¡¡¡û®e«$¬JUI°*I°*IV%IÂª$IÂª$IX$	«$aU$aU$¬JUIUI°*IV%=qþùçåååéÅÖÖÖåËû¶HXwccc;wîJKK/^ÜØØxåÊüÞxã¼k×®g§»wïÎ´s^åÿ_Ûo9>ýàÁù·nÝêÎ°*=î¡³gÏÆvwwwlWVV&W¾òÊ+¹©=ï½÷^l;v¬¥¥%6öîÝ;ÓÎ9VüWä7[îïï?yòd{úôéøxêÔ©ë×¯ÇF]]]GGÇú©ÜËV¥ÇTYYY 4:::í0pÊ7/¹xûöí8>6Ö®];ÓÎ¹þ»-ú+òË-wvvÆZ6½µÞÞÞDâdÏ'.]ê^°*=¦V­ZUWW;w®àªÆÆÆÄ³Ô¼,ÝÄ³iwÎ±â¿"¿¹Ür«±Z5wzmyyù¼Ué¡jooÏýGõõõýýýÅÊÔ¶|çJKKgÚ®YÖ¬Ó^;í-ÏÎjOOÏàà`GGGÊj,yÝËV¥ÇWooo,éRüZZZÕùýë8VãªmÛ¶¥±qþüùäà;vÄÆ¢E^~ùew±UéqwöìÙÚÚÚ©àWSìAàG²ZÛÚÚM×¬YìÁªUéÛi||<*++É¼dQªô5DÓî|$«Õ¹Üò´¬^»víÖ­[ÉW^yÅ=+aUzLÕÔÔ=Sl'£üÆg¾y|ðAò(qþo¼L»óaXM/Îå§e5ÿ¦6lØà^°*=¦®_¿¾iÓ¦Å,]ºtçÎccc3WmÞ¼9³-ÚµkWrä´;	«s¹åi[mooOÕ½,aUÒ:wîêñ3gí[·nÅÅU«VùIX$	«$aU$aU$¬JUI°ú¤ô«_ýêûßÿþIÊV?üá!ÕÇÝ¶mÛ^~ùåAIÊV?ùÉOÿWAaõq°úúë¯ÿ»$e«^z	«XÅª$aUX$¬«XUa«U¬bU°*¬JVU¬JÂª°UIÂª°*IXV±*	«Â*V%	«XÅª$aUX$¬«XUaõ400°víÚÒÒÒÕ«WÏ4¼°*	«ÂêMÛÛÛcãüùóååå_õÕé¢^í5¬*«]¹råÿeº¿üå/îe¬bõ[(ø¬©©ùòË/sÓõê«¯ú¨LöÜs1Â+~øýLþyæEqvîe¬bõ±611±xñâø·wâÄ¸x·¨ýìgV«Êjë×ÿäØÿÞéÿÉä³ØÿÜs+ÜËXÅê·Pggç²eË<·*¬b«Âê£©´´«Â*V±*¬>x±ÑÓÓÓÐÐUa«X;Ú»wo[[[zÌ¾ûnß¾7nÜxï½÷o$nüäÉÉvSSÓäää@ÎÁããÙ³góOíý÷ßý/_nmm;wîlß¾½¿¿?=´»wï:t(9>ÃbV¿®¯¯oõêÕ±N­¯¯Æª°U¬&/:¹yóf²½uëÖd£««+ÿtÿ'|rôèÑÄ§æææÄÚ(¬K²ÿW^¡r4ßgèr¹¯Y:ÿÅ¤ù_±¤¤$=òðáÃ	qË6lN:¼¶&=àâÅqRq.qÌîÝ»O>kÚØ?ýôÓØ¤?ÞÑÑRÄ7mös©¨¨åøà'ôAàÂª°Õø pKKK,éR>ÈXÒÌñ1Ö ÁO±µ±?@iãÀ fÑÝñññy½¤´¶¶6$TUï´GÎrÕ±cÇ¯phh(¾;w~üñÇ¡ûØØX|ññ%íØ±#ùËð¬»»Ó¦MqUû=ÝØSp³ñÉÇø¾Å·ººººÅ|°±*a«O«aLLâ©ù«ÌXÈ&R6O5::zàÀVÆÆÆä1Ò`çÞ¾ûâSRHÎäéª«W¯Ö××§gmÜ¸1ùòâøtµÿEÆê3¤q@¬Ac¬~þùçÉOÉ#ÀéãÆôQ²ëòô1áo×àà`'ø`cUÂ*VhVU]ÖøØÐÐÒ0±½eËôù²dµk¾øxäÈÄÝÕÐ+>79 ÄÊ³,ÿªk×®­Y³&n*Ý_YY_Liiiì¿|ùrÁe¦½*àìííM/Æ¢ó­·Þz÷Ýwc&_U,gãìÒ5hr"Áê­[·âì¤ÉÄÁmñ£¾³NzÀ,'ø0cUÂ*VPVCü¥Ðâ¹1ÝµZrSÉÁ	lsauBÄä¡×bHnÜ¸QSS3ígå_¾ºººükÌÃ?>Õ±øÜcý°zòäÉäAàøÊcaüK|yév½iÓ¦ä'ûw.³àU	«X¢«É+@c!ÿàä;w¦ÒþÌey7ûÊ¯¬¬l¦/8½êÃ?Ìì:|á°v¼È9v^ºt)§±¬ËÕøúÓ8`§9s&Î4½6þ¢äÅÀóZ­>Ú±*a«O1«ããão¼ñFKKK,é>þøãcÇÝ¹s'öoÞ¼¹Õ^­ëRYY944,I~%rÚ«Ö¯_áÂôä%ÊÉ+ã«ýôÓOcÙÚÖÖÖßß¿Z-xÍpqñÓÃ#G_gúc.«ÕÙwÎ÷`¬JXÅêÓÁj¾I/¿ür,ïbûúõëáÙõ©Â°wßw^«ÕyQÔÛÛ[]]]ZZ_y²vL¯öªX¶?Ò577$¬ÆÆ-[ÌsþÌù¿ÉúÈYi«V±åÕj1B±ÕaúÛ®Ñ®]»Ü±cÇGtåÊoqJomm½zõjò¡~øa²T½víZñÅ¬Þºu+¾øøé!=£äßÛ¹ïÄÛA`UXÅ*V¿¹æõ[ªOK÷Û¬bUXÅ*V°*a«O«¹ÿéÃ¹<WüjÕü÷V±*¬buA³ZSSþæÜYMKß/PXÅª°U¬æZZZöìÙó`¬æ¿_ °Ua«Xýû´×&o4¯wi¸Wô~Â*VU¬bõïo@ÌwµZü~Â*VU¬bõïíÙ³§¥¥e^«Õ÷V±*¬b«_OËcccsü>ÓÞ/PXÅª°U¬þç´|òäÉy±:íû«XV±ºpYV±*a«XV%¬b«ÂªU¬bUXÅªUa«X°U¬«V±Ua«Â*V±*¬bUÂ*V±*¬JXÅ*VU	«XÅª°U	«Â*V±*a«XV%¬b«Â*VU¬bUXÅªU¬b«X°U¬«V±Ua«VU¬bUÂ*V±*¬JXÅ*VU¬«XÅª°U	«XÅ*V±*a«XV%¬b«Â*V%¬b«XÅªU¬bUX°U¬«XV±Ua«V±U¬bUÂ*V±*¬JXÅ*VU¬«XÅª°U	«XÅª°*a«XV±*¬b«Â*V%¬º±U¬JXÅ*VU	«XÅª°Ua«XV±*a«XV%¬b«ÂªU¬bUXÅªUa«X°U¬«V±Ua«Â*V±*¬bUÂ*V±*¬JXÅ*VU	«XÅª°U	«Â*V±*a«XV%¬b«Â*VU¬bUXÅªU¬bUX°U¬«V±Ua«VU¬bUÂ*V±*¬JXÅ*VU¬«XÅª°U	«XÅª°*a«XV%¬b«Â*V%¬«XÅªU¬bUX°U¬«XV±Ua«V±U¬bUÂ*V±*¬JXÅ*VU¬JXÅ*V±U	«XÅª°*a«XV±*¬b«Â*V%¬b«XÅªU¬bUX°U¬«X°Ua«V±UaUÂ*V±*¬bUXÅ*VU¬JXÅ*V±U	«XÅª°*a«XV±*¬b«Â*V%¬b«ÂªU¬bUXÅª°U¬«X°*¬b«V±UaUÂ*V±*¬bUXÅ*VÕ¯§§§¶¶¶´´´¦¦¦¯¯«Â*V±*¬>xçÏ'NTUUõÕWûzñÅ_íµ¸_|ñÅo3ÝgfzÂ*V±*¬¶hÑ¢/¿üò¿µdÉaõ¹ç*^xþ7ük&ÿü·u«s¹é	«XÅª°úõöönÛ¶íxØ´%¬XÕÂbõöíÛcccX5maÕøÄª°úPÝ¸q£©©ixxøz%°iKX5>±ªÂjWWWCCÃÈÈÈ,Ç`Õ´Uã«Âêª¨¨ÈåUÓVO¬«ßlX5maÕøÄª°UÓOã«XÅªiKX5>±*¬¶Uã«Â*VM[X5>±*¬bÕ´%¬XVM[ÂªñUa«¦-¬XV±jÚñ)¬b«¦-aÕøÄª°jÚVO¬«X5maÕøÄª°UÓ°j|bUX5m	«Æ'VUÓi«Æ'VU¬¶d|«XÅªiKX5>±*¬¶Uã«Â*VM[X5>±*¬bÕ´%¬XVM[ÂªñUaÕ´eÚÂªñUa«¦-Â*V±jÚVO¬«¦-aÕøÄª°UÓVO¬«X5mÉø4>±*¬¶Uã«z"X½uëÖèèhámårkÖ¬ÁªiKÆ§ñU¬Îÿ3s¹ôâäädìillÄªiKÆ§ñU¬Î»p´­­mÝºuq±³³3.`Õ´%ãÓøÄ*VpÁzéÒ¥øx÷îÝÜT¡lãT±ßyç¬¶d|«X¬ÃÃÃ7nÜ¡¡¡þþþdÿÍ7cÏÆ±jÚñ)¬bu¬&¦îÛ·/¹¸téÒdãøñã6mÉøV±:×Î?ßÓÓÓÛÛ»råÊTÙä©VÏ­¶d|XÅê¼ªCCCññúõëqq||<¶/_UÓOã«XG]]]¥¥¥ë×¯ÏßÙÑÑQRRòÖ[o`Õ´%ãÓøÄ*Vç·T­««+Ø³cÇáááØ¸|ù2VM[2>ÝËXÅê<Xa^=.ÅªiKÆ§«X«Évòë4mmmÉÅë×¯ÇÅ]»vaÕ´%ãSXÅêüV«===ÉûBä_úôéä"nÝºUÓOa«÷/V¥UUUöìöÚ3gÎXUÓOa«ßfX5maÕøÄª«¹>H/&¯îééÁªiKÆ§ñU¬Îµ+W&o¦ßÜÜ|ôèÑ²²²ØßÞÞúúú°jÚñi|b«sjdddß¾Áç'âã#GöîÝ»dÉø¸uëV¶d|XÅê<:sæL:::¼¸££cÅÉcÂ«V­ê*¸M«¦-Â*Vg¬««+È<vìX²ZöÙg7nÜ866?ÿüó8`rr29ÀjÕ´%ãSXÅêV«É3©ÁjeeåÚµk[[[cOr@þß«X5mÉøV±:cÝÝÝÉû<¤×ÖÖnÙ²¥··7yóåËWTTxnÕ´%ãÓ½U¬ÎÕæææøxôèÑäAàô±ßäßÿ¬¶d|º±Õû466¶ÿþ½÷&ïMKÕøøÞïs¹Ü+W±jÚñé^Æ*VïßøøxòJ¥Ø¾zõj zèÐ¡ü§]á¯Ù`Õ´Uã«Ê8«É¯Ö$ÿeM|Ü¾zÕ|8UÓOã«XG	®;wîhcOò»ªÉúÕÿ·jÚñi|b«s-yõwÞí%Kttt¼ðÂo¾ùfríÅq±jÚñ)¬buÆîÜ¹³zõêTÐÿñwvvæ >ÚjÕ´%ãSXÅêVM[X5>±*¬bÕ´%ãÓøÄ*V±jÚVO¬«¦-aÕøÄª°UÓVO¬«X5m	«Æ'VUÓ°j|bUXÅªi«Æ'VU¬¶d|«XÅªiKX5>±*¬¶Uã«Â*VM[X5>±*¬bÕ´%¬XVM[ÂªñUaÕ´eÚÂªñUa«¦-Â*V±jÚVO¬«¦-aÕøÄª°UÓVO¬«X5m	«Æ'VUÓ°j|bUX5m¶°j|bUXÅªiKÆ§°U¬¶Uã«ÂªiKX5>±*¬bÕ´Uã«Â*VM[2>O¬«¦-aÕøÄª°jÚ2maÕøÄª°UÓOa«X5m	«Æ'VUÓ°j|bUXÅªi«Æ'VU¬¶d|XVM[ÂªñUaÕ´eÚÂªñUa«¦-Â*V±jÚVO¬«¦-aÕøÄª°UÓVO¬«X5mÉø4>±U¬¶Uã«ÂªiË´Uã«Â*VM[2>±U¬bÕ´%¬XVM[ÂªñUa«¦-¬XV±jÚñi|b«X5m	«Æ'VUÓ°j|bUXÅªi«Æ'VU¬¶Uã«ÂªiKX5>±ª¬³:22RQQUÓVO¬«[©êêêUÓVO¬«[x988²:99ù?zá^í5ÓÖÂ¶þô§?ýÏL÷/ÿ²*Ãã³ýÿ|ðÝï.Îö=ø?ü«zâ[Ígõ¿õüóÏcuÁ²úÛßþ6Ç3«/~&ÃãóÄïödûî³Y±ª'UcµÕþ5«w_üYþýÎ6«ÿ¼tqï¾«Â*V±U¬bUXÅ*V±U¬«XÅ*V±U¬bõ	«XÅ*V±*¬b«XÅ*V±U¬b«XÅ*VU¬b«XÅª°U¬b«XV±U¬b«XV±U¬b«Â*V±U¬bUXÅ*V±U¬b«XÅ*V±U¬«XÅ*V±Ua«XÅ*V±*¬b«XÅ*V±*¬b«XÅ*VU¬b«XÅª°U¬b«XÅ*V±U¬b«XV±U¬b«Â*V±U¬bUXÅ*V±U¬bUXÅ*V±U¬«XÅ*V±Ua«XÅ*V±U¬b«XÅ*V±*¬b«XÅ*VU¬b«XÅª°U¬b«XÅª°U¬b«XV±U¬b«Â*V±U¬b«XÅ*V±U¬bUXÅ*V±U¬«XÅ*V±Ua«XÅ*V±Ua«XÅ*V±*¬b«XÅ*VU¬b«XÅ*V±U¬b«XÅª°U¬b«XV±U¬b«Â*V±U¬b«XÅ*V±U¬bUXÅ*V±U¬«XÅ*V±U¬b«XÅ*V±Ua«XÅ*V±*¬b«XÅ*VU¬b«XÅ*V±U¬b«XÅª°U¬b«XV±U¬b«Â*V±U¬b«Â*V±U¬bUXÅ*V±U¬«XÅ*V±U¬b«XÅ*V±Ua«XÅ*V±*¬b«XÅ*VU¬b«XÅ*VU¬b«XÅª°U¬b«XV±U¬b«XÅ*V±U¬b«Â*V±U¬bUXÅ*V±U¬«XÅ*V±U¬«XÅ*V±Ua«XÅ*V±*¬b«XÅ*V±U¬b«XÅ*VU¬b«XÅª°U¬b«XV±U¬b«XV±U¬b«Â*V±U¬bUXÅ*V±U¬b«XÅ*V±U¬«XÅ*V±Ua«XÅ*V±*¬b«XÅ*V±*¬b«XÅ*VU¬b«XÅª°U¬b«XÅ*V±U¬b«XV±U¬b«Â*V±U¬bUXÅ*V±U¬b«XÅ*V±U¬«XÅ*V±Ua«XÅ*V±U¬b«XÅ*V±*¬b«XÅ*VU¬b«XÅª°U¬b«XÅ*V±U¬b«XV±U¬b«Â*V±U¬b«XÅ*V±U¬bUXÅ*V±U¬«XÅ*V±Ua«XÅ*V±U¬b«XÅ*V±*¬b«XÅ*VU¬b«XÅª°U¬b«XÅª°U¬b«XV±U¬b«Â*V±U¬b«XÅ*V±U¬bUXÅ*V±U¬«XÅ*V±UaõÔ××·bÅÒÒÒÕ«W=«XÅ*V±*¬>xÇæææ·ß~«XÅ*V±*¬>xååå±1>>^QQñå_Võ½ïï;ßùNÕKág)É+.]Ëåª²Û²eËâ³z÷Åú§ÿáñ¹ä»Ïfûî³[ºtéïÙ°ÕG¿ýÕW_ý¯¢Þ÷ÝÃyÐyæººº­-Öññû×¿þõöâ/þà?ØÝJJJ~üãgõìêëëc|nÙ²%«'øüóÏ<¼÷»ßýõ¯Ve1§¤ÛeeeßÄ_±|ùòßüæ7Y½óº»»cÚÊð¿Ìÿüç¯¾újÿù=ûì³Éó ì÷¿ÿÏ¿ýíoY=ÁM6mÜ¸"zX]¶lÙøøxò plc«XÅ*VÕ---±±U¬b«ÂêwîÜ¹`¯¤¤dÅ===XÅ*V±Uaõ«XÅ*V±*¬>²Ö­[÷Ùgeõìþüç?ÿèG?Íê	¾ÿþû?ýéO3<>kjj¾øâ¬ÝÿøÇÉë'2Ù/ùË_üâÑÂbU$¬JUI°úT×ÓÓS[[[ZZZSSÓ××woÖwðËû?i¬]»6ù·:ËØ	&uvvær¹ûÅSwÃÃÃ¹¼²w÷Ý½·©©©¬¬lÕªU]]]Ù;Áü»/yLþVÿ³ÊÊÊóçÏÇÆ'ªªªîÍúþsysÿ'­ø÷ÙÞÞqåååÙ;Áhbb"~6JYÍÒ	¶µµ:s<§ñîûðÃ÷ïß?99¦¾ðÂIñÇáV§iÑ¢E÷ÞÁ?ÿY®zò;út¬È3yûöí;xð`ÊjN0LM~*Ë)<w_üØwéÒ¥`ÒÕ«Wëëë3|Âê4õöönÛ¶í^Ñ;øç3ËUOøbnñâ¿ÿg5±"ÏÞ	^»vmÍ51¥¬fé+++âKs¼|ùröî¾ø::?ÑÆRµ¿¿?ÿ£6Ä½ñ)¬ÎØíÛ·ÇÆÆîÍúþáÍý¿¹:;;wNÎØ	ÆÕÝÝoêI¬ûÅÓÞ¸q#y°!cg_óÑ£Gc#~h2y÷ÖÕÕÍå,êFXý	«©©ixx8¹8Ë;ø?7÷ÿ¦WÙ;ÁÜ?á0g3vvù_g&Çç½©çcE>³xÚgaõïuuu544¤fyÿÇðæþ¼ÊÊÊS¯y3ÍÞ	æû½0î¾¡¡¡ä¿LÞ;vìhmm½7õõuëÖer|®_¿þÂYaÕÂ***Ö:Ó¾ÿ,W=áõõõ­^½:ÖõõõÉ<c'XÌjN°···ºº:î¾^z)dÍÞÝ7::ºaÃäÉãÁÁÁLÏ²²²äHÙÂª$IX$	«$	«$aU$¬J$¬JUI°*IV%IV%IÂª$IXø±ËÕÖÖÎëøwÞy§`Ïo¼á;)	«Zè7oöÚ;wîðyùòåøØÙÙî=¾°ªÞG(nÝºµqª¶©CÅUMMMù¬ÄÇ#GÄÇÝ»wÇÎE½ôÒK!´ï§$¬jávõêÕÐñÌ3ùp8p`Æ¹Ü¥KâãÉ'ówÍ###±á¿ÒU-Ð&''ÂXÀ²ÿ?Tßºu+Ö¯qí§~|<|øpBr|<uêTGúÞJÂªÞÏåV¬X±fÍôßäåK±¹gÏôø»wï&­­­×®]Ko$¶O8ãããÛ·o/XþJVµ ªÕÕÕÓ®V¯ÓÛÛÛ×××ÝÝ¾¦)v;w.Y×úöJÂªîu¬Æ"uåÊ/^cÎ?ßzë­K.½9UGGGò°ppë»*	«Z¸¬æÿÒj«±¢MaG¯_¿*pO(Z°ªÒÝ»wëêêb.Y²$66oÞý§J.FÉ³ªaj¨¹sçÎØ>zôhìß¿ÿÍ7CÙØSUUµnÝ:ßXIXÕÂuÚÅëL«ÇÏ?rÃÉþeË566úJÂª4WVãÚ¥K~òÉ'ííí±½ûöäª¸¸cÇß@IXæÄêÕ«WÒ·oßN/:u*¸X°*ÍjKKï$¬JUI°*I°*IV%IÂª$IÂª$IX$	«$aU$aU$¬JUI°*I°*IV%IÂª$Iº_ÿû9ô±Ù;·IEND®B`


GqòkUUÕSµßÒÒâ"aUzQêEO;¿­­-\»vmî¢Ë/Ç¢§a«$¬JUI°*I°*IV%IÂª$IÂª$IX$	«$aU$aU$¬JUIUI°*IV%=wôÑGeeeé³mmm.ô°HXu###Û¶mçÎÛÜÜ|ýúõÌV®J¥Ò+oß¾ÎD1qÿþýÇÍÌ§ÞÞÞÆÆÆÒÒÒ6ÄõäÞ·<¯9îá2ÏnÜ¸Ñ7WÂªô¬kii	Î;Ó.éÊÊÊdQÿo¼(³k×®>vìØÑ£Gcb÷îÝO555êµk×._¾Ü¼ysÖùØ|òäÉXzæÌ8=úô­[·b¢¡¡¡££cÙD¾ËV¥gTÀO:2YMÎÞ»w/ÖúúúÇÍò/p*ÃÖ¬ùeÓWÒÝÝHÌ9qâÄüùó%¬JÏ¨ÅCÕÕÕ/^ÌZÔÜÜxf5KOÇDâÙ¤3§Ô¥Kâ¹¯æsÍY¬Æh5ÆÜé¥eeeÓc^V¥éÔÞÞú[Ë/ïííÍG¦m+**Êt®¸¸øq3S5é©¬¬¥ÎZ4é5?Õ®®®þþþ4«1äõ]°*=»º»»cHÆïèÑ£ÕükjjK­^½:wQ¬&¯Ë¥1ßdå­[·ÆDiié+|%¬JÏºsçÎÕÖÖæîÞNà<G«­­­1¿¼¼üÎ;¹K§±øÔ©S¦uuuÉ¬JXf¦ÑÑÑp¨¤¤äq¬&Ú¥ßC4éÌ|ºqãFÜV »çù	7«7oÞ¼÷n2ç7Þð°*=£^yå°'péd?jÖ'>3YÝ³gO²8ó/ÎÌ§Í7Çú'NÈþeþÛ-æsÍ²yUMMM¾ËV¥gÔ­[·Ö­[7wîÜ¢¢¢ùóçoÛ¶-ë°¬Æ¢õë×Ç³´´tûöíÉÎÌ§²²²I÷§§ó¹æI_[nooOÕwYÂª¤¼ºxñâùóçÓ=6¾÷n]¼x±HÂª$IX$	«$	«$aU$¬JÕç·_ÿú×ßøÆ7Ê%éEîå_îîîVg¾Í7¯X±¢_^ä^õÕÜÿ*(¬Î«o¿ýöÿ¤¹×_«XÅª$aUX$¬«XUa«U¬bU°*¬JVU¬JÂª°UIÂª°*IXV±*	«Â*V%	«XÅª$aUX$¬«XUaõójhh¨¢¢"¶§§§¼¼¼¸¸¸¦¦æÜ¹sXUa5ßâGªºº:úÿ÷¡¹¹ùØ±c1ÑÚÚºvíÚÉé­·ÞÂª$¬«Ùýýý¬ÇÄèèhb<x¬7ß|Óï¤$¬«ÝV³¦ïçôóÿÜhUVÕ§³ZTT.))ñÚª¤dµ¯¯ïüß×ÖÖöî»ï¦WØ¶m[2±sçÎ»wïf]¼µµõöíÛ1qúôéë×¯?ÒÄÊÜ¢ýû÷ß¹s'9sæòåË1qàÀ7ofnfÒ­[·?L<x0ÖÁêX]°`Áèèh²8¦±*©YKb¢£££³³3¸ÿ~«ãããë×¯OxÂ­dé;ª¹k×®@+&Þÿý<3_qKl7o^qqqUUULg®»hxx¸©©)E/Û91±fÍ£GÆ:»wï>ölØùÑG¥¯çÄ±ÎªU«âtïÞ½q$¿ÍLÆÙÌGãqÝ¸qcáÂOYHéQæÌÇ$sø÷°ºnÝºxèc"N±*©0w:u*°IX½råJ¡`/½zõ#GbfÍ¿ööö±süaÇKûÔâv·oß9§±±1îFLÄ9n÷Éân$¾ÆdI³äîÅlTâR9###|ðAOOOÌ	kãRï¼óÎ¹sçÒ~'ï¶¶lÙ2%YbìÛÐÐéKî,A>¹4wN2MxX²øS"þ(//ïêêÂª¤BcõúõëÉÈ,iâÉ=³öì!lÂL(Ãµ¸xgÓêìÛ·/yÞçë	¨ru³¤éZZZâVWWç1gÎ9ÉÆéÜ¹s3WÎ]´`Á¸qñ05OV/_÷*¶%=ZäÒ×*'÷üÒ¥K1Ôû¤?lE<ó'1òNþxÂV$Ç½Ê;'KÐLrçDñhÇãóìjX4YMÏ2wgF7nLã´¿¿ÿêÕ«1âÌæYeeewwwLÜ¾;dÍzËKæ»J']sâ¸víZ]]]²(æqµ~øaBcÈÜÉ(fÆÙdßuòG@Æ 0ÜiSãqH³Å¦å9þÎE4wNZÐôÒÜ9Éùx@r_ÉÆª$½;Êê;w§øà*	«£££###XÓ­f¼o4­æ.9YÐ¯!tyóæÍà3McÜÉd4CÀdlk&ï­t4ù×Fþ[«©<J»Y»Ç±*I/<«·nÝÑgKKKJC©dÏpÌß´iS²B>¬>y´:00V$¯t~ÉÌcd-Z»vm²G·¯¯oéÒ¥1Ø Åh/+û£êDÐtÆÌØÌä½ÁûöíKîó±cÇnÜ¸ÜÊâ:Ó·¶¶æÿRq>¬æ¹~ò*¬JÒ¬b5Æ|éK<y2Ù9ùÚjÕéí±ouuu4ãî%Òioo3gNè2þüôK	6¹W¬X¯««;3×ñw@"nÜ¸ÏñÁððpæNï@+wh<wuu¥_Qþ,ïÝÉçX6=4Oj¬JÒÊjXÕÓÓóäubeË%/F÷îÝ;|øpòdx·cÇáj3ø¼#Ñäó¦Éë¦Éh8K©seÎQi=NMoQò)X[Åª$MUe½ë«UaUf)«©T*ëUÌ|êëë«¯¯Oþ±frÍ¹Ç<V±*©Y­ªªJv~æÏjhÚÞÞ.]*++ôc	«XTp¬îÚµ+ykO«Oý°f`#ÝäHóHXÅª¤cuxx8,¼÷nþ£ÕäÃ6É'^cëOzÌ#a«ÕGGuß±cÇTG«É¬é$¥gfwPXÅª¤Âbu||¼ªª*ÿÑjeeeúhâ?Æ,Z´èÑdÇ<V±*©@Y4ñMógµ§§§¦¦&F¥õõõÉ?Ü1U¬J* VU¬JVUIÂª°UIXV±*IXÅ*V%	«Âª$aUXÅª$¬«X$¬b«UaU°*¬bU°U¬JVUIÂª°UIXV±ªç¤?þø¿Î|òo°U¬jö¶¥R©­ÿeÕ³ÿÛýíoë[ ¬b«U¬pWÿ×ágÿµ¦é?aUXÅ*VU¬«ÂªUaUXÅª°*¬B«XV±*¬«Â*VUa«Âª°*¬bUX°U¬«XVU	«Âª°UaUXV±*¬bUXVU¬«Â*VUaUXÅª°*a«XV±*¬«VUa«Âª°*¬bUXÅª°*¬«XVU¬«Âª°UaUÂ*V±*¬bUXV%¬«Â*VUaUXÅª°UaUXV±*¬«XVUa«ÂªU¬bUXÅª°*¬JXVU¬«Âª°úùuþüùêêêâââÇýÀaUXVÕ¼*++»yófLÄiEEELçUaUXVójÉ%·oß8é¤&ëÍ7ßô;)¬«ÂêSêéé)--8ã´»»ûáÃí9½ñÆF«Âª°*¬>½úúú«W¯&¾644xmUXVÕéW\<é4VUaUXr1Bíëë+W®ÄÈ«Âª°*¬N¿ë×¯¦1NÓÆª°*¬«_lXVUa«ÂªU¬bUXÅª°*¬«XVU¬«Âª°Ua«Âª°*¬bUXV±*¬«Â*VU	«XÅª°UaUXV±*¬«XVUa«Â*VU¬bUXÅª°*¬JXVU¬«V±Ua«Âª°*¬bUXV±*¬«Â*VU¬«XÅª°UaUX°*¬«XVU¬b«Â*VUaUXÅª°*¬bUXVU¬«V±Ua«Âª°*aUXV±*¬~®òÉ'ÏîÜñUaUX=¬~óÿ0wÎ«5Ïþ+JýñôUaUX=¬.[öê±ÿ±cF¶ºâåo`«Âª°U¬bUXÅª°U¬«XV±*¬b«Â*V±UaUXÅ*V±*¬bUXÅ*VU¬«XV±Ua«XÅª°*a«XV±*¬bUXÅ*VU¬b«Âª°U¬bUXÅª°U¬«XV±*¬b«Â*V±UaUÂ*V±*¬bUXÅ*V±U¬«XÅ*VUa«XÅª°Ua«XV±*¬bUXÅ*VU¬b«ÂªU¬bUXÅª°U¬«XV±U¬«Â*V±Ua«Â*V±*¬bUXÅª°U¬«XÅ*VU	«XÅª°Ua«XV¿îß¿ßÒÒRRR²xñâóçÏcUXÅ*VÕé·wïÞýû÷©K,Áª°U¬«Ó¯¦¦æêÕ«é³áërª¨¨xë­·üN«XÅª°ú<XZZCÕÞÞÞ`uSNßùÎw°*¬b«ÂêÓ+**:räHLv­®®ÎN`a«XV§ß2G®XV±UauúmÝºµ­­-&úúú.]Ua«XV§ßððpSSSSëêêúûû±*¬b«ÂêVU¬bUXÅª°Ua«XV±U¬«Â*V±Ua«Â*V±ªÂcõîÝ»ÃÃÃÙ×J=îXV±*¬bõL¥ÓgÇÇÇcNss3VU¬«XrCCCáè©S§ã6tvvÆÙ¾¾>¬«XV±:ÍëÕ«Wãôþýû©BÙæb"ÎnÚ´	«Â*VU¬NAÖÁÁÁÛ·oÇÄÀÀ@ooo2ÿÎ;1gÍ5XV±*¬bu¬&¦îÛ·/9;þüdâøñãv«XV±o.]êêêêîî^´hQZÙÇ"«Â*VU¬>e¨:00§·nÝ³£££1½páB¬«XV±:Î?_\¼lÙ²ÌEEE«W¯Âª°Ua«Sª644dÍÙºuëàà`Lv«Â*VU¬NÕø±Híëë9/_NûUa«Â*V§Àjeee2|æÔ©SÉÙ[·nÅÙíÛ·cUXÅª°Õ©V»ººãBd.=sæLr»wïbUXÅª°Õ§£Òªªª;wNºôìÙ³YÖbUXÅª°Õç+¬«XÅªf'«©TjÏ=é³É»ºº°*¬bUXÅj¾õõõ-Z´(9~kkë#GJJJb~ÌéééÁª°Ua«y544´oß¾àóÄqzøðáÝ»wÏ7ïèÑ£qºqãF;U¬«XBgÏP7wtt'û/^Ü1Qpþ+VU¬«XlçÏ2;VçÌ³fÍ8ûÑGÅãããÉF«Â*VU¬æ5ZM^IV+++ëëëÛÚÚbN²Booo,JÿïU¬«XV±úØ.ç!½¸¶¶vÃÝÝÝÉÁ.áµUa«Â*Vóeµµµ5N9ìNïûMdøË_þ«Â*VU¬>¥ýû÷ïÞ½;96aUãt×®]ÿvu©Ôõë×]ÄXV±*¬bõé&ïTé7n¢Ì|ÙõýVU¬bU³Õä£5É¿¬Ó-[¶¤íÙ³'æ¼÷ÞXÍêOúS<2ßüfù³ÿ*)ùòk¯-Ã*V±U=§¬&%¸nÛ¶- 9ÉgUñ«ÿ·U<ã|å+¥ç>Þÿì¿ö¿»±¾þ?`«XÅª_VP7mÚÓóæÍëèèX²dÉªU«¥W®ÄÄjÕxÞç»xg[¬b«XÕóÈê§~ZSSôÑßþygggæjê3gV±U¬«X®Ã*V±U¬«XÅ*V±*¬b«XÅ*V±Ua«XÅ*V±*¬b«XÅ*VU¬b«XÅ*VU¬b«XÅª°U¬b«XV±U¬bUXÅ*V±U¬b«Â*V±U¬bUXÅ*V±U¬«XÅ*V±*¬b«XÅ*V±Ua«XÅ*V±*¬b«XÅª°U¬b«XÅ*VU¬b«XÅª°U¬b«XV±U¬bUXÅ*V±U¬b«Â*V±U¬bUXÅ*V±U¬b«XÅ*V±U¬«XÅ*V±Ua«XÅ*V±*¬b«XÅª°U¬b«XÅ*VU¬b«XÅª°U¬b«XV¿è:;;S©V±U¬bUXý¬ÕÖÖ&¬>|øð¿çôýï«/«×¯_ÿxZ¿~=V±UaõÑ¾û8°úàÁ9¾õÖ[X!X]â[ùjCÍ³ÿÛÅ*V±ªBgõæÍuuuãããvÏVÃÙêÿX_U¬bUÎjSSÓþßÝÂ*V±U¬«õÞüXÅ*V±UaõóñÕh«XÅ*VU¬b«XV±úâU¬b«XV±U¬bUXÅ*V±U¬b«Â*V±U¬bUXÅ*V±U¬«XÅ*V±U¬«XÅ*V±Ua«XÅ*V±*¬b«XÅª°U¬b«XÅ*VU¬b«XÅª°U¬b«XV±U¬bUXÅ*V±U¬b«Â*V±U¬bUXÅ*V±Ua«XÅ*V±U¬«XÅ*V±Ua«XÅ*V±*¬b«XÅª°U¬b«XÅ*VU¬b«XÅª°U¬b«XÅ*V±U¬b«XV±U¬b«Â*V±U¬bUXÅ*V±Ua«XÅ*V±U¬«XÅ*V±Uau&X]µjå¿üËOý×¼òë_ÿ*V±ÕÏ½ú¥^³¦i÷îÅªÕT*µÿÝÏþkÃúÆù_	«XÅêçÞLýR7ýó÷+¿½«ÂjjFtNünç?ÎU¬bõ`õEüÍÂ*V±U¬b«XV±U¬b«Â*V±U¬bUXÅ*V±U¬b«XÅ*V±U¬«XÅ*V±Ua«XÅ*V±U¬b«XÅ*V±*¬b«XÅ*VU¬b«XÅª°U¬b«XÅ*V±U¬b«XV±U¬b«Â*V±U¬bUXÅ*V±U¬bUXÅ*V±U¬«XÅ*V±Ua«XÅ*V±U¬b«XÅ*V±*¬b«XÅ*VU¬b«XÅª°U¬b«XÅª°U¬b«XV±U¬b«Â*V±U¬b«XÅ*V±U¬bUXÅ*V±U¬«XÅ*V±Ua«XÅ*V±Ua«XÅ*V±*¬b«XÅ*VÕ¼ëêêª­­-..~åWzzz°U¬b«Âêô«¬¬¼téRL8q¢ªª*&þwN?ûÙÏ°U¬b«ÂêÔ*--ðàAj²Þ|óM¬bõ¹eµâå¯§f¨¹s¿2S¬¦f4¬«O©»»óæÍ1ñ?sZ³fÑ*VgV+¿ýòþw7ÎÈM/üÆ?Î«/½Tzîãý3%:VÕ'uïÞ½æææ¯­b«XÅª°úºûvKKËàà wc«XÅª°ú:þ|ccãÐÐÐÖÁ*V±U¬«yUQQù6¬b«XÅª°úÅU¬b«XV±U¬b«XÅ*V±U¬b«Â*V±U¬bUXÅ*V±U¬«XÅ*V±U¬«XÅ*V±Ua«XÅ*V±*¬b«XÅ*V±U¬b«XÅ*VU¬b«XÅª°U¬b«XV±U¬b«XÅ*V±U¬b«Â*V±U¬bUXÅ*V±U¬b«XÅ*V±U¬«XÅ*V±Ua«XÅ*V±*¬b«XÅ*V±U¬b«XÅ*VU¬b«XÅª°U¬b«XÅ*V±U¬b«XV±U¬b«Â*V±U¬bUXÅ*V±U¬b«XÅ*V±U¬«XÅ*V±Ua«Ïäëÿ¶åÛß^òÛhãÆÿüOË°U¬b«X=¬þëÎµ±Õ1j|ö__þrñÊ·¿U¬b«XÅê¬bµæß/þÉ~íÇ?jÀ*V±U¬b«XÅ*V±*¬b«XÅ*VU¬b«XÅª°U¬b«XÅª°U¬b«XV±U¬b«Â*V±U¬b«XÅ*V±U¬bUXÅ*V±U¬«XÅ*V±Ua«XÅ*V±Ua«XÅ*V±*¬b«XÅ*VU¬b«XÅ*V±U¬b«XÅª°U¬b«XV±U¬b«Â*V±U¬b«Â*V±U¬bUXÅ*V±U¬«XÅ*V±U¬b«XÅ*V±Ua«XÅ*V±*¬b«XÅ*VU¬b«XÅ*V±U¬b«XÅª°U¬b«XV±U¬b«XÅ*V±U¬b«Â*V±U¬bUXÅ*V±U¬«¡òòòâââsçÎa«XÅ*VÕé×ÜÜ|ìØ±hmm]»v-V±U¬bUX~eeeããã11::ZQQñàÁª¾úÕ¯¾ôÒKUÓ-~ã©çÙÍû93uÓ%%Å3uÓ_úÒ¿©NM4C[ý¥¢¢/ÍÔþ¯ÌÔ>ÿ«sgê¦gävã¡þrqñ´ây«XýÂ+..Î~øðá¯rzçw:txºmü-X° ººzz]¿~ý+6ÎD1îã7¦wÙ6ÄsÖøÃé]|Í5+W®­^=Ñô.Wlu|Ë¦wñýèG±á3²ÕqÏãÛ=½Ë~÷»ßýÚ×¾6íþÁ~0íGì3öÚk¯Mû²ñ§|MMÍ´/¾ûöi?ýîw¿ûóÿ!¬~±¥§KJJ·+~õúù`~ÿûßÔVÿá­þË_þRP[½÷îúúúBüÞ÷¾÷_üµ¬Æp0ÇgóÆ*V±U¬«ÓoÝºuG8mnnÆ*V±U¬«ÓïâÅ.,***//ïêêÂ*V±U¬«³6¬b«X°ú¹õÓþôW¿úUAmò_ÿú×oë[çÏ/¨­¾téRlõÈÈHAmõo~ó¦¦¦Bû¥þÉO~ràÀOnÂª$IX$	«$	«S®¯¯¯¾¾>9Ör'ý?ðÕÙÙJ¥ºi³f«SÈVß¿¿¥¥¥¤¤dñâÅÉKæ³~«3¿ËÉgçZXùâw©½½ýÑÄVÊÊÊ=ñèÿùüc¥±±±ÚÚÚ4«°Õ§N`òÜ´Y³Õ÷îÝ¿ÿøøxºdÉÂù	b[bCjÕç¨3gÎ¼òÊ+rþ¹Î½píÛ·ïÀiVa«ÃÔäO¨|6mÖluüáxõêÕBÛêèÆË//¨MV£qÛÜ¹s'N<Ê9úæOXôbuóæÍºººx6I³Z[]YYÙØØÛ~íÚµÙê¸ó,--¡joooluÔÔÔÔÝÝ]8?ÞÂêsWgggrPâ'ýÿ9ÿÇSzÆ¹páÂ£W¡ºi³f«ÓÝ¾;Ù3Q[räÈ¿$âïÙêþþþ|¶köýx«Ï×ßõxôÿçüLáàï+­Î,y-­Î¼óó¾wïÞ£ç³]³òÇ[XÉ*++ûúúb¢«««±±ñÑþÿÿcéùúÔM5[ßëd´Z8ßë­[·¶µµ=xÓûÒ¥Kd«-[vùòå|¶köýR«3ü¿|ùòÁÁÁG9úÂÏsþ>«°ÕÝÝÝÕÕÕñ½~ýõ×CÖÙêááá¦¦¦äåþþþÙêäHóã-¬JUIUI°*IV%IÂª$IÂª$IX$	«$	«$aU$¬JUiÖýÐ§RµµµSZÓ¦MYsV®Uz###âúõë']úé§fñyíÚµ8íììLÏ9LIXU¡÷î»ï7nlèÔDÉÄÁcQKKK&«qzøðá8Ý±cGÌìèè(--ýõ×Ch§$¬ªp»qãFèxöìÙL8ßï½Çþz¤RW¯^Ó'OfÎbÂ¿äUhãããaI³àL³ù®ïÞ½ã×Xúá&§JHÓÓ§OÇ:É¢XÓc+	«*¼õTj`` ¼¼¼®®.½ã7yûRL$FîÜ¹3½þýû÷¶¶¶7o¦¯$¦O8£££[¶lÉþJÂªTCÕ±±±ÄÅêêêIG«YÁ×¯_OÖéîîîéé¹páBú=M1óâÅÉ¸ÖÃ+	«*Ü1k¬Æ uÑ¢EW®u.]º§«W¯¾zõêª:::ÝÂÁ­GUVU¸¬f~h5ÕÑ¦_a½Ä·nÝJM¸¦WÈZ°ªBéþýû1ÄçÍë×¯OÎÆüõ%g£äUÕ05ÔÜ¶m[L9r$æïß¿ÿÎ;¡lÌ©ªªZºt©VVU¸²N:xÜNà`õøñãk655%ó,XÐÜÜì!U)_V[[[céüùó?øàöööÞ²eK²¨««+ÎnÝºÕ(	«R^¬Þ¸q£¯¯/¶§§çÞ½é³§ONï.$¬JÿÆêÑ£G=°*IV%IÂª$IÂª$IX$	«$aU$aU$¬JUIUI°*IV%IÂª$IÂª$IX$	«$é1ý_Ð3áãýëIEND®B`


常態 Q-Q 圖


êñx´Éöl¥vXÿü¥PïEQY-((î¶··k]ùþûïÕÃªIáï~÷»ef«ú¬z½Þ¤¨''­Û T­ö)©wÉö_|!W[U7?ÔG+**^÷¤ÿ[!Ô];+ÿDMMüÝ ¾XíëJÊj*wyQVåOù*N§zØÉÉI¹TÏyUU`3ÑZ¨·üA`Uu´öîÝ»êG2KUÏ¤ÊÕ>úh¹ß¥eõÑ£GêÅÆI÷ÒfI4ÛVnÞ¼)%~®vs||ÕË¥Á±XìE/0VG¶e)<<<¬f¢~¥§ÕÜeqVµ©¿ÚIVAV-"¬Þ¹sGMøÔaÕGTEÔ*HËgUÿÜªl$Ý@²¤-3¤¿~iÃÅYéo?11¡XJª¸ZP6ä?uêÔVhÉúâµ_[qEwÑgµ¶¶6;;Ûív«QI½÷ÞddØ:;;ÉªúO_|øáW®ÓRªB¥_>iÉ¬êß·ª-a(âp8ÔãëòÑlUæÇòIÊ'pîÜ¹¤ÀjÝ(õ6Ö%×STO«7À¼èó<öì¾ûÒÒÒNgGGz.vù/-õ»$euñ§÷ûßÿ¬¬[zí²úðáÃÉÉIíª)iÊ*KÚ«^Ö¤^¬´äk´4iWU,UAÖÖA,))ÑOµ«*ÃK¾hIóóó2UÇW5¹ß»wïKÚ¯¨¨àgdØÄ¾þúëÌÌÌþ©7mêiÜ8Ô1jõ¾áå¿L5mÈ* «UÈ*dUÈ*dUÈ*d²È*d²È*ÿÖÓÓ­]½páÂîÝ»¬ëíñãÇÇ&¥§§ïÜ¹3D£Qý>l2´×ÔÔXÈÆ'O^´sEôÿDÒçâ#ËÝO>­¿Z^^Î7 «Àz«¨¨]½zU¶¯]»&ÛyyyêC·nÝòûý¦jO]]lwvvvttÈÆûï¿ÿ¢)ZüOè¥òÈ_ýµ|ôÊ+ryùòå»wïÊÆo¼Ñ××÷Ö¾ËYÖÉ;$B>(qÒ7O]ôèÜ^6öïßÿ¢©þÞ.ú'ôRyäÁÁAËj6::ªJ¬ö?>33ï2@Vu+r»ÝÃÃÃIªgZó$`Ú¶l¨-¹3Eÿ	½T9)«2[9·öÑìììeYVåÒ¥K¦8p`rrrñRk[ZZ¾sééé/ÚiZÊ2sÖ%?ºä#/Õ7nÜºu«¯¯OËªLyù.dX?£££2¥Óâ×ÑÑ±ú¬®ì·wuY;vLR*×¯_W7®®®¢¢"¾ÅYÖÛÕ«Wóóó%HIoM1vxMf«www«¨=d «À«1??/Ú±cÇ§&µh¯!ZrçÌVSyä%³zçÎD"¡öøý~¾³YÖÇãöHd[GMzÇ§¾y~ø¡:J¬ÇË;WUíj*¼dVõUZZÊw «À:¹÷îÑ£GwîÜyüøñÇ¿¨yò¡`0(ÓÙuË%w®IVSyä%[øðá¥K´w²ò]È*Ãa­Çß|óÚN$r577!È*d²È*d²YÝþå_þÅn·;xÕ~ñ_nî¬;v¬¨¨è¯Úo¾ùÿñ>«ï¼óÎÿàUûÍo~CV «d@VÉ*¬UÈ*d¬È*YU²Y%«²JVd¬@V «d@VÉ*¬nÀ¬Þ¸q#???==ÝãñUY5.//ïúõë²qþüù½÷þøãW9tèYÕÉÈÈøáLKyûí·ù^ÈjªFGG;&Où»¿û;f«²ªGÇóÜ*¬®Ê½÷***îß¿Ï+duUÂáðÁ<xÀlduµrrrô/M"«²Êr²JV «U² «d²Y%«²JVd¬@V «d@VÉ*d²JVd¬È*Y¬@VÉ*¬UY%«U² «d@VÉ*d²JVd¬È*Y¬UY%«²JV «U²øÉÜÜ?Y%«ÕêííÝ³gÓéÇãd¬F£EEEf³¹¼¼¬ÌVÉ*ÀÙÙÙ«Õêñx&&&6þ'LVÔÅív»ÍfkooO$âs&«gjjÊëõL¦ªª*°n¢Ï¬6uÔ×b±K6ÝçOVE[[kAû&ýÈ*àÕ)((IjUUÕF~ÿYlh³³³åååf³ÙçóMMMmö/¬^D"ÑÚÚêp8ìvWW×Öø¢È*à),,´X,|²Ø¸fffÙl.++ÛGÉ*àÕH$ÍÍÍYYYn·Ëõ%«W`hhHjj³Ùêëë7õkÉ*àUÅb¥¥¥f³Y.7×Id°È¬ôäÉ2CÝ³gÏf¬6þþ~§Ói±XZ[[7ËBùd¬ÀDJKKM&SyyùÌÌÌ¶úÚÉ*`ÍÌÍÍÕ××[­Öm8d°6B¡ÓéÌÊÊísÔ¬ÖX$)..6LÁ`P¶·óPUqñx¼®®Îjµú|¾p8ÌUA]]]N§ÓáplÕ%È*`=x½^³ÙF²0"UUU©Ó£J²0B[(ßétvww3 d`P¿ÇãIjMMÍV:=*Y%«°®b±X0 1 d`D"hll´Ùl2Oíííe@È*À þþþ=öÈ$µ¾¾~Û.DV«555¥L²ýÒÛ·µµJ·üéTÉ* UsssÕÕÕ2C-,,Jå.UUU2©mooÙ­ÄÕétnó·±UÀÿ#it¹­±±1§réh,ÓöÔÔÔÈ¬UØ¾FFF|>Éd(®è(n]]]SS~Ü=++¬UØ¤+=ê«W[[ÛÒÒ¢ßH$d¾KVÉ*l/Ò?)¢$Ðår>=j»ÇãÑß·¹¹ÙëõU²ÛH8v»Ý¥®®n5oûúýþ¢¢"éNMMÕ××;Y%«°-D£QµdR XóÇãq©©ÌPeÚZVVÊrÈ*YMOfê¨/K&U²Æ¸Ýniª%È*Y"H PK&q¾q²JVÀ uÔ×jµÊ<Uf«Y%«`Poo¯Z2©¥¥£¾d¬AHÄï÷L¦`0ÈQ_²ºäääUXz¯Õj5¶d¶EVÝn·üÙEV`¡P£¾dõå¤·nÝÒ²úÃ?ür×_ýÐ¡C|/lOSSS~¿ßl6WVVrºS²-«?þøãEÞ|óM²`ÖÖÖªòÇÆÆ²ºâ¬rP(d_ÐÖÖÆQ_²JVÀ ú|>¤ÊT£¾d¬AÑÊÊJ	ªd£¾d¬qmmmv»ÝétB!F¬²½^¯:ê;77ÇU²FhGý~?§2%«dkii±ÙlN§Ó£U²Æx<ÅR__ÏQ_²JVÀ H$RRRb6ËÊÊ8êKVÉ*$³ÒÚÚZ«ÕºgÏ/2 d¬AÒQ»Ý.M=yòd<g@È*Y#fffÁ Ùl.--D"Y%«`ÌJdêõzÃá0BVÉ*N§Ýnojjb¡|²JVÀ ªÓ£òæ²JVÀ íiTÏ722ÂU²F$ÆÆFµdRww7BVÉ*=LRY2¬U0.AÉäóùä¿Z¬U0"H477Ûl6Ãx­/Y%«`ÐÀÀÀ=¬VkCCGÉ*Y¦¦¦M&K&U²)hll¬ªªjnnU;D½Åb),,dÉ$²JV %]]]YYYuuummm@ÀårÍÌÌtwwËÍfkiiáiT²JV %RP»Ý®Mï'¤²f³YËQ_UXööö`0¨¶gggÕéQÓÒÒþíßþÁYijjª««¶¶6¶Ê<µµµÕétÆb1dV¦¿¿?//ÏëõÍfªÊuddÄáp02 «°2ÑÊÊÊ×^m×®]ÿþïÿþ¿^ìñxdÂÊà¬@ªDKKZ(¿­­­¬¬,++K;[*ã²©p»Ýê¨¯~É¤X,Æi@V UÑh4L&¿ß?55Å¬2mhhPGU0H:êr¹,§GYã"ßï7L@£¾ «`P<¹©Õjy*GAVÉ*ãB¡Z(¿¡¡÷¬Uù|>uÔ72  «djÉ$³Ùìv»U²À µP>§GY%«V%½^É9ê²JV$ÍæÂÂÂ¡¡!d¬0B[2£¾ «dÀªhåWVVÎÎÎ2  «dHD-_XX866Æ¬U577[­V»ÝÞÖÖÆQ_U²À ù­  Àb±TWWsÔd¬0Hõ5ÍÅÅÅ333È*Y`ÐÉ'ÕBùýýýÈ*Y`ÐÐÐPaa¡ÅbijjâiTU²À H$RZZªÞ<ÃQ_U²À ¹¹¹ÚÚZ«Õêv»Y2	[9«7oÞô/ÝÝÝ&izzZÛ£v>Zöûí·dýýýRSÍ¶7ÏLMMµ¶¶677súlè¬ÎÏÏ?öì¿Âd¿%ïÝ»§öÈÕ'N0[`´°¤¤Äl6WUU­æ¨oSSSVV<H]]ËåÇÇã/6bVU>ÇÇÇÓÒÒúúúÔÙýòC,»víÚ¿?YºÙÙÙÅâõzÃáðjJ¦§N§S­®JPý~¿<8UUV¡¿úùçkWsss÷íÛGV¤¨»»[æcML¿ï[[[&Áòø36bVåàìÙ³ÒÎÎN¹=ryøða§A5m­¨¨ «^jll¬¨¨hõGõJJJ¿·UæÁ66bV<y"?vÞ¾[.¯]»&.]Ònãt:óóóÉ*eÄb±ÊÊJ	ªduddd¹¶¶6é¯TÖãñ0æØYUTVO8ÇÕAàÜÜÅj/f¶`mmm6ÍårB¡5ðH$b·Û[[[ÕËäêêêbØ±Ñ³:88¨=·úÅ_0[ðRòÿ×ëµX,2¡üùLðù|YYY/^dä±É²*)ÕÏVÁ Y FËËËÍfsIIÉú,$³UNqMÕË/Ë¯zÚuzzZ~y>|HV(2+mhhPå3qYý=zôôéÓû÷ï«+-~Ëza°¬ápX-ßØØÈBù «É¾ÿþûÏ?ÿ¹÷nSÝn·võÔ©SêuÂdØÎäÏë²²2³ÙX(du9Ú1Þx<.½råþ£2/_&«À¶¥?¸å? Fd3Ø0èâÅÒÔÖÖVú¬UE"ââbÉTYYÉKpAVW¶&ð~¨6ÔªúÃÃÃ§¼¼¼¯¯/''÷­ÛöZ_§rÔdÕHVO>­6ä·H6.]º$ÛwîÜíîînùí"«À6ÑÞÞîr¹ÔÂFõY]ÑÑQµàÔTmmÙý»wïª¬fggU`Ëòù|jX,Æ¬®vÆòÌÌLÕ÷ÞOö¨×h³U²lm2+­««³Z­«?=*°­³ª>sælÈ/Õo¼¡eUf®²þî»ïU`«joow:YYYkrzT¬¾p¶ÊA``k)((XÛÓ£duÙªJìýû÷É*°%ÍÍÍIJ%¨ÅÅÅcccÈêÏÕ¯¾úJ½YÀÀH$Óéd¡|`³zíÚ5u¼Wj*ò×«lÛl¶ãÇ?^ÝÙ*°eÃa·Û-ÔÚÚZVxÖ>«óóó*®/_~úôéõë×µùëï~÷;²l±XLµ¸¸xjj~¬uV8Ê¶ü%+Û.Ë	²lñx¼¡¡Áf³Ùívùuf@1«÷îÝÊo¶g||ôõõÉ¶üy«k3W²l.½½½.Ëb±ÔÔÔÌÍÍ1 ÀÏÕùùùÑÑÑÅ3W©©¶çöíÛjþJVÍ%Êï¯ÏkD(~×ÖÖJÎù`;ÎV9°õÈ¬´¾¾ÞjµºÝîuËü£Þ­­­ãäÉ|/@VWk|| ÒÓÓ÷íÛwõêU²¬3/:NÅ"SÆx<¾nÿnuuµLµEfggå¿¡¡!¾#Ø^Y=¼ ° û'j[½U¶S@¹qgg§l;wîÈ#?þøãEÞ|óM²¬¹p8¬Ê¼E"uþ×].WÒ:Muuu|_°½²úôéSµÚþâ¥!ÉÎÎV8??óÃ?8yýõ×:Ä÷X+25¬¬¬4Í¯äs°ÙlIë	·µµUUUñÝÁöÊê2+.¾ä6¬¥¥Å¶@6^áBù^¯W:ªßã÷ûåSâ²ºª¬¦¥¥iÛ;vì «ÀÏG-¤ÖåK&ÙívuX,V]]íñxxK¶cV=¦-UµÓìÚµKÝK.e¬?h4ÍfsaaáÆyYÐÈÈHQQÌ³²²'BÇö­êSªÖV/YRÛ+z¨£GvttÈèµNd0LæÚIØY]ÍAàáááÝ»w§¥¥97nU`°P>°½²ÊrÀÏ!ùõôûý,U²¤úZ­V§Ó¬UÀ m¡üúúz^Ul¬Ê_ÊdXHÄï÷«eÎ8êl¬ªWüh¯þÕ¶;::äêVu «ÀÏ!«òeÊy`ÍÕ'Op`C	BRSÍÖÐÐÀgMUNlSSSê¨o0F£°³ÇzzÔêHòòÞ½Õ2IòþÍ7ßUàg5;;[[[»ÑLÈêª~]"Å²³³÷ïß¯^»$ñc¶ü¬B¡ÝnååduÍ²*ùôz½ÝÝÝééér!¿ágÎ9ö¬:÷ª6//¬kkllÌçóÉ$u#,ÕµÉê;w$«ãããËw÷ðáÃdX+ÚéQ%«WØ:Yd8p@6&&&<ÏÑ£Gúúú¬Vë'æççoß¾ÍA``­´µµÙívL¶fVõïaÄêÊs«ÀÚÃ^¯W-ÏIÀ¶ÈêáÃÔºdX=í¨/åÛ"«RÐ÷ß_.zz¬Vkfff__é3¯dXF"hii±ÙlN§óâÅ°]²úé§ªmùåÏÏÏ×HâJVÂá°Ûí¶X,õ¶]VµvîÞ½»  @ÿ¡óçÏU`E¢Ñh04Í¥¥¥H¶ïlU[BþÊVÏ°NNNU Eñx¼®®Îf³íÙ³G~¹¥|´²²²¤¤Dæ²333°¥²ÚÓÓÕgÏÉþ·Þz,)R:N«ÕÚÔÔ$]æÕÕÕÒÝööö«Üu­U½;w9r¥öÓNúÒ£¾Réh,ÓöÈWîÈ0[0«ÁXÖÖÖZ,ÇS¹DT¦³ú=³³³YYY&@VÉ*¶µP(äp8l6ÛKúêI[ZZô<ã	U²mjdd¤°°P-¿Ò§EÛÛÛå¾úóÕ´µµy½^F «dÛDT-TPPâQß$Thii©zpWWÝn7öPÈ*YÅf%9lnn¶ÙlCæ«y¨¹¹¹ªª*õ²a¹ööö2¼Y%«ØF¤|G&©555©?¬Uà-++ szT¬UÀ m¡|ËÕÖÖ¦²JVp»ÝV«µ¾¾ò²JV"H 0LÅÅÅ,U²Çen*3T§1 Y%«A¡PÈårISy «d0hjjÊï÷«%8G@VÉ*`Ðììlmm­µ°°7Ïd¬ÆB!ûÞ<U²'SÏ'TªÊÈ*Yªò%«õ@VãÚÚÚìv»ÓéB²½^¯:êËIÈ*`vÔ×ï÷OMM1 È*`¶P¾Óéä¦È*`v»ÝòUÀ¸h4Ífs à¨/²H$ÔéQ9ê¬Æ©Ó£JS¥¬,¬i§GËh4Ê «ê¨¯:=ªÌVd0¨··×årÙl¶ú «AHÄï÷L¦`0ÈQ_d¬Â x<^__oµZd¬Â îînú «d«566æ÷ûÍfsee%§G@VÉ*ÖÔÔX,¯×ËéQU²Dcc£Ãá°Ûí­­­õ@VÉ*ÃÇjµ<yòU²"Hii©Ùlöù|333²JVa¶dËåêêêâ¨/²JVavÔ·©©  «dE"ââb³Ù,Y%«0Hf¥'OªÌS9=*²JVaRK&ñæd¬Â¸©©)¯×«NÊkU²fggëêê¬V«d53 È*YA­­­ö¼yY%«0ndd¤°°P-ÏéQU²ºÞ&''ýë_ÿñÿñ/ùËøØ¼_:êk±X'&&øÎ «du½õôôüÑýÑ¾ûþð?ÈôN&yý×½¿¬]]]|[U²újØl6ýøÈÌõµ×^Ön¢/A~F½^¯LRkjj8=*²JV_LUãñ¸~ç¯~õ«¿ù¿Ù$	2ÃæÍ3È*Yõþó?ÿ3---)«¿þõ¯ûÛßnðÏgµP¾ÓédÉ$d¬nééé'OÔçJöüáØÈs¿ÔÓ£ «duÃùçþç×^íÿñ%¨òÍv8»wïN¿n333eeef³¹¤¤òU²º555½þúë×´´´¼¼¼ùªÖÖÖÊµ  %U²ãÔQ_ÍÆéQU²ã¦¦¦JJJL&Z_d¬Â X,VUUe6GFFdu=<xð ''¬n1/^´Ûí6­¹¹£¾Èê:v»Ý&Óÿÿ|þÏ"duiQQLRkkkY2	Y]WÒË[·niYýáLKyûí·ù^n|ÑhT½y¦  £¾Èê+£eõÇü_:tÙêÆ×ÞÞîp8Ng[[GÕU[Ýúûûez*Ôºº:L@V_AD²ºÙÅb1õæÏÇéQUf«0(H477gee¹ú «dÆ]¼xQjj±Xêëë9ê¬²F£EEEògPqqñØØ¬U¡Õãñ°P>²JVa{»ÓéT¯õÝ°§²JV7ºÒÒRÉTVVÆéQ¬Â ÙÙÙÊÊJ¡:îînÈ*jkkSå755ñZ_ «0(½^É£Ñ(dFhGÝn÷ÐÐdF$ÛÙ`É$ «0(ËôT&©2Uåô¨@VaP4TÏÇI@VaP"hhh°Ùlv»=1 @VaÐÀÀ:ê[[[ËQ_ «0(Éä÷ûY2	È*RG­V«Óéìííe@¬Â é(§G²JVW+øý~É8êd¬ÇenjµZÝn÷ÀÀd¬K&Y%««255¥ú²P>U²jÜììlmm­Ùl.,,d¡| «dÕ¸P(d_ÐÖÖÆQ_ «dÕ ±±1ÏÇI@VÉêªÄb±êêjÊ²JVW«««+++Ëét²P>U²j|;=K&Y%««ÅÔéQKJJX2	È*Y5®±±Ñ± ««×úY%«É÷¯°°£¾@VÉêªÌÌÌ¨£¾ÅÅÅü4Y%«F$ææf«ÕºgÏ/òsd¬Ôßß/5IêÉ'9êd¬455U\Ìk¬ÕUÇã27µÙl.Kf«üàY%«õööJM-KSSo²JV)**2Lååå333ü¼Y%«FÌÍÍÕÔÔÍfÇÃQ_ «dÕ¸ÞÞ^Ãa³ÙÚÛÛ9êd¬422RXX(ÔªªªX,Æ(U²jÄÜÜ¤TUâÊ&U²jZ2ÉápØíö®®.~(¬UfggÝn·Õje¡| «duÔÕÕ±dU²Y¬UY%«²JV «U² «d²Y%«²JVd¬@V «d@VÉ*d²JVd¬È*Y¬@VÉ*¬UY%«U² «d@VÉ*d²JVd¬È*Y¬UY%«²JVØÄY½qãF~~~zzºÇã'«²j^Þõë×eãüùó÷î%«²º6222=vlÉí¡Cø^ÈjªFGG¥ ÕCüâ¿ «²ªGÇs@VWÆôuõÞ½÷ïßçÀ²º*ápøàÁ<à6²ºZ999&² «, «d²Y%«²JV «U² «d@VÉ*d²JVd¬@V «:ÕÕÕ¯¿þúÞ5õgög.k/RãZÀ8¤>Æ8¤èOÿôO®×ÿù3)ÊÍÍ]óámú¬F£Ñ/×Ïçs»Ý_"5ññùCäïÀCrrr>Ì8¤(3330)Ú±cGyyùÚ>f(ú¯ÿú¯ÍÕÃßÿýß¿ýöÛÏù5.++cRôWõWÕÕÕCòóó?øàÆ!Eò'ò¯ÿú¯CdfÙÙÙ¹>ÿY%«d¬U²JVÉ*Y%«d¬UU²JVÉ*È*Y%«dd¬U²JVÉêËÈ¯ñßþíßò3¢ãÇÿÓ?ýã¢÷ÞïÔ©SC~ûÛß~öÙgC~óßttt0)úÕ¯~ÕÛÛKVØdÈ*d²úÊÝ¸q#???==ÝãñË¹t8²gß¾W¯^ågBozzzÿþýjp®TLÿýÅp-ãþýû&kyO<©¨¨Ø±cGnnn8f¸^Ò6´´´õ®mÕ¼¼¼ë×¯ËÆùóç÷îÝû|áÕ­êbçÎ;rä?zòówéÒ%ÙAËÎÎf¸^êéÓ§òwUkÝÝÝÒ	ýk§NúäO=&Mu¹Wdd|Ös¸¶õAà¹ZÈOªlÌÏÏçääðS¸¤+W®Èüáz©?þøôéÓZV®eHSÕmkù¿qoÞ¼Ép­H,;pàÀ:×öÍêèèè±cÇd#==]Û©ß6ýÚ¹s§tBæ÷×òîÜ¹SPP ¿ºZV®eäåå<xPEí»ï¾c¸'òÙgÉd@¦ªW*JKKå¿úuþeÜ¦YôèQ xüø±l«ÃîÊ;øAààà®]»®þ_»víùÂó:jÃ÷î©c!×2dpÚÛÛeCþ?D®ºuëÖo¼¡Àõ.Óöü®¨¨¸ÿ¾º*µWGT9ð¢?®ü:ýOWêÔs×2ôÂ/c*N:¥_`dÝkÛe5<xðÁÚ£GªÅJäR¦°ü,êåååMOO?_xµÃz_ùéJå§ëöíÛê/]~º^ªººúÂÏ^ïõz®zë­·&&&Öÿ¿úmÕ¤ùÄðððîÝ»ÓÒÒÄE½ñññûöÉÆPókEYe¸1::êv»å§ËçóIY®å=|ø°´´T=ëÖ-+C ê5JëüËÈrUÈ*dUÈ*d²È*d²È*dÀ¾ùæÉôäÉ¹üøãeOffæ¾ûº»»þS§N½ðwÞdRç?Y:ûïÿuõñãÇrUìY¶§Oöõõøá¹.ÈåG$6-ëÃùå_jWÝn÷þýûäî.]>|XÛóé§Êjw=ÚY>TVzzødØRd:88(ÄÊå+WÿtútýlU«ì;wäêÝ»wåòÀj§þ|ÃêîµµµI¤¾òÊ~õÕW²çwÞí/¾øB¶Ï;Ç÷ «À¦÷õ×_ïÜ¹S'ìèè8sætNfú(?^m;ª¸Ê½dn*2Ý·oªvvvÊo¿ýV»»NOOKÂÕGÉ©,ãU`kýÆLþ¹ª§?3|ôÑGÒHUJýlU»:³úää¤lLLLÈ¥:¶lìß¿_cÕiyÙV%>ö¬ìÑÎ7 «ÀVpùòåùùy)¢z5'	Õ¼%g«êêµk×rrrÔ±°ìüî»ïvïÞäÈÙÐ?¾<ø»ï¾««û&Zo@V­3a¸ê_©+Û%%%KfUÚ9::*îÝ»§ÕXUoß¾ôàÓÓÓrùÍ7ßhÓÖf«2Ùå»U`+PÏ9sfçÎíííjîîr¹º¢?,³OUÇ7oÊ¥Ü¬ozQÚÈÎÎR>öLõa^r¶ª «À&öÁ¨°É¥F£Q¹WäCV«Uµ¢*ÅMz4Iéýû÷Õ>º¸ÌV²lYñxüÁêý-r©^I¤éTuìììt8I÷Rïp=zô¨VG#óòòøÙ*@Ví@½û%ÉeGG¶?--í­·ÞRª¬ÊG%´jcppPÍD'''åRæ¬êH¯LU_øÿ³U¬Ûz©§ªËãÇ«#ÀRÊaÒB>TjYUËJzu¥³ÕéééeUÆÝ»w/_¾¬]½wï^8ÖsPoK-))ÑÏezzäj"Êsssý~¿úP<eff.ÎêW_¥^ý$·O­ÖÔÔ¨5%øvdØ^öîÝÛÙÙ©]½råJÒBü2ýM°ªe¥ÊLI²Y¬²Y¬@VY¬@VY¬@V «¬@V «àùóÿ_Óòû¡IEND®B`


M¼ûî»Ó|osºÄ^no@­ËñãÇ]VS¶$YYÜGþwjsd%sîÞ½;MÛdÈ´víZm¦zXæk£Øiî.Z¹r¥4ÛËË+66Vf<xPæïÞ½[®eeeéïòæoÊ|ù¢2_ËÕììlí.			jùK.©ªÉWÒÕÞÞ.7©õÒ¯©Üäp8&~ªÖrwé®<		¹:Íëäs½KVå	JÌU « «þfO¨à4Yôèú§òäI¹1º*£L	Ã=´è4e[?ùämÚb±¨ÆHôËìÛ·O_ /Î¸"[·nÕ®Ê÷f6U¥;&·ÊÌÌL[É¹|22ñ¡Ôb~ú©~¦Ze	ÿ¤_®wÑgUµË'NÈz¬¬nFFééé.3]²*)u)«hmmU¯ý~óÝ®JõZkOOÏ­[·T½«¥BíýF÷º±~>úHöññÑ¦ÊpP¾÷îéA¾¯¯o^^,óî»ïª×µ«7nÜé¸¸¸I7Kdd¤Üê²GY_fFGG?»¨¬677o§­ÚÉª¦ÕìÓ¿òUà%"ýK»Ý.cMo©è2èT¯Áj;Vkjj´»Hn%¢ªp+W®iiLß¹sGf?~¿üòKë¹sç´w3MUù._¾¬o°>«SÝwª9Jpp°ö¾by ©w	©2(a__ß¤E*>éÛ ¦yc×¢Ðe­_XVõs­¬n¦©©I½iÒÑªVªÊ¥Lh¾8NµSpº¿%Iç²Ue&77wÆ¬Ê]vCÖ××OªªJ= Ëh5--M[FÊ§¥2Òô«O:wQë+fYm]d´ª&È*È*àÔ`NgÍÄ¬ªá©~áë×¯K±dâóÏ?Woý§ïº¼":ÕhUßLoß¾ýïöÎjû_]î+ßþAôdõ$Qjº¹¹YVÕNVeóæÍr¹qãÆ÷WËOlg`` öhfyI³ª¾:×ÔÔ¨ Êómç4YYÜÆØØÅbÑsÚÞJíE`ýþK¡EQYMLLîVVVj]ùòË/ÕÃªAáÏ~ö³iF«ú¬Z­VÔ]ÎÛ Tß­ö-©#dúÓO?«.ûVÕò@Ýíò¾'ýsRwíõXùùùùò¼A­¬¶^.YÍ]¦Êª<5µTÛÓÓ#j7YYÜÖB½é_VYU¯ÖÞ¹sG½áHFijOªxðàtK3eõÑ£GêÍÆ.÷ÒF.â2ÚVúûû¥ÄßèönvuuÉ´z»²4ØétNõcõÊ¶2åÛÛÛÕHTÑééYî21«ÚÐ_Í$« «À1¬Þ¾[øÔËªÆ©¨³ MUý¾UpY@²¤fH/ý©'fUj¤_¾»»[bÉ¥âê2!ßü¦:CÖ'Ûaús+Îé.ú¬ÆÅÅ©¢N&µk×.²²,555ÓdUýÓ|ðAss³JR*ýé&Íªþ¸Uíò f³Y=¾þD¾SVe|,ß¤|'NpyX7JÆ:éùÕîauÌTßçñãÇããã½¼¼"##«ªªÔ¾ØéWmöwqÉêÄoï×¿þ5YYõÚ©²úàÁíªÉe8³,iïvy[z³Ò¤ïýÑÞÐ¤]U±Tu9½p__vÄôôtýðQ»ª2<é!@ñ¨zùÙï¢÷±±±3j?;;ßIUÀþùçÁÁÁ/û¤îhÚÙ§ñå¡^£VÇO¿jØU@V «UÈ* «UÈ* «UÈ*dUÈ*dUT__ª]=ölxx8 «Àöøñã=öH|||322~Í7L&máüüüq2ñäÉ©fÎFOOÏºuëüýýwíÚ%3ñå#ËwxäÈýÕ¬¬,~¸Y^´ììlÐÅeúÒ¥K2­nºqãFZZiSXX(Ó555UUU2ñî»ïN5s6âãã%¨×¯_ïîî;îÞ½ÛeÙ<²´ùóÏ?[å²©©éÎ;2ñúë¯766¾92@VDÂ&zðàÁ¤C@>«êê£GdyHJJjæÿM&¶ºÌÍ#·¶¶ÊXVÎÎNUb5çÌ3ÁÁÁü²¼ +W®ÅÅÅµ··»Ü¡z¦eU¦MËêÙ¤3çäòåËrÇCgóÈ.YÑª¹µ[CCCçydsçÎ¾³víÚãH­m^^^úÎùøøL5Ó4I¿ÇGGGË­öËM>òôY½råÊ7µ¬Ê2@V§³³StZüªªª=«³g³Ùä^[·nxÓ,³ªöËJJeB¾já¼¼<ð÷÷OMMåGUàE»xñbBBÂÄcç÷"ð,G«'Nùf³yxxxâ­óx¸®®NÕ411QÍ!«Y~£££Ò!__ß©²ªµÆiï!tæl8NùZÅ¯<OóåfÕÛ·o¨9iiiüd²¼ EÚ#qiõ:ªËú¬~ðÁêUbý/ÎÝ»wËògÎqýcþî+Îæ'Íªþ¡l6?e¬/È;wvîÜèåå¼gÏÓ2è³*7effÊÓßß???_-9éÌÙôUbmz6<é¾Õ;wN;2@VÌJ»Ýn×z|áÂ5=22"WW®&È*d²È*d²YüñÇaaaf~hË-ëììtï¬îÞ½;55õ?´7Þxã?þã?Ü>«7n|Àí¯ÿú¯É*d¬È*YU²Y¬UY%«²JV «d@VÉ*¬UÈ*d¬È*YÕ6«­­­&¬Èê³KHHPYýúë¯'Ø°aYÕY9tèÐ#GTV¿úê+ÓdÞ~ûm~²:Û·o'&&>úTøÉÿ÷Ïh@Vgf³Ù.]ºôí7Ä¾UYF./öUY>e´ «d@V9d²JVd¬@V «d@VÉ*¬UÈ*d¬È*Y¬@VÉ*¬UY%«UÈ*YU² «d²JVd¬È*Y¬@VÉ*¬UY%«U² «d@VÉ*d²JVd¬È*Y¬UY%«²JV «U² «d@VÉ*d%¬UY%«UÈ*YU² «d²Y%«²JV «U² «d@VÉ*d²JVd¬@V «d@VÉ*¬UÈ*d¬È*Y¬@VÉ*¬UY%«UÈ*YU² «d²JVd¬È*YÌÙðð0Y%«g500ÖÝÝMV_¬^¹r%!!ÁÇÇÇb±tuuUxùµµµeffÄÄÄÕ%«ÑÑÑ/_3gÎÄÆÆUxiTTT$''L&«ÕZ[[+s¶ÜéE`ÿ¯¾újÅK,Ù°a¿ÐðæççKsrrNçwe>åççÑÒÒbØ­ç6YíììQé×_t7Þx¬Às100 uÌÊÊêèèèîîÎÍÍ¼víÚTËÛíöÌÌL©iXXÄØáp|ºGV=z$O?~ÌÀð½Ê§#eÝ±cËb###ÕÕÕ)))&I2´wüºqVïÞ½ïÞ=Þ	ß·äää«W¯êçÈÕb±hW%¥TÉjCCw ºqVívûºuëîß¿Ï6ðX­Öý¹(½½½999AAA~~~:lfád5""Â¤CVàUTT®Úl¶íÛ·§¥¥yÍfY@¬l(wÍ*§ixxX¬Ñºººßÿþ÷EÆ¦2ªINN®¨¨àõ^²i§Iccc||<==e´jäfÈ*`þÔ3ÞÞÞAAA½½½l²óUÀLYY;PÉ*`Î8`¬Ux8`¬UxZZZ8`¬Ux&ÃÃÃ111ê'OÖÖÖ>|¸²²sùU²³500 £Ò   ¡ªOZ,/sä¦óçÏ³¡È*YéLuÀU®jISeÆ¬d¬À$O>=Õ3N§Ól6»¼ãWÆ¬l:²JVàO¥ÔÄÄÄêêêÌtttHq]fÉÙd¬À·®]»§ÉÊÊæI2r		qy°V¸lF²JV]KKKzzºÔTZXX8000ã]rssSSSUYÕIcbbø(r²JVä°¼¼<99Y0#cÍÙwQî«Î!T³Ù,wçô¿d¬0(§ÓYRR&AMKK÷'ÌÈhÕn·ËèU²À®^½*£L??¿üü|¹ªæË8UF®¹¹¹RJ6Y%«0óçÏ§¦¦zGFF>|xhhH»I¦­V«U½X`U²®Ô%¥&I²ZYY9ñÛ¼¼<Í¦Í¿víµ­­­GVÉ*üvÆÁ?a&**ÊåÝ¿2f-,,dU²<ã`@@:óóóg<`FÒë2nnn.[¬UÆ%i<útjjê4'HÕjêçlÚ´©¬¬MJVÉ*#*--T03×ikk²JGNNÅbÑ¿§	d¬0õz¯:AR~~þ¼ÏÉ eMNNV¯geeñÉäd¬0VVWWk0S^^Îà¬U3GKJ%¨ÕÎpDVÉ*¸ª¯¯_±b¯¯oPPÐúõë'···WwÆf@VÉ*C+//÷ððÿEMMMÇ×ÊÚÒÒáíím6ØñIVÉ*LG 2Õ®J8/^üË_þ²¢¢BûõN]¶Y%«07ozyyé_õ¬þä'?ñññQÌÌûf@VÉ*#fÕÓÓSeUÛ*MçCLÉ*Y9óóó[¿~ZZO£¢¢>þøc__ß_ýêWl²JV`Ô'ÌüÅ_üÉdZ²dÉßýÝßeggKe£££'¾d¬+§Ó)ÿ:ûûûõ0ÓÐÐ¼téÒ+VäääÐT²JV`#%%EÆ¦aaajxºÿ~Y9èk¯½¡)--ý«¿ú«÷ß-²s ãÑ²²²ððpý3½½½!!!²³200 v ÔÔÔoît8l(UúÈ6uÀUeÀzúôiªºb­¬ÀôÙæò	3N§3,,¬®®N[877×f³±Ñ@Và[555ñññááárù/ÿò/ê#Û¦ù¶¶6îØ±£¨¨Èjµ&&&ò6`UøÖÞ½½¼¼äÈ?ÿó?/_¾03ã'ÌÈU³®­­åå_UøÖÍ7=<<òóóÕcbbþáþAæôôô°q@VÉ*9Þ¶m÷8ÉªvÀLxxøþýûÙ> «dÀ¬hÌøùù-Y²ÄåfV¬XQPPÀVY%«f 0c6¥¬ÍÍÍ&©­­M[àøG=Ûd¬þäääíFë×¯÷ññÙ»w¯ÄU.e1¶È*Y0ÁÁAuÀP322>þøãÚÚZwùîß¿éÒ¥-		²²Ñðreµ¿¿?mü×ÕÕÉsÃ¾¾>myäÈÿÅ_UßÞÞÞuÆÁßüæ7RV??¿ÀÀ@¬¬,6Ü#«£££O>ýãCLwïÞUsäê¾û­ø^µ´´¨fd*CU¶µµyzzj#Ñ//¯¶Ü «*]]]ò[ÛØØ¨æøûûË´ÌÏÍÍW_5))¬x+**bbbÔ«««µ¨?ýéO×¬Y£_XF«K.e£Á=²ªÊ*ôWO<©]]¹re||<YðxT0##ÔÌÌL»Ýî²@xxø~¨#ã×E±éàYmmm­««;~ü¸¤´¦¦F.eÞ¼YÆ©ò¯­ÙÙÙdÀ3êèèÈÈÈP;P¥¬.&ÏãþóëçHeÙp¬>yòD.?~,í¼uëºtI.Ï;§-@VÌ:`&==]§qqqeeeN§såKJJdÉ¦¦&uõæÍK,Ù¶m[nUEeuß¾ÃÃÃêEà+WÊ(V0£Uó088X^^®±ÙlÚ5ò?gÿþýò!''çø6Ë-«W¯^³f<,ÉöeµµµUÛ·úé§2Z0o½½½YYY!!!yyy~dC/^ìçç'íbtT¿KUF«?ÿùÏ¥¯2¦Âí³*)ÕV333É*Ùèèè©úuÀÌTKÊTjª%Sò)eÕYUy(¿èj·k__ÃáxðàY0fN>=ã'NüL·åËçää°1áÆYôèÑØØØ½÷Ô&r£Þ,e%«&500pøða³Ùìçç·cÇI_ï¨¿¿_F´.3¥Çë×¯gÂ³úå_<yRÂ®oj\võÀêÂd:`FêVPP0ýû'òôôÔÞî«q_¸wVí5Þááa)hss³þVÎÊLùí'«~wÀÌ¦MÔ3ÚþÑÁÁÁÚÚÚúúúÙ¼ÉHþÈWþ£=Yª-f_,à6YålÌ4O½Þ+OµSSSÏ?¯ßúÎ;ïxxxúúúzyyýö·¿ñW¯^-%wB/Y²ÄeðU²,LW¯^Ugñeffæµk×Ø»w¯¤QûDñ_ýêWRVw$Mª¿¿¿¼¼¯qNVåÙâ| &ÔYõÛÛÛ-KVVVcccDDÇ­ÕÖÖ¦^ïAªuªi/^ìr"ß¨¨(þÒaÜ¬9rDM$&&ÊÄ¹sçdúöíÛ2]WW@VC*++ò¯Àjµ>zúÑ¤Ëò¼|ÕªUlI+«êòÇ£&222´Óîß¹sGe544¬nMþsrrÒÓÓ¦:Ç½"·ª×Õ?»Ý>ã¨Â×××eÏè5kÞzë-¶<UíËUVwíÚ%sÔ×h£U²¸µ¼¼¼ÊÊÊkdd¤Ãá¸433ÓÛÛ[*õ¸u)))r/í%bI¬_å	:ÆÊªö"ðÑ£Ge"  àõ×_×²*OTeÂÇÇgëÖ­dpSRé¨þ ÒÂÂBùëÖ®ªf¤òWUVV6£/_.ÿ.d/MÍÊÊbãÃ¸Yt´ÊÀÀ -))ÑÏj¨ââbI©:ã`CCÃl^ïÆ±cÇ¶lÙ²mÛ¶ÚÚZ¶<­þi´ªïÞ=²¸¦¦¦½÷JGoÞ¼©æÈT¿´3000''G;`fgÈê<³zêÔ))u¼¸õêë+d0êéé©©ÉÉÉÚ0´¥¥å'?ù,6ý3YK.©×¥¦2+ÓòvÏ=gÎQË0ZÜÂ¶mÛ|µ30¼ÿþûRÖ¶¶6	ªÕj'Çü±úy®ø¯÷du£££*®MMMccc/_ÖÆ¯?ûÙÏÈ*àF¤©.çzõúõëþéþiÑ¢Eòwííí½jÕª6ð½dU¨Oá©LÛív>ö¬UÀxxx¸ÌÙ¼ysPPvÀLoo/[	ø~³z÷î])¨SÓÕÕ%seÚétªµ+Y^æÑjyyùÃÿÀ¿¿ÿüÈê|^îìì8rjsnÝº¥Æ¯dxyÔ××ËQ||ü-[´©ï¼óÎ¢E²²²Ô3f³YÆ¯­­­l.àÅVùÀíìÝ»Wz¬êååUSSÓÛÛãíí­Þ$$±äpû¬vuuÉsdùçÑ/^$«Àó%ÿú·&­_¿^æh0#W¹µ©©ÏY~¬vvvn1®î;jZ½*Ó³@YX ËÄ'¶oßþõ×_à7Þ «ÀüÈ4::úáøÉ+**Ô32xýÇüG~ø¬©³íO<5Äüªøê«¯"'X²dÉøYó°eË¤¤¤õ_y"ÛÒÒ"Ó.Öøa²:ÍæÇÇÇgÒi^ÝÕ«W-ü¾òÊ+Ú3mmm2ZÕNU`AeÕËËKöõõ%«À¼õ÷÷ÿô§?]²dÉâÅ-[fµZex%sBBBêëëeùCãcM(«O>ÕNáUu¦9yõÕWÕ½äR¦É*0ï¦.Z´èµ×^ûÅ/~!òµ²²rdddxxxÍ5r«R¥©|þðÒVõ)UçVoYRÓsz¨;wVUUÉõ^'²ÌhÕªUÁÁÁ~~~;vìèíí_¿~=[p¿¬>ËÀíííòÇ/O«Ífó+WÈ*0W2¿Ä?û³?ÓÂÌûï¿/VlÀXYåtÀüÔÖÖª¨réëë[UU¥_`ÿþýd «dÓé,//WgLOO·ÛíÇ?|fÅúÅBBBx «dÃáÈÏÏ÷ñññððPÚöÖ[o©³#õ÷÷/Y²déÒ¥ûÇIS9pû¬Uà¹kiiIKKóóó[¼x±§§gqqñÃñè©(=?Ò-[ÌãdJS=«­­­6M½ã711Q÷¯6]UU¥NÛMVçBJY[[,Y)))§Nqª:UAªËnÕ'Oð	6À188¨ÞÊ+#ÔYSSè²ä+öîÝËÜ/«|0ðzU:&¿ù¿ùÍoZZZÔMòÇ¼hÑ"			ùðÃÙngUþªëëëÕÙ¢¢¢bccÕiL&ÓÈ*0êÍæíí'eÈÊÊ*,,¿²ôôtUS¼nÛ¶M»×±cÇ<==µO&à®£ÕË/KDNghhhRRzïÄÑ*0Wê#Û¤Ô;v?^¬2?]m´´´üüüãoSÊÂRÜäädªgUòiµZëêê|||äÒßß?((èèÑ£ÇW½*¡&«À/^,ãÔÿüÏÿTó>,Tý½½½R5Ýßß¿eËU«V½õÖ[MMMlFÀ³zûömÉjWW×ôÝÝ¼y3Y¦a·Û333ex*M'¦û·¦v£æåå»ÜÅÏÏí,´¬J2×®]+ÝÝÝeçÎãöíÛ7::zëÖ-^&522R]]¢ù¿ùq©[¤¬×®]+--Á«þçÏc-«úcXåÿ¾ ì[ÁÁA7ëªÅÅÅQQQ2èg¢òÄôáøxôÉ8U###Tqe+w³mÕÍ7gSçÈÎÎ&«02SÆÄÄÍféerr²jgoo¯:`Fè?aFµÓå***da1«gåqÔYjkkÙ¼ÀÍªôÝwßËúúúàààÆÆF¹ZYY9ãW²Ên·K8Õ'ÒÒÒW^yeÍ5ÞÞÞÒZéåÄ!¬,/ùÔÏ¦ª*r-ÃrV?ùä5-O·ô7É¿²²Ùl~øÝ3ROOÏåËk's¨  Àjµ:uµººZ§.¡`¬jíOLLÔßtæÌ²­££#777==].Õç²	ÅráÂ¢¢"ª¨¿ûÝïÒÒÒ¦(Y>  @â%%V')`ÜÑªv:¸¸8µµ§§¬bçf³¹¸¸X¡%%%aaa26¸.[¶ÌËËKnÒv geeÍøCCCòWÚÛÛ;22ÂæÕúúz¬>úTæ¿ùæ¼e	ÓéÁ¥6B<xÐÓÓS~ùô£ISµc«òÕ9Ü¾;§ÚÇB%È«W¯jï6jhhHOO¨;`ÆÛÛ[òyàÀão8Ák~~~^^^PPÚÕ¬ò	6ÀÃ¶¶¶ÈÈHÅbµZeZQQ¡²¦0#Ò]uv¯ÒÒR1Û «døÖµk×dÜyúôiuµ££C¤ÒËµk×L¦åË«6¨%''¬UàOòóóÔôððpeeexx¸:ã`ff¦$¶ººZRZ[[Å½Y%«p2L,+++--Õ>%íÅHOOoiiq8êoooÍ¦©®®nÓ¦M111iiiÚY%«x©äååÈQ&^Ø!(ï¼óÎ_þå_JMåÈÍÍííímhhHMMåU²·$EJÏÔÕÁÁAÅ"ÃÖïõJ¶kkk­V«Édòòò«Ý;ÎÄÄD7ósÈ*Y[1¢Ëg?þû/J¶¥Ù2&V;P%®¿ûÝïd¨¾cÇÐvµ «d/»«W¯>º­­M;BTz&Õ/síÚµçþ¥åaóóóÔõïé¡ª|XmÐ¬U¼Ôd¸iÓ¦ÈÈHZ­VPçÅÍ§_R¯²äsüÒêÜê|úl²w"AÕÞTQQâp8Ô£Ruåªgý+ÊX611Ñd2É¥<òÄl²÷ãt:%êmAAVVV>?ºÆb±Heegojoooaa¡MeÉÇÅ «XP$l2ZuYTT¤ÿ(ï¡qÏø$ÉÒQuÀUrÎÆ@VáÆ$ùùùQQQAAAÉÉÉêíHÒ9ÚçuêêêÔÔTõz¯Lóz/²JVÝäÍjµÚl¶¹*yS¯+ÙSeÅd¨óìñk0#_Wû(7 «dÕíUVVJVõçHª¨¨9*¥999êmÀ2Æ£YäîÚ3òÈÝÝÝld¬.(.çviWe´*JË>Ëé	[ZZÔ3gõ²Y%«Saa¡ËÇ¼HóBBBËËØ7&&Fí@évê`d¬âÐÖÖæp8´9yyyÏø°2ºÕ>afÓ¦M2Z%¨È*Y5ââb)ëûï¿/£II Åbyiív»:`FZPPÀÉU²º`ÉïÜþýû¥ ýýý.óefnnnYYÙüÎ¨III1LQQQó~ «pÉÉÉ+V¬Xºt©tô¹<¬äS0£>a¦¡¡×U²ºÀmÛ¶Í××·§§G]-//Ä>ãIsrrÔ'ÌpÀ²JVBúÛßþV?gõêÕë×¯ß£µ´´¤¥¥ñ	3È*Y5(9YYY«V­ÓèINNæd¬w´êrÚiêìG«úf222d´Ê&@VÉªq½óÎ;-jjjRWdü:«0¬UL^VIi`` }||µºàd¬bÆÚÚÚiÉ3È*YÅsÀ3È*Y5®ºººüüügÿhq@VÉª¡Ùl¶¸¸¸Òq%==/ÕrÀ²JVñ°¤¤ÄjµJµ:¦¦¦Îéd0¬UüDÔåØ»Ý.¡ÍõÌäæærÀ²JVÎb±¸äðÚµkqqqÓÜEÀLLLLyy9;PU²oíØ±£¨¨H?§¸¸8##cÒ%eeeêæÖÖÖ²Y%«øîîî°°°Ã+))«W¯^uYLF´ê)1Ì «d»víZzzzÈ¸´´4d¶´´ÈàÕÛÛ;22fU²ùÐï@µX,Ú»¬U|;*ÍÏÏß´iSVVVCCÃTigªb9`Y%«puþüù°°°¢¢"ÉdYYYHHË»=ÔqPHØnÈ*Y«é¨þÄÝÝÝ~~~Z8µ3Ê µ¤¤¨È*YÅ$¢ÅefzzzmmmEEErr²Éd¬ò	3@V1«¬&&&êçÈx4..îÏÿüÏe#¿@l% «õ"°:I¡$633Ó×××ÃÃ#77×ét²¬bnjkk_yåõ	3r¹lÙ²ââb6UÌÍÐÐPii©¤ÔËËËl6GDD¤¦¦±e¬bÔ3!!!yyy0dóqþüyÍ¦Î8¨Î÷Ë6²¹®­­MLL4LV«O²ùp8EEEaaa~~~0d¬ÎÇÕ«WÕÔ   )«ômd¬ÎvÆÁ¸¸¸²²2ý'ÌôööJnùÌ «Áàà D422Òd2¥¤¤ÔÕÕéw vttÈM111V«U°¥¥¥l1 «Ãá(((P;PssseHê²ÀÀÀ@HH¤T¶»»[:Mdb·ÛÓÓÓ½½½Õ»MuêáÃ333õsZZZdäÊ²oÏå[]]­INN®¨¨~wi^^,ã2S¶lIX8Y½råJBBÅbéêê"«³188XZZ%#ÔM6É s6÷Ñjnn®~NGG£UXPY¾|ù²L9s&66V&þß999dUéîîÎÊÊÍ2§3ª«uuuêªÓé1®´­'«zþþþ_õi2o¿ý6?Ë°°°ÈÈÈ°ÎãÎ?/INMMÝ±cLäååq¢%XYíììÜ½÷×_ýolØ°Ñª6ZÆãM$®µµµß*X YôèQFFÆãÇÙ· «s£½´«®Þ½7;;ûÞ½¼@VÝn_·nÝýû÷9À@VUDDþ­Id@V9¬UÈ*d¬È*Y¬@VÉ*¬UY%«UÈ*YU²Y¬UY%«²JV «U² «d@VÉ*d¬È*YU²Y¬UY%«²JV «d@VÉ*¬UÈ*d¬È*YU²Y%«²JVd¬@V «d@VÉ*¬UÈ*d¬È*Y¬@VÉ*¬UY%«UÈ*YU²Y¬UY%«²JV «U² «d²Y%«²JVd¬@V «d@VÉ*¬UÈ*YU² «d²Y%«²JVd¬@VÉ*¬UY%«¸V[[[M&YÕg566@Vdõ98tèÐ#GTV>ºØØØ6ð³ÕÜ¾;11QjªeuÃË-#«²:3ÍvéÒ¥o¿!^Õy0G?­Í!«²úZËh@VÉ*¬r:È*d¬È*Y¬@VÉ*¬UY%«UÈ*YU²Y¬UYýäåå-Y²$ö¹zíµ×¢¢¢béÇ?þñ~ô#Ã®ûÊ+¹îÑÑÑ]÷Yw¹4æêËºËOß°ë.õÏ÷1¥GnUÃñÙó÷!mÝº5<<Üë¾k×.___c®~~¾Édú×ýW®ûdÝ¸îeee²îûöí3æ¯½ü½gee=ßÇ¬®®þÿù÷Îê÷á¿üåÛo¿mÌuÿðÃå)1×½ªª*00ÐëÞÖÖ&ÿ^ÿ÷ÿ×ëþ_ÿõ_²îÊºÿû¿ÿ»1íedYSSób¾Y%«d¬U²JVÉ*Y%«d¬U²JVÉ*Y%«d¬U²JVÉ*Y%«d¬U²:÷Þï¿ø1×½¼¼|ãÆÆúúúc®ûåË_íµÇpÝoÞ¼)ëþßÿýß"%ë~åÊcþÚ¯^½º¡¡¬àfÈ*d²ú»råJBBÅbéêê9ri6eN||üÅðº÷õõ%%%©5moo7Ôº+­­­&Óç³î÷îÝ3éjÝ<yíëë»råJ»Ýn´ßyýÏÝËËËP«/?î¸¸¸äßá²ùòe8sæLll¬Lddd¨w8qbûöíxÝåéÜ¹sß¿i%44ÔPë.ÆÆÆäUã¬]]¤E?Ç8ë~àÀ>úèéÓ§òO6**Êh¿óYeY_C­¾ü»û¶LÈeDDÄ¿ìïï¯¶¾üÕÉÄèè¨Úú^ss³Ö¶î:räUã¬»4U=ÒÿÇ1ÈºËSÉþþ~c®»Æét®]»Öh«/Ï¢îÞ½+r©Q½°u7nV;;;wïÞ->>>ÚLýôB±JZd°n¨ug¬òG¥eÕ8ë½nÝ:YGÙ×¯_7ÔºËÚ;vL@Ë?Ö£ý½+6MþÝí×¾««K~îò÷.jõ_Øº4«=ÊÈÈPî©]¯¯¯V¿µµõÕW_5ÔºËK.3¾·IÍ1àÏ]¶«W)³î²¦2!Ï'äYî7nÜxýõ×õÄ «¤^¨¾ª-ðÂÖÝY.ÙÙÙ÷îÝSW%0£££ê#POÖ³î¦ÿË°?wõßÄ8ë®_;£ýÎ+ñº~dõ'M_Øº.«v»Ýºu÷ïß×æìÜ¹³ªªêñÚÉv¯ttt__ß7ão`¨u×÷Õ?÷[·n©'Fû¹çåå=öñ·Á[­VþÎ¿ùæÝÝÝüw'#Tõïî/¾ëÃe5""ÂeÔÒÞÞîååe6ö½ºººâããåÛÚµkÕ`Ý8ë>1«ÆY÷ÎÎNu¤AJJzqÖýÁ6MíW¾qãçÕûtökïp8Ôñr)Ó/rÝ9d²Yd²Y¬²Y¬²Y0.L¦'OÈå¡CdNppp|||]]]FFüLù7o2©Ïrú¼÷_ÿú×êêãÇåªúàRdX ÆÆÆ?øàÜÙ³gåòàÁrd·Ûõáüì³Ï´«qqqIIIãäîçÎ6oÞ¬ÍùäOdÎ´»´··ËíS>TVëëëùdXPdÚÚÚ*ÄÊessó7ßH»~´ªUööíÛrõÎ;r¹víZ5SÿyÃêî.s¤¾òl6<à©S§dÎÆeúÓO?é'Nð³È*àö¢££?ÿüóÀÀ@)d²ªªêèÑ£Ò9qê£xæÌ5m6eHªâ*÷±©LÈ`4>>^UkjjdÎ_|¡Ý]Mûúú$ájÎ£GdTíU`aýÅL'OTcSµû3xÜÁ¥ªúÑªv/õùí===2ÑÝÝ-êc½e"))I¿°ê´<L«?~h.]çU`!hjj"ª¨ÃÃÃ###ªyVÕÕK.EDD¨×r%À2óúõëáááÛ·o	ýãËoÝºU½n¬îk³Ù-Ã «ÀÂ°JïÔéôôôI³*íììì9wïÞU»Eµ«êåß[·n¹<x__¸pAæªq°6ZÁ.?¬ÚKzôèÑÀÀÀÊÊJ5vôññªûþE`ª:ö÷÷Ë¥,Ö8N½)IMJ)>*.Óú0O:ZÕUÀ½÷Þ*lr©FC.»ººÔ¹)  @·¢*Åuy4Ié½÷ÔÇÆÆ&¶Ñ*@Vkxxøþýûêø¹Tï$R:UkjjÌf³Ë½Ô®;wîÔê¨odttô$ÿ­d0uôÓéËªª*m¾×o¾)¥TY[%´j¢µµUDzzäRÆ¬ê^ªNùÑ*@V#PªqªºÜ³gzX+ÙS9t9ÑàT %¥ZVÕi%.^¼8×Ñj__ß4IÈ*·qçÎ¦¦&íêÝ»wív»v2uXjzzº~,[__/WGFF$²ðÊ+ÓÒÒÔMÃÃÃrSppðÄ¬:uJ½ûIw­æçç«sJðãÈ*`,±±±555ÚÕææfñËð×eÀªN(UfHUÈ*dUÈ*d²È*d²È*d²Yd²Yß|óÿÐuë]Ð:IEND®B`


糾正常態 Q-Q 圖


¼WÉg5ÛcÚ,«È*)FlVd5Íá¦tiöýÞýòå½´²¿¿?5&xÍ	Êö§åü6o¾ùæèY9_ssóÝ¡ý®ùÞýr·vs,¤æ'¸1ËåwGÌ²®1µâ4Mó÷:ævÉD¦ýØqiZH/|cùYuÚ	2d<QQo8ÿ­Æ÷Óë5kÖÄ6Q÷´¯8mõÌ3±<öì¢?â3fÄ¥¯§©««»/WIwóÐ¡CÏÉîKz5-§Fßó²ÊC/ú²£¤Át¦­Ù«ùùVä6@ëkkkc9c9Z+·oß^ã¸8p è´Y¹c|Â|gµ`'p6-¯»k×®X(xe1ÖÌ93mßóðãfkdg#Té»ÍgÓGéß F9ò9²ÓÙ(_Ì#Ã)«iZâ®uvv>#­Ibb#Ïfb³4KN/dÆ¤0fÃ÷i'Qñ¢ArÚx¯íl!*:é7[EVy¼<x0MÈÎV¤Ò¤rôÉº;´Ç5½6ÚoyIIt¢à5ÔôÐ¼zõê±ÏVÓr|Å¢ÇÁÆ3èÌ®Ò)¶IX: 7ûÒ3õìÖòï~YµjUÁ¾±]ZpÈRt%Ý(4g÷Æ¡àeÈ¢N;cpf«/Î¶IÑÑÈèÑ¯^tý®îrLãîÌ3'»/1[MëÖ­UdÇHe¯SÏjþýiãÓ§OÇ£|,ìß¿?ú[?$?ß-Ø8Òl5;î)-[MC³×_g5®ßmÜ~|õþµR#ca÷îÝ£ÌVÓ6'NHÓ¬4+M».S"Zi.õþòåËQÁ´Ç8úaùqÜ£yÓ~oÙ±Ti@ò¯.çM<9Ó9f«Ùl8,Y²døgªªª´ýðvN2%ûN2c¼JÑ¬¦ï!¦ÎíííiÐÒnóô²·¬"«<ânß¾ÝÐÐM²i8?±Ëö²¦¬666FwÓ±é±8sKH=ÿüó£ÌVóYmjj*Ø ßgY---Ý¹sgúÞbVzM[fÉ)Þ!¶¼çl5ÝTjCÔ:ÍË·lÙg_~ùåøêé¦Ñ çïiênviÿ*ù¹oÉ4òÙ6éÝM±÷1½b±A<¹I®²à¸§¢kDæ³ý±ñ%â¾Äs ôË~FYËUFÊj]Üêt³éGÆYVUoôi¶:|jöÖ^¼x1p3¾¾¾ìµÉìXÙe5æé`ãtvÞ¼yé¢JÞýõÀñRhã¡<1aJÏ²Ãï9[Móªf¥9k¶>íÅ]·n]<«Ho!Í¿5?éà¦ü±Wém²G>ù§ù)ØsÄ¼9J|7÷êfU§ï$ö]=À8í¥gqË½½½i&ïýÄ®2<«Ùn´RVUkcÉj$$MÒ®ÈCÒ#ovDÏ(YÍ¿¶©2I½1#ò>eûÓîÐ2¦Óa±õÙFïyÈR[[[Üñ4§iþ4¤y[:§à­/÷«à-.éþvÒiÕìÓ'ô¡tòÏHÒ¡U±?7ô	M/¾øâðÏv(ú9»J>«ñü£²²röìÙé¦ø+V¬UdÇW±Õô@aKÈRÜóT4?ù÷­fSÏ¸êêêtûù¿~õà¦,³Ü¦	VÁ<åßTSô6ùuÊöýÆfiÇo<uÈ]Üßßî]|éáG8§Ý³/ñg/	>[ÇÇ`¦uó;ÓRË~6dz©;½f¤AÛ¾iié3â^§×bGÿ1ý*Yþí­_¿^VU_é¸Ó²zýúõLv6Í¦Vcù¥ìhá¥/SÐ¤À¬/øÄÄôX?<«éÓñ½ém¯wÞúßñ3ïØ&fl/NßOÌk#1K»£óï$I9ekN<¹aÃ´w:Û]pÒÁYÙÙËTÐO<[Ëò¿téÒüô1;2<úÿÈï?îÝHª8Þ«¤³fÍºçGígoùYå1¶©S§>ì÷¢àc^ÍÄD¶c²>ÏaøKÔ)êcåß<ö4~s¤ÁLïýn¦i+È*È*È* « « «¬¬¬¬²²²È*È*È*ðèìì¬¬¬ÌÎîÛ·¯ªªÊ°¬ÂvóæÍµk×FÊÊÊ¦LÒÒÒ200ß`É%%%%ÙÆ/¿üòä!±pëÖ­VKþK|oc¼å¸ú-[òg_|ñE?UxÐV®êîîå£GÆr]]]ºèÌ3/.ÖlØ°!ÛÛÛÛÚÚbáÕW_iåÿyc¹åþþþýû÷Ç¥Ó^¼x1~úé®®®yCüAVá)//]¿~½è0âo^:ãÆØ>æÎ;ÒÊ±þÝûyc¹åÃÇ»µ¾¾¾Tâ´fïÞ½S§NõSY¤¶¶6:4öìÞÞÞZZZRÏ²æEÀ²åXH=+ºr¼±ÜrAVc¶sîìÒÊÊÊqeUøJ8Pò¥ô÷÷Pfm+--Íw®¬¬l¤%Å2g-ziÑ[=«Ç?sæLWWWÕòú)¬ÂÓ××Sº,~mmm_=«ãûëýjY^zé¥Hi,;v,m¼fÍX4iÒÂýAVáAëîî3gN©à­)Û	|_f«Ø	ÜÑÑjÚØØÖÈ*È*ü¿188*//©yiRcHvQÑ÷e¶:[.ÕóçÏ_»v-­Y¼x±,È*< ÑS,§ý¨ïøÌ7ïõ×_Oóïx)ºò«d5;;[.ÕüM577û)¬ÂrñâÅÖÖÖ)S¦N:uíÚµ7oÞ©yqÑòåËc:;iÒ¤_~9mYtåÉêXn¹èk«×¯_?pà@öNV?eU`Lzzz²9r$-_»v-ÎÖÖÖ"UUU@V@V@V@VYÿ÷?úôjø¾ûÝïöõõÉêÝ^ziáÂgà+øÁ~ðoÿöo²úË¬>ûì³ÿ¾?üÃ?UY@VeYUdUV@VeYUdUVUYUdUVUY@VedUVUY@VeY¨³zâÄêêê²²²úúúîîîá>|¸¤¤DVÕkiiioo;v,[¶¬àÒÛ·oÏ3'eõÿ÷óÌ3ÏÈ*²ú+wîÜÁÁÁK7oÞ¼eËÕ/¾ø¢¤þð~!Õ_*+++ºÎ?ßØØÑÍvßæ/þâ/ÌVÕ_)--ÍËËËó577=zô÷Êk«ÈêXL6mpp0íå_»3¿NVÕhmmmkk8mii)ºÙ*²:&½½½UUU¥¥¥ÕÕÕÇ/ÚQY@V²*«Èª¬¬Ê*²*«Èª¬¬Ê*²*«Èª¬ «²²*«Èª¬ «²²*«Èª¬ «²¬Ê*Èª¬ «²¬Ê*ðÿÂµk×.`UY¾¨éÒ¥Kxâ3fTWW¿óÎ;ÆYU`ÔÆÆÆ^x!MUñ_DúÓdUVqûà"®Ù(ëôéÓók@VenØ°¡`eLX²*«Àø¼ÿþû/¼ðB~Íxâ	³UdUVq;wîÜôéÓ;::²5Ë/onn62Èª¬ñóÿ¼¢¢"æ¬¯½öZcccSSwÚ «²LÜ§~º÷î7Þxã>¸råAVeYUUY@VeYUdUV@VeYUdUV@VeYUdUVUYYUdUVUYYUdUVUY@VedUVUY@VedUVUY@VeYUUY&èÜ¹sñ°xáÂC¬Ê*0qóçÏ1cFVTT¬Y³æÚµkYU`Ü¢ ëÖ­K)9kÄõoþæo²*«À¸utt455å×|üñÇO<ñ	+iVO8Q]]]VVV__ßÝÝ¿èøñãsæÌb3YûéOºaÃ3fÌ08<Ymiiioo;v,[¶,Q]]Ý±cÇbaïÞ½³fÍU`¸÷ßÿ^È¯¹páÙ*oV+++ïÜ¹555#m6iÒ¤Øì©ªªzægüBÀcëÜ¹sÓ§OïèèÈÖ¬^½º¹¹ÙÈðfµ¬¬¬èr^___ÌJ#«/S__/«ðûðÃczsÖ×^­©©©±±ÑÛlx|³ZZZ-ßàÆ---7oÞ´eÎúÞï½ñÆÿôOÿd÷/uV§M688vÇrÁ¥.]Z¹rååË	¬Þ[kkk[[[,ÄiÌJóõôô,Z´èêÕ«Þ`¬IoooUUUiiiuuõñãÇu7J~yGjjjJrdYõqÈª¬ «²²*«Èª¬ «²²*«Èª¬|SøáMMM'O1cÆªU«>ûì3YU&ØÔwÞyçÚµkË/ohhxË*«²p,0_3þü5²*«ILUþçO4uõêÕ²*«ÛÌ3?ýôÓü¿û»¿Û°a¬Ê*<²âQï7ÞX³fMþçþ§á>ß«ææælÂ¿`ÕÕÕ~ø¡¬Ê*<>úè£'xbùòåÿøÿÓ§OÿùÏnX¸_>ûì³ÆÆÆ¦¦¦wÞy'æ©ÑÔ8ÇAVeÇECCÃÛo¿ýà¢¬.2Ü/W®øÕ«WoØ°á1§Êª¬òùôÓOzê©#J/^ü³ýÌà¬Ê*Ï/~ñ¦¦¦Ë/ÿý÷Èª¬Âø|öÙgù5¯R]]ýÿñdUVaÜ^íµ?þ8þøÿø^0, «²´nÝº³Î1ã'X½zuLX	Èª¬ÂWSÕcYUdUVUYYUdUVUY@VeUVUY@VeYUUY@VeYUdUVeYUdUVUYYUdUVUY@VedUVUY@VedUVUY@VeYUUY@VeYÑ7=zàÀ[·nÉ*²:ÑÑmÛ¶Í9sÅûöí³û÷ïÓ¥KvuuíÞ½;<(«Èê²ºdÉ´ÜÑÑ1cÆ´sÖ;wî|968|ø°¬ «cÊêúõë+**bºqãÆI&Å$5VvwwÇÂo¾Ë/^UduLY]¸paZ>þ|*h¬Ü´iSLXzzbùôéÓ²¬/«±ÜØØöîÝGå³gÏÊ*²:¦¬7¾þúëi!­ìïïïêêJ+oÜ¸!«Èê²ÚÔÔT0sS§NÝ¼yóàÁ^[@VÇ¤³³3ª9yòä!±<uêÔ´uëÖ¥éì7ðÍ¬²À7+«/_¾sçNz;M:$8wÍ±cÇÆ~;'N¨®®.++«¯¯ïîîý¢­U¾éYMnÞ¼¦¤ÙÊóçÏÇÛ3gîy---ííí±°cÇeË~ÑÄÖôó'ò'¿ÿû¿ÿs	yï½÷þôOÿôé§~æg~òÇÖ÷¿ÿýûÕ'OF>/_^ôÒôÚjMMÍè7RYYf½¿hk¾øâøTTTÜ·¬ÖÖÖÎ7oômN:µÿþÑ·ImÑå¢MlÍÿ7Ì~ô£ü'ÀøÍ5kýúõÙÙ)S¦üó?ÿ³yýà?øÆýÒÒÒl¹¼¼|ô&¶Æk«ÀýòÙgM>ýÚµkùkÖ¬yûí·×VïCVûúúiÒñ¥´¼gÏ#Çò(·0mÚ´ÁÁÁ´Ï6G¿hbkd¸Y­®®.ÈêºuëdUVïOVoß¾^ÈÌ¿uëÖ­c¿ÖÖÖ¶¶¶XÓ¿hbkd¸vïÞ½páB¶§§ÇÈÈê×²x¼Yííí­ªª*--ßËãÇg7Rô¢­Uà>VTT¼ñÆüñÏ~ö³¨ìêÕ«¬~S²êã ±¬K.ê©§.öÛoìFV¿RVïÜ¹)]»víð¬¦×8eYà5½'²eYxVí@VedUVUY@VÇÕk×®É*²zonnnNGü666fGÿfËmmmé?ÄÉ*²z·nÝºûpU¾qYxÉ*²*«<êY½råJgggúÂ3gÎ5+laIIÉ#GdYcÇEDÏ;WYY9wîÜt<ptËlYß[k:::ÊÊÊâtÒ¤I[·nÝ¾úæÚºº:Y@VïáüùóÕ'NÞÝ%KÈ*²zï©êbá£>jhhhmm]>¤««kòäÉ¯¼òÊàààÙ³gí@VÇ÷¹Ø|A½¶¬~¥¬.Y²¤eHú¬¥+WÊ*²:î]Á¯¾újvvvN<yêÔ©]]]qv÷îÝ÷|åUVÕÂ¬¾õÖ[iyÆsæÌÉ_qUduYÍÚYUUÕØØ¿hïÞ½²¬Nd¶ÄìÙ³Ó+¬ýýý²¬#«Y½sçN¬7oCÕ	2eÊ²eË|Ô>²ê?Ø¬Ê*LVKJJ^|ñÅìl¬éééUdu|.]ºíëëËW6­ «ã088¸aÃåË§÷ÒìÝ»7ý÷òì#óYY@VKo¡Ù·o_6IMÿÌ|ÅùëóÏ?/«Èê^UÍ>D)ÓG_¼x1¿ÁÖ­[eYwVkkk¯^½·nÝÊVnß¾]VÕ1eµ®®.ýßò4[M+«ªª²¶mÛ&«Èê¸g«© ±|üøñ´róæÍ²¬N0«áöíÛÙÊ-[¶È*²:ñ¬ýç6²¬ãµÕ#Õ¼oõÝwßM«ªªÚÛÛ×®][ð~Y@V|F5O:Î~þùçùKwíÚßE,«Èªÿ`<«;wîÌÚ~ö²«¬ «ãöùçç?øæÍqv×®]ÃwË*²:âË«stuuUWWG«ºDS[[[c¡½½=çÍ'«Èê.8içÎ^[@V'âêÕ«ÄÜtóæÍé¬¦w²¦©êÆeYÇÿ3ÏSVíÚ5Y@VïíèÑ£Ëd´ÓÖå_5öì¥¥¥²¬u¦ªsçÎõ¾Udõ«¾ÁæìÙ³Ù?UduâÒ»T:ÔÕÕßYÊ>4XVÕ15µ­­íÎXsøðáXsæÌ³Udu®=vìX~eAV³i.«ÈênÝºù¬.Y²$;xãÆ±~ÕªUÙáÁAVÕ±~aþ³²¬úÇpðÈê)S~úéa:::JJJöìÙ#«ÈêXå?^?R:cÆìÃ"â¢ÎÎNY@VÇÕHiÇW^yeÒ¤IqËûöíN>-«Èêý­8pàëèâ'ª««ËÊÊêëë»»»ó?~|Î9qQCCCl&«<dY5kVúæë×¯<yò«¯¾ËqÑ'|òudµ¥¥¥½½=vìØ±lÙ²üEuuué­´÷îoLVxÈ²zñâÅ8î¹ç²5-ºûöþýû¿¦½¸é]=1'®©©i³I&ÅfÿgÚÚÚgyÆ/ß¬¬8p ;èwÚ´iéÖX:uj,lÛ¶-E÷¾gµ¬¬¬èr^___ÌJ#«?æÉ'U¾qY½uëV6CmnnÎ,Yòµsÿ®åååÃ7¸qãFôþæÍvðÐd5¿+øëÎjÉb9fÆé¿½Æi,lyéÒ¥+W^¾|ÙÀ<4Yíëë[2d¤Àiyþüù÷=±­­­mmm±§1+Í_ÔÓÓ³hÑ¢«W¯zSV³O¾yóæªU«¢ çÏ³'O<tèÐ×ñj¦···ªªª´´´ºº:û÷éi"[SSS#«<YÕÝ»w§Ãó¸ÅÝ¹sg¬ollôqÈê½uuuÕÖÖ¦±EÝ¸q#ÊºbÅY@VýUY@VeYUdUV@VeYUdUV@VeYUdUVUYYUdUVUYYUdUVUY@VedUVUY@VedUVUY@VeYUUY@VeYUUY@VeYUdUV@VeYUdUV@VeYUdUVUYYUdUVUY@VeUVUY@VeYUUY@VeYUdUVeYUdUVUYYUdUVUY@VedõîÝ'NTWWÕ××wwwßàðáÃ%%%²¬Þ[KKK,ìØ±cÙ²eÞ¾Î9YVÿk?û³?UdõW*++ïÜ¹555nÞ¼yË-)«_|ñEI1?üáýB «¿TVVVt9?¾±±1¢ÍV?æùç7[@V¥´´4[.//Ï_ÔÜÜ|ôèÑ_Þ+¯­ «#ÉößÆò´iÓÓNàX.ºY¶±¬ «£immmkk8mii)Ãf«Èê½õööVUUVWW?~¼hGeu¼®ò×ý×12«V­ú÷ÿwð¸dÕÇAÜwÿõ_ÿõÛ¿ýÛßþö·ÿàþàw~çw¾õ­oýä'?1,²*«1sæÌ¨iLXÓÙhjÕ@Veu"~ã7~ãÔ©Sù5QÙU«VYÕñ ¬?þýÑYÕqûÖ·¾ÕÙÙ_SQQñWõWF@VeuÜb@¾ýíoÇïÇÿ:$8¦ª¿õ[¿uáÂ# «²:ßûÞ÷JJJ¦Lò¿ùßùÎw<hLdUV'îÔ©Sï¼óNüd «²¬Ê*²*«Èª¬¬Ê*²*«Èª¬¬Ê*²*«Èª¬ «²²*«Èª¬ «²²*«Èª¬ «²¬Ê*Èª¬ «²¬Ê*Èª¬ «²¬Ê*²*« «²¬Ê*²*« «²¬Ê*²*«Èª¬¬Ê*²*«Èª¬ «²*«Èª¬ «²¬Ê*Èª¬ «²¬Ê*²*«²¬Ê*²*«Èª¬¬Ê*²*«Èª¬ «²*«Èª¬ «÷Ù'ª««ËÊÊêëë»»»óÝºukåÊåååµµµ===²¬ÞCKKK,ìØ±cÙ²eù6nÜøæoÞ¹s':sæLY@Vï¡²²2Â555ùbþzêÔ©ìllöÒ0³fÍzægüB «¿TVVVt9Ý¶mÛ¤IbªÚßßYfï~÷»²¬þJiii¶^^pÑîÝ»cáôéÓv «E|)§M688vÇr~³üÙ¬¬ «E´¶¶¶µµÅB¶´´ä/Z³fÍ¾ûbáäÉMMM²¬ÞCoooUUUiiiuuõñãÇ³él^¿~½¹¹9æ©gÎUdÕÇA «²¬Ê*Èª¬ «²¬Ê*Èª¬ «²¬Ê*²*« «²¬Ê*²*« «¿þÏã¾óïÌºfÎùäOÎbdµµµ¿÷¿gFòÔSOÅÅ©¡IüþÄQÄ£P<Qüîïþîý¬Èê/ìº¯êêê.­Ò8dãÆ%%%6m2#ùó?ÿóòòrã0Ä½qÅ3öÙûxï½÷ÞÿüÏÿÈêý¿Ík×®5£4iÒûï¿oFrêÔ©ÈêHÿ!_130£øË¿üËEQ|ÿûßíµ×ÀUYUYUYUYUYUYEVeUVeYUYUdUVeUVeUVeõë÷£ýhÓ¦MÆaõõõÿú¯ÿjF200ðäOþ÷ÿ·¡É¿üË¿|ïß3£øÛ¿ýÛ+VQ477oÝºUVàa"« « «ãÇÏ3§¬¬¬¡¡áÄ±&N«««cMww·!:yòäÜ¹sÓôöö¢¢>ò«?@ãSàòåË%9h¸[·n­²¼¼¼¶¶¶§§ÇÉ[Niié"Y·ºººcÇÅÂÞ½gÍ---ííí±°cÇeË¢ø=pà@,Ä@UVV¢ánß¾OÎ²¬ÑüCT`ãÆo¾ùæ;w¢©3gÎ4D£a1y`C$«_É¤Iâ4Ê¿Ü±088XSScX29½!nóæÍ[¶lÉ²j|DSÓ3³!þäõÔ©SèÎ;·`Á9D²:q/½ôR,e+óËùllÊ)Ó¢çÏoll?ï,«Æ§@]]Ý¢Eb(b N>mAØ¶m[<³©j¿!Isss<V?È?4Y 7n´´´Ü¼y3Ó^û¤¼¼Üàd><mÚ4C4üïüèÑ£w^ûIkÏH.]ºvx¢1 »wïxÚO>QQgÎyúé§óöHV'ø§¾råÊË/§³QÁÁÁ´c!U§è×þê~ñ]zø3Dòà¯l$7n9~ÐÀÉê¸õôô,Z´èêÕ«ÙÖÖÖ¶¶¶XÓÂ¢ººº'OÞ:j: !©¯~Fú:ölzëW¨¨5kÖìÛ·ïîÐ÷MMM¨¨yóæôÑGø±ZVÇ­¦¦¦`ªÑÛÛ[UUUZZZ]]!1D'N¨¯¯gÐ,HszC4zVO¾¾¾Ù³gÇ¯Ðüùó£¬h¸ë×¯777§ÓJ¢¢»:Ò1JòMV@V@V@VYYYYdddUU`9RRRrëÖ­8Ý¼ys¬:ujGGGKKKÆú7ø¸PRþ)Ê(Ò¿a_¿~:óæÍ8þ(È*ð¸ûvWW×ë¯¿Û·o_nÚ´)N+**zzzòáÜµkWvvöìÙsçÎ]>$®~àÀØ`É%Ù·Þz+Ö~=»Jooo¬ÉþHÊjgg§²<Rbzøðá6N:t÷Ëÿ­f=þ|½xñb.X° ­ÌÿGátõuëÖ¬úÆjnnÜ³gO¬yöÙgcyçÎ±¼cÇ?dxèÕÕÕíß¿Ê)Q¸Èd[[ÛÖ­[£s1ãÌGqïÞ½i¹ºº:¦¤)®q­ÆBLFëëëÓTµ½½=Ö|òÉ'ÙÕÓÜôäÉð´æÆ±&*küUàÑú«.)y÷ÝwÓÜ4½ü9uÈ¦M¢©ùÙjv­ôoÕûûûcá£>Óôÿ±caîÜ¹ùS§ãvb9xûöí±&»ñèº²<<888EL/ ^¹råÚµk©yEg«éìÑ£GkjjÒ¾Üp¬<útUUÕ²eËb!ûqãÏ=÷o®ÛÜÜè¸?dxt&¬×üº±¼téÒ¢YvöõõÅK.¥E³VÓîß³gÏÜøÉ'ãôÈ#Ù47Í³ÙjLvýUàQ^%Ýºuë)SvïÞæeee3gÎìøR~'pÌ>SO:§±Y×tPRZ¨¬¬RÞ¹s'n<óa.:[ÍOAVØüã¶8M³Ï8=qâDZM<9;Ú(IMâÜZ¤ôòåËé áÛ·oo§Ù*È*<²®rõêÕôþ8MG¥W:SÛÛÛ«««®ÞáÚÚÚÕ1ßÈººº"f« «ð8Hï~9wî¶µµeëKKKçÍ¥LYK#´iáðáÃi&Úßß§1gMzcª:âcÙ*È*<Ò[HÓ<5®]»6íâFöR>hðúõë)Ò,«éc%º»»Ç;[=yòä(IY/^<xð`vöÒ¥K===Ù9¤·¥.]º4?íìì³×®]ÆÆµµµ/N]¹r%.:uêð¬îÙ³'ýÛÌV_~ùåô~È*ðx5kVvöÐ¡CÄÓß	kúXÄ¨²))È*È*È*È* « « «¬¬¬¬²²²È*È*È*<êþÍÒÈú IEND®B`


ú¬N7âÛo¿äFuww«§Ú­'²üÇ?þqÑ_RnuÈÔÔTÏ× ï_òòåKùäÙú­Ö®]k¨iGGºhãÆü¯¬bá»uë;zdµålooïÙ³g#""ÔÃýÃÕúK.É©L[gþRbíXQå«®®özCVå¬aþZRR"ëøáýWId¥Ê©ú=åtÃrMYøê«¯´+Ê@"'§===r*7ÿñ¯Qï#·%<<§Z322"­=tèúÑrº÷îéX¨Ù¹,tNW¯^®vÿþY/ã,KU¨Ô3!yZ E/,,ùÛ<èoû~z#¿º-'O4ÜL¹WUU,jB¦I´fi©iÕjÕZòãO[e½6áËåQ;66V&Aµ9Wuh×®]Ú'??ßð+íÜ¹S»HYÍöÔ÷´X,?«¿¿_NÕu>ªª$ÏW²ª«:uêÇ©¹2KöËÍÊÊ³j°üªÚÓY¯ÍVÕ4õæÍò#dún_ýµáæ¯_¿^®,(7DâËY¹ÚÍ_µjº¾|7U5àJºd-©ÑÿÕä"ucT­ÃÂÂäË¥»2n² ggØæ?Û/1dUlÕéÈ¹	ddæ>çQÁ²*áQßÿ½¾%2ËS5-SÁ¡¬réwß§-'%%©ÇeyìÖ_çË/¿n¶ªæúGyÃOTíTËjÚª%P&y2)fTT×_OeUÈÍÈMÛ»w¯ÌÕäfê³*sGµýS&Á*ØrY)I¤6EÓogV·´µµõµO?ýT;+ã,7_Uª¢¢B.'Cyyy*·òÔDn»þ5c=uµÓ§OëWª?<ñúÓgû%ú¬j[ ´aT[É*È*)Jl+YUs8ÃNMÕ¶ßzyOmìééQ¯Õ^µ´íÆêAYo¿ývæ¬Ê/;;ûÇ©í®úþøÓfmfYPW0ì/#	ÔOpe)%Óoi]ej+WS5iÖßjÛI&e©¶cË¥jA½jøÅô³jµØð2¤<QQ_Xÿ«Êï#Óë¢¢"¹Ô]m+Ö^[üø±,'$$xýÇÄÄÈ¥WÇÕÄÅÅ½/Q7óÚµkLÑnzU-«fÞòUÌÒ?UÉE=Ê¤SmhÕ^XÕÏ·$·jZY+Ëòø+ËÒYyòäIõ§Äµ¡¡Áë´¬ÊÜQ~	¡¾ÁY5lÖ¦ÅòsÏ=+WeÃáP×ßÙs¿Ùè)ÚY	úmµËYµçzÅW&Rýç7nh»,ÉtVÊ'³FÉ°ÊªV¹iõõõ3ÃtkXkûHË³¹%«2eR(3BÏmÚTÜënP3ì¤6Û/Ñ66Èyô3[YÅâÒÔÔ¤&d^g«ª4ªmS´Ý~Úâª^Hé^n2I'¯¡ªæÂÂBßg«jY~¢×ý`åô^fWj$×IÚ¡WûÑºöÝôï~)((0¼è+Ú¥]¤+êHéÕ¤Y¸O7!½Xm|Á1ÌV7mÚ¤]G&ý^·H#¥÷^º×õsøueÒ,7gÕªUÚmÙªZ())!« «XDÔHÒ3«ú÷wª+?|øPåeáòåËj×ßÄ)úù®a+ât³Um¿'YÞºu«6Õ^õÌª|ü¶òýå§Ëhæ¥)UUU3ÌVÕuºººÔ4KÍJÕ¦KÕk½ª-ÆÒo	Ìå&öæUoFòú»iûR©Ñ¿º¬*RËjÆ,³Um6,233=ß;«DEE©ë¶3,,LûM4>~×¬ªßA¦ÎN§SÚl®^ö&« «Xà&&&´	6Ó6ë'vÚVVÕäädé®Ú3V=konQ©Ï>ûlÙª>«©©©+¨õÛl%«f³ùôéÓêwYÚëU]SKzºækg«ê[©6H­Õ¼üèÑ£r¶¸¸X~ºz§©tE&èú[ªº«O5ÕÿýÜWË¤yí:êÝM²,·Q½l1¹<¹QîÜ¹Ó°ß×ãZ(ym¬ü¹-òHýá´¿!«¾|ÉtY¡[!C­¾­ú¨q&« «Xøoôn¶ê9µU[kÕG2³éììÔ^Ôö[Ve^¨v6Vg×¯_¯v ©äÿÿF`ùA*´òP®ÿ2aRÏ´Ý_;[Uó*f©9«¶^mÅ-))gê-¤ú÷¡êPíÜ¤ß÷J½MöÆ# * ÃEæÍRâu¯nªYµúM¤ÁjÛµ×ÕVzy6 ß¹½½]ÍDÏÞÏíK<³ªmÆP+É*È*5_²*	Q$µ)rtzäÕöè!«ú×Võ/U*êøGÚ3dA¿ë¶ÝXm)£z¹Wíkx¬×Þ3úÚ]ª««å« <EPóWõ¤AÍÛÔþ8·¾ná-.êó|;©á0YÕ>¡¨7©[¤F¢v­ùCMw¦üü|Ïc;x=NäÜ¾DUyþ þ¸j¿cÇ²²ÅKÍØ¦Ëªz TÅ°©h)UîúÃ'yÍþ«ÚÔS¾ÝnWß_ð[Ï/	®zÁRË­`ð¤S×7Øèê¤mû«©¿òÔA¿wqOOºuò£=÷pVg/ñi/	Ï<[ËË`ªuõÕTË½R½Ô­Þ3Ý <y211Ñl6ÇÄÄÈ­V¯ÅÎügòýKYõüõöîÝKVAV±x©ýN§ËêË/%0ÚYy47L­|9Ê¶·°¡Cjg%¯ûË$8ÃzÃÕc½gVÕÕþ½êm¯?N½õS¿áWfÞr±mÚ´Iý>2¯@Ê<LmÖ¿DåD¤­éíí---U[§µMÙ vÎÒÎªXªx,ßMËVV~ú¨Uùóôä÷[7ÝQgû%jCÅ+^¨í-¿YÅ""¶ðððù~+6¼þª?ÜH¶e²®çàùµº3ý;O£ÿP©Þ=óÍTÓV¬@V «¬@V «¬@V «U@V «U@V «Uó@DDvöÒ¥KQQQ@Vwmlll÷îÝÒ$ÅÓßß¯¿Bff¦ÉdÒ®\:E^½z5ÝJ_ôôôlÜ¸1$$$((hÇò<7¿³üGÕÍÏÏçUà]Û¹s§D¨µµUoÞ¼)Ëqqqê¢ÇoÚ´É4E­)--e§ÓY]]-_õÕt+(Aøðaww·|á®]»Wðå;K/_¾,^»vMNeaíÚµë§ðWÈ*ðHØ$B/_¾ô:8é³ªÎÊõeaÍ5Ó­õ?°É$ÓVÃJ_¾sss³ÌeµoÒÙÙ©J¬ÖÔÔÔóWÈ*ðÄÆÆJÚÛÛåää¨iYiË² zæuå¬ÜºuK¾ÐóÕP_¾³!«2[9·viDDÄÜ2¬sÑÐÐ`úÉzzz<çZÛÌf³¾seº&o¼þcccqqqréÙ³gyýÎ3gµ££ãñãÇZVeÊË_ «À»ÓÙÙ)S:-~ÕÕÕoUßeggËWúé§ùUõº¬¤Tdâ«®$!!!éééü²¼k­­­«V­òÜ;·À>ÎVO:%ëív»Ëåò¼tëêêTMÕ²Uà1>>..«jR;:EÛÈëJ_ÈÏ(znyáÇùÕÁÁA·Û­ÖlÚ´¿,@Vw$))IÚ#qeµÕðOV¿þúkµXÿ¯+±k×.¹~MMñù§èËwöUý·ÊÎÎæ¯UàÎÍÍ3Íááá»wï6AU¹(//O¦!!!ÅÅÅê^Wú"""ÂëVbmÙïìõµÕ/_644hïdå¯U>ioookkÓz|ãÆµìv»ålll,CUÈ*dUÈ*d²º`ýû¿ÿdd¤7ðávvvÕ?¡&==ý1oà7¿ùÍý×Õ?gõO>ù_ÞÀoû[²JVd¬È*YU²Y%«²JVd¬È*Y%«²JVd¬È*Y¬UY%«²JVd¬rU² «d@VÉ*d¬È*YU² «d²JVd¬È*Y¬UY%«²JVd¬@VÉ*¬UY%«U² «d@VÉ*¬UÈ*YU² «d²JVd¬È*YU²Y%«²JVd¬@VÉ*¬UY%«²JV «d@VÉ*¬UY%«d@VÉ*¬UY%«U² «d@VÉ*¬U²ü*++·lÙòùç×ÖÖ2 «dÀÜ­rÉ%«W¯þè£øYõ#]]]v»Ýb±$&&¶¶¶ê/êèèXµj$W#«?ÈÏÏêééQgåÈl6ïÛ·Yõ999N§SN:µuëVýEqqq·nÝ+VÈÂ¨ù!«À»ôÁ|óÍ7ú5Û·oÿVFdÕ/DDDLNNÊÂøøxttôtW		ùÓþdòæ÷¿ÿ=wà	niiÑ¯)//·ÙlÈª_°X,^õ:;;wíÚ%­233­ïÝnÏÏÏ×¯IKKKLLdd@VýÙlÖ<¯0::366Æk«?¨¬¬¨¨¨Pg÷íÛ'gëëëU¿°téÒññqµX>ölçÎ###ì	øIéûï¿,ÏËËËU[]]-r*³RýEmmm7n|ñâo°üÍÐÐÓé¬­­u¹Èªioo'¼v»½££ã/7Ãôç­ß5¬È*U² «d²úËgõÑ£GÉÉÉ)))%%%Ï?ç~­¯¯oË-üqSS²JVßZVG~~þÝ»woß¾-¥¬XÀêëëÍf³ºÛËSÉÃ!	²JVç®¨¨ÈpàrWÃB,SUílEEÅ%KdþÊÈ¼.«²²²°°°´´´­­!«.«òl]æ©ú5W®9+w5,HòðñÞïÞrj³Ù°¾<§æRÖX­VY`XÈ*Y%«X°NgXXa¥ÃáØ³góæÒÒÒµ³÷îÝíîîfdÈ*ÙéÉ'ò ¢­Z²dImm-óÌûf;vÁ!«ì²Ä.KXÈ3ªàà`µp__ß²eË/_Î°¼9yÜ1¬,--åÈÉdu1¾ÁF²$Aå6X~÷»ßÉ500PNãââäÉ%còVÈsô«W¯jgÝn·aÈ*¬;wîp¸ü·«®®Îf³]¼xQ*Ï×³³³³²²d!«dæâúõë)))¡¡¡êSßyâBVÉ*¬UÈ*YU² «d²JVd¬È*YU²óËåâø©d¬ÀêîîNKK´Ûí111I¬U£Øl¶#G¨×ÕÕIZZ²JV`Öõk;ÁÈU²,^.kß¾òYPPpçÎÄwYYY­¾2gdÈ*Y)éhpp°ÕjMKK[¶lY@@ÓédX|$SÕÊÊJý©lzz:#CVÉ*°HIPSRR´³_|ñE``à'O_~=&&æÞ½êìóçÏe0;ÆÈU²,F===QÃgUTT08>*//·ÙlEEE¥¥¥#//Ï?'«dX¤N§DÔ°RÚ sVÇw2[­¬¬¾^½z¦U²,^úÝdæj±XÎ;Çà¬U,|ÂÛõÑGÉµ©©IU6..î>0lÈ*YÅâv»KJJl6[LLzMmoÑï~÷;³Êé²eËU²,///==ýÑ£G²,§iii¥¥¥ËÛuçÎ&© «dß½÷ìv»þ8æýýýV«#d¬UÌZmmí¶mÛ+eÂ*ÿQ@VÉ*YÅì´´´HD+ö|d¬¾r¹uuuÚóçÏÇÇÇ³×@VÉ*YÅLù¬¬¬,,,,--mkk3LXm6æÌY¾û6#U²JVáÝóçÏSSS¥¬´ÛíGÑ_áÑ£G²F*>xðÈ*Y%«VQQQvv¶¶]WÂÉÇA «dsäp8ÔÛR52+õýÍ©MMMÛ·oß²eKyy9	U²ÅÎjµvA:sæLaa¡/_»yóæÄÄÄuëÖY,¨¨(U²E-55U:ª_qâÄ×~¡LOÕ!mÕf³­^½!È*YÅâÕÒÒ)e9kAAARR/ÇQIêöíÛõk$±2geH²JV±ØËj·ÛóóóÜõÍ7ßVL&Æ «dµÕSôk¤²AAA@VÉ*09î%K¶oß®f·åååå/¾`d²JV¹¨¯¯·Z­æ)%%%	@VÉ*ðFd¶Ú××Ç8d¬@VÉ*¬UY%«U² «d@VÉ*¬UÈ*YU²àç544tìØ±ÂÂÂÜ½Y%«æèöíÛv»Û¶m'N(--µÙl©È*Yà«ªê+ÚÝÝÍÈ¬U³sïÞ½øøx·Û­_YPP -@VÉ*È¬455Õ°²´´´¼¼ÁY%«fÇårY­Vý&_Yãp8®^½Êà¬U³vâÄ	»Ý®:zïÞ½M6eee6d¬bþ¼+W¾ÿþû¿úÕ¯ÒÒÒwãâÅIIIÁÁÁ2O-..~þü9c²JV1¿µ´´,Y²dÝºuMMMçÎ³ÙlQQQ.È*YfM"úñÇë×È´õ/¾`d²JVY©j__~Í-[V¯^ÍÈøN¹6oÞ|àÀ&úXàY½yófCCÉdzõêY<³úäÉýÏ?ÿ¬ú®¼¼<  `ùòåiii2Ñ3<MHV¥£cÇ.]³/_Ó¬¬¬ÆÆÆªª*Ynjj"«XäØü&zzzÌfó¾û´5òc·Û,Ì¬fffªåºººµ,sÖÉÉÉ¹Bss3YÅ"Ç.KobÏ=2OÕ¯©ªL^@,Ì¬îÝ»×jµÊ$µ¬¬,$$D&©²²µµU¾ýö[Y&«üãÅÅÅË<UúÊl|·yóf1ÃÊÀÀÀ;wî08XYMOOWËª ²òÐ¡C2amkkåUs¶oß¾°°0ýô3[ÅÂÏª,'''«Y¸qã,?ú¬3É§ÌòSRRTGe*3~ÃkÕÀÂÉª(++ûúë¯ÕZÙÓÓÓØØ¨VUoB¶¤¬V«Uæ©ZbÕÔÔTÃÌUúúúÆÆÆxmÀÛÒÒÒrîÜ9Ã[Õúúz©fhhhÝYWÒWY())QÓY?|3+YøWVGFF&''ÕÛiÔ.ÁÚ»knÝºÅá duÖÆÆÆÔ´¿¿_[988(k,ËãÇÉ*¬ú¤··WòçõRõÚjtt4YÕ×]¿~ýÌ×éëë»|ù2YU>ÁwÕÎÎÎÌ)9Sê~¢/`2d¬ÈêëMLL¨ýõof=~ü8³UüâäîÎÑwÌ§¬z=FYÅ/KR)§V«5??ÿùóç²JV1Û¶mS)ÄÊY)+Ã`~durrRRº÷nÏ¬¿öË»ººìv»ÅbILLlmmù¢¹­!«ÊÝ»wÛíÖO^eÎ:00Àà³UJeYÂÔ.Kjyæ¯k:NY8uêÔÖ­[g¾hnkÈêär¹*++KKKÛÚÚ´õµµµyyy+§¥¥é¯ãÎ;·gÏ9å òY5fuV#""ÔNO2µ5;Âó¢9¬å=d"ûáæaÞúÇüÇ¿ú«¿?âo~ó>úèý÷ßONNVeddüÍßüÍöíÛµ+Ë²³Ï>óÏÛ"¿°Åb1Íaaaæ)²?10/ÄÆÆú]VåÅë²×æ°F²ú[QQQòÈÿ¶víÚ¥KÉßQþ^ÿ÷¯]d³Ù¤©ÚÙ¹ôUÿáþA"*÷uYPgívû_ÿõ_ûçm~ý%K$¨² ÖËí,&D~Uyn®-Ë#ãÌÍmç£çÏÇÇÇ¹#GHJïÝ»'¹ÝîÈÈHÃÎ½,--UË<¯MJJç#==½¿¿ßo·ý6üÊîçU¨=äTg¾hnkÈê|tàÀmÛ¶é×H5Õ	­L@×?sæ4X;+émkk»xñ¢Üãõ»/ù	¿a¥¬ùâ/¸dÕKVåmæ¯ÍÍÍ­®®95Éó¢¹­!«óüí®^½ª_#SUªeÉêõë×õfdd8qbÞÝÌÚÚZÅ¢­Ê²¬Y,÷`Ñeµ¹¹9;;[íñ¬íý«-KØÔ'ÄÍðMÚÛÛ£¢¢Ìf³<PvtthmözÑÜÖÕùH&¦ýÛ·oË]K-?^þ¾2çm=*((âÎÓc>Øl¶åË?yòDMÄW®ÎþÀÀbÌê«W¯8Ô>~&gÎIIIÑRÃhge¶*W¾æççÏßJPå&,Y²Äjµ|ðÁ===ÜÅU>Á?«¬¬,	§ÌY¯!SÕ|BIieeå;wø»d¬âgáv»¥49992O=xð Çõ°(²êr¹êëëÕÎ·cÅj/ÉtãÆ² «³sëÖ-èÀÀ@DDÄ5kÔ>GÒ-f«²:»·Ö¤¦¦ÖÕÕY,9		±Z­Ç?yò¤ú<s	m\YÕ×¬vuuÍÜÝÌÌL² «¯ªnØ°Aº»»rssÕ¡CCC¿üòËñññ§O²@VgwI¬¾ ¼¶Àÿ¹õ|c~ÕÌÌÌ)êXK;wî$«üVýï½æp8£¢¢æïAE° ²*ýê«¯äTî£¡¡¡ááár¶ªªêµ¯¼U¿'OX,Ï?ÿ¶.[¶,11_dõ»ï¾SË111«V­Ò_$q%«üÍ=>øàý;wîp4føEVµvFEE%''ë/ª©©!«üÍæÍÓÒÒ+9~$ük¶ª"!!A½ÂÚÓÓCVøálÕf³1[fµ¾¾ÞÕÉÉIY¿~ýzvYà<y"sS^[?fU/,,lëÖ­jÿùÅbQËÃ³Ê'ØGdZYY¹gÏ§ÓÉh¬U,|22ÍV«õÜ¹s	°À³j2òóóµ³===²¦­­¬o>³Ûíò+ËÛ·o¸ÊìlV=&íììÔWV0[U<xP\!Ï<®Â]³RPP YÕï*e5°³:>>^ZZjµZóòòÔijjjÔ§GGGk0ÔOd[V¯^½¹ÿþë×¯8qÂf³1ÏÀ¬¤¤¤HGóW°22ÀÌªzÍ¥K´Iªú0ó;vèg®öÙ¢ÍªÝn¿xñ¢v¶»»;44t``;"|´nÝ:Ãµ§§¬3«(É²:ððð°þÇ_YUgX%xîðQyyy`` ¤T[³zõêeË12À¢Èjllì/dáÕ«WÚÊ'O.Ú¬&''VªmãÜá»ÄÄÄ÷Þ¯  ào¾Y¾|9G×xVãââÔç«ÙªZ¥]¡¢¢bqfÕívÛl¶mÍ£G¬Vë¸#bVòóó%¨òoµnÝ:ýÌUq¹ïÞ§qrc¬5[UíïïåµòðáÃöµÕÚÚZ)ë3g$¥W®rä÷B¼EjW¸ÔÔT¹w©÷á0&ÀBËªÐV=zt1¿Á¦­­-+++>>>==]¹â-ºxñbLLÌSÕÙóçÏKYÙ'XYõúá6x»¯_¿®_Ç`~gUÿÚjCCç;píÀÀÏÍjµÖ9s¦  æeV=ß·úý÷ß«-ÀQQQN§s÷îÝ÷ÛUà-JHH¸wï~Miié`^fUò)ÕìëëSgøáý¥gÏÕo"&«À[·ÿþ´´4íÐXÍvûömFYål_ÛíÎÉÉq82I-,,=sæÃ,¬>Z´íeW²üÜZZZÊËË;v÷î]FX YýáôG³gÏõÜ8LVduÚW×Lill´ÛíÒªÆ)ÒÔÜÜ:²¼~ýz² «>ílØéôéÓ¼¶ «sñâÅSdnzøðaõ«ê¬jªZVVFVdug®í¦$­2v»É*üâø<ÏêÍ7·NÑ´$ÓÖ¼È.ÈJ³ÙLVàræÌ»Ýi³Ù6mÚÔßßÏøiVµyªª®Y³÷­¿55&&¦­­í§>F°´´4>>þùóç?fUóôéSíÃàÈ*øÏOÌÎÎæµü:«ê]ª×®]kllÔoV»,i&«ðIV=ç¯fU5µººzr¬inn5?f¶¿,·Ûm³Ù$®ú(--epü1«.KzëÖ-ýJCVµ4'«ðîeeeé?.P$CKK#ãwYõêäs||©í&ëå¯¨í,È*ü"dª 3Tyd¶Z­|$ÿÎVµù¨þXKì²þæÊ+G9qâá3záYåádõ­e5,,líÚµ9êêêL&ÓÈ*¬úJxIiLLv°¹¨¾¾¬Èê,²*)­òå_È©,_ºtI.zøð!YÕ·3[mhh «²:¬®X±BùÞ½CCC¿úê+Y®¯¯îß¿OVduYÓO?ýT[³qãÆË/³'0¬úª¡¡AÛéwéÒ¥êVY]² «>kI¡fggkË¼o@Vç¾)¬ÈêÜ³ÚÙÙ9eºÀj9--¬Èª¯G+((ÊÙÞÞÞk×®ùáKªdà¿YÕªª*µ°þûRÜÓ§OËúääd² «¯×ØØ[]]=ÝFGG¥¬;vì «²Ê'Ø@VÉ*¬UY%«²JV «d@VÉ*¬UÈ*YU² «d@VÉ*d¬È*YU²Y%«²JVd¬ÎÛíâ®	d¬¾þþþììlÍc·Û+++¹Y%«sár¹âããÕTõöíÛWÊd¬Î4##C¿FÊ*sVî£@VÉê¬yÎMeÂ:00ÀÝÈ*Yìß¿_¿ÆårÙl69ånd¬Îü¬Vkww·~þÎÈ*YóçÏËô4//O¦­ÉÉÉ©©©l²JVçîÑ£GUUUG¹rå¬UY%«d@VÉ*¬¾]]]v»Ýb±$&&¶¶¶ê/êèèXµj$W#«²ú999N§SN:µuëVýEqqq·nÝ+VÈËÃ?ýÓ?UYýÉÉIYîj!!!úÓLÞüþ÷¿ç «f±X¼.ëuvvîÚµKþÍ73[Õ¿0ÍÚrPPçFGGsrrÆÆÆxm@V½Ð¶ßÊòÒ¥KÇÇÇÕF`Y6Ù³g;wîaO`Y½ÜÜÜêêjYSê/jkkÛ¸qã/x¬ú¤½½=**Êl6Ûíöm:+§ÑÑÑú]È*¬r8Y%«²JV «d@VÉ*¬UÈ*YU² «dõç¶gÏ÷ß?  Àb±¤¤¤qç²JVçB~óÀÀÀo¾ùÆår555Ù¦È2÷o «duvdb*Ôúúzý³Je¹Y%«³ãt:ÃÂÂ+×­[ÇKÅ@VÉê¬Éßï½÷Þ3lò]¹råöíÛ¹Y%«³¼eËýü5   ©©û7U²:kõõõK,Y¾|y~~þºuë¤©²ÀÈ*Y£¾¾¾Í7¯òã?Ù*÷l «d@VÉ*d¬È*YU² «d²JVd¬È*Y¬UY%«²JVd¬@VÉ*¬UY%«²JVÉ*¬UY%«²JV «d@VÉ*¬UY%«d@VÉ*¬UY%«U² «d@VÉ*¬UÈ*YU² «d²JVd¬È*YU²ºàÔÕÕdee<zôû=U²:GG±Ûí×¯_/..ìîîæ®d¬ÎÚ½÷¤£rª­9vìXJJw «duÖdZXX¨_ãv»m6ÛóçÏ¹÷Y%«³sâÄÒÒRÃJ»Ý>44Ä½È*YÃár¹´5W¯^¬r×²JVç"'''--M½¼ZWWg³Ùjkk¹ëY%«s!SÕââb³¦¤¤HY¹ßY%«?+W®Hnûûûù²JVçîØ±c6-LgËËËù¯²JVçBf¨111Ú»Z<xàp8.^¼È?U²:kçÏ×¯¦nÚ´ «duÖdª_#gãããõkä.Â¬Õ×ÏV|kkkõ³Õ¢¢¢ÐÐÐÔÔÔCd¬þ?3¿¶zäÈÎªyªÛíÞ¿¿Lde «dÕ»cÇY­Vµ'°ÍfjÑ/ÒÒÒØ¡	È*YÉÀÀ@Ýý¨CCC25&¬äßÈ*Y5¼SUUÅÈY%«³¥¦ÿüùóZv	²JVçB*YÜ¶mÚXî+U²:w·oßyjKK|d¬È*YU² «d¬È*YU² «d²JVd¬È*YU²JVd¬È*YU²ÀÏjWWÝn·X,­­­Whnn6Ld@V_/''ÇétÊÂ©S§¶nÝj¸tbbbÕªUd@V199)ãããÑÑÑK>|ôèQU¹Ú.+V¬øÃþÀ@VÿÌb±x]ÉÉÉRS-«ððáUYý³Ù¬-é/ÊÎÎ¾yóæodu:¦ÈòÒ¥KÇÇÇÕF`Yöz5íÊd@Vg[]]-r3]­Èêëµ··GEEÍf»ÝÞÑÑáµ£d@V9¬UY%«U² «d@VÉ*d¬È*YU² «d²JVd¬È*Y¬z(**úÕ¯~µâíq8¿þõ¯W`z±±±÷wÇ8L'>>^HNéÈýGq<Écã0¿ýÛ¿»Õ²úgýýýgßª¸¸¸ôôô³ÅbÙ¹s'ã0²²2ÉtèÐ!b:ÿüÏÿÄ8Ì`Ãò@Ï8Ì &&æO>yßðüùóÿó?ÿCVß>¹7ïÞ½qAHHÈÅéôõõIV?~ÌPLG¿dfÀ8Ìà_ÿõ_7nÜÈ8Ì`õêÕû÷ï?¬U²JVÉ*Y%«d¬U²JVAVÉ*Y%« «d¬UU²JVÉ*Y%«dõç·ûöC13HLLüÏÿüOÆa:ýýý¿þõ¯ÿû¿ÿ¡ÎüÇ|ôÑGÃþíßþmÇÃ²³³?NVOÈ*d²ºôöö®Y³Æb±$&&¶··Ë®®.»Ý®Ö´¶¶2DJss³Éô;C¤722bÒa|<½zõjçÎAAA±±±mmmÇn³ÙÌyNBBÂ»¬&«³&Y¸uëVDD,äää8NY8uêÔÖ­["111±jÕ*-«^]]4C¿ñ1(++ûöÛo'''åÑáp0D3a1a<Éãóàà ,Èittô;"²:w×®]KJJR<ùÿññqõÇÃáÃ=ªe!Ò¦ªgfúÿÆÇðäµ¯¯!z­60D^É²gÏÉª'gïfÈê§baaaÒ9k±X´ôË<7LNN»¯UH/..nãÆ22J>d|<É TTTÈ£aOOC4ìììÎÎNþË¼êêê»<É©¥w3Dduî.]*ê%((õ7oþ8õòZÃy%O¢ÕÆÇ@¤ªªJäi<ù`¼züøñÚµkõÆé­Y³Fmó¾ªz7CDVßô9µJÇÇÕÚEÎôÿcf þ·ý ð_6²²2Óë!ò||~÷÷"²:kqqq½½½²ÐÑÑ¡[]]-rÃéûª"Ã]èéÓ§j¶Ê]È«¢¢¢K.ý8µãjj*CäÕúõë»»»µ³ÌPÕcõýû÷eæúÎ¬ÎZWWWbb¢<÷Ù°aÃÈÈ¬ioo2Ív»]ZËyf!ÒëììTûý§¥¥©]*/_fgg«ÕÑ"¯:Ô8üyÕßß¯Þ)§²üÎ¬@V «U@V «UÈ* «UÈ* «UstãÆÉôêÕ+9=|ø°¬	OLL¬««ËÉÉSY_VV6íãÉ¤>7fêcê÷îÝ«ÎÉYõ!©Y°@LLL466~ýõ×¹K.Éé¡CäÔjµ¶µµéÃyöìYílBBÂ5kò¦È744È233µ5ß÷¬yùò¥ö%ííí²FûÕúúzþ «¤677Kä$±rzíÚµúlyýlU«ìàà Ó6¨/^¼0Ì_KJJk¤¾ò²³³å^¸pAÖ|òÉ'²|úôiY>uêUó^\ÜåËÃÂÂ¤pÉêêêãÇKçdÆ©bMMZ¶Ûí2%Uq¯¹©,Èd411QMUN§¬¹ÿ¾öåjnÚÛÛ+	WkFGGeTñY°°þ«M¦ï¿ÿ^ÍMÕËáS:$T¥ÔÏVµ¯R;ßÓÓ#ÝÝÝrª>BÖ¬Y£¿²ê´|YV%>yò¤¬Ñ¾¹t?È* ©©i||¨^@u¹·[5ÏëlU½yóftt´Ú+>Úºu«,è¿¿|óO?ýTm7V_m(´|þ «ÎUâªßSW³²²¼fUÚÙÙÙ)k=¦^Õ^XU>jøæ½½½rzãÆm«æÁÚlU&»ü@V,êUÒãÇUUU©¹£Åbq8u?ÑoÙ§ªc__ÊÕ§¨ÔBDDrrrR¾¹,ëÃìu¶ªdÀ<öÇ?þQMNÕì³¿¿_N»ººÔ¹(44TÛÛHQMâ¾¤tddDí$<11áÙNf«Y,ËõâÅõþ9U©W:UN§Ýn7|zknn®VG#ãââ¼<v0[È*°¨w¿Èiuuµ¶Þl6¯_¿^J©²*JhÕBss³öôôÈ©ÌYÕ^ªNûØÁl «Àb ÞBªæ©êt÷îÝj°W²§rh8ÐàË/U %¥ZVÕa%Z[[g;[ííí!ÉY0o755ig=ÖÖÖ¦ÌA½-5++K?­¯¯³n·[r(WÝ´iºÈårÉEáááY½páÚûI®o­«cJðçY°¸¬X±Âétjg¯]»f8¿LVuXD©2SR¬@V «U@V «U@V «UÈ* «UÈ* «UÈ*ÝÿÖ6Ý©ñ*IEND®B`


ûöÓ¤Óé;v8Ø¬Å¬Ëåæææ¦¦¦p;11f¢(ª­&ÇîÜ¹sx_~ÙÙ*k4«[¶lªöµ««+R©Tm5z&7®³ä"0²ºÄ¹i6­T*Õ+½aÜ¸Î¬ «óáurr2.^¼Î ¿¿dd$Âm±XLnÕùË/óÔpÆa&<ÄJ¥â8.J=Ä¦¦û-É*²êí UYYYUdUVUYYUdUVUYYYUdUVUYYUdUVUY@VedUVUY@VedUVUY@VeYUUY@VeYUUY@VeYUdUV@VeYUdUV@V@VeYUdUV@VeYUdUV@V@VeYUdUV@VeYUdUV@V@VeYUdUV@VeYU`iZIv¯¬Ê*@½:uÊ~UYUd@VYõYM¾rJ¥ÂL¹ã8¢B¡0>>Ü¸Î¬²¬þÅèèèððpÅ0p·¯¯/¹M%YdYýÌ+W^yåê8ÉÌÍÍA¥RÉçóÉÍ,ñ¿.²qãÆUÖnV'&&ªã(jóÉñâ¥Õ=´´´È* «¬Ý¬^ºt©«««v·úkU:NnYgÉE`@VÕ»öïß?44T»Íf+JõJo'·¬³$«¬"«wmÛ¶íÂµ»ýýý###anÅbrË:K²È*²úÙåÜê"UËåR©TÇ¥R©ö8÷[U@VUoÈª¬Êª¬È*² «Èª¬²¬Ê*¬Êª¬È*²*«¬"«²È*²*«²¬È*²*«¬"«² «²*«²¬Ê* «Èª¬²¬Ê*¬Êª¬È*²*«¬"«² «²*«²¬Ê* «Èª¬²¬Ê*¬Êª¬È*²*«¬"«² «²*«²¬Ê* «Èª¬²¬Ê*¬Êª¬È*²*«¬"«² «²*«|Î?¿Åüä'?±UdUV×ßÿþ÷M+fëÖ­ö0²¬Ê*KÛ·o_øc? «Èª¬"« «²*«È*È*²*«²²¬Ê*²¬Êª¬Ê*²²¬"« «Èª¬Ê*È*²*«È*Èª¬Ê*²²¬Êª¬¬"«²¬"«Èª¬"« «È*²²¬Êª¬¬"«²¬¬Êª¬"« «Èª¬Ê*È*²*«È*²¬Ê*²²*«²¬¬"«²*« «Èª¬"« «²úÝ¼ys`` NoÚ´éôéÓa¦ÇqEQ¡POndYß¿ÿÁçææBS7oÞfÅâèèh÷õõ%7^°tçÎÿëÖ­===¶¬¬²³Î;§¦¦3L&T6*J>¯³tûöíEÖ¯_/«²²ÊÍjECCCÍÍÍáTõý÷ß¯Î$Wl|¿%edYO¥RG	>ø ³³³:S[M§Ó6¾ß¬Ê*È*²:ÍfJ¥R½Ò¿$«²²¬Î;v,&''_zé¥0èïï	p[,×YUYYEVç?ùäÞÞÞpÚÙÙyéÒ¥0b.K¥RqJ¥ÏbSÓýdUVAVUo¬ò¸ùåm[¶|uÛ¶¯­ÄÇºuOttVèoÜø¬Ã'«²¬òxI§ÿîà=£¿y£á>üUYEVyì<ûl<~êàÔn¸ÿdUVUdUVeUVUdUVeUVUUdUVedUVeUVUdUVeUVUdUVUYUUdUVUdUVeUVUdUVeUVUUdUVedUVeUVUdUVeUVUdUVUddYUdYUYUdYUYUddYUYYUYUdYUYUdYUdYYEVeUVS@VeUVeYEVeUVeYYEVeUVAVeUVeYEVeUVeYEVeYEVAVUYUUYUY]®]»öß+fpp0|SX¹¯åÊGYEVeõ1rêÔ©ðï6ÿ¿Ùì_|j¾xxØ;ÿå5GYEVeõñÊê×ºøíæà=ßùÎ¿9È*²*«²*«Èª¬Êª¬Êª¬"«²*«²¬"«²¬Êª¬Ê*²*«²*«²*«Èª¬Êª¬Êª¬"«²*«²¬¬"«²*«²¬ÊêêÎj8~²*«²¬Êª¬þ³:;;ßîÝ»ôÓOeUVeYUY]®ã·gÏâ=ïÞS¥YUYEVeUVìÊ+áàýáH^þéOê"°¬Ê*²*«²ú·G..xµÕ°¬Êª¬"«²*«ËýI¥?ü0ãÎÎÎÚßê/Á¯~õ«0~óÍ7eUVeYUYð©ê­[·ªqmkksXVeYUYý~dIVeUVUYÕÏ3«É_Z]ÕpFû_aUYUdUV/«7oÞìêê:qâD8xë×¯Ý»wWïùÝ÷TïacYUYEVeUVPÖ%O^]UYEVeUV?²*«²¬Êª¬Ê*²²ÊcÕYUYEVeUV¦0S.ã8¢¨P('7®³$«²*«Èª¬®¬ã÷ýïÁÌk¯½¶Ï÷Ýw¼±Xááá¾¾¾úKgÙ¹s§¬>¬¦R©¦Æ´sçk Ë±~ýÃ×YGî>·cccÉßU3ù|~9_!4õøñãÉL&SýU×J¥²à,XºûöO¦;v8Ø!«ûíæßüº#Èr¤ÓiYåagurr2Ü>|÷Ão¼&O8ÑÜÜþc³³³ü---ÝÝÝQuvvB0®­&ÇK.ýÏ"ßýîw­>¬ærOoý§îã¹M¹]»¾í²Ï<óô?¶?×ÏsYmà¬NMMÛwÞy'9yàÀë×¯A©TZænooT*Uÿ«Ü¦Î×V~V½¶ÊªçµU^VÃyaõïÕTÿRM¸ýõ¯]ýÛ«áö½÷ÞÛTÂËüÕRf³ÙJ¥R½ÒÆÉê,Éª¬Ê*²*«¶Z¥cÇ]½zµvªÆG¿×_Áß9_|øÃ?¬­vwwAõWtÂm±XLnUYmì¬^¾|¹Òr¹|æÌO?ý´Ö×ê¬þÙjøÄ¶¶¶(Âe3á³r¹ã¸v¹ú»7K.Éª¬Ê*²*««!«á$uãÆ/^ÇïÜ¹sáö[ßúÖÔÔÔÎN8Q½,rëí dUVUYÕgÕW?úè£êÏs¸V*Ú_[ð§XeUVeYUY­÷N¡?øÁÂøÈ#á(<xðÚµk¡²a¦µµõ¥^òæ²*«Èª¬Êêr³ú»ßý.ù5½½½Õùl6¿)UYUdUVeu	ÃÃÃáÈmØ°á¿üåñãÇÃøõ×_¯.J¥pwppPVeUVUYÕe¹råÊäädòðoÜ¸Q»ûÞïãºä:UYUdUVeµ1Èª¬Ê*²*«²*«²*«Èª¬Êª¬Êª¬"«²*«²¬¬"«²*«²¬Êª¬Êª¬Ê*²*«²*«È*²*«Èª¬Êª¬"«Èª¬Êª¬"«²*«²*«²¬Êª¬Ê*²²¬Êª¬Ê*²*«²*«²*«Èª¬Êª¬"«Èª¬"«Èª¬"«Èª¬Êª¬"«²*«²*«²¬Êª¬Ê*²²¬Êª¬Ê*²*«²*«²*«Èª¬Êª¬"«Èª¬"«Èª¬"«Èª¬Êª¬"«²*«²*«²¬Êª¬Ê*²²¬Êª¬Ê*²*«²*«²*«Èª¬Êª¬"«Èª¬"«Èª¬"«Èª¬Êª¬"«²*«²*«²¬Êª¬Ê*²²¬Êª¬Ê*²*«²*«²*«Èª¬Êª¬"«Èª¬"«Èª¬"«Èª¬Êª¬ò¨|éK_ýÍ¡¬÷!«²*«²*«<v²ÙgBYÃ9ëJ|<ñD:f¾¸¬Êª¬Êª¬²¶òGö¬Ê*²²¬"«²¬"«²*«²¬"«²*«²²*«²¬¬"«²*«²¬"«²*«²¬"«²*«²²*«²¬Ê*²¬Êª¬Ê*²¬Êª¬Ê*Èª¬Ê*²²Jgull,<Ãªãr¹ÇqEBa||<¹Y%YUYEVÕ»nÝºÕÑÑQËj±Xááá¾¾¾än.ò½ïOVeUVUÖnVß~ûíCÕ²ÉdæææÂ R©äóùänß¾Ý´;v8Ø²*«È*k1«W¯^íìì±¬e5¢Újr¼xéÎ;'éééq¶*«²¬²F³ÚÛÛæÌ»âÏYM¥RµÕt:Ü¸Î×VeUVUdu~ÁõÛ0Íf+JõJo'7®³$«²*«È*²úW­úûûGFFÂ ÜÅä6udUVeYEVÈjx¹JÅq«K.Éª¬Ê*²¬z;YUdUVeUVUUdYUdYUYUdYUYUUYUddYUYUdYUYUdYUddYEVeYEVeUVeYEVeUVedUVeYYEVeUVeYEVeUVeYEVeUVedUVeYUdYUYUdYUYUUYUddYUYUdYUYUdYUYUUYUddYUYUdYUYUUYUêdõëýÍ÷ÑûíÞµ«×DVUY?þÙgóÛ¶m%>^xaÓºuO¬Ðoyaó[oý»#¬"«²ºVìÛ·/|Ç±UdUVUUYUddYUYYEVeYEVeUVeYYEVUUdUVedYUddUVeYYEVeUVAVUYEVUdUVUUdYYEVeUVAVUYEVAVeUVUUdUVedYUdYEVeYYUYEVAVUYUUdUVUUYUddYUYYEVeYEVUYEVAVeUVUUdUVedY­krrrË-Qê+ËqWgÆÇÇ×YUYYEVçC ?çÎËd2aP,GGGÃ`xx¸¯¯/¹q%YUUdõ/N<ÙÞÞ!®sssaP©Tòù|rKaüÕE~úé[VáråØ½²ºnÝZ·n]xÒ=z4Ü¢¨¶/^ºsçÎ,Ê*«²D±XÜ¾bì^YýÛe³Ù0H¥RµÉt:Ü¦ÎÀ² «óOFCJõJo5´5udUVdu¾¥¥err2J¥Rwwwô÷÷A¸-Éë,Éª¬ÈêÝß)á<õW^	3á!ær¹T*Çqhíg±©é~K²*«²êí U@VeYUd@VeUVdUVU@VeYUYUYUdUYEVdYUYUYUdUYEVdUVcúÓN-[x¦¬.û.ØÃ¬Êêò³ý¬i%ÙÃ¬Ê*²*« « «²¬Ê*²*« «²¬Ê*²*«Èª¬¬Ê*²*«Èª¬¬Ê*²*«Èª¬ «²²*«Èª¬ «²²*«Èª¬ «²¬Ê*Èª¬ «²¬Ê*È*Èª¬ «²¬Ê*Èª¬ «²¬Ê*È*Èª¬ «²¬Ê*Èª¬ «²¬Ê*È*¬¬J¥(ÚÛÛËår	·qB¡0>>Ü¸Î¬ «ó---çÎ£G¶¶¶A±Xááá¾¾¾äÆudYý+ÍÍÍá6ÉÌÍÍA¥RÉçóÉ,ñà"_ùÊWzzzlÖtV'&&ÂfDQTL/¬~/ùË²ÀÎê7Åâììl§R©Ú|:NnVgÉE`dõ®éééêÝl6[©TªWzÃ8¹e%Y@VçO>ÝÝÝýúõÚLÿÈÈHÛpÜ¸Î¬ «óù|¾)!ÌËåR©TÇ¥Ré³xÿ%Y@V½²*« « «²¬Ê*²*« «²¬Ê*²*« « «²¬Ê*²*« «²¬Ê*²*«ÈêjÏêàààSO=ÕJ#áî9ûUìÅ_Ü´iSKK]ÑÐBnVV/_¾|·k×®L&c?°ýüç?ojjzóÍ7íöÛßþöã?^åYe8tèÐæÍíV±k×®¬þñ´+V%YEVAVUddYYEVeUVAVAVUÍ#G¾ñoØ¬büñóÏ?áÂ»BVYYY¿Áõë×óùüâùr¹ÇqEBa||Ü¢AJ¥ðLnooÏjOrYtöìÙ¶¶¶¦¦%Åbqtt4ûúúì+TKKË¹sçÂàèÑ£­­­ä²+èÕW_½téÒYÍd2sssaP©T<ÓÜÜìI.«°òÏÂ¥²EÑchP÷îõ$Ux4YM¥Rµq:¶hh7nÜ(³³³ä²&«Ùl¶R©Ìß»>Æökzzz```ffÆáeµ¿¿dd$Âmøß|uúôéîîîë×¯Ë*<¬Vï=6Ë¥R©8K¥½DÊçóM	ä²È*È*È* « « « «¬¬¬¬²²¬±±±íÛ·§ïéèèª¿ýììl&Iþ¡'O¶µµEQÔÚÚÆdV§Ã7-ràÀûmëÖ­ÞÞÞä,J¥RµÏãÉÉÉ:ó¬ÂªµaÃÐ¼3gÎÌÝóÎ;ï»ë×¯_rã÷ß¿³³sÁßÝ»woÿèG?Å·afêÌ²«ÖO>÷_üâæÍþÇÏ±cÇYmmmã0·aÜÒÒRgUXµÞzë­j#×­[÷ãÿø£>ª³qWWW©Tªõµ:EQòuÖ0nnn®3È*¬fû÷ïÁ«½:888;;»ÓÖÅãêÝÔ:ó¬Â*:zèÐ¡êë¬ÁÀÀ@2éldx°O>ù$Ä5Ù¿åduóæÍa<===ÿç×P[[[ëÌ²«VÇ!xG©þ$ðÉ'ÃÝl6ûï¬îÙ³'øÃqò'~ï7È*¬Zo¿ýöâß[=|øðò³zñâÅäçÖ~?õ~ó¬Âj644ÔÞÞÝÓÕÕ566öàïY8|J¡PálmmM~úýæYYYYdddUUUU@V@V@VYYYYdªÿè.¢ª»>EIEND®B`


T-TEST GROUPS=S100(1 2)
  /MISSING=ANALYSIS
  /VARIABLES=年龄
  /CRITERIA=CI(.95).


T 檢定


附註	
已建立輸出	15-MAY-2019 19:05:26	
備註		
輸入	作用中資料集	数据集3	
	過濾器	<無>	
	粗細	<無>	
	分割檔案	<無>	
	工作資料檔案中的 N 列	75	
遺漏值處理	遺漏的定義	使用者定義的遺漏值會被視為遺漏。	
	已使用觀察值	每一個分析的統計資量是根據觀察值，該觀察值對於該分析中的任何變數沒有遺漏或超出範圍的資料	
語法	T-TEST GROUPS=S100(1 2)
  /MISSING=ANALYSIS
  /VARIABLES=年龄
  /CRITERIA=CI(.95).	
資源	處理器時間	00:00:00.00	
	經歷時間	00:00:00.00	


群組統計資料	
	S-100	N	平均數	標準偏差	標準錯誤平均值	
年龄	1.0	7	45.571	14.1405	5.3446	
	2.0	36	50.056	11.4466	1.9078	


獨立樣本檢定	
	Levene 的變異數相等測試	針對平均值是否相等的 t 測試	
	F	顯著性	T	df	顯著性 （雙尾）	
						
年龄	採用相等變異數	.594	.445	-.914	41	.366	
	不採用相等變異數			-.790	7.605	.453	

獨立樣本檢定	
	針對平均值是否相等的 t 測試	
	平均差異	標準誤差	95% 差異數的信賴區間	
			下限	上限	
年龄	採用相等變異數	-4.4841	4.9070	-14.3940	5.4258	
	不採用相等變異數	-4.4841	5.6749	-17.6896	8.7213	

CROSSTABS
  /TABLES=S100 BY 性别
  /FORMAT=AVALUE TABLES
  /STATISTICS=CHISQ CORR KAPPA
  /CELLS=COUNT EXPECTED COLUMN
  /COUNT ROUND CELL
  /METHOD=EXACT TIMER(5).


交叉表


附註	
已建立輸出	15-MAY-2019 19:06:25	
備註		
輸入	作用中資料集	数据集3	
	過濾器	<無>	
	粗細	<無>	
	分割檔案	<無>	
	工作資料檔案中的 N 列	75	
遺漏值處理	遺漏的定義	使用者定義的遺漏值會被視為遺漏。	
	已使用觀察值	每一個表格的統計資料都以每一個表格中，所有變數指定範圍中具有有效資料的所有觀察值為基礎。	
語法	CROSSTABS
  /TABLES=S100 BY 性别
  /FORMAT=AVALUE TABLES
  /STATISTICS=CHISQ CORR KAPPA
  /CELLS=COUNT EXPECTED COLUMN
  /COUNT ROUND CELL
  /METHOD=EXACT TIMER(5).	
資源	處理器時間	00:00:00.03	
	經歷時間	00:00:00.04	
	要求的維度	2	
	可用的資料格	131029	
	精確統計資料的時間	0:00:00.00	


警告	
CORR 統計資料只可用於數值資料。	


觀察值處理摘要	
	觀察值	
	有效	遺漏	總計	
	N	百分比	N	百分比	N	百分比	
S-100 * 性别	43	57.3%	32	42.7%	75	100.0%	


S-100*性别 交叉列表	
	性别	總計	
	男	女		
S-100	1.0	計數	4	3	7	
		預期計數	3.7	3.3	7.0	
		性别 內的 %	17.4%	15.0%	16.3%	
	2.0	計數	19	17	36	
		預期計數	19.3	16.7	36.0	
		性别 內的 %	82.6%	85.0%	83.7%	
總計	計數	23	20	43	
	預期計數	23.0	20.0	43.0	
	性别 內的 %	100.0%	100.0%	100.0%	


卡方測試	
	數值	df	漸近顯著性 （2 端）	精確顯著性（2 端）	精確顯著性（1 端）	
皮爾森 (Pearson) 卡方	.045a	1	.832	1.000	.582	
持續更正b	.000	1	1.000			
概似比	.045	1	.832	1.000	.582	
費雪 (Fisher) 確切檢定				1.000	.582	
有效觀察值個數	43					

a. 2 資料格 (50.0%) 預期計數小於 5。預期的計數下限為 3.26。	
b. 只針對 2x2 表格進行計算	


對稱的測量a	
	數值	
合約的測量	卡帕 (Kappa)	.b	
有效觀察值個數	43	

a. 相關性統計資料只可用於數值資料。	
b. 無法計算卡帕 (Kappa) 統計資料。它需要雙向表格，其中的變數具有相同的類型。	
