# Supplementary material for: High-order radiomics features based on T2 FLAIR MRI predict multiple glioma immunohistochemical features: A more precise and personalized gliomas management
Source: PLoS One. 2020 Jan 22;15(1):e0227703. doi: 10.1371/journal.pone.0227703 (PMC6975558; doi:10.1371/journal.pone.0227703)
Supplement: S3 File — (ZIP) [file pone.0227703.s021.zip › statistical analysis/vimentin/HL.doc]

GET DATA /TYPE=XLSX
  /FILE='C:\project\hebeishengerglioma\数据分析\T2 vimentin\3.xlsx'
  /SHEET=name '3'
  /CELLRANGE=full
  /READNAMES=on
  /ASSUMEDSTRWIDTH=32767.
EXECUTE.
DATASET NAME 数据集4 WINDOW=FRONT.
LOGISTIC REGRESSION VARIABLES Label
  /METHOD=ENTER ShortRunEmphasis_angle135_offset4 RunLengthNonuniformity_AllDirection_offset4_SD GLCMEntropy_angle45_offset1
  /SAVE=PRED
  /PRINT=GOODFIT CI(95)
  /CRITERIA=PIN(0.95) POUT(0.99) ITERATE(20) CUT(0.5).


羅吉斯迴歸


附註	
已建立輸出	16-MAY-2019 14:24:12	
備註		
輸入	作用中資料集	数据集4	
	過濾器	<無>	
	粗細	<無>	
	分割檔案	<無>	
	工作資料檔案中的 N 列	42	
遺漏值處理	遺漏的定義	將使用者定義的遺漏值視為遺漏	
語法	LOGISTIC REGRESSION VARIABLES Label
  /METHOD=ENTER ShortRunEmphasis_angle135_offset4 RunLengthNonuniformity_AllDirection_offset4_SD GLCMEntropy_angle45_offset1
  /SAVE=PRED
  /PRINT=GOODFIT CI(95)
  /CRITERIA=PIN(0.95) POUT(0.99) ITERATE(20) CUT(0.5).	
資源	處理器時間	00:00:00.00	
	經歷時間	00:00:00.03	
已建立或修改變數	PRE_1	預測機率	


[数据集4] 


觀察值處理摘要	
未加權的觀察值a	N	百分比	
選取的觀察值	包含在分析中	42	100.0	
	遺漏觀察值	0	.0	
	總計	42	100.0	
未選取的觀察值	0	.0	
總計	42	100.0	

a. 如果加權有效，請參閱分類表以取得觀察值的總數。	


應變數編碼	
原始值	內部值	
0	0	
1	1	


區塊 0：開始區塊


分類表a,b	
	觀察值	預測值	
		Label	正確百分比	
		0	1		
步驟 0	Label	0	0	18	.0	
		1	0	24	100.0	
	整體百分比			57.1	

a. 常數包含在模型中。	
b. 分割值為 .500	


方程式中的變數	
	B	S.E.	Wald	df	顯著性	Exp(B)	
步驟 0	常數	.288	.312	.851	1	.356	1.333	


未在方程式中的變數	
	分數	df	顯著性	
步驟 0	變數	ShortRunEmphasis_angle135_offset4	5.222	1	.022	
		RunLengthNonuniformity_AllDirection_offset4_SD	5.037	1	.025	
		GLCMEntropy_angle45_offset1	6.224	1	.013	
	整體統計資料	12.100	3	.007	


區塊 1：方法 = 輸入


模型係數的 Omnibus 測試	
	卡方	df	顯著性	
步驟 1	步驟	20.328	3	.000	
	區塊	20.328	3	.000	
	模型	20.328	3	.000	


模型摘要	
步驟	-2 對數概似	Cox & Snell R 平方	Nagelkerke R 平方	
1	37.037a	.384	.515	

a. 估計在疊代號 9 處終止，因為參數估計的變更小於 .001。	


Hosmer 與 Lemeshow 測試	
步驟	卡方	df	顯著性	
1	6.833	8	.555	


適用於 Hosmer 與 Lemeshow 測試的列聯表格	
	Label = 0	Label = 1	總計	
	觀察值	期望	觀察值	期望		
步驟 1	1	4	3.529	0	.471	4	
	2	2	3.090	2	.910	4	
	3	3	2.808	1	1.192	4	
	4	4	2.467	0	1.533	4	
	5	1	2.237	3	1.763	4	
	6	2	1.825	2	2.175	4	
	7	1	1.372	3	2.628	4	
	8	1	.614	3	3.386	4	
	9	0	.058	5	4.942	5	
	10	0	.000	5	5.000	5	


分類表a	
	觀察值	預測值	
		Label	正確百分比	
		0	1		
步驟 1	Label	0	14	4	77.8	
		1	7	17	70.8	
	整體百分比			73.8	

a. 分割值為 .500	


方程式中的變數	
	B	S.E.	Wald	df	顯著性	
						
步驟 1a	ShortRunEmphasis_angle135_offset4	-9.020	7.971	1.280	1	.258	
	RunLengthNonuniformity_AllDirection_offset4_SD	1.137	1.192	.910	1	.340	
	GLCMEntropy_angle45_offset1	-1.947	.881	4.884	1	.027	
	常數	4.276	3.194	1.793	1	.181	

方程式中的變數	
	Exp(B)	95% EXP(B) 之信賴區間	
		下限	上限	
步驟 1a	ShortRunEmphasis_angle135_offset4	.000	.000	737.148	
	RunLengthNonuniformity_AllDirection_offset4_SD	3.116	.302	32.199	
	GLCMEntropy_angle45_offset1	.143	.025	.802	
	常數	71.943			

a. 步驟 1 上輸入的變數：[%1:, 1:	
