# Supplementary material for: High-order radiomics features based on T2 FLAIR MRI predict multiple glioma immunohistochemical features: A more precise and personalized gliomas management
Source: PLoS One. 2020 Jan 22;15(1):e0227703. doi: 10.1371/journal.pone.0227703 (PMC6975558; doi:10.1371/journal.pone.0227703)
Supplement: S3 File — (ZIP) [file pone.0227703.s021.zip › statistical analysis/vimentin/spss clinical.doc]

GET DATA /TYPE=XLSX
  /FILE='C:\project\hebeishengerglioma\数据分析\T2 vimentin\T2FLAIR2018.12免疫组化数据 - 副本(1).xlsx'
  /SHEET=name 'Sheet1'
  /CELLRANGE=full
  /READNAMES=on
  /ASSUMEDSTRWIDTH=32767.
EXECUTE.
DATASET NAME 数据集2 WINDOW=FRONT.
DATASET CLOSE 数据集1.
EXAMINE VARIABLES=年龄 BY Vimentin
  /PLOT BOXPLOT HISTOGRAM NPPLOT
  /COMPARE GROUPS
  /STATISTICS DESCRIPTIVES
  /CINTERVAL 95
  /MISSING LISTWISE
  /NOTOTAL.


探索


附註	
已建立輸出	15-MAY-2019 18:59:11	
備註		
輸入	作用中資料集	数据集2	
	過濾器	<無>	
	粗細	<無>	
	分割檔案	<無>	
	工作資料檔案中的 N 列	76	
遺漏值處理	遺漏的定義	應變數的使用者定義遺漏值視為遺漏。	
	已使用觀察值	統計資料是根據所使用任何應變數或係數沒有遺漏值的觀察值。	
語法	EXAMINE VARIABLES=年龄 BY Vimentin
  /PLOT BOXPLOT HISTOGRAM NPPLOT
  /COMPARE GROUPS
  /STATISTICS DESCRIPTIVES
  /CINTERVAL 95
  /MISSING LISTWISE
  /NOTOTAL.	
資源	處理器時間	00:00:01.31	
	經歷時間	00:00:01.28	


Vimentin 


觀察值處理摘要	
	Vimentin	觀察值	
		有效	遺漏	總計	
		N	百分比	N	百分比	N	百分比	
年龄	1.0	6	100.0%	0	0.0%	6	100.0%	
	2.0	38	100.0%	0	0.0%	38	100.0%	


描述性統計資料	
	Vimentin	統計資料	標準錯誤	
年龄	1.0	平均數	47.167	3.1981	
		95% 平均數的信賴區間	下限	38.946		
			上限	55.388		
		5% 修整的平均值	47.241		
		中位數	48.000		
		變異數	61.367		
		標準偏差	7.8337		
		最小值	35.0		
		最大值	58.0		
		範圍	23.0		
		內四分位距	11.8		
		偏斜度	-.327	.845	
		峰度	.492	1.741	
	2.0	平均數	49.447	2.3868	
		95% 平均數的信賴區間	下限	44.611		
			上限	54.283		
		5% 修整的平均值	50.053		
		中位數	50.500		
		變異數	216.470		
		標準偏差	14.7129		
		最小值	7.0		
		最大值	72.0		
		範圍	65.0		
		內四分位距	20.8		
		偏斜度	-.640	.383	
		峰度	.362	.750	


常態檢定	
	Vimentin	Kolmogorov-Smirnova	Shapiro-Wilk	
		統計資料	df	顯著性	統計資料	df	顯著性	
年龄	1.0	.146	6	.200*	.985	6	.975	
	2.0	.092	38	.200*	.961	38	.203	

*. 這是 true 顯著的下限。	
a. Lilliefors 顯著更正	


年龄


直方圖


Ù£é5ÀXV±U¬bu¶õöö.[¶,~SSÓÑ£Gcþ©S§bNww7VU¬b«XU>ãðÈ#÷î]´hÑ±cÇâpûöív«XÅ*V±ú;w.@J/nkk+++Kû/_Þ6^p+VU¬b«X¶ööö óøñãi´úì³ÏnÚ´ixx8~úé§±ÀØØXZÀhUXÅ*V±ÕYVÓ3©ÁjEEÅªU«ZZZbNZ §§'NÊ~÷*VU¬b«X¶ô9ÙÀ555Û¶mëêêJþ°téÒòòrÏ­«XÅ*V±:[VâðèÑ£i'pvßoú ÃþðXV±U¬bõ!<xpïÞ½é³	c¨ï¾ûî?_sõêÕ´ø¡ÕÛÛ»jÕª¢¢¢êêêéþ¼°U¬b«ã£ÕôJ¥¾víZ zøðáÜ§]gù6Ð4éáÂÒÒÒø¿Â*V±U¬j.³ÞZ¾²&wíÚ=iß¾1ç>x¤<sæÌÊ+ïß¿ª×_HXÅ*V°_þòqòÒKeùúKûÛ¿ý[¬*¬¦FGGo¿ýv@sÒUÓøuß·²páÂôáqôOjÃF«XÅ*Vórû>óLÉg?=¯ú?ùîï½UåÕôê;bzÑ¢Emmm+V¬Ó©.]JâÞºuk6vöìÙ%Kxn«XÅêStûÆÆbUy`õîÝ»ÕÕÕYAüËw¹¨gÎýÅa«XÅ*V5w?y½½½Æ¿½®®«XÅ*V±*¬>~ÝÝÝ1ðqêºuë¦Û]U¬b«XVóV±U¬bUXÅª°U¬b«XV±U¬«Â*V±Ua«XÅ*V±*¬bUXÅ*V±*¬«XÅ*VU¬b«XÅª°Ua«XÅ*V±*¬b«XVU¬b«Â*V±U¬bUXÅª°U¬bUXV±U¬«Â*V±Ua«Â*V±U¬bUXÅ*V±*¬«XÅ*VU¬b«XÅª°Ua«XÅª°*¬b«XVU¬b«Â*VU¬b«XÅª°U¬bUXV±U¬«XÅ*V±Ua«Â*V±UaUXÅ*V±*¬«XÅ*VU¬b«XÅ*V±Ua«XÅª°*¬b«XV±U¬b«Â*VU¬b«Âª°U¬bUXV±U¬«XÅ*V±U¬b«Â*V±UaUXÅ*V±*¬b«XÅ*VU¬«XÅ*V±Ua«XÅª°*¬b«XV±U¬b«XÅ*VU¬b«Âª°U¬bUXÅ*V±U¬«XV±U¬b«Â*V±UaUXÅ*V±*¬b«XÅ*VÕ<ÖÙÙYSSSTT´råÊîîn¬b«XÅª°úøUTTp!&+++±U¬b«Âj~*))û¯Z±bÅo¼¤/¥_ýêWïåµ6üÉ^U¬ÎògÅ·nÝ¯?¿Ý»w¿°ä«XÕ¼`µ««+F¥ÁêÔË/¿Õ/«`&ÉÄG¾~þ¹7×ÿV±:Ëç+ù£o#_~¯¿ö­ÅÏ?UÍVïÜ¹³yóæááa;Õü2ó§ßûwÿé?®Æ*VçÆöbUÈêÍ7nÝºåÀXÅ*V±*¬>Qíííuuu3,U¬b«XVgUyyy&'¬b«XÅª°ú»«XÅ*V±*¬b«XÅ*V±U¬«±½XVU¬b«Â*V±U¬bUXÅ*V±¬bUXV±U¬«XÅ*V±Ua«XÅ*V±U¬bUXÅíÅª°*¬b«XV±U¬b«Â*V±UÌ`«Âª°U¬bUXV±U¬«XÅ*V±U¬b«Â*fl/VUa«XÅª°U¬b«XV±U¬b«XVU¬b«Âª°U¬bUXÅ*V±U¬b«XÅ*V1c±*¬«XÅ*VU¬b«XÅª°U¬b3XÅª°*¬bÕöbUXV±U¬«XÅ*V±U¬b«XÅ*fl/VUa«XÅª°U¬b«XV±U¬b«XVU¬Ú^¬«Â*V±Ua«XÅ*V±U¬b«XÅíÅª°*¬b«XV±U¬b«Â*V±U¬b«XÅª°UÛUaUXÅ*V±*¬b«XÅ*V±U¬b«±½XVU¬b«Â*V±U¬bUXÅ*V±U¬b«XV±j±*¬«XÅ*VU¬b«XÅª°U¬b3¶«*PVÊËË±U¬b«ÂêRUUUÌ¿®CÇ¤6lØð$¬^½zõyí¿ü%VÕoÕV~òÉ'ùºqÿþïÿþÅ²%±½XÕÓÊjxÙ××eõþýû©zýõ×û÷¥ÊãÂ¼ü<óLIVÕgùý<Þ¾%%¿¿øùç0c±ª§û¹ÕÜÑê¯'µmÛ¶'­®]û<þ[~öÓ/½TÕÂa3¶«ÂêL¬æý¹U¬b3¶«Â*V±ÛUa«XÅ*V±U¬b«XÅíÅªæ«3U¬bÆöbUXÅ*V±U¬b«XÅ*V1c±*¬b«±½XV±UÌØ^¬«XÅ*V1U¬«XÅ*fl/VU¬b3¶«Â*V±U¬b«XÅ*V±ÛUa«XÅíÅª°U¬bÆöbUXÅ*V±¬bUXÅ*V1c±*¬b«±½XV±U¬Ú^¬b«XÅ*V1c±*¬b«±½XV±UÌØ^¬«XÅ*V1U¬«XÅ*fl/VU¬b3¶«Â*V±UÛU¬b«XÅ*fl/VU¬b3¶«Â*V±ÛUa«XÅ*f°Ua«XÅíÅª°U¬bÆöbUXÅ*V±j±U¬b«XÅíÅª°U¬bÆöbUXÅ*V1c±*¬b«XÅ*V±*¬b«±½XV±UÌØ^¬«XÅ*fl/V±U¬b«±½XV±UÌØ^¬«XÅ*fl/VU¬b«XÅ*V±U¬b3¶«Â*V±ÛUa«XÅíÅ*V±U¬b3¶«Â*V±ÛUa«XÅíÅª°U¬b«XÅ*V±U¬bÆöbUXÅ*V1c±*¬b«±½XVóXwwwYYYQQQuuõgU¬bÆöbUXü6oÞ|üøñhjjzë­·°UÌØ^¬«_iiéØØXLß¿¿rRÏ?ÿüsÏ=Wù¸¥¨ègã3/?/Ìd2q±_ÿú×W¬XQY¨UTT,_¾üÉ/'¶1¶7_×^üüÞïý¼_`oßEðl~W/3íÃÛûµ¯í±ÿ¿â«XÍsEEE¹ÓþùÿÔ~ð?þøÈãöÎ;ïlÏk»víõÕW®#ZcccüÃÿÝßýÝ^NSSS~¯½M6½ùæ1ñ¯|å»ßýî_àk¯½öÖ[oåkõ¶nÝ·lLÄ[VVöä¸q¼<^iÿøÿ8nßXÛ|mo¾Ênïb(V°·ïË/¿¼lÙ²¼_ÿõ_?öÿ×'|òë_ÿBXÍgñ¿...~®µ¿üË¿n]Á®^GGGÜíì>ÿüóG-ØÕûó?ÿóõë×ìê=6nß»wïìÆ¿ssssÁ®^øú½ïÏ½¿æ «K,I;c«XÅ*V±*¬>~[¶l9vìXLÄáæÍ±U¬b«Âêãwþüù¥K.X° ¬¬¬³³«XÅ*V±*¬ÎÇ°U¬bUÂjÞzÿý÷ÿìÏþ¬`Wïç?ÿù×¿þõ¡¡¡]Ãoë[­­­»zõWõýï¿`Wïþáâöý§ú§]Ão~ómmm»zñá~LX$	«$aU$a5gÎðÝ³ùÚüÖÙÙYSS¿qåÊñÛmõzW­Z~cú¶Z½léå?xûÞºu+S¡­Þ½÷/_ÞÞÞ^·oîµ>y¦0ÿÕyT`PUU½Ï0ãwÌækò[EEÅb¢¹¹¹²²²ÐV/îN:±¥¥¥¶z©ÑÑÑxh½j[[[Ã­Y®Ã¿zû÷ï?xðàØØXºbÅÂ¼Sñ«ã÷ò«ó¥õë×÷õõå²:á»rá¤/ ]½3gÎÄxº0WïÀÊÞÄµajz~ñ°éòåË»zÙ®]»/þÿ«óìÊauÂwä.6ÃI¿ëºººvîÜY«cÁó?é-¶z×¯_¯­­;ÓìM¿+VòÊ+¶zñ[>çb¨ÚÓÓS°ÿõõõñRÈÿ¿Âê¼fuïø²¾6àÎ;7o.ÌÕ0þäeúÀçB[½¸ÃíèèÈ½ó¼yófîÔêÅoLèäüyíõõõ­^½ºÿÕùÎêßð¥|m@Üá644Üºu«0WoÂÃÿB[½ÌoWÈW`º£/¨ÕËý-yû>8Ôûÿ+¬bu¦ïøâ¿6 ½½½®®n`` 0W¯¢¢¢··÷Áø+c=mõ¦¼íìïïOðÜ½wKKËñ|¯Y³¦0oßµk×^¼x±0ÿAU¬þ¦)¿ -ðÅm@yyùÁVA­^wwwuuucÖ­[ÆÓµzSÞÄµ]]]UUUq¾òÊ+!k¡­ÞÐÐPzê·¯¯¯0oßå§"àí+¬J$¬JUI°*IV%IV%IÂª$IX$IX$	«$aU$¬J$¬J³û[Ïdjjjiù;vLóæoº&%aUó½ááá@qëÖ­Sz÷îÝ	|^¹r%Ï=9::sÊËË]°ªùÞûï¿(nß¾óx­ã¥ÃÇI¹¬öööÆá#Gâ°±±1f¶µµ¼òÊ+!´ëSV5»víZèxîÜ¹?øàiÿ+2Ë/ÇáÉ'sgÍ1á+9%aUó´±±±0¤àÌ²ûM×1~Sô£¥Ã?þ8§OeÒI±¤ëVV5ÿþÄ3þþþ²²²ÚÚÚìßôò¥HFîÙ³'»ü½÷ÒDKKËõë×³ÓÍÍÍ1122²k×®	Ã_IÂªæÅPutt4¹XUU5åhuBaðÕ«WÓ2]]]ÝÝÝÙ×4ÅÌóçÏ§q­«WV5Ç¬³d5©Ë-»téR,sáÂ8Ü¸qãåË7×ÖÖv·®UIXÕüe5÷M«XmöÖÐ7í%¾qãFf¼Àudd$»À¡%	«/Ý»woõêÕ1Ä-Z[·nMGcþÖñÒÑ(=«¦o¿ývL=z4æ<xðöíÛ¡lÌ©¬¬f+VV5erð:ÝNà`õÄ¹KÖ××§ùK,Ù¼y³«TV¥Ù²ÚÔÔ§.^¼ø£>:uêTLïÚµ+ÔÙÙGwïÞíUiV¬^»v­··7´»»ûÎ;Ù£§OÎî.$¬JÿÌê±cÇ°*IV%IÂª$IÂª$IX$	«$	«$aU$¬JUIUI°*IV%IÂª$IÂª$IX$	«$éaý$ù1(IEND®B`


常態 Q-Q 圖


ôÑw'SQQµÈår?þjõWyüø±<Ë/«GõöÛo'ÔôæÍêGöìá÷dH%7nÜPcÇ%Luuu	³ÓÓÓ_õU^^J×÷ß¯æ_¸pANeØºò=Jµ¨(÷ïß9===¿c5¢ñE2ñüùóÉ°[æËcÞ»wOJ½zèëëÛ¾mmíàà Êm2Y^C?üpÇöRWØËOå¨e9sæLÂbÊ$« «@êðÈÿîÆÆFm±dÎVh-ËîÝ»µê`¯bW¸ºhÇÒì;wÊÌ?þXæ;vL®ÕÔÔÄ_åÝwßùr§2_ËÙ£GjW)))Q¿~ýºªp%]£££ò#µK*?BÉPÕ:;;[®.Ý2!gWxüe¯UyÒGæÈ"UU ³*¸BV<y¢þéñÅr*c>uVF>ø@®PVùég¦M;NÕéPüe?_ k×®ýâ<xP;+ÍjµªJþùçòSyáõzUn%çòe¢¢¢"ù¦ÔÅ¾üòËøj%üKÞûË^%>«Ú¨]æ=V&Ô;ÀddH12R¬ªªJUIiBYÕÄðð°zï÷§?ªTïµÞºuëîÝ»ª^	×ÕR¡>ý)îcøË|úé§Útff¦6]VVð1¤å<|ø0þä.¶mÛVWW'ùðÃÕÅÚg«wîÜiÃ±äj±ÙlòÓOåöefQQÑºõÊ+ûiË¢>dUÓêößyÈ*°Hÿä4ÈXSÆ[êº:Õ°Ú«ÚU$·QU¸;vÈ´´D¦ïß¿/3Ï9#ÓÏ=¸^ºtIû6ÓYÍÊÊpãÆøÇgu¹ë.7GÉÉÉÑ¾W,¯äbê[BêLÊpzzzÉÕ"_òkP+|±ëe¯¢Ðea5ZÃhdH1/_VßBZr´@*¢rX$Ú¦/ápX(¸ÒßÁ ÍKøUe¦¶¶ö³*×Mør`` ù===êF«n·[»OK#e¤»ä½/9_ÇUÔòÊ Y§¤¤D[­ª²²¤$5Ó¾YU5<¿ð÷ß/Åo¾ùFõw×¢øñnÂ;¢ËVµï=Éô¡C~úùÓYíó×ëÊ¿øOdãÍfI¾rå­ªYÈé¾û¯[PP .ÜÎììlíhVy%³ªûúúTPåõöá4YYRÆÂÂÓéÔsÚ§ÚÀñ_µ-Êjii©t·»»[ëÊ³gÏÔÍªAáo~óF«ñYu¹P_NNØodR=Zí!©-dúË/¿³	­ªÈõÓ£G&|ï)þµB©»ö~¬ÜE½¼nP«-WBVWså²*/Md)l6ºÙ[·nÉ©úÌ¬¬©Dka¼ßVYUïÖÞ¿_áHFiãããêT9ûñÇ¯ô·ôKYòäú²qÂµ´ÑgÂ$¶Û·oKûtsrrR¦Õ×¥Áápx¹/«w¶e)·<::ªF¢JüÖrä¬jC5¬¬[Äj²zïÞ=5àSo«>Y¤*¢ö´rVã?[H´ÝÅ_+~×ÉYÅ_>ª],%TP&äÁ<yr¹=4É`=yß+ï[ñ¥®Õ¼¼<Ã¡VÚÔûï¿OVAV­ ¯¯o¬ªúâÄW®ÓRªB¿û¤%³¿Ýª¶C¹«Õªn?~G¾ËVe|,RÀÙ³gÞVûR±.¹?Eõñ°Úf¹ÇyæÌ]»veddØl¶õYìÊ¶ú«$d5ùáýîw¿#« «ÀV ¾C»?~|ëÖ-í¬)a¸½,ißNøZú²ÒßýÑ¾Ð¤U±TMØ½ðôô´¶Äªªªøá£vVexÉM4??/ãQõòÚ¯¢÷;wîüÅ]í=zßIU óÍ7999ý2nkÚÕ§qóPïQ«íW^L5lÈ* «UÈ*dUÈ*dUÈ*d²È*d²È*300§½páBAA« «ÀJvíÚe0îÜ¹£Íiãt:Z<ò¶Ø ?Ëu½¯,ykO>­¯¯7/çÏ¯ðxN:¶¦¦_¬+9yò¤ãôéÓÚÏ>ûLæ8qb²:>>^YY¹¾Yn·¹[kllù===2ñá&_æÖ­[ß|óüôÊ+rzùòåû÷ïËÄÛo¿=88øî"~s²,!J0***´9399r#Tí6Õ",yËêGO<yüø±L%_fxxXÆ²Ú­IûUÕóçÏçääðU`iGÉt4iíCÄø8©ék×®fggúé§§N	«Õ*£:uÉÕ#G²²²$Kñ×r]¹ñK.i3µ»H¾¯Ë¯ÇãQÉ«òÀâïEËçYÑêõë×µæåå-c¬øµµµîînS>vìØrYÃa5âÄP($?u>ø@Î^¸pa``@&N<ÝÏ?ÿü»ï¾¿|òí¯pyÃR^v/¿Õ7oÞ¹sgppPËªyùµÈ*°4J<ªªªdúàÁê3ÅåR§¾ã£¦çççÕ´´J]fûöíjþ/dbÇñ×öìYBÌVÎjòå_â|mYU¯-$¥2qãÆuáºº:±¸öy0²$ÊàLóôéS9lÈÕd/yZEK£åöeogÉéu­êx¸¿¿_Õ´´´TÍ!«YVräÈ©vºdV3#ÓZußÎrÓk­©¯,U~eIeõÞ½±XLÍq»ÝüÎdXCÿ]¸pAw>úÒìíÛ·e¢¤¤dåËggg'¼±ü²ª=qâLô,Zn%³SÕÕÕüÎdXÖ³gÏÔû·rúøñcÝYÕv¶°mÛ¶ûöÝ¿åË_ºtI.¹÷îËª<B¯×+w%UÎ.ÕäÏVeµÈ£Õ¶dåw «Vett4h=þöÛoÕt,ÿ²Y¬@VY¬@V7È?ýÓ?åçç[xÝÞ|óÍñññÔÎê±cÇ*++ïðº½óÎ;ÿöoÿòYÝ·oßxÝþú¯ÿ¬@VÉ*¬UY%«UÈ*YU² «d²JVd¬È*Y¬@VÉ*¬UYÝY½yófIIIff¦Óé$«²ª_QQÑ7dâüùó;wîüñÇ¯$Ù»w/YÕõÃ?òÞïñÈêj;vL&'ùøF«²ºZO<ñx<O>å³UY]=zôáÃ|@V×$ìÙ³çÑ£Gl` «kUXXÿÕ$² «ì@VÉ*d²JVd¬@V «d@VÉ*¬UÈ*d¬È*Y¬@VÉ*¬UY%«UÈ*YU² «d²JVd¬È*Y¬@VÉ*àU	Ãd¬Ö*655Y,©©)²JVú9N³ÙÜÚÚÅÈ*Yè177WWWg4].×ììì+º²Øúü~¿ÕjAj[[[4uwDV[Y(òx<¡ººzffæUßYlY]]]%??¿··wcî¬¶ ºÝn¤ú|¾H$²a÷KV[J,kii1Í6mhhhï¬¶	Ãa4æææ6þUÀVF%¥Ôòòrëëzdò®^½j·ÛM&SKKË«ØÉY¤H$âóùdêv»_ÅÎÉ* ]ôööæçç[,®®®MòÈ* õB!µýÇãéÍóÀÈ* ÅtttÈÕf³ùýþÍöØÈ* eAË¥vòðZ¶!«­@ÛÉÃáÙ´¬6»@  vòÐÔÔôz·!«FÕö3¯w'dòü~¿Ýn·X,|JVvT·Û½©¶!«£vò°I%«d¶ í ©^¯w#JVÉ*l5ÚAR7áNÈ* eA¤¾Æ¤U²[A,knnAjqqqJl?CVT (--AjKKKªRÉ*à5RM&Ëå[iÑÈ*`C8³ÙÜÞÞ*;y «M'ÔÖÖÊ ÕãñÌÎÎnÉe$«pîÜ9Íf·ÛSq'd°©jOö ©d¬@jèèè°X,ÅÅÅ@ ¬^ýû÷Æúúú-?H%«W%µ¶¶Íæ-¶ýYl¨@ àp8L&SKKËÖÛ~¬6H4­­­5n·«n?CVahhÈf³åççwvv¦á ¬ÖG8öz½2H­®®é4_d _WWPezîÜ9ÖYè«ªªCZm?CVë,µ··L&Ã122Â!«ÆÆÆFcccc4eUsssuuuÔÊÊJ++¬tºxñ¢Íf3Lmmm¯zû®®®ÃWWW755E"²JV`ë°IäÔö3SSS¯úîä^ÊËË¥âCCC2ít:SëûPd°¬ÞÞÞÜÜÜüüüþþþ¸;§JSã;*Eohh «dRÛììlee¥Á`ðx<¶'B¨SãçU²©Jm?c6ív»ßïßÈ»'ÜãÔÔÓé$«dRÒÄÄDyy¹R>ßÆ¨ÙÜÜ¼ÿþø92%«dRL4mjj2LU	Ãkyr¹w³É8µ±±ÑápÌÌÌÕõôèÑ£ÂÂB²¯ÎÈÈHii©ÑhÑáëÝÉª»Ëår:2NM­¦¦@VGGGå¥Á` «ð2¦vòPQQ111ÁÙâY^Þ¹sGËê?üð¼ñÆ÷îå¹å÷û­V«ÙlnooOç¤¦QVÿøh~Îê?þx:É;ï¼CVà¥ÃauüÇro´ÕuË*oÀÚuuuY,ÜÜJVÉ*è'SË%ÿT>,VVY%« G,kkk3Í6íêÕ«¬²JV@'ùG¯¶©¯¯O­=×UvH4mll4LÅÅÅl?CVÉ*è744äp8dÚÜÜÌ ¬UÐ?H­©© VVV²ýY%« _¿ÕjµX,ìä¬UÐ/<Á°ÿþ;H*È*lA­­­2Bqjoo/k¬UÐibbBíä¡¦¦&±BÈ*Y=¢ÑhCCÑht8ìä¬UÐ/HMM&SKK_M"«dtD">O$ujjBVÉ*èÔÛÛo±X:::¤U²:B!µýòÕ$²JV@?ÊUÆ©~¿µAVÉ*è433SYY)TÏDX!d¬±X¬¥¥Ål6°BÈ*YÔö3F£±¡¡ãÏU²:i;y(//ç ©d¬~~¿ßn·L&¶!«dôD"^¯×`0¸Ýnvò@VÉ*è§vò 8þY%« ßÌÌOÙÉY%«°Vf³Ùf³±²JV@?í ©l?CVÉ*èÅÔARÙÉY%« I%«dÖvTvò@VÉ*¬ÉÅ9H*Y%«H¢(ÿ7Ï;·Êg8öz½2Heû²JVà»)¥Íf«ªªF£+»»;w;y «dþH$"ìèèÐ­2­©©YòÂSSSû÷ï7r©d¬@¢sçÎI)ãçH/-Kòg¥íííìä¬UXI[[[SSSÂLigü`Tíä¤U²¿ ¿¿¿¢¢"~ÎÔÔTnn®­ª<È Õét±ºÈ*YH8%2UÃRÙææf)..¦Êvå/1¬Uø£P(är¹l6UÆ©uuu×úúz£Ñ(s8H*È*è«üë úý~«Õ*íêêb' « Óìì¬Çã1^¯íg@V@¿ÞÞ^Ë¢þþ~ÖÈ*è455UQQÁ dÖ$µ¶¶L&§ÓX! « ÓØØXii©RÙ~dt644F© «°&~¿ßápX,ööv© « S$©­­Ajee%_MYýúûûsss¤¬ÀÈÀT§l?²JV¬Ukk«ÕjµÙl|5	d¬ÐO$A*6]Voß¾í^äñxúûûåwtzzZ£f:uJæ÷ÝwdÀk§m?S^^Î .«óóó/^¼øãMf³ùÁj=~ü8£UtÔápL¦ææf?ÍUÏÉÉÉÁÁA5'++K¦e~mm­Llß¾½¬¬¬x"ÏçAªËå¬lÞ¬ª²ø³_|ñvvÇ»ví"«^ÞÞÞüü|ÅÒÙÙÉ :«ÃÃÃýýýgÎöõõÉ©ÌÓÈ8ÕëõªaëÑ£GÉ*ÔARÝnw8f`³gõùóçrúôéSù­½÷®^¿~]N/]º¤]Æf³U¬££CF¨V«õÜ¹sRYUTV?FÕÀ;vìQ¬öõ`F«6ÒÌÌÚÉÏçD"¬¤dVµÏV¿üòKF«6J[ZZÌf³RX!Ø"YÆV½^/Yðª©ígFcSSÓÜÜ+[$«/_F£êc×éééP(ôøñc²àÕÑvòPZZ*ÿãX!HÕ¬>yòdaaááÃêËJÉÜ¨/KYÉ*WÄï÷ÛívÉÔÒÒÂñgÚYöìÙ_|!á,((oªÃáÐÎ<yRO¬X_HÄëõÊË533ÃAÊgUÑÞã×òûåÊøÊpVf^¾|¬XGÚN:::V¿ýLÿþýûå¥¿;wÕÍU``#ÉÀÔívËëõªªª:þL»ÍfÊ-È©Ýnokkc¬U ÉØÔl6çææ^¼xñ¥®eh;55g3;;ËZÅæÊª¼f<qâPÕu:555l·`íÔARFc]]<øý~Ý&Ì¬®®îïïgÝbÓeõÔ©Sj¢´´T&.]º$Ó÷îÝiùdn±X¬©©Ij·ÛuïäA²*M©6¯gc³du||ðAjª&Ô^­Õn÷ïß¿¯²GVè£íä¡¡¡a-;yÃò?ùM`¾BMUíå999*«ï¿ÿ¾ÌQ®ÑF«dÚARËËË'&&Ö~íííjÏûÚWZ[[YÏØDYÕÞ>ú´LÈ+Á·ß~[Ëª"33óàÁdÀKÊÏÏ7LÍÍÍëxüµMqq±ÛíflÞ¬.9ZåM`:Ãa¯×+T)_ü¶@úVUb>|HV¼/Z­V§ööö²6@VÿÕ¯¿þZé7¬ÒììlUUIEZgõúõëêý^©©LÔÖÖÊ´ÅbùàÎ?¯.ÃhÀÊb±X[[üë°Ùl$iÕùùy×Ë//,,Ü¸qC¿þæ7¿!«~ÑÔÔTeeåÚ·¶BV:*Oe:Èôä4U+R[ZZÌfózm?¤|V<x pjs&''eÎàà LÃaõ±6r%«yît:ÕAR×qû ³:???>><rjsîÞ½«Æ¯dFØ~d#ØX+¿ßo·Û-KWWkdõ´Z­»víºvíY¶H$¢ö.§/uT ²:>>~`gQÿÏÔ´ÚzU¦Wrá¾¾>8öì¡C~üñÇÓIÞyç²¤Ê+fÍ&£UÖÈê²ÔÞöw¡O^^ºÁùùùÂÂÂ~øÁä7ÞØ»w/Ï%Á`EEÚÉÛÏ¬êßã>KNó&0rÔARÍf³Ãáa¬¾¬fddhÓÛ¶m#«@4¥¥¥F£QÊÊö3 «/wÈUmw	YUû`z)Û·oW×S&«@Ê«««[Ç¤i7ZO©Ú'°úÊ~©:räHOOLÈérßu"«À¦544d³Ùä¿££A*Èêúdu-odddX­Ö7oU UD"Ï§vòÀö3 «%«ìHE½½½$d¬X+ÊðTþð½^/IY%«ôëèèPIe' «ÕX,FV­'sT`=³:<<]­¾ñ[ZZªûWîéé³ËíÕ¬©HÆd29¶Ö3«Ï?ç6@úÔtddäÄo½õ;y^IV90&®^½ZXXøæoþêW¿úÓ?ýÓ¿ýÛ¿¥©À+Ìª¼PG²Ûí;wîT»I2ß~û-YRZ0Ü¶mÛ¯ýkµH$RQQÑÐÐÀ^áhõÆÑp8WVV¦¾»$ñc´¤4¨¼P?g·Û=33£fÉhVðJ²*o.«¿¿?33SN³²²ä%íéÓ§Ï9£½*¡-**"«@Ê¿_Íf2|>_Â»¾2õ¬VïÝ»'Y» «@	ÃÕÕÕòÇ[[[ûûßÿÞëõÆÿôêÕ«µ¬Vå¯n÷îÝ2Nç#G¼ÍfóñãÇçççïÞ½ËÀ@ioo·X,v»hhèï[­V©¬333ÒÔîînVðW÷Ùêðð°$6¾ |¶¤yëêê¢Ñhü|yÝ\?â655±®Èê<Ô~!=JV #Ñ³Ù:66¶äed*ÿ>Ø§ðÊ³*ýðÃåt``@þ,srrålww÷/~òJV×.ÈHTþxÛÚÚØ ØYýì³ÏÔ´Íf+))ÿÄ¬S8öz½F£±²²2²BÍU­¥¥¥ñ?:þ<Y6!¿ß/Ô®®.©À&­j»p8êÖ[·nU`S	BG©UUUÚNl¢¬$dõÅ2ÿÝwßå+KÀ¦ÒÞÞkµZ/^¼ÈÚ6cVãegg:t]íLµ<D"VYå6À&ÅÍf³Ýn÷ûý¬¬U@§ââb£ÑØÐÐÀV§Y%«þAª¤Têr¹ÛÉ²JV_æ÷ûÕñgZ[[Ù~ «dÐIm?£:55ÅÈ*Ytêêê²X,ùùù½½½¬¬U@§Ê Õçó±ý@VÉ* vüÍ¦¬U@	ÃÁö3Y%«ÀD£QµýLyy¹ÄU²ètõêU»Ýn2ZZZØ~ «dÐ)ø|>¤²ý@VÉ*°&½½½ùùù¥««µU²èÔö3G¦Y!Y%«N2BµÙl «dÐ/ºµ¶È*YtÒvòàp8FFFX!Y%«N@@íä¡©©íg²JV=ÁàoûÛ_ýêW$ «dÐ?Bý³?û³?ÿó?ÏÎÎ®¯¯ÿ¿ùªª*ªY%«ÀKB¿þõ¯ÕARÕö3Ñh´´´ã»d¬/GÚ¹ûö?ù?ùùøù2leýd¬«¢$õïþîï¤¬	où¶··U¬U`U´¤ª<8Îîîní§¼	U²¬J0,//O8Hj ÈÍÍmmm©¬¬ä+KY%«À/hoo7L26M>HªäÖãñ8ËÕÜÜ,VV@VÉ*°ì UÆ ÒÔ¦¦&	U²è$mhh VTTÌÎÎ²B²JVÔ-Kgg'd¬:ÍÍÍù|>£Ñxøða©Y%«~ÝÝÝV«5??ÿÜ¹s¬¬U@§p8ìõzeZ[[ËAR²JVýÚÛÛ-Ýn¬¬U@'é¨ÚÉCcc#T¬U@§X,ÖÜÜ,A¬NMM±B²JVÆÆÆÙlnmmeû¬U@§H$¢¶q»ÝR²JVý®^½Z\l±Xº»»¤d¬:ÃaÇ#Ôêêj°²B²JV:;;óóó­V«VYY%«N³³³n·Û`0ÔÕÕ1H@Vb±XSSÙlv8ò'Ä@VFFFN§:H*_M@VýÔúúz	ªËå` «N~¿ßf³ISÛÚÚ¤ «NápøðáÃF£qÿþý$YôëííÍÏÏ7Í2ÁÚ@Vd`*ÃSÁàñx¤ «~2BÍÍÍ½xñ"kYtÚARÙÉ²èFÕNNg ` «Nò 5AjKKô¬zD"ÚÚZ	jEE;y@VÉ*ôëïïWÛÏ´¶¶²d¬B§P(T]]­vò033Ã@VÉ*têîîÎ_ÔÑÑÁ @eõÑ£GdëE¦ÁëõÎÍÍ±B¤QVGGGüÔæü¿$>¬bZ[[ÍfsqqñÕ«WYÒ.«ÒË;wîhYýáKyï½÷x.±²±±1µÚÚZ©Ò4«|4?gõÇü¿IöîÝËh+ÅbMMM&ÉétJ!ÈªÏV7ÆÔÔTgggûyT:êp8d*ÅW¤cVµ·vÉêëîî6Í555v»½ªª*¥w6$¾¾¾^©åååR0Ze´º¡Á`nn®ü6hMr»Ý¥]®®®ÜEòZA*²JV7Zkk«RãçLMMÉ5ådffF$µºº¤ «ìâõ¨««ëììLi2Rk)ÚÛÛÍfs~~>ÛÏ «dõ5IwñsGª<þ	Ëe4kjjÂá0O(²JV_§H$b³ÙÔ62Ú³Ûí½½½ÿG£Ñ	ª¼à«IÈ*YÝ,fff***rss­.^¼¸ùs L¦¾¬ÕÍ8økJ¯>:HêÔÔO²JV¡Sooo~~¾Åbáø3È*Y~¡PÈãñ9iV²JV¡Me*ãT¿ßÏÚ@VÉ*tÒêóùRâ£_d¬b3Åb---ê ©###¬d¬B'µýÑhlhhà ©È*YNÚNÊËË'&&X!È*YN~¿ßn·L&¶@VÉ*ôD"^¯×`0¸Ýnvò¬Uè§vò Rb_Ä@V±IÍÌÌÈð< «dkÕÑÑa6m6;y@VÉ*ôSIA*ÛÏ «dúÅb±¦¦&uTvò¬UèÇARU²u $< «dk244ÄARU²µÃG©l?¬U¬É¹sçr±d¬B¿ªª*ÁPSSÃ Y%«Ð¯««< «dk%¿Rååå$Y%«Xhcc£Éd¬±BU²ýTµÖÖV¶@VÉ*tÃuuuìäÈ*ÖÊï÷[­Vvòdköïß¯:;;Ë²=dTÚÙÙËö3@V±&333ê ©^¯<YþAj[[Ùlv8CCC¬ «ÐibbÂétÆÆÆÆh4Ê²=æææjkk%¨.+²B¬B§«W¯ÚívijKKÛÏYNH¤¦¦Fêv»gffX!@V¡Sgg§ÕjÍÍÍ	©@V¡S(á©Á`ðù|ápd:µ¶¶Z,§^¼xµd:AµýLmm-I²¢ÑhKKÙl¬rT «dU¿µý;y²JVõD"ÚN¤Y%«ú©<Íf¶²JVõÃV;yà ©@VÉª~]]]A*U²º&333û÷ï72Ted¬ê×ÛÛ+TÍÆAR¬UýÆÆÆÊËËFc=;y²JVuÅb­­­&<Y%«kÅbiiia'@VÉªNsss>íg¬Õµêïï·Ûí2Híèè`û «dUÿ µ¦¦A*U²ºV26ÍÏÏ·Ùl½½½R¬Ud`ZUUe0|>ÛÏY%«ú©=Ê Õï÷ó«d¬ê411±ÿ~£ÑØÐÐÀ È*YÕ¯©©IÊN¬ÕuPUUÕÖÖÆN¬UY%«UÈ*YU² «d²JVd¬È*Y¬@VÉ*¬UY%«lõ¬Þ¼y³¤¤$33ÓétNNNUYÕ¯¨¨èÆ2qþüù;wUY]YYY/^¼8Dr»wï^KY]­ññq)¨duo7ß|¬Èêj=yòÄãñ<ú7dõå~¦Î>xðàèÑ£>äÀ²º&@`Ï==bY]«ÂÂBC² «ì@VÉ*d²JVd¬@V «d@VÉ*¬UÈ*d¬È*Y¬@VãÔÕÕ½ñÆ;×Õ[o½e·Ûw¦¥¿üË¿ü¿ø´]ö;v¤ç²¥í²Ë²Ëiz.¾,»<ûi»ìòW¿¾·)=Jù¬B¡¯Ö[EEÃáø*-<x°   =ýý÷ßß¶m[z.½Á`øçþç4'OÊ²·´´¤á²wttÈ²?~<=íåï½¦¦fo³··÷¿þë¿R;«¯Â?þã?¾÷Þé¹ì¿ÿýïå%Ez.OOOvvvz.ûÈÈüýïÿþï4ÿ÷eMÃeÿÃþ Ëþ¯ÿú¯éùk/#Ë¾¾¾¹/²JVÉ*Y%«d¬U²JVÉ*Y%«d¬U²JVÉ*Y%«d¬U²JVÉ*Y%«dõ|ôÑGÿ÷ËÞÙÙ¹oß¾ôô7n¼õÖ[O>MÃeÿÿøYöÿüÏÿLÃeR²ì7oÞLÏ_û¿ú«¿òûýdCV «Õ×nzzº¬¬,33s×®]£££2grrÒjµª9×®]K«eøð¡!ÎöµÅLç=yÙÓçyO^Ò´zÞ?úçÏ=ztÛ¶m;vìùÔ§]Ve^ºté§Å/näååÉÇãQß;öì¡CÒjÙûûûå7/Múí_Iú<ïÉË>Ïò¦Õó¼øéóÔ<yòÓO?ñâ4Õn·oäS¾o_¹rÅétÊFV½LÌÏÏ¦Õ²Ëm:øäON:¥¥%­÷eOç=yIÓêyO^üôyêeqûöí×òÔ§cVåvv¶ü9þ¼ÍÌÌÔ~?Ë^TT´gÏYêÒÒÒï¿ÿ~/ø½÷dåJKKú<ïÉË>Ïò¦Õßòâ§ÏS/ËøùçgeeÉPõÖ­[ùÔ§ïhuxxxûöí2¡ÍÜ¶m[Z-»æÁjüºUUWW_¿~ý~ãNKú<ïÉË>Ïò¦áßûOôêåîîî	yõ ¯!6ò©Oëo«,ùùyõÎ@Bl¶ü²ÇÛÚÿbÿ[Z=ïÉË>Ïò¦çßûOôÖ~êãÜþWvY-**7oîÙ³G&9ÒÓÓóÓâí<OZ-»Ì¹÷®zéªælyZWÒçyO^öôyÞ4­÷äÅO§¾®®îÂ?-nár¹6ò©O»¬NNNîÚµK^¼ìÞ½ûáÃ2gtt´   ##Ãjµní=%/ûøø¸Ãá9ògViIç=yÙÓçyO^Ò´zÞ?úÇWWW«OïÜ¹³O=»¬@V «¬@V «U@V «U@V «ðí·ßçÏËé'|"srrrvíÚÕßßïñxäTæ<yrÙ¿yAÐcêç¿ûÝïÔÙ§OÊYuôJdØ"O8!»pá~üñÇrj±X@|8¿úê+í¬Ãá(++ó.«_ºtI.pàÀmÎg&s?~¬]ettTæhGùPYà)È*°¥È uxxX"'Ó+W®üôóÊãG«ZeïÝ»'gïß¿/§»wïV3=z0~mhhH#õ;ª®®üúë¯eÎ¾ûdúË/¿é³gÏòdHyEEEß|óMvv¶N2ÙÓÓsúôié8ã£xþüy5mµZeHªâ*×±©LÈ`t×®]j¨Ú××'s¾ûî;íêjl:==-	Ws<y"s¤²¬¬[ë/Ö`øâ/ÔØTü³èã?FªRÆVµk©cßºuK&Á ªc;ËDYYYüU§åvdZøÌ32G»qé:O@V­àòåËóóóRDõj4ÅbªyKVÕÙë×¯ª÷r%À2óûï¿/((8tèLÄß¾ÜøÁÕûÆêºÕÕÕ	ÛáÈ*°u¬×øoêÊtUUÕYvË¨EµVÕÛ¿wïÞM¸ñééi9ýöÛoµa®k£Uìò,dØÔ§¤§OÎÎÎîîîVcÇÌÌL»ÝÞÿ³ø7eô©êxûöm9.R_JRyyyRÊ/^ÈËt|­ÆU ôÑG*lrªF¡PHN'''ÕùÙlÖ¾m¤¨¦JqnMRúðáCõ%áäv2ZÈ*°eE£ÑG©í[äTHÒ©êØ××gµZ®¥¶p=räVÇøF-ñÑ*@Vt ¶~	ÃrÚÓÓ£ÍÏÈÈx÷Ýw¥*«òS	­V#Ñ[·nÉ©YÕ;½2T]öÿ£U¬é@mBªÆ©êô>PïKq%*	;|üø±¤¤TËªÚ­Äµk×^v´:==½B² eÜ¿ÿòåËÚÙmgj³Ôªªªø±ìÀÀÅbC¹ð;Ün·úQ4åää$gõë¯¿Vß~Ë'VëëëÕ>%x:²¤;wöõõig¯°#~þ&XÕn¥ÊI²Y¬²Y¬@VY¬@VY¬@V «¬@V «à§þ?.HÖÖ-vuIEND®B`


ýþýûeaÛ¶m¶ùàÁrëÑ£Gå²­­íòåË²ðÜsÏ>|øq<r²LBå'++K[#ñ5ÝÝÝóe:ÍakÈJõÝÝ¸qãúõë²°fÍw<vìeµ"íW%VkxäddãÚµk²dY18jùøñãÉÉÉñññôÑ®]»dÁb±È¨Nm#¹Ú´iÄL²äõzoß¾|_Ï'÷~èÐ!m¥ö)&~®íïÉÉ'¿|aÁEËç4YÑê'´['1@Vü   @âQ[[+Ër<w:1uRÓ¡¡!µ¼ûv¿ß/?µÍ;ï¼#W8ÐÒÒ";vì¾ïÇ|îÜ¹àí'~üi¶×MfÒïèæÍ©©©rëgrSTTTðg¾kVO:uáÂÃkY!/¬A¡Ä#''G_í5õâT©S¯ñQËcccjYZ¥¶Y¾|¹ZçÎYX±bEð¿ùæMÕÛÏËåÉ÷2ñ¦fUýo!)øªeAÆâÚóÁÈ*J(3	òäR²!kf½Ë*Z-·÷úq&]áhuïÞ½²Þb±·1	ÜÜÜ¬j®ÖU¬ÓÙ´iÔB»üã¯Ê=&0!!Aµ*ýq¦Z¾«¡¡¡booï¤¬Y³F½dIÌð%K*«.]UkN'¬Srhã¿Ãüü|õ¢ÙYX½zõôÛÇÇÇL,ßgV·lÙ"766NÕõíÛ·ËÂþqS½ÁfÒ¬(ËÅc «À¾ùæ5+×¯_;«ÚÁdÈøÊ+¯|yúí:$[®]»v¶²8é,±¶,_¡ÇãO'_êÄãE|?Ås«²[ä«ÕÞÉÊc «f¤³³Óçói=þâ/Ôòèèhðë°UÈ*d²È*d²:GþéþÉl6[xØ|òÉ3gÎÌï¬nÙ²%;;ûÛóÏ?ÿÿùó>«¯¼òÊ×<lñAV «d@VÉ*¬UÈ*d¬È*YU²Y%«²JVd¬@V «d@VÉ*¬>Y=uêÔêÕ«£££Gww7YÕð¥¦¦<yRW®Ýwßàå_&«ðhÆVUUÕGH\Ü·ß~«ÌK/½Äc5f³Y²ZSSãr¹l6Ûàà Y$9sfË-²pk_ÿú×Và§JSûûûµ5ÅÅÅÙÙÙdõá»qãÛí¾yó&Ï­À|Q:.xM 0LrIV¦+W®lÞ¼ùêÕ«¼æÂÂÂêêê)))CCCdõ¡ñù|ëÖ­»vío°ùEêt:×tuuY,ý]?êYMNN~iYù"Øl¶¢¢"õ`iªU'_É*ÌßïÏÉÉ1LT§VUU-øo¬¬ÑÑÑý|*Y¬UY%«²JV «ehh(??¿²²¬UÀ©©©1)))­­­d¬ÂÔÕÕ©×ëµcJU²¸gCCCTÉªÄuådðhihh0«©©__<YÌI`ee¥Ûív¹Æ´­ÏçËÈÈÐétgxxx>î²RA)¢Óél=næe»çççëõz»ÝÞÞÞ>÷YÌÂÂÂà9ÛÌÌÌ²²²Ü·®®Îl6Æªªªy7ëKV³Oª!¯-jjjr¹ßëìÙ³YYY:Îívûýþ°È*`v²*^ÓÚÚ:MV×ë]³¾d0ûóòò×äääL5	µZF£l0ßgÉ*`öÛl6)ëÙq'==âKúûûNçBõ%«UV³¦SË¨TÆ¦AÆ©óâ0dðJMcccÞ¬/YÌ¿ßïv»u:Óéìëë[ðß/Y<jÖw|¬UxÈ&Ôõù|v»]¯×½Þùrò²JVà¡)//7LE.Ýn·v_í0dÖ¬îWaa¡ÃáèééQõx<rU­UUUjÖ·®®nqî²¸7ÒQÁ088¼Òn·Ûl¶E8ëKV÷¥««+333¸²ùùù+V¬9~!Y%«»JIIQ¯TR'¿øÅ/jkkÙ9dpÏ²²²~ýë_;N½^/CÕ¦¦&£Ñ2-LVÉ*àî@AAADDÄÒ¥K%®¹¹¹ÒÔÅóÎT²5ê0f³ùO>ill,++«­­eJV÷æ¿þë¿^|ñEN¯½Kdpod0úôÓOGFFØØØ¸|²JVàA9tèP\ô®]»ÀÐÐPVVVii)¬îÔãñDEE-[¶,øÙÓ¾¾>ÉÄ¬fDY^^n4m6Û[o½URR²AJJßïgGUÀ]´··KMCee¥ôµ¡¡Áívo0<<Ìh¬îB yyyz½^:Úßß¯VÍæææfm³ËÅî"«Éi³¾v»ÝçóÜÚÑÑ!ÃÓÜÜÜÌÌLÃÁlÈ*`ríííRSiªõ² V5f­««ô644,æÔUÀ$jÖ7''G¿Þ4##Ãét655µ¶¶®_¿>==]Ý²RUUÉdJII9rä¶²¸¸Xür$³±»È*`r>O¤¡¤¤$dFWÖwuu¯«2`e§U@(uÊq½^Ý××7qÉjÈ©ÈzzÈ*Yª««KII1Í²0Õ»Nóòò×HÙd644´··O|¥®@³²²Ô¬ïôï[m6Uî"ãTi*T"«°èHÿÔ[KN§G%®j½$¶¨¨hYßIË*cÖôq2N¥©dÊÊJ»Ý®½¦µµUÊÚßß_WWg±Xd¸9Í¬/]V¯]»LV`*ô7ó7V«U©2TåHdõ:;;åá¢Óýñë¹=Á[o½EV,fF£Q[%%%111===ì²ú'¤.²úí·ßê&óÒK/ñ³°G«ò§üë ×úfggÿã?þ#¬NNËêwßwxi*£UYeeåOúÓµk×êõzÇó»ßýN=·Ê!«wÉ*Ï­@5ëå'M>!È*YimmµZ­F£±ªªê¿ÿû¿d¯Q"«dîÁï½÷ÄODFFÊÇ´´4:JVÉ*ÉårIP£££~úé¿ú«¿å·ß~ÝBV9Ü³êêji<öØcåååêûöí`ÀJVÉ*Ü¿ßïv»¥©qqqÁ7-]º´©©]DVÉ*ÜJËÊÊF£ÝnóÍ7-KÈd¬UÏ'5¦ôööFDD´µµi0	LVÉ*Üßï÷x<z½>'''øä3¿üå/£¢¢¶nÝÚÑÑñöÛoó%²JV`:£££UUU2BµÙlO>óÞï-[¶ì±Ç3L^¯=FVÉ*LNÍúÎäã «d¦$ÍÏÏ×ëõë×¯?ö,;¬U¸7nËýìgF£Ñjµ644°gÈ*YnjTTTEELSSSu:Ý²eË&>²JVà.eú»ßýNÍúfee<x0ä3 «df¤¥¥%66Ö<®¦¦F­|ê©§xY/Y%«poÎ=û³ýL§Óhë-[öÞï±È*YÊxT¯×gddDEEåççk7UWWGDD>½DVÉ*Ü]SSÅbQoH­¯¯Z­Ö¼¼¼U«VEFF²È*Y»èééÉÉÉÑétn·Ûï÷këåï¯üùzægþò/ÿ²££EVÉ*L'ÈØTF¨)))­­­òW©/^dÏU²÷¦½½ÝjµÆÆÆJY,YòØcÆ_üâRY%«pwjÖ×étöõõDEEýò¿T)=ú´$öÅ_dGU²Ó---!©õU+%¨2lÞ¬­­MÆ¬XÉ*Y)µ··Ûl6½^ïõzßºjÕª¼¼¼e3ÞNCVÉ*L"dÖ7äVùõóÿ<xÍÀÀ£U²JV 6ëk6ëêêoVáliiVTThë<½GVÉ*üQWW6ë|Êñ#GØívÅb2222zzz¤©QQQ			V«U¤©¢¬Uø=¿ß¯N>#Õ9å¸DTjZ[[;:®²²R²ÒQëÖ­[eðÊ$«d~¯ªªJÍúÖÔÔL<Cjnnnyyyð¢¢"NMCVÉ*êèèHOOAªU§Åu8!¯Zjoo_¿~=¬Uø½ifCdggKG×ÔÖÖÊHVÉ*ü@ÍúYíííÝ·oßÀÀÀ¤WVVÊVßêÐÐÍfkhh`7U²`±óù|ÒHN§fO>-q1«:´ïªU«&ïi^^Ùl.***,,-ËÊÊØd¬XÔ$¢jÖ×n·«³¶]¼xñ±ÇËÈÈP)1«$SÊ:éÝåokEE1Y%«¾nj³¾jåÖ­[eMðfÚd¦#ËÌÌLNçñxO9.^õÕ¬¬¬í9´/È*Lbhh¨°°0xÖ7V-[¼FÊhdB577ÍfÁPVV|Yïõz.^¼(ÑÝ¸q£ºIjúãÿ8--½²¿×ÓÓ¥ÓéW¿¶^ª##×ªªªôôt¹ÚÔÔoµZe!))Cû¬ÀFFFJJJÔ)ÇCà ä¦ÌÌLmW²³³eä*ÕÕÕ[·n­¯¯g¬À¥¦±±±!§×HSCaUïae×¬Àõ÷÷¿þúëK.]²dÃá¨­­¸YFFFÈ¡åªlÌYVUUýèGv»½§§çëñãà[­ÖÊÊÊóòò×ÈUí²_«|ÊXS½÷'?ùIð»b¤¯ÒÚ©àÁÁA³Ù*ëeãòòrÉ2~È*EgxxØårétºÿüçW°C¿&µ««+deNNiÓéT£[¬X¤FGG+++Õañ¥©¹¹¹²,¥,((ÙÒn·sð^U²`J2út8QQQáðáÃj¥ßïúé§,Y<ô¬««³X,ÁGÈ*üvò¿þë¿.//¾µ©©)--M­^¯·¦¦ÆãñHS>ûd¬µwï^Ifll¬üû×ý×C=ô÷÷Ûl6­HtC©U²à÷>ÿüóv»½¹¹¹®®Nd`rñ§ÓÉîY%«&722âõzÃýÙiÂÿz|6899ùñÇ×æxe`*­²²Ó°³ÚÝÝm±X¢££åÿÍãÇU555Y­Vù[¡^yrìû6L¦ÜÜ())a§afÕív×××ËÂÞ½ßxã² X¿üÐétEEEê`f³9äì§ÕÕÕrë#G$ÀÏ:«wîÜ±±±äädYþË	äÓ_~%°¨Í&áÜ·o_oo¯¬ÏÎÎ®ªªÞ2++K6`¬þ :::xY²Z0Á3Ï<CVEEÍúÆ÷ßÿÇ?þqdd¤:àCRRR[[,KG¥»~¿ßãñddd_ÅÕ¨¨(m9&&I``ëïïW!t»ÝñññO=õÔÅå&¹´X,RÖI©dåj^^§Yý£åË©I`Y&«À¢%£Ï²²25ë«Nºoß¾èèèàèÀÀYO>ÍîYÜ¦Möïß/r)ÿU`qjoo·ÛíF£±ªªJ;Ä ×ëµZ­![ª`öÈêä:;;¢¢¢,Ë©S§È*°Øøý~õZ_¹9VcccG«ÃÃÃVAV9PjÖWF2NUGdff¦§§<þþþ%K¿"iÕªUË-c×¬UÂçó©Y_)«6ë#Míèèèëë+..6Lt©Y­VkLLTV½Í «dÀÔ»bôzÈ¬oSS6xÊ·¼¼Úwß¾[·n­®®æ4 «dÀUUUÉU­­­!7y½Þà5###2`e§¬U¡|>_ffflllð¬oHV+++×ÈØ¬¬UB;å¸Óé9`¯ÜÔÔÔÔÒÒ"f «dÀjjjÌfsJJÊÄYß7FDDÄÇÇÇÄÄDEEíÛ·oâKzzzØ «dÀ×gÏFÊ ÕëõªÏÛºu«Ü$ãTuõí·ß²9sF½ÁÆápäæær.U²`ºY_Í%KB^ dµZùMY%«þD]]õi6Y÷Ì3Ï°AVÉ*=6++kªYß111MMMÁk222^|ñEv#È*Y»@  ) JV%®n388X^^^XX(ýýýRÐ¥K¨[ëëëeüÚÖÖÆÎY%«À¢VWWgµZÍfó4³¾===&ÉãñÔÔÔHYeãöövJJzê©eËÉBii);d¬W__ÓéAj~~þôçw8Ázhmm²Ê]äÏsßï=ô²JVÅKõçT³¾ÁÁAÎY);;ûÈ#ìIU²,j2Ä|ë­·~ô£-]ºtûöí0DWWWfffÈJÇÓÐÐÀþY%«Àâõïÿþïqqq:Nþ²üÝßýÉdª©©¹ë½FFFF£Y»å®Ã¬U`aR§HIIéèèÐ¡a&Ç,))q8êÐ~¿ýúõ¹¹¹ìUU²,FG±ÙlK,Y¶lYÈKòóóCN;3¯×+cV©²q8s*È*Yööög622rõêÕÿöoÿ6ñ)ÒâââcNOª3y:X,Yps»ÝÍÍÍ:îüùóÚµr×®]²þÜ¹sd×þöoÿ6""â'Øºu«ËåJMMò§Õjå½ «águllìÎ;¿ÿ:Á`¸råZ#Wß÷]F«Ààóùdx*MýÕ¯~¥.e`j³Ù,a¿?Âü?Ãèdõ¾&%ÝÝÝQQQVkâââdYÖÈÂòåË×¬YCVùhxx877W¯×¯Zµêõ×_¾IÆ¦&iÿþý#66VÆ©EEEw=ö/@VgTV|õO>Ñ®®X±"--¬ódRþKNHHØ¹sgEEOC6HIIbG¬ÎZV;ÖÜÜ¼gÏIi½¹Ü°aS=¶nÞ¼¬óHGGGRRRDDÄ³Ï>»uëVÅòÚk¯¥¦¦¿^×çóÉzöÈêlfõÖ­[ryóæMiçW_%'NËCiÛÈÿ³«W¯&«À¼ £ÏÂÂÂÈÈHÁ ¿Îjåðð°Ãá°Ûíë×¯Wo6ÿ§åW»¶¶=²:ûÀ*«ï¾û®ü'«&W¬X!¿uÚË­ódÒd2Y­V¤¶¶¶ß$W×®][TT$·Jq322ä·=<À¬;vLnõÓO?e´<úSmùìÙ³RÊØØXõ²#ö÷÷o,Wm6;x8YV=YRMùQ©:ÂÑ¶mÛ´S«	^ár¹Bî+W×¯_ÏÞBVÛÚÚzÚõüùó~¿ÿúõëdxDdggçææª¡ê|_YYü~SÏ'ÅÕö«®ª÷§xY½qãÆíÛ·¯^½ª^¬4ñ-7êÁRV²<9b·Û¥ gÏ¾Ê õ­·ÞZ²dÉÄ÷655IJÓÓÓ322d¡®®½<ð¬~óÍ7|ò3)))¸©ò«]Ý±cz0Yº¿ÿû¿/..ÍÊÊRù»0qcU_Áqð9Êª¢ÍñÊïôèÑ£Á·ÊpVV¶µµU`vÉ³¡¡¡¼¼CÎ$3wÞyGÆ¦f³¹ªªJõµÙlÚ³ª~V90÷d iµZ×¯__RRât:-KÈkwCÈ­²ü÷ÿñÚúêêjÉ*òÈ*°xI¥2âÔÖeddLZGYY\l0Ôd¯mF£×ë­©©ñx<ÒãIg<´¬Êÿ¿Û·oWê¨ú#//ïðáÃÉÉÉ¼o]òkr®SigJJÊàà`Èuuu2¨5LQ-º2Òûý~ö'ðÈeu×®]j!==]:$Ë.]åææfù7¬³¨©©)777d¥F>vUët:õzQQÑyð³zæÌuÀ©©Zp»ÝÚa÷/_¾¬²HVYtöìYOùJ8F£z7j ())geD+[²»yUíå			*«o¾ù¦¬Q'®ÑF«du2«z¿©¼¼<YnooW³¾!Gx0²ªMïÞ½[ä¿ãçNËª!::úµ×^#«ÀìR)5ÍYYYÑîîn¹_=é+³¾ÀüÎê¤£U&Ø¸qcLLLDDëchhÈçóÊØÔh4Úíö#G°K6ZU½zõ*YîÚÔ¨¨¨Y®¯¯_ºtéªU«îé#´¶¶:ØØØòòrfÕÏ?ÿæçç3	LcxxX©---ÚÞÞ^YÓÖÖ6»ûý~Ç£×ë].×ôÇ0?²zâÄ	5ß+5Y6ï¼óNcc£ÚÑ*0	j|||ÈÊ§zÊëõÞõ¾jÖ×b±0ë,¬©¸Ê?×·oß>yò¤6~ýõ×É*0=ùÅ	Y¹lÙ²÷ÞoÉïpzzzlllqqñÄ3ÏÇYê¬p2<eÏ'ËË²Ü^¯ßºu«vµ¾¾>""âôéÓn<44är¹BN9`ádõÊ+RP	§¶¦»»[Ö>|XåO"ÖF®dññÇKG%¯¾újddäÛo¿=q³ÑÑQÙÀh4Z­Ö¦¦&ö°³:66væÌ#W©©¶æ«¯¾RãW²LEF¨iii&ég©®®¸AWWÍfAª×ëåÔ§ÀB­r`z>¯²²²ªª*¼úýþüü|	ªÓéä0YÇµk×É*)¢Ùl.***,,4ååå÷t÷Ó8Y`g"«gÎÙ0Î=®ùÔ²z÷ª,ÏüvvvÚív¹ºúÝwßíàùç'«xôIåÁ¬ÿõøùd,ü^Ìp)Té1!QVoß¾­¶?ñÐá^^¸pAËê·ß~2ÁÒ¥K_~ùe~xÄ­_¿>ä¥ÕÕÕêhøÓJP%ÉÁgy°(²:Íîó0	ùÎáp¼¦½½ÝétN?À­Édªªªâ0Y%«À¹ÊÊÊà5ÅÅÅn,ÎÎÎG¾Ükpp½,Þ¬Þ¹sG;DHVÕ1fQ¬b!ñù|2îlhhW]]m6zzB6öz½±±±Ìúduª:&°zÉZf´EBn0¬V«zé#GdYj*ÍÈÈØÔºº:¹UZRRÂR²:yVÆ"T^^ÒÞÞ.ËOç8õü¨G'¹·¿¿_6P/ç0Y°Yåp_d i4£²FÆ©[Fn±©j%Ã­­­ì=¬UàOtuueff¬,..xüé¨äVÍúrò¬U`6-ä-1ùùùÁøíïïw»Ýòât:õÈjY?4dÃá~;_F£Ê§ü1ëÕ»8vìËåR¯øMOO×^ý«-ïß¿_®FGGU,x===f³ÙãñÔÔÔx½^í@¾>Ïn·«Ï0ëÕéÜºu3Ø`Ññ¨A«ªªT/+**òóóKKKåV¿ß/U'aÖ «R]]Ñh,..!©Ëå²Ùl!FÖÊ2~-Ù]Y½7@ ¥¥ESÉjµ®RbI§ÓñÅdTz)¿HÚékvv¶ZÖN>ãõz9ù@VÃtòäIèÐÐPbbâ5kÔk$~V±ðÔÖÖFþ³4LÚ)Ç3228å8@VÃ°üoÞÜÜ-qqqF£q÷îÝöìQç^Ð¦¦¦U,2<Y)óeËÉ(¶¦¦Ïd5Ì¬^ºtI²ÚÝÝ=w7lØ@V±`9rÄn·kíizzº<Îe¨Ê¬/@Vï+«ò§díÚµ²ÐÓÓãp86mÚäwøðaÁðî»ïõÕWLcÉÊÊÊÍÍ½xñ¢×ëùøv@Vgí¹ÕcÇIbÊs«XÀdTúì³ÏJP###Fã?ÿó?³O²:ûYÝ°a:.ÄæÍÉ*íä3ò?wî; «³ðÂmÛ¶ÉeKKÁ`HHH8|ø°­­½ë3¯dóvB»ÝÞÑÑÁð ²ºsçNµ²zõêà$®d:ùÑh¬ªªâµ¾lVµv&%%¥§§ßÔØØHV1¯ùý~uòÇ#Ëìs7ZÕa·ÛÕ3¬½½½dóJ+++eÊ¬/9ÍjKKKHVïÜ¹#ë_xá^²yª««ËápÄÆÆ1ë`î²,>>þ7ÞàPûGz÷íÛ700 ­)**Òëõ999|ÀÃÌ*g°Á<rúôi£Ñ%©©©.//OII±ÙlrYfêâÅ=öXFFF «2ZüñÇõãÕJ «ÀlÝºU©jyhh(??_­:îàÁìd¸7¯¾újVVÖ×ã§7Lf³¹©©IB[]]ÍÎ@V­>ñÄÚ)ÇEddäéÓ§Ù9È*pFFFÞ~ûmN¤N9yæeË±sU`¦FGG+**,ÉdúÍo~½téR«Õ*			UU`¦ºººìv»^¯/,,T§AjuuõÖ­[÷íÛÇ«U`F$¢±±±ÙÙÙWv²JV¦³Ùl4kjj8!²JV&y¸geeét:ªªY_ «d÷LF¥^¯W¯×;ÏÇ@VÉ*ÂÔÜÜ¢íÀ¬/²JV¦þþ~§Ó©Óé³¾È*YEdTZZZj0<ÊÙ!È*YEZ[[¥¦Ìú «d÷¥¿¿ýúõ:Îív±CU²pÈ¨´¬¬Ì`0ØívfU²ðµ··KMFcUU³¾È*YEü~¿ÛíV³¾²Ì@VÉ*Â¡fe*ãT­²CU²0ù|>5ë+eeÖY%«ßï÷x<z½Y_d¬ÎÔàà`WW×ÈÈÓ`UUU2BµZ­­­­ìd¬Þ]OOOzzºÍfËÊÊ2¥¥¥<R¿õÍÌÌeÖY%«35<<l±X*++U9úûûívmmíb~Ê>ÉÏÏ×ëõN§³¯¯ß[d¬Î5777xzGæ¢ÖÔÔÍæfU²zÏ«««CVÆÆÆ.ÂGçÙ³g333eêõzyY§NZ½zutt´Ãáèîî^Y-///..^Ó××gµZÕãY_dõHMM=yò¤,466®Rþß7níéé1LÚ!mGGGsrrÏ²®®NÍúÊ¿¢Èê÷í·ßê&óÒK/-¤JCC5//¯¤¤DéÙÙÙ@`1<Ï=Å¬/²úÀ9sfË-²08Á­*òÕÖÖ·¶¶.¦Ê÷() JV%®üf «Ð7Ün÷Í79ÊÒTWWgµZÍf3³¾ÈêìÓ¦vÕÕ+W®lÞ¼ùêÕ«¼páéëës:2HÍÏÏf «Ïç[·nÝµk×8&ð£Íúfdd0ë¬Îäääà&Õ¡µµUÍúÖÔÔpBdÃA LÚ)Çõ@VÉ*Â'£Ò£Ñèp8:::Ø!È*YE¤£RS½^_ZZºHÞ¬UÌ¾¡¡!uÂìììv²JVmÖ7¤ «d÷Eõå0È*YEøúûûsss9!²JVq_FGG+++Åbá©È*YEøºººÔ¬oAA³¾È*YE$¢RuB+;Y%«Smm­Åb1LÌúYEød`®Óéòòòü~?;È*Â1<<ëp8|>;È*ÂT]]m±XFcEE!²0É%##C§Ó¹Ýnf¬"L###EEEz½Þf³9rdaª««KII1ÌúYEøúúúÖ¯_¯ÓéW?;È*Â122R\k³ÙÙ!@V¦ºº:Å"M²2ëdaêëës¹ÎétÊ2;È*Â1::ZVV&#Ô¦¦&vU©µµÕjµrÊq «dõ¾ôõõ¹Ýn	ªÓéäã@VÉjÔ)Çccc-K]];È*YÏçcÖÈ*Y½_jÖ7++Y_ «d5|qrÈ*Yü333eZPPÀ¬/U²&haa!³¾@VÉêýR§7LÌúY%«áëêêr:2HÍÏÏçã@VÉjÔÉg$¨WvU²¦ææff¬ÕûÕÓÓãt:u:]^^³¾@VÉj@ii©Á`ÈÈÈðù|<¦¬Õ09r$%%Åh4VVV2ëd¬©¿¿_r<77Y_ «d5LÃÃÃ%%%AÆ©­­­<¬Õ0ÕÕÕÍf½^_ZZÊa¬Õ0õ÷÷geeét:·ÛÝ××ÇcÈ*YG P³¾¥©©G-U²¦ææfÍ¦×ë¥¬ÌúY%«aêééÉÉÉÑétYYY²ÌÈ*YÇèèhYYÁ`0Í<L¬Õ0µ··«Yßááa£@VÉj8Õ¬/'²JVÃ7::ZZZj4Íf3'²ú ?~Í5ÑÑÑiii2«C©ÃY¤¦'O&&&.°¬JDóóóõz½ÍfG «säèÑ£2¤ûöÛo'0/¿üòüÚï£££ÆqÕÕÕÌúY#·oß×étß÷]Õók´êóùÔ¬¯ÛíâQdu®;vlùòåóxxxXÍúÚívN9du.èþ dttô¼Îª¬Õ¬oYY³¾@VÔÔÔóçÏËÂ©S§Ö­[7O³*ÓÌÌL5ëËk¬>4ÝÝÝiii2N]»víÕ«Wç]V=z­/'²Êá Â'µX,rÈ*Y½/~¿_r<''SY%«÷%ddd0ëd¬@V «d@VÉ*d²JVd¬È*Y¬@VÉ*¬UÈ*d¬È*YU²Y¬UY%«UÈ*YU² «d²Y%«²JV «U² «d@VÉ*d²JVd¬È*Y¬UY%«²JV «U² «d@VÉ*d¬È*YU²Y¬UYeÇÓétd@Vï×íÛ·W¯^MVdu|øá»víRY½sçÎ	V®òË/ó³Õ»¸téRzzºÔTËêË<ùäd@VïÎår8qâ/I`Yîµ5d@Vg¡µVd¬È*¬@VÉ*¬UÈ*d¬È*YU²Y¬UY%«UÈ*YÕ¤°°péÒ¥+gÕÓO?mµZWbÎýô§?ýÉO~Â~©©©+V¬`?Ì=Í&^.ÙsOö¼üÍÝ)=÷YõûýÍ¶¬¬,»ÝþæÜÆüqöÃÜ+**Òétÿò/ÿÂ®c;wî=¿mÛ6vÅÜÉËËÝYWW÷¿ÿû¿ó;«Âo~ó^zéÌ¹ªªª'|ý0÷:::äûÿýßÿ±+æØåËeÏwww³+æ,ëëëçæsU²JVÉ*È*Y%«d¬¬UU²²JVÉ*È*YY%« «d¬ÞÍûï¿ÿ«_ýÇÜÜøÿù³æÞÉ'~úé7o²+æØÕ«WeÏÿÏÿü»bî­Zµªµµ¬0ÏUÈ*dõÑÑÝÝm±X¢££ÓÒÒ?ÎCáA;uêÔêÕ«e;õª~séØ±c:ÿ»uëÖæÍcbbV¬XáóùØùsFö¶ÝnWû¹³³s.÷üâÍªÛíV/Û»wïo¼Á£ðAKMM=yò¤,466®Áû¶üO£e=?gvìØñÑGÝ¹sGþÊ[­VvþILL¼té,ÈerròyÝbÞéòX±±1µÓ1gâââøÌ¥?üp×®]ZVÙósFFüåòOÌ+WdA.Õ?4s¶çoV£££']ÆvæÌ-[¶ð#3òßzzzºüAÑ²ÊË¿3ü±ü)ÙÙùs¦»»[v»<æåRþæÌå_¼YÒcbbxÎ7n¸Ýnõ¦I~sÃår8qâßö?d=?gjkkeáË/¿nØùsfÍ5j@úúÜsÏÍå_¼Y]¾|ùØØesàÊ+7o¾zõ*?¹¤ûSìù9þ;2BbçÏÙ<ÁÃÚó7«6mÚ¿¿,È¥x>h>oÝºu×®]ãGðûÊcóçÏgff²óçPeËÂ¹sçdä:~ñfµ³³3)))**Êb±:uGá2fâGð°²Ê3×¯_w¹ZJOO¿pá;Îøý~©©ìy¹å¹Üó²Y¬²Y¬@VY¬@VY¬Ä_|¡ÓénÝº%~ø¡¬IHHHKKknnv»Ýr)ëwìØ1åï¼N§N¥2uÎóßþö·êêÍ7åª:o(²,·oß>|øðöíÛ%rË>ø@.F£Ïççg¦]µÛíkÖ¬ñ»:tH6Ø°a¶fçÎ²æúõëÚ]:;;evÕ~Y¤;vL"'Ë£G~ÿVµÊ^ºtI®^¾|Y.×®]«VõVÝÝëõ¬úÊ'r¹?ÿüsYóÊ+¯Èò§~*Ë÷îågU`ÞKMM=xð`||¼N2¹ÿþÝ»wKçdÄÅÆÆFµl±XdHªâ*÷±©,È`4--MUëëëeÍ¹sç´»«±éùóç%ájÍ7dTýU`aýÆêt|òª§?ÆðÁÒHUÊàÑªv/uóÞÞ^YèééKuVmYX³fMðÆªÓòqdYxÏ=²FûàÒu~Y¶¶¶±±1)¢z5ªæM:ZUWO8¬ær%À²òË/¿LJJzã7d!øãËíµ×Ô¼±º¯Ëå)´|~YÎUâüJ]YÎÉÉ4«ÒÎ3gÎÈ+W®¨§Eµ'VÕôïW_òÁÏ?/_|ñ6ÌUã`m´*]~Yõ,éîÝ»ãããkkkÕØ1::Újµ6ÿAð$°>UäR6;<N½(I-$&&J)ïÜ¹#Ã<éh5x(¬óØûï¿¯Â&jôé÷ûå²»»[­öj#E5UòÑ$¥W¯^U/¾ûöÄv2ZÈ*°`k×®©÷·È¥z%z¦SÕ±¾¾Þb±ÜK½ÃuÓ¦MZ:ÉßF«Yõî¡¡!¹Ü¿¿¶>**ê^Rª¬Ê­ZµpìØ15íííK³ª^ªNùwÑ*@VÅ@½TSÕå;ï¼£f¥¸=Ã^¿~]RRªeUVâøñã÷:Z=þü4IÈ*yãòåËmmmÚÕ+W®ø|>í`êm©999ÁcÙ¹:::*9W¬Xát:ÕM@@nJHHÕÏ?ÿúI¶­©cJðãÈ*°¸¬²¾¾^»zôèÑñËð7dÀª(UfHUÈ*dUÈ*d²È*d²È*d²Yd²Yßÿÿ5ÄáÖSÄäIEND®B`


糾正常態 Q-Q 圖


ýë­ «÷¥»»»ªªª´´´P(?~|ÈÊ*²êë UY@VedUVUY@VedUVUY@VeYUUY@VeYUUY@VeYUdUV@VeYUdUVø®]»öê«¯Î?¿¶¶våÊ_|ñ¬Ê*c´|ùòhê'|òÙgmØ°aÊ)'OUY`Ô>üðÃÙ³gÇ5[òÆo,]ºTVeQkll|ûí·óK"±1aUY`,Yë­·òK¾þúkYUÆâÃ?1cF¤4[òê«¯Ú	,«ÑêÕ«gÏÝÜÜüÑG­2*ûÙgÉª¬0Fï½÷ÞsÏ=·|ùò6xqüÞYUdUVUY@VedUVUY@VeYUY@VeYUdUV@VeYUdUVUYUdUVUY@VedUVUY@VeYUUY@VeYUUY@VeYUdUV@VeYUdUVàQÍjOOO¡P(++«©©éììÌ_tüøñ¹sçÆEsæÌÕdY½úúúÖÖÖìÜ¹sÅùfÎyìØ±ìÝ»wÖ¬Y²¬ÞCeeeúúúª««[­¢¢"VûÍ UUUO=õ²ú²²²!Çy'NYidõÅAjjjdYýÒÒÒl^>x7oÖ××ßºuËN`dõ¦NÚ××vÇ¸èÒ+W®¬Y³æêÕ«GÃ~¸víÚåË766~ùå6²ú544´´´Ä NcV¿¨««kÉ%×¯_÷x4¼ñÆBá_ÿõ_?þøã_|ñ±Ç;yò¤Í¬>HÝÝÝUUU¥¥¥ñíøñãº%ßÝêêêYqí³Ï>Æi¶äí·ß~â'ldÕ×A£ÔuëÖåôööN2åë¯¿¶qUYFçwÞill,ZX(¾úê+YU`t>ùä¢¶µµÅ[YU`,V¯^][[ûÑGñÅo¿ýv4õü£Í¬Ê*0Fo½õÖÂgÏ]__Ïb6²*«Èª¬¬Ê*²*«Èª¬ «²²*«Èª¬ «²²*«Èª¬ «²¬Ê*Èª¬ «²¬Ê*Èª¬ «²¬Ê*²*« «²¬Ê*²*« «²¬Ê*²*«Èª¬¬Ê*²*«Èª¬¬Ê*²*«Èª¬ «²²*«Èª¬ «²¬Êª¬ «²¬Ê*²*« «²¬Ê*'O>÷Üs³gÏ^¸páë¯¿ÞÛÛk²*«Å_|1eÊ6|öÙg]]]/^¾|ùÃ/¿üríÚµµµµO<ñDccãµk×ü#¬Ê*èk¯½JE±ÞÿýöEÀc=^Äì¥K>ä/@Ve¨Ô×__òÎ;ï¼øâãâE@x_¬Þ¯7o=zôÀ%%%·oßUÆuV¿úê«ü·ß~û¡Íêøz²zoÑÑíÛ·Ï1cÕªUûöí³û÷ïÓxÝÑÑÑÜÜãÊ*ãÅsÏ=·aÃììµk×æÌÓÖÖæEðeuÙ²eiO=Ó¦MKã³ö÷÷_½z5V8|ø°¬2^|ùåñ2qåÊôÑûï¿M]½zõCûne¼~Í¿Û9öl;a|gõå_4iRLR***b;;;c°eË_¾|YVG.^¼ØØØø»ßý.f®ÍÍÍùaÀ¿üå/Ó>ø ¶¶6ÆYñÕÅ§ñ¥KRAcá¦MbÂÚÕÕã³gÏÊ*üH¾þúë×^-¦­uuuï¼ó¦Â£ÕÇå4Ø»wo9ã.È*²z_YMMM7nL´ðÔ©SiáÍ7eY½¯¬Î?¿hæ3gÎÜºuëàÁÞ[@VïKTsÂ	mb<yòä4¾Æ ±±1MgÂ³Ê*WV¯^½Úßß>N	Î>]sìØ±û¿B¡PVVVSSÓÙÙ9òEc["«<ìYMnÝº¦¤çÏÏ^ºt)DØÎ;wÏk¨¯¯ommÁÎ;W¬X1òEc[rb¿û»¿â'>àW¿úÕÌêéÓ§#+W®òÒôÞjuuõÈWRYYf½E+¾hK¾ýöÛøL4éeuúôé,y3gÎìß¿äubR;äxÈÆ¶äÿòûßÿ~Ñ¢E_Àðë_ÿú¡û6¥¥¥Ù¸¼¼|äÆ¶Ä«÷VO8±l@ý¶ï¥ñ=bã®aêÔ©imG¾hlKdñÕ;wî¤72ófÝ¶mÛý_CCCCKKKâ´(À/ÛY`|duÈïUV»»»«ªªJKKÂñãÇ³+ò¢±-U~.YõuÈêÿ×ßß)]¿~ýà¬¦÷8eYã5'²Æ²¬=«v «²²*«Èª¬ «£Ëjoo¯¬ «÷vøðáºººtÄommmvôo6niiI!NVÕ¸ûöÝñIVxè²:~É*²*«<êY½víZúÂ3fÌ5+maIIÉ#GdYcÇED/^¼XYY9oÞ¼t<ptËlYÝGkæÏßÖÖVVV§&MÚ¶mÛ;Òß3ÐÎ9SVÕ¸téRdµ§§gäî.[¶LVÕOU-Z'OÎ3§¡¡aå	&¼ôÒK.øKéíímkkã7Ï?oðPg5ÿ½Ø|A½·üÅEGãÿüùó7lØðÜsÏM4é£>²Y7Y]¶lYýô]KkÖ¬Uà/háÂÙÙhjÕ=«QÐW^y%NÛÛÛ'L0yòä8ÛÜÜ|Ïw^eøxqÚ´i½½½ù1g§&=«o¾ùfÇxîÜ¹ùâ,«ÀOïÓO??~ÑÂW_õõ×_·qxØ³µ³ªªª¶¶6ÑÞ½eøéýõ×wùFh?üðCq3[Í¾böìÙéÖS§NÉ*ð±nÝºèèÉ'cüÕW_½ðÂñº¿h·0<Ymoo/Êj,_°`C¿ ×_=æ¬Ó¦M2eJ]]ÝÅmö¬æM8qÅ¾jx¨?Þ$qUÁYUx8²ZRR²zõêìì©S§bIWW¬ «£såÊè'òf«Èê(ôõõmØ°aÒ¤I+W®L¥Ù»woúëåÕÕÕÙWæ'²²¬-fß¾Ù$5ý1óU«Våg®Ï>û¬¬ «÷õ®jö%J1N_|ùòåüÛ¶mUduÔY>úõë×cpûöílá;dY½¯¬Î93ýÝò4[M«ªª²¶oß.«Èê¨g«© çÏññãÇÓÂÍ7Ë*²:Æ¬;wîd·nÝ*«ÈêØ³:ä·Uduï­8p`ð'p	¬ås«ï¾ûnÚÕÚÚº~ýú¢ÏÛÈ*²:´ÈgTóÌ3éì7ß|¿t÷îÝù]Ä²¬ú6ðgu×®]ùoÛÏÞvUduÔ¾ùæü·ßºu+ÎîÞ½ðÎaY@VuÞB¡­êMmhhAkkk,X «ÈêÓ®]»¼·¬Åõë×¹éæÍÓXMdMSÕ¦¦&Y@VGñ÷Ì³Ã¢UEöööÊ*²zoG]1 û¢¥¶®ü^,Ù³gO,,--Udõ~ç©iª:oÞ<[@Vèl.ý18Y@VÇ.JõÐ¡Cù=Àé¥ìKeY½¯¦¶´´ô%%çÎ3[@VGáÚµkQÐcÇåe5ûæ²¬ëöíÛÏ¾¾¾Èê²eË²ãbùÚµk³Ã¬ «÷ûùïZrÈ²êÃÀCÕ'>ùäõ´µµìÙ³GVÕûÿzýHé´iÓ²/ÚÛÛeYEV#¥m^zé¥8ñ¾ûâ¢³gÏÊ*²ú`f«ø1ºØÓÓS(ÊÊÊjjj:;;ó?~|îÜ¹qÑ9sb5Y`euÖ¬Yé¿üòË&LxåWbÜÞÞþùç?FVëëë[[[c°sçÎ+Vä/9sfú(íÞ½ãÉ*ã,«/_Ógy&[²dÉ;wîìß¿ÿGÚ[YY>ÕsâêêêáV«¨¨Õþ× Ó§Oê©§< x¸²zàÀì ß©S§¦wXc<yòälß¾=E÷gµ¬¬lÈqÞ'bVYýý ?þ¸¬ðÐeõöíÛÙµ®®./[¶ìG=æ(ÿ7ËË¯póæÍèý­[·ì`Üd5¿+øÇÎjÉ÷b3ãô×^ã4ÆEk^¹reÍ5W¯^u$0ã&«'NX6`¸Ài¼páÂØÄiÌJóuuu-Y²äúõë>`ÀxÊjömÀ·nÝZ»vmôÒ¥KqöôéÓú1ÞRÍtwwWUUìÏ§§luuuI¬0>²"«ÍÍÍé0àüîGqwíÚËkkk²zoÓ§OO;ctóæÍ(ëªU«dYõl@VeYUdUVUYYUdUVUYYUdUVUY@VedUVUY@VedUVUY@VeYUUY@VeYUUY@VeYUdUV@VeYUdUV@VeYUdUVUYYUdUVUYYUdUVUY@VedUVUY@VeYUY@VeYUdUV@VeYUdUVUYUdUVUY@VedUVUY@VeYUÕ»wzzBYYYMMMggçà>"«Èê½Õ××·¶¶Æ`çÎ+V¬(ºôÎ;sçÎÍ²ú¿ùû¿ÿY@Vÿ¤²²²¿¿?ÕÕÕEnÞ¼yëÖ­)«ß~ûmÉP~ûÛßz@ «ß)++r.]ºT[[ÑÍf«òì³Ï­ «RZZËËËóÕÕÕ=zô»å½Udu8ÙþÛO:µ¯¯/íñ«e+Ë*²:Äiýp6[@Vï­»»»ªªª´´´P(?~|ÈÊ*²êë UY@VedUVUY@VedUVUY@VeYUUY@VeYUUY@VeYUdUV@VeYUdUVùòË/ÛÚÚþøÇ?^¼xÑ@Veuì^íµI&ÕÕÕ-_¾<ï¿ÿ¾Ç¬ÊêX¼÷ÞBá³Ï>Kg?ýôÓ)S¦ÄÌÕC@VeuÔ~÷»ßðÁù%o½õÖêÕ«=ÔdUVGmÎ9_|ñE~ÉÇ¼téR5YÕQ«««ï½÷òK^õÕ^xÁC@VeuÔººº¦LòÉ'¤³mmmqöäÉj²*«cÑÜÜ<aÂÚÚÚ9sæ?þØã@Veuì®]»öé§þçþgoo¯¬Ê*Èª¬ «²¬Ê*Èª¬ «²¬Ê*²*« «²¬Ê*²*« «²¬Ê*²*«Èª¬¬Ê*²*«Èª¬¬Ê*²*«Èª¬ «²²*«Èª¬ «²¬Êª¬ «²¬Ê*²*« «²¬Ê*²úÒÓÓS(ÊÊÊjjj:;;óÝ¾Í5åååÓ§OïêêUdõêëë[[[c°sçÎ+Vä/jjjÚ²eK4uÆ²¬ÞCeee3ÕÕÕùbþzæÌìl¬öü ³fÍzê©§< Õï9Ng·oß^QQSÕS§NEVä¿ø¬ «RZZËËË.jnnÁÙ³gkkkí@VPò½O:µ¯¯/íq~µüÙ¢¬¬ «ChhhhiiAÖ××ç/Z·nÝ¾ûbpúôéùóçË*²zÝÝÝUUU¥¥¥BáøñãÙt6NoÜ¸QWWóÔÚÚÚsçÎÉ*²êë UY@VedUVUY@VedUVUY@VeYUUY@VeYUÕ?ÿãqõW5ëÁ1cÆã?>áM>ýoþæoláüò¿M§6ÅpâñÈvA<Åsí0¿þë¿~°ÏÕYýÎùóçw?P3gÎxñnWVV¶fÍÛa8MMM%%%6m²)óÿðååå¶Ã-ZOô¶Ã¦MöôÓO?À+|ÿý÷ÿû¿ÿ[V¼x4¯_¿ÞvAEEÅ|`;çÌ3ÕáþB0!¿bf`;àþé,Yb;àW¿úÕ«¯¾úü"YUYUYUYUYUYUdUVeUVUYUYEVeUVeUVeUV|¿ÿýï7mÚd; ¦¦æßÿýßmá?þñÇÿ¯ÿú/b8ÿöoÿö·û·¶ÃþùÿyÕªU¶Ãêêê¶mÛ&«0È*È*Èê#àôéÓóæÍ+++«©©éîî%W¯^-É±Ãg[£§§§P(¤ÖÙÙiãm¡"7Ð=7GQÛ·o¯Y³¦¼¼|úôé]]]?Ù£È¦µø÷8pà@;VYY¶¶¶øÇ³eòîÜ¹3wîÜìÿvkkkvîÜ¹bÅÛ§hûx¼A<î¹<455mÙ²¥¿¿?:cÆìQ$«cwèÐ¡9sæÄ Ê)´d6oÞ¼uëÖ,ñú#Ü1èëë«®®¶¶PÁÄCèÈ£hðèÌ3?ý£HVÇ8Õ8qb<'îÝ»7ÎÎ9sÉ%eeeµµµgÏµ.]º"¾Y6bãdæÇ¶OZâ!Tdðñºç&ò(*bûöí1U=uêÔOö(Õ±;|øðÔ©SóK®æ¯?suuuGýîáõ6JKK³KËËËm¢íã!4lxÝsyÇLsssâEF¼ÔøÉE²úC_-ñ>Õ¢èÐxýÑ××ww`ßKÑkÛÇChdixÝsyÉ?NÒsõOó(ÕQ9sæéÓ§cpüøñôma±äÂéE¢ï+êG444´´´Ä Nëëëm¢íã!4øYÑñºç&ò(*²nÝºûöÝøøÆüùó²G¬ZOOOMMM¼öY´hÑÕ«WcÉ'fÏK.hhp6º»»«ªªJKKB¼±e¶PÁÄCèÈ£¨È7êêêÒÍé;·G¬¬¬¬²²²²È*È*È* « «À9r¤¤¤äöíÛqºyóæX2yòä¶¶¶úúú8åMMMÃ>/¤¿ø1ôgØ_~ùåtöÖ­[q6ýyKUàqçÎ7FäöíÛ§6mÓI&uuuåÃ¹÷îìììÙ³çÍ·r@üøbeËeKÞ|óÍXrãÆìGº»»cIög@RVÛÛÛý «À#%&©ÈEbãôÐ¡Cw¿ÿÛéùÙjVÙK.ÅÙË/Çé¢EÒÂë×¯Í_DãÕÕÕÅîÙ³'<ýôÓ1ÞµkWwîÜéßYÆ½3gîß¿âÄQ¸ÈdKKË¶mÛ¢s1ãÌGqïÞ½ibJâ?sÓÄd´¦¦&MU[[[cÉçýx>:Ü¼y3Demdx´þW¼ûî»inÞþ<`Ó¦MÑÈTÊül5û©ôgÕO:'OÆiúãÏ17o^~åÔé¸§ïØ±#dW]÷¬öõõEÓ¨×®]ëííMÍr¶Î=z´ºº:íËÇÂ³gÏVUU­X±"ùë+ægÒ~ãô³uuuEëñ¬Î5â?R7ÆË/2«ÑÎ'NÄ+W®¤·E³7VÓîß.]ùéÓ§ãôÈ#Ù47Í³ÙjLvý+ «À£ ½KºmÛ¶'677§¹cYYÙ3Ú¾ß	³ÏTÇ3gÎÄi¬Ö1 QÊþþþ¸òçÃ<äl5?YÆ±?üá)lqfçÏÓ´$.0aBv´QÅ-º¶HéÕ«WÓAÂwîÜÜN³UUxd]»víúõëéó-q$Jït¦:¶¶¶¢JpmhhÈêoäÌ3xî0[Yôé/ÆiKKK¶¼´´tÁQÊÕ¸4BN3ÑS§NÅiÌYÓÞªûÜa¶²?é#¤iN×¯_öGq#)E_4xãÆÈHiÕôµ£­>z$¬ãÆåË<½råJWWWöeéc©Ë/ÏÏeÛÛÛãloooä0V>úÒ¥KÓE×®]&O<8«öìIG?ÅúE³Õ_|1§døy5kVkkkvöÐ¡CE_ÄÓß¢	kúZÄ¨²))È*È*È*È* « « «¬¬¬¬²²²È*È*È*<êþã·9õ®,IEND®B`


ºdZZÆ÷ïß×¸¶¶ÖUÅÀZè¸V®úûûõ£¡fµ¡´´Ôº°Ïç3cccËÊÊ¬+644h¡Ò>ÔÝ;vì.ÐÙÙiÏêPk@öîÝ«³ô ÚÛÛÍËëÑéÅÆøÃYºzÔa222´>G£¿Ê'OtôêÇÜ«¥K:jÚÒÒbÎZ¹r%ÿ×@V1ý]½zÕÌN2ÏÚúñæÍG1O÷·oß6ËO<©SM[¿EØz"6Lùjjj^ÞUýè¿hù·ß~k¿¤f^¨Ss?uºbÅ]RO>ùÄºðÓ§O5W6PätÚÑÑ¡S=ü»F]ÿKTTþM³äÑ£Gjí§~jnZ§Û¶mêkÐ:HgÏ9.vãÆ-×zVãuî½÷L¨Ì+!½,PÑò³sÑï.Äý±ÿÌ«W0ºæ±<xÐñ0õWAVAV1S	¦h?Ó2Ó¬ÈÈH«%?ü´XË­Yì0W×³öüùóÕ0MÍæ¡­[·ZSÍ7;îÒ-[¬³LÍlÏün·Ûÿ¶ºººtzñâEs¯¿þÚT944T¯VYÍÅ*++Ü«YÚ¯ÍÎÎÖf°îªõrDË­Ùª¨©W®Mhù×vîÜéxøË/×µõ@¬)¾~Ô´þ¢EÌåõ¯ªi«ti­³Ì±ÿÖty°¦Öººº«õ¦~fÿX¯âÈª^lÕÙhYYÅùó«à0YUxÌå_|aofz25Ó2¼aÊªs?ûì3kfõÜm¿Ì~8ÔlÕÌíÏò[4í4ãîîn3mµ¨IæObÆÇÇ¼&«¢©è¡ôÑG«éaÚ³ª¹£Ùþ©I°	¶. ª&ÖÍ¾Ù<Ò¦¦¦)ï¾û®õ£Ö³¾©TEEÎÕ¡üü|[½4Ñc·¿glg.vøðaûBóëÓ·>Ö«Ø³jm°V£ÙLVAV1#h¦¨Ø8:²jæp)m¿?üôöÙ>ÙÑÑa#ëZO¯Ö>AÖvcó¤l¿ÌÞ½Ïªæ|999?nwµwô6k0k`.àØ_F	´Op5ËTÉì3k£«¦¶ºNÍ¤Ùþ¨5·S&54Û±u®·BwÌ>«6oCêÖªî°ý®êþhz]TT¤Ë¨îf[±õÞêÝ»w5NII	ø+NLLÔ¹wÇÍINN«yþüù5¬ÇbÞd5cófÁð[²úg¢	æ(æIPN³¡ÕzcÕ>ßRnÍ´Z>þ|õü«±Z¢4ïq*®gÏ¸­#«;ê(öûgÕ±Øëv9¢ãE-IJJ2Ñößo6aõ£Beî­`ýhö<2ïøj¨rØ·<_ºtÉÚeIÓYO³FeØdÕL+E­¾¾~øõ0ÔCkki½ÑÅÌ,Ù¼©I¡fþÛ´U<ànPÃì¤6Ö«X´Nú­¬bfill4²³USSß k· ·¸7Òû+	Q'ï¡§æÂÂÂÑÏVÍX·p?X½bPï5»23$]F0³C¯uÓºõ¯Ù?ýRPPàxÓWë.Kêy *½4[÷¡ÖãmÈ6µr³ÕU«VYÑ¤?à¶5R½xëã*æ!kÒ¬³hÑ"ë±h¶j%%%dd3YïSúgÕþùNsáÛ·oëY^Ó§O]SÙç»­CÍV­ý4ÎÍÍµ¦¡Öû¯þYÕUtoõïëÖ5%þ¶L#5¨®®f¶j.ÓÖÖf¦YfVj6]6(Zf.nõþÑ£Gª Ùb¬~+lë!8öæ5Fxß¬©Ì±¿»l®D±1k¶jÍeÝºuþ5âããÍåýÛaÝË(¯0«æ>hê[kVÙlnÞö&« «æ?fM¬iµØ>±³¶²¬¦§§«»fÏXó¸ÅL¤Þï½af«ö¬fdd8.`vN¶o³UV].×áÃÍÓ¬Èìõj.i%ÇÁ|BÆÄÙªù§LTk3/ß·o~,..Ö­Oª+ Û©é®õo	¨ýVìs_+fÍ[1nÒXÑ¼ìXcº^Üs·lÙâØï)àq-eÞÚ«ÐcÑk ó³~G¬æ*CeU«NB«Úü³æW`Ö3YYÅôçø çP³Uÿ©­ÙZÿþ³Ãf6­­­ÖÖ¾²ãËªæfgcóãòåËÍDJþð7ëLhõTnÿ4a2¯¬ÝG­y¦YfÎj-7[qKJJôªÂ|Ôþ9Tû4;7Ù÷½2½téÿ°¿°¯ÇCóføÛ»fVmîl¶]ÜÁØl¥×«ýËÍÍÍf&jø÷~|WñÏªµÃ,$« «ÑFU%ÄLÌ¦È§Ì3¯µGÏ0Yµ¿·j«Ò0Ç?²>¡×'k»±Ùª)£y»×ìëx®·>3:â.K555zàf"¨fþj^4yÙÇñÑÇãr|ÄÅÜ1ÿ:ÓèUëèùyDöW$f×*ô(++êM7oö?¶CÀãDï*ö¬êõGLLLJJùå)þ¦MÈ*È*f.3c*«æÒKa3°RjÜíOûçV­©§þÇcþûÁoý¯®	®yÃÒÊ­`9ðdÿPMÀØØêdmûÕÅÌ_½t°ï]ÜÑÑanÚg³yÖñohh¨õðð³UÓ¼­kßl>,dZðØæ­nó¡VÚÁSSS].Wbb¢µy/vø_Óè¯âÈªÿÝûè£È*È*f.³ßéPYòäcý¨gsÇÔj4GY²övtÈì¬pG4s,w1Ñ<×ûgÕaØìßk>öúÃàG?í~5óÖe4c[µj¹?×*ÍÑöO¨@Ö7o­ÓÖ¦lÇC0;gY?X:x¬ÍÊvv¶úhýh2<ü÷ØéþëÑuTÅ±^Ål¨X¸páÚ·>òUÌ ´EEE½èÂqØaÇû¯öÃ²­Éº9ÿ[Ô&ê£Ù?<ú4NfeÏ@ÿ0Í´ «UÈ* «UÈ* «UÈ*dUÈ*dUÈ*dÀôQ__cýxòäÉøøxV@Vá¤¦¦Ü½×Z¢±¤¥¥ý0øØ2Iÿ-'â¶:::V®ºiÓ¦¾¾>Ç´¤¸¸8|Ï=æþìÛ·ÏþãæÍùÈ*0²²2cÿþýÖÏ>ûLKvîÜ9	YmmmÍÊÊÀ¬êUzûöíöövýk[·nu´´TËkkkkjj4øäO¶ùôéÓ:÷üùó:mll¼ÿ¾K.mhhX>¿¬üdffZK-ikkQf¨Ãüã¶:G÷ôéÓ'Oh°xñbÿ+^¼xQsYëQûMÍ'NDEEñU °øøxeãñãÇ÷ôôhl½hÏ755%$$DDDìÝ»wß¾x<ÍêÌe«¼¼<ÅLY*))yþü¹ýº>O×Õ?~öìYk¡uþ·å¸üzÕþ@,ºcö[±ò9LV5[½råunLLLÀdÀêêjujßvê:Õ´»»ÛwîÜÙÕÕ¥âg.³mÛ6ýxòäÉúúzÊÊÊì×­¨¨¸qãýòþÿþ0	$à#êëëKNNÖ¹Gqår¹ì·âv»GÌjKKËÝ»w¬¬jÊË@VÀ4)T<²³³5~÷ÝwÍC¥ÎìãcÆýýýf¬VËÄÆÆåÌ?ß~Ýo¿ýÖ³á³êùQÊÉÉÑµôXüÏeVÍk¥TM|Í4Ðz?Y@MÎMòtªlhÉh²ç?6Ñ²X¹ë¿p<ÊÙjee¥<ÿsÇ±¸®®ÎÔ4==Ý,!«Y§ZX§ÿû_e	ÒØªò¸ÿ¡Æ#êîîU;::^`ñâÅf%å.K&«÷îÝëíí5KV­ZÅß@V!©ÖüïäÉãÎaAAÙi¶³³SEùÇåÕ­[·êÂ'Nªë;wîÔ fÐP°	Uû?Ãß@V!ûí·fû­N<y2î¬Z[ÐqÍ5÷ïßþògÏÕ%W¬X1QY	¸ØëæççëFÃÂÂtWýñÃï­jµèÞZdåo «F¥¹¹ÙçóY=¾té÷ööÚ÷Ã@V «UÈ* «UÈêäOúS\á×¿þukk+YýñÐ3YYYwø~ûÛßþ÷ÿ7Yý1«kÖ¬ù+?Ã?ÿó?U² «d@VÉ*¬UÈ*YU² «d@VÉ*YU² «d@VÉ*d¬È*YU² «/tVÛÚÚ<ÛíNMMmjj²ÕÒÒ²hÑ"¦UàÅòðáÃâââ¤¤¤ÈÈÈ%K9su²t^¯·¶¶VÊÊÊÜÜYÉÉÉW¯^ÕàÄ.üþûïÏûY½z5Y¦ ÞÞÞ;wîèÇS§NEGGë5²11ô÷÷'$$u±°°°ï¾û.$·ß~?`ª©®®VVWkÉ¹sç<kd5¸ÜnwÀ±]kkëÖ­[5xæçßþíß­SPIIÉ¡CÕo¾ù²D.Ëú_àéÓ§^¯·¯¯÷VHiié=ìK4s~øð!+d5bccûûûÍF`ç>xð`Ë-=bO`àÅrùòå¸¸¸®®.kÉ®]»233Y3 «ÁWSS£N5+µåóùV®øñc>`¼ÔQuÇUUU999)))·nÝbµ¬Wsss||¼Ëåòx<---!?>û®IdxáèyJY-,,<pàïª¬r8Y%«¦ª?ÿùÏo½õÖo¼±aÃ¯¾ú²JVÓæÍgÍµlÙ2<Ï+¯¼rùòeVÈ*Y0fjªf«ÖõuîÜ¹¬U²`ÌÞÿýäädûo¾ùF¡ÌMÁ½½½uuuöì©®®¶øgDçÎÓµªªªØ¬USÂ4=u,t¹sîÜ¹QZZºqãÆÈÈÈÑý¿§§gíÚµIIIºVAA®¥$óÛ$«dÀ/¬¢¢ÂívÛ?fóÇ?þQY´; ¦Ø' ³gÏqöYTT©¸¯]»¦²ò0Y%«~y¯¾úªÔØØ¨ñÇ<kÖ,ç=UMLL´ú_4gõ?p±æ©×¯_·/)Äo¬U¿0MUßxã_~YA;w®Ê:i7­YfFFcáöíÛ.ö§×WUUòÛ$«dÀÌõðáCÒ|ù«%==Ä·WããÇÛäää8pUJVÉ*)GAMSRRÂÃÃÓÒÒ=¼ÛÒé&ÚÛÛÍ¼9??ßñ°]¸p!::úÜ¹sÜ©´´tÁ|Y%«¦¢ìììÌÌLóæ¥&AÝÏv×®]³êVTJM:»»»Gs-Å>..ÎãñèZYYY)ïeÈ¤¹5¯È*Y¦?M¬lÍÔPöívuu8Iõ§ÛïêWUU¥[¯ÒOÜU²Lsåååþ»ÔjR8ÊIäP)?þøc=üå/+V³5U°h=/Y²?9²JVé¬ººzóæÍÈEGGOÚ¤ð«¯¾3gÎìÙ³-[öÚk¯Í5ëüã4X±ò_±-É*Y¦³;wî(¢öIÕ®]»²²²&í$%%©¦VÅÕTuôsÖÎÎÎ©ù%²¸¯¼%«dæ?n½¸qãFun2wzé¥NTeF¼âÇüÊ+¯XÀj]Òýq¼iîÜ9e¿7²JVé¯½½]ÔÂÂÂ=öéð÷?êèÄÌÌÌßýîwÃ_±¤¤ÄÚtéòn·»££cJ­U¯×kíb]WWêÔ)þØÈ*Yf¢Í7Ï7/444>>¾¼¼<x7¤:Ö××ÛhêüÁ­9sæ8.£àT"RïÃÃÃ,Y¢²òwEVÉ*0½ùæê;._¾l¾ö|ÄÎ=fÏmÞÜU4½åWF|RwÉ±Sîçë¯¿Îï¬USKmm­Ëå²oP=tè2¼mÞxã_~yîÜ¹æk§ê;.¶lÙ²·Þz_Y%«¦6h¶êX¨ÚõÁÎÎNÅ[O©£ü`&µVéX_/øõU²`ÊeUÓGÇÂ9sæL©ÝmTßW_Õívkª¦:>$²JVL	ó½ôÒK¹xÜ***Þyçõë×³-Y%«¦.ÿ]~ÿûßæ×®]+,,ÌÎÎÖ©ÏçcM¬U?zÿý÷£££×yóæu4Áºººµk×¦¤¤èôøñãjpddäöíÛ/P^^çøT¬US]]m"ªÉ¨Âùùç'&&*·nÝÒiRRRTT½£¹ª²yÈ~U²àG*SNN¦w *]æ¸<SfÇD´ªªêW¿úÕ9sì÷S¡u¹£*Àd¬ª½½]5Ý±cÇÃAöìÑ_~ùeoo¯Î2_öjÆ©y§=¢öÙ+¯¼bÿ0îªZëèÆÙd¬Jíq|Õ~Ôó&¯iiiK,Qe­«jB;É_åæÌM¦K¢¢¢tO¬%ºK/¿üò1Vnù;^EéSaaayy9[ÈÉ*Y&ÚéØêáÂ%ÊJ©ÏçSYÍºpFFFxxxUUÕdf5;;Û1é¥þë¿þËZrëÖ­°°0½Ðÿëà«zAPTTÄï×N¿Sý*Íy½ÞIþ² ²JV!++Ëñ¥f¹¹¹			ö%ßZ¡U´<	Ø$PDrûUz-ùû¿ÿû.Kº­¦¤¤Ì=Áúñß|=¥¨ zÙñ~µ-Y%«ÀP^^®	¨µ]WM6Ù/³eË_ýêWö%î¬]»v2ï¤%M¹=j>`£®ZµêÅý,Vøúõë_õU=ÀßýîwÁ;êEuuµãPPºi­ÉIÞ¤OVÉ*0ýåääh÷ù %KÌ?ß¾ö,]ºtÑ¢Eö%Êz6wÒúNÏ;7mV~||üÜ¹sKJJvìØ1oÞ¼9sæ|õÕWAziâx]y¬U`â:uªxêÕØØ¨Iµ³Ï;wþîïþî_ÿõ_íWØØ~øóiú¨¦Ú;ª9«^Ùã¶ô+Óûq³1ßY%«@ÐUUUggg½ÞÈÈÈMk¬w7>|¨§~MmYQ?Ók¯½¦yªImm­&¬Aº¹ÌÌÌ7íÌzÙvèÐ!~d¬¡»»»nÙY´¼¼Ø¢¢¢ÒÒÒ¤¤¤üü|vúù^õÕ;vØ466Î=;H7§×CÊª~z¤Ó]»vñ+ «døÅh¶ªÉúzîÜ9:!Þzë­yóæÙ¼ùæÂNÂk&~d¬n¾úê«9sæhÎZ[[[__ÿÆo¸ÝnÇç@VÉ*Vgg§j:gÐë¯¿þ¿üuBVÉ*üèÐ¡Cë×¯ß°aÇ"&«dÆ¯§§çÕW_u»ÝË-ÓìsÖ¬Y</U²ãi#IÏÎ.ëã?fÍU²L+½½½G-...))9sæ+$HBCCíKÞÿýäädÖY%«Àô¡ÉSFF9tCyyyRRRAA¬Y³f9®k>éË!«d>òóósrr¬ª²)))ö/%ÅD3gcï²eËRSSY3d¬ÓÇãq|¡æÑ£G_		QRRâr¹4CýëàîKï¿ÿþK/½T__Ï!«d>âââ|Ï;çøJpLÍVÝn·â:kÖ¬¹sçÒT²:ÚÚÚôêXX©©©MMMÃ5¾%dbýúõÑÑÑ³gÏ6¸®3++Ëq,õ7ú;&Pggg¾Ù31«^¯WÏPTVVæææÖø<÷óïÿþïd£×ÓÓ3oÞ¼Ë/¿óÎ;[8w>mv-22òÀzÔß|óMqq1ß¯	¼HYÐ ¿¿?!!aø³Æ±ä»ï¾ ôtÊeÕív<kK¾ÿþû?o¿ýö?þã?V£óßüæÍ7ßt,ÔuïÞ½¬`&SJ¦].5þ¬ñ-á½UüLË-çwUÞØ<å²Ûßßo¶Ùj<üYã[BVñ3¿üòËÖõë×Ï;5Õ)Õ¼¼¼têõz?k|KÈ*~¾7ÞxãW^)((Pbß|óM>_`fµ¹¹9>>Þåry<¿Ý¡go	YÅøàæÍ·dÉ¾5SÍõë×:ôùç_¸p!¨7ÔÛÛ»ûöððð´´´êêj²Êá È*iÅL¸¨¨¨´´4111???xqÞ¸qcff¦*þ×ÁCèµ¦ZNVÉ*Y0MhzªÎÉÃÍ7%ã¶._¾¬Ú¿! ½½]ÓVÝ(Y%«dÀtPXXè80&YYYÁ¸­ør+##ãÚµkd¬UÓAvv¶:j_rëÖ­ã¶ÔoUÜ±0%%åË/¿$«d¬3Èªªªµk×ã¶®_¿mmpºº:Ç3c¿¬UÓæ¦qqqÇ7?^»vMÞþÀåååú÷5mÕMlß¾]uÌÉ*YHO=:µ··³B/_NLLLKKËÈÈt¼Õ¿êýUëoÆnþ%«d¿]»véÉîÀÖëúºº:VäúõëªÎØrÉ*YÅ4§'8ÿw¡4°8Y%«À¨hZTTäX6Ã7d¬ãÌjqq±cá%KÈ*@VÉ*0f×®]ëîî¶ø|¾èèh6d¬ã¡ÙjJJÊ3gnÝºUUU¥¦=zÕU²Ó¡CV­Z¥¸®]»v&¼ «d²JVd¬È*YU²JVd¬È*YU²¼à®_¿~üøñ.ðÅ&Y%«Àøõööæçç<7feeÅÅÅï«­²JVÉ*¦¹ââbÕÔ¤9sFe½uëk «dÓÇµk×³³³uªÿÁ»¡ÈÈHGDu»víâWU²iâøñãgÏ=.á¢££´aöo¾ILLt,¬ªª*((à·U²éàáÃê¨Ïç³;wNíííÆÍé¶îÜ¹c_RTTÄl «dÓ"íX¤/6ç½U¬ULg×ëu,ÌÈÈ¸víZ0nÎì	¬ZóÍtY%«>îÜ¹ÞÝÝm-¹~ýºõ¥æs«*ú7ß|Ã¯ «dÓJIIIJJÙØçói^ÎjÈ*YÆéóÏ?WMgÏ½`ÁY!Y%«U² «d@VÉ*¬UÈ*YÅêáÃÇß³gNùt)@VÉ*0~_~ùeRRÒÚµk·oß®SÇ¤.Á£¿Þððð´´´êêj²JV_FOO:zàÀkIUUUtt4sV¼@ì9¹pá^&ÎdU²©åòåËZÂÁñ¢¸sç^ÚÉÙÞÞìCrU²vêÔ©7:æçç?~<à?øàæ²:Î9ãÿ7¼/ «dÎõë×ãââì¯ëÍfaÇWÅõÕWóæÍs»ÝIIIsçÎÕ@Ó¦sçÎ­]»Ö±0%%%H_wHVÉ*0ÍM³²²Ì÷wuuéÊÿµ¿BûÚk¯©¸æÇwÞygöìÙÖÀ/H/ÃÃÃ/`-9zô¨þbÉ*Y~zö)..NLLÔiaa¡£/½ô&¬öÊê¡CXÎ9£²êO·ªªJ/ÕTÏ7C;Y%«º4Uø¿¶¶622Ò±0))é÷¿ÿ=+SDû®]»TÖ=öè/yæ<p²JVñâéììô­2[È*YÆãµ×^óx<êë_÷iZ¶lÙÜ¹syo «¦­­MÏ2n·;55µ©©É~VKKË¢EtVZZ.FV1¨ ¯¿þú¬Y³"##].WDDDGG« «ÆëõÖÖÖjPYYk?+99ùêÕ«8qbáÂdÓf«Òÿaæ©Y`111ô÷÷'$$u±°°0]ìw~4Ó]½z5²ú#·Ûpl×ÚÚªY©²Zèçõ×_'«²ú7.Ëú_àéÓ§^¯·¯¯À²@ÈO4íïï75vÁ[¶lyôèÈêÈòòòjjj4Ð©f¥ö³|>ßÊ+?~ÌlduTããã].Çãiii±¦³:MHH±!«²Êá d¬È*Y¬UY%«²JVnV>zåÊ³gÏ<ö¬Èê8TQQ´iÓ¦'OêÇÓ§Oë4;;»¡¡¡ººZãÆÆF² «£ÊêºuëÌ¸®®.11Ñ5gxôè.pñâE² «£ÊêG©IjYYYXX&©ZØÔÔ¤ÁÞ½5¾ÿ>YÕQe5++ËïÝ»gª~ú©&¬>OãÛ·oUY[V5NOO7'NhpéÒ%¿þúk² «£ý¦¶²²²;wYØÑÑÑÐÐ`>ú¬Èê¨²á¹jÐÙÙÙ×××ØØÈ«²:*õõõªfxxxÝ £¢¢Ì@Õ ¤¤ÄLg§àYÉ*`jeõÑ£Gæã4f`ëÓ5W¯^åp²:ffJÚÕÕe-¼wï¸Ýî»wïUY7o*ùùùÏ5ï­&$$UYÁüùó/_>üe:;;O>MVdo°`r³ÚÚÚºnwPÝOÌøØ±c!!!UYÙóçÏÍþÀö³îß¿Ù*¬N² «d)Õ¥tÛ¶mþYíïï'«²:þªÆf%3&«²:þ¬²@VÉ*d¬È*YÕ±eµ··¬ÈêÈ.^¼cöøMOO·öþµÆ555æâÈ*¬àÙ³gj@Vù² «d0½³ÚÓÓS__oS´páBsØÂK.UY«W¯*¢ÝÝÝ111/6û«[ÌVdul­ÉÈÈ¨««s»Ý:Ü¿ÿÁÍ÷+´ÉÉÉd@VGpïÞ=eµ­­møî®[·¬ÈêÈSÕ+VhÐÞÞ?¨¡¡!<<üÃ?ìïïÿúë¯Ù «c;.k/(ï­ÈêÏÊêºuë¼Ì±¶lÙBVduÌ?ùäÖ××GEE544èÇêêêßy%«²êÌêgfÆ-²¥¸UYCV­vÆÇÇ§§§ÛÏ:qâYÕñÌV­ÃA¤¤¤wX;::È*¬!«õõõ¬hùòåËÙe	@VÇ)"""77CíÈ*ß`ÀÌj[[Çãq»Ý©©©MMMC¬^°¬ª^7o¶~ìèèÐÏÔ¬z½ÞÚÚZ*++ý7;?þ|Ñ¢E&«ßÿý~?¿ýíoÉ*`ÊeõÁªWkk«½²ÃL'JLLÌÀÀùb×Ç¹»wïÞ·o¹ß÷]¢¹sç®^½?ÀTÉªzVZZo>KsâÄ	óíåêuCûDv¹Ýîcó½:éééfWd6À/âÀ)))ááá:-//'«#3Ý:yò¤5I5_f¾iÓ&ûÌõ½÷ÞFV].5µsåÊsëd&_aa¡jªÞhìóù4Þ±cYÕ»ªÖA46¾ÿ¾ýû÷ï¨üDãØØXÍÍ¤Yãfs4Y ¹~ýztttww·ÉìÙ³>|HVÇÕùóç?~üXgÏY<Ùj^^^MM:õz½CÝ=f«0ÉN:µqãFÇÂ3y%«#d599Ù|o¹­ñññÖ***ÕææfÝËåòx<---;JV`ò]¾|9++Ë±pÁ³Õ±ÍVMA»ºº46©Ó`÷îÝf¸¸¸ºº:kÉÑ£GÕÞÞ^²:¬Z÷íÛGV`¦MX£££«ªª4¾víÚ´|¤Õ_nCV`æ¸sçÎ=ÔÔ]»vÝºukº>Ìà¾·zöìYÿOàLàÀd0m³êÿ¹Õ/¾øÂl¯­­Ý¶mãó6d@VS>UÍÎÎNóã·ß~k?÷È#öMÄd@Vùb8&=«¶mßzÛ¬Èêûí·ö£÷õõéÇ#Gøo&«²:äÛ«544x<µªa§Amm­ÆË/'«²:ªý;1>|÷Vdu<?~¼bæ¦»wï6_°j>Éj¦ªeeed@VÇðæÖnJjãÜÞÞ^² «#»råJî ë@K¶æÿDK;¦ö¯'«²:Â<ÕLU/^ÌçVdõç~Àæë¯¿¶¾¬ÈêøO©?¾¡¡Á¾Øì²d4¬Èê¨ZSS30HK.^¼¨%wïÞe¶ «cÐÓÓ£^½zÕ¾ÐUë+ÍÉ*¬éÙ³gÊg¿²ºnÝ:kà²²2-/((°v² «£=¡ýXKì² «|1S «K.õú©««		9vìYÕÑ²^_)MLL´¡³êëëÉ*¬!«JiÝ ?ü0,,L§<yRgÝ¾¬ÈêÄÌVÏ=KVduY]¸p¡ùó>ú(<<üO>Ñ¸¾¾^gÝ¸q¬Èê²zÿþ¾ûî»Ö+W>þüôéÓì	 «£uöìYk§ßØØXó«ÆQQQTTTèUYÕ±¬jNN5^·n[Õño&«²:þ¬¶¶¶®4ÔF`3ÎÌÌ$«²:Ú£÷õõ¨ ÷îÝÓ7oÞ<þü|K¬¦nVEY­®®6»Û¸¯â>|XËÓÓÓÉ*¬¬¡¡aþüù555C]àéÓ§*ë¦MÈ*¬ò6U² «d@VÉ*¬UÈ*YU² «d²JVd¬È*YU²Y%«²:AÚÚÚ<ÛíNMMmjj²õìÙ³-[¶Î?ßçóUY×ë­­­Õ ²²277×~VYYÙÞ½ÔÔ¤¤$-ù~6lØ@VdõobbbÌ·¦÷÷÷'$$ØÏÒüµ³³Óúñ»ï¾	äí·ßæ@Väv»ÍaaaªvtthÉ?ÌVdõ¹kê8«ººZÛ·o§§§óÞ*¬`m¿Õ866¶¿¿ßlÖØ~1û,YÕòòòjjj4Ð©×ëµUTTtòäInÞ¼AVduÍÍÍñññ.Ëãñ´´´XÓY>yò$''GóÔôôô»wïUYåp²JVd¬@VÉ*¬UY%«U² «d@VÉ*¬UÈ*YU² «d²JVd¬È*YU²Y%«²JVd¬È*Y%«²JVd¬È*Y¬UY%«²JVd¬UY%«²JVd¬@VÉ*¬UY%«²JVÉ*¬UY%«²JV «d@VÉ*¬UY%«U² «d@VÉ*d¬È*YU² «/dVÛÚÚ<ÛíNMMmjj²åóùRRRÌYÍÍÍd@VGàõzkkk5¨¬¬ÌÍÍµsïÞ=t@VdujçÀÀýýýv&%%=xð@jüÝwß%ø	_½z5²ú#·Ûpl¶è´µµõûï¿?à'##Ù*¬þËå²Æ¡¡¡ö³/^ÜÙÙiúºtéR6Èj!?Ñ866¶¿¿ßlÖxY² «äååÕÔÔh S¯×k?K3Ô7ojpãÆÍ*¬ ¹¹9>>Þåry<k:«Ó®®.ÕTóTjLVdÃAÈ*YU²Y%«²JVd¬@VÉ*¬UY%«²JV «d@VÉ*¬UÈ*YU² «d@VÉ*d¬È*YU² «d¬È*YU² «d²JVd¬È*YU²JVd¬È*Yªºººììì;wîðWd¬º`Áªªª.ÇÅÅéÌ:U²:f§NJLLìîî¶|þùçª,è@VÉêÛôööFGG?|ø¿u «duÌYÕôÔ±0..¬Y%«cvêÔ©kÉñãÇÙd¬SNNNFFÆnÝº¥kddäåËùC²JVÇiÏ=¶z½^v²JVd¬È*Y%«²JVd¬È*Y¬UY%«²JVd¬UY%«²?NHHð_ÞÖÖæñxÜnwjjjSSYÕ477§¤¤¸ç^¯·¶¶VÊÊÊÜÜ «#PïÞ½0«111ô÷÷k:«ñV?.z5²j»ß²êv»íceuµ_ÿú×d@VGÎªËå²Æ¡¡¡lÕÀ5Ïjlll¿Ù¬1YÕñÏVóòòjjj4Ð©×ë%«²:¬ããã].Çãiii!«²Êá d¬È*Y¬UY%«²JV «d@VÉ*¬UY%«U² «d@VÉ*dÕ¦¨¨hîÜ¹'NRRÒ?üÃ?,Dðýæ7¿aU³ªYÕ"«Z)!«?êêê:2¡³²² øô_â_þå_X@O@«V­b=L×^íí·ßf=LÄÄÄ5kÖLà?xôèÑÿùÿ!«oÅÛ¶mc=Lú§úÿøÖÃ$ÈÈÈøÏÿüOÖÃ$X²dIii)ëa¼ùæÛ·o"«d¬¬U²JVÉ*YY%« «dd¬¬UU²JVAVÉ*Y¾÷ßÿÓO?e=L7îÙ³õ0	6lØð§?ýõ0	Þï½ûö±&ANNÎþýûÉ*/²Y¬N'mmmÇív§¦¦655±B&Ë¢E´ÓÒÒ´¶YçÁvñâÅþ¼êÙ³g[¶l	?¾ÏçcUVoJJY±ÍÍÍ¶ªÉêøy½ÞÚÚZ*++sssY!.99ùêÕ«8qbáÂ¬ó zþü¹^ÄXYeUIYYÙÞ½ô¤Äª÷îi ÓI[ÕdõgýÎôCþþ~ó;Cð±Îj÷îÝûöí³²ÊªÍ:;;y&zÕòàÁtj^ÁLÎª&«ãçv»1áZ[[·nÝÊ:½OOO×3UVuð7***ô2QOô¬êàikkÓzÖ´Nõ2i«¬Ëå²Æ¡¡¡¬ yúô©×ëíëëcONNÎ+W~|Fø)«¬êà=oTWWkpûöm½aUÏâÅÍõuéÒ¥¶ªÉêøÅÆÆö÷÷í	³BáÁ[¶lyôèë<¨Bþ/VuP7&Vuð6ü"«¬_^^^MM:Õt2á|>ßÊ+?~Ì:Ì¾²ªª¨¨èäÉÜ¼y3##U<¡j%kpãÆÍÕñknnw¹§¥¥2áS(Öù¤eU$O<ÉÉÉÑä)==ýîÝ»¬êàéêêRMµªuªñ¤­j²Y¬@VY¬@V «¬@V «¬@VÓ¥KBBB=¦ÓÝ»wkITTTjjj]]×ëÕ©ù¼b¾æ;Ï?úè#óc__~4ß$UÓÄóçÏvîÜ©È<yR§~ú©N###>=G±~LIIY¼xqþ ]ýìÙ³ºÀºuë¬%ö<yòÄºJss³X_b²Z__Ï¯dÀ´¢IêÅ9%V§çÏÿá§/*·ÏV­ÊÞ»wO?Þ¿_§+V¬0í_sk®^RRâX¢úêrrrô;vLKÖ¬Y£ñáÃ5®¬¬äw²à|úôéN¬©©Ù¿¿:§§='N0cÇ£)©«®¥¹©¦¦¦©jmm­Ü¸qÃººÞ¼yS	7K>ª%ª,ëdÀôú_òÅ_¹©yû3jÐ§~ªFRÚg«ÖµÌwwtthÐÞÞ®Só=Û,^¼Ø~aÓiý;<xPK¬ç²`:hllìïïWÍ¨===½½½¦yg«æÇ+W®$$$m¹°Þ¾;>>>77Wû¿¯üÝwß5ÛÍusrrÖ¿Ã/dÀô°*®ö=u5ÎÎÎUµ³µµUK<x`ÞµÞX5¿þúkÇ?~óæM^ºtÉæy°5[ÕdßÈ*éÀ¼Kºÿþêêj3wt»ÝIIIu?±oÖìÓÔ±³³S§ºXÃ ³SÄÄÄ¨úÇ5¶9àlÕ>È*Øþð6ÙgWWNÛÚÚÌnímd¦ª¸M)ôèÙIøùóçþíd¶U`Úêééyüø±ù|NÍDæNSÇÚÚZÇã¸ùk^^UG#<w0[È*0O¿twwë´¦¦ÆZîr¹/_®R¬êÖ.^¼hf¢:ÕÕléÕTuÈçf«YfóR3O5§Û¶m3[UÏäÐq Á'O@*¥VVÍa%Æ:[½yóæ0IÈ*Æýû÷­<xàóù¬9¥fggÛç²õõõú±··W9ÔçÏ¿jÕ*sVOOÎòÏê±cÇÌÞOº¼c¶Z\l)Á¯dÀÌ²páÂÚÚZëÇóçÏ;Ä¯é¯cÂj¨*3%È*d²Yd²Yd²Y¬²Y¬²Y¬0ÝýAlÝt	IEND®B`


IVYUYèêê:tèPmØ°!ÚÛÛÃ óùÊ«,É* «²*«W¯^Ý¾8Ommm=öl	1É$Él6[(>yÄdUYUoÈª¬Êª¬²*«È*¬"«²º@páè9sæ=¬Êª¬Êê"òòË/'æ=¬Êª¬Ê*ÓÛ»w¯Èª¬Êª¬"«Èª¬Êª¬"« «Èª¬Ê*Èª¬Êª¬"«Èª¬Êª¬"«Èª¬"«È*È*²*«²²*«²*«È*²*«²*«È*È*²*«²²*«²*«È*²*«²*«È*²*«È*²²¬Êª¬¬Êª¬Ê*²¬Êª¬Ê*²²¬Êª¬¬Êª¬Ê*²¬Êª¬Ê*²¬Ê*²¬¬"«²*« «²*«²¬"«²*«²¬¬"«²*« «²*«²¬"«²*«²¬"«²*«²¬¬"«²*« «²*«²¬"«²*«²¬¬"«²*« «²*«²¬"«²*«²ºh?·áÎ-[¶ô¡Rstçáaÿà ²¬Êê<rôèÑÆÆ/85÷ÕõÃmmmyGYEVeu~eõ¿Ö7×âýûÿîwÿÇd&R©T¢f9|²*«²*«Èª¬Êª¬Êª¬âE`/Ëª¬"« «Èª¬Êª¬"«²*«²*«²¬Êª¬Ê*²¬Ê*²¬Ê*²¬Êª¬Ê*²*«²*«²*«Èª¬Êª¬"« «Èª¬Êª¬"«²*«²º²Z»oêöÌ3O;È*²*«²zü÷?å"«Èª¬Î¯¬66~±ëÛjîkóS-ùüGYEVeu~eÕïVUYUYEVeYEVeUVeYUYUYUdUVeUVUUdUVeUVUYUYUYEVeUVeUVeYUYUddYUYUdUVeUVeUVUYUYEVUYEVeUVeYuødUVeUVUYUYUYEVeUVeYYEVeUVeYUYUYUdUVeUVUdUVUdUVUdUVeUVUY]äYÇ¯¥¥EVeUVUYÕOÕ±±±pü:::¦]½~ýº¬.Ô¬ãÊ:_-O<¶funî<<ìgÆDV§YÝ·o_8~Ï?ÿ|þ¶Ã·ÅW_5,uvvÊêÂsáÂ£s&ü--<sæîþÏ9ã"«ÌÇ¬;w.¼ãÇW¾ ü_üÂÀ|÷îõCYÕEÕñññpäÂ)é¤ß³³6UdYUYé¿TúðÃ³Ùlkkkùßø/Áo~ó0~ñÅeYEVeUVï~ªzóæÍ8®MMM^FVUYÕûðOdYEVeUVïgV+ÿÓê¤¬3ÚÿðoXeUVAVý	ª½¬Þ¸qcýúõG	oÉ%aÐÑÑ_ó·ÅW°±¬"«Èª¬Êê]Ê:íÉ«UdUVeõþUdYUYUddyÕÞÞ^YEVUYÕæØ±cáIÅb6¢¨¹¹¹¿¿¿r³*K²*« «²º@²ßÎ;'Í|ë[ßáÍoÞ¼ÙÒÒRÎj>ïëëîîî¶¶¶Ê-§.bÛ¶m²*«p_<úèçúþ°'µæ¾ü	ªÕ¬#÷ÁËpºYÉ0Ëåfx'?ÿùÏ_yårVëëëãÿêZ*&ÝÉ¤¥[·n%¦³eË[VáÓK§²sÖ¹øzè¡T&³|îÜ ÎêÐÐP¸<pàß¯9ìÙ³'L9r¤®®.|³±±±»ÞÃùóç[[[ãwíg¢(*¯V§]âûßÿ¾³UYù/þôCûAV'guxx8ñÆûöí»råJê÷°ûö'NÄ·gÉdy5JUnÉïVedÎj8/?¯&þ¤pùÛßþ6þìÕpùÖ[omâ¥°eõ0W3étºT*Å¯ôqåÆUdUVAV©í³Õò»,:tèüùóåLñÁÃ Äï^ô9çUúÚÛÛãÿ¢.óù|å6UdUVAV©í¬~øá###qÅâ'®_¿^ÎdüÍª­NÍj¸U&I&Ùl¶ür¼:í¬Ê*È*!«á$µ¡¡áý÷ßOS§NËçnxxxÛmG_¹õvÈ*Èª¬ÞESSSüëÕýë_ñoFC¥Rùà&«¬"« «²zG¡©¡»wïãðÙ¿ÿåËCeÃÌêÕ«7lØàÍUUYiV_íµÊ_nß¾=O§Ówú'E²¬¬Êê4º»»ÃÓbéÒ¥¿þõ¯ÿú×¿ñ/¼/pµ««KVUUYsçÎU¾þµk×ÊWßzë­ð¤ö£ÎeYYÕÚ «²²¬Ê*²¬"«²¬¬Êª¬"« «Èª¬Ê*È*²*«È*Èª¬Ê*²²¬Êª¬¬"«²¬"«Èª¬"« «²*«È*È*²*«²²¬Êêböúë¯'æÌ×¿þuYEVeu9úô¦9ó³ýÌFVUYdYUYEVdYU@VUYUYUYEVeUdUVYEVe@VeUVdYU@VUYUYUYEVeUdUVYEVe@VeUVdYU@VUYUYUYEVeUdUVYEVe@VeUVdYU@VUYUYUYEVdYU@VUYUYUYEVeUdUVdUVe@VUYEVeUdUVdUVeà~hhhHÌ»WVeUYUYYUdUVUY@VedUVUY@VedUVUY@VeYUUY@VeYUUY@VeYUdUV@VeYUdUV@V@VeYUdUV@VeYUdUV@V@VeYB¡ÐÒÒEÑÚµkÅb	Ùl6Ì477÷÷÷WnÕU«V:u*<¸zõê0ÈçóaÐÝÝÝÖÖV¹ñ¤¥?þø§Ø¸q£¬°H³Z©®®.××A©TÊårLZºuëVzÏ|æ3[¶lq°XÔYÜµkWDQT¬W_ò"0²úk×®åóù±±±0N&åùT*U¹Y%Y@VÿíâÅ.]¯¦ÓéR©¿ÒÆ[VYUdub```óæÍW®Ï´··÷ööA¸§°WYUdu"Ë%*ð3L2Ìf³Báxç%Y@V½²*« « «²¬Ê*²*« «²¬Ê*²*« « «²¬Ê*²*« «²¬Ê*²*«Èª¬¬Ê*²*«Èª¬¬Ê*²*«Èª¬ «²²*«Èª¬ «²²*«Èª¬ «²¬Ê*Èª¬ «²¬Ê*È*Èª¬ «²¬Ê*Èª¬ «²¬Ê*È*Èª¬ «²0+ï¾ûî×¾öµ(Ö¬YsìØ±òüo¾H$ìYUjúç?ÿ9?¾lÙ²xrttô«_ýª¬Êª¬ÌÒáÃüñxüÒK/íÛ·OVeUVîY87øáCDÿûß«ÃÃÃO<ñÄÕ«WeUVe`Þ|óÍåËÁ³Ï>ûöÛo¬Êª¬Ì^EqM+Ù-²*«3µråÊwß7þö·¿=ùäK*«²poþþ÷¿¯Y³&§nÜ¸ñÿü§¬Êª¬ «²²*«Èª¬Ýþô§ÖÖÖÏ~ö³?úÑFGGíYUÙøÕ¯~ÕÐÐpôèÑ0>sæLø¹ÜÕÕe·Èª¬Ü³Ë//Y²$Ô´<sáÂ0óÞïÙ9²*«÷&uÃ&¿óï¼þúëv¬Ê*À½	g¥MMM&yæÃÛ9²*«÷¬¡¡¡§§§|õôéÓK,±gdUVîYèèç?ÿùßýîwöìY¶lÙ_þò»EVe`.ðòË/ïÜ¹óÇ?þq¨¬"«²²²*«Èª¬ «²²*«Èª¬ «²¬Ê*Èª¬ «²¬Ê*Èª¬ «²¬Ê*²*« «²¬Ê*²*« «²¬Ê*²*«Èª¬¬Ê*²*«Èª¬¬¬VS,³ÙlEÍÍÍýýý²¬Î^>ïëëîîî¶¶6Y@Vg¯¾¾~||<J¥R.ã®)üñ§~ÚÁ@Vï"¢ÊqÈêSS|á_UdõîÉdyJ¥¼¬Î^:.JñÀa,«Èêìµ··÷ööA¸Ìçó²¬ÎF"ñï&I&Ùl¶P(È*²êí UYYYUdUVUYYUdUVUYYYUdUVUYYUdUVUYÅÕ®®®~x5µì±Çûò¿l?°å+_Y±bÅªU«ìr³ð³:22r·cÇúúzûì¿üe"xñÅíöÇ?þñ£>ZàYexåWí°Ë/¬¾óÎ;vÅ$«È*È*²¬¬"« «Èª¬Ê*È*È*²J­éééùæ7¿i?°ôÑG+W®<sæ]!«¬¬¬Â=¸råJ.:_,³ÙlEÍÍÍýýýv5ªP(´´´gòÚµkÃ³Ú<y²©©)æyÏçûúúÂ »»»­­Í¾¢F­ZµêÔ©SapðàÁÕ«WË*Ì¡­[·=vÚ¬Ö××A©TötjN]]'¹¬ÂÜ?§ËjEÓ¡FîÚµËáÁd5LÇ©TÊ^¢¦]»v-ÏyË*<¬¦ÓéR©4qûõ±0¶¨]/^ììì¼té'¹¬ÂËjoooËð×|500°yóæ+W®xË*<¬ÆWO<ÉdÉd6-ö5*Ë%*xË* « « «¬¬¬¬²²²²È*È*pïDågv&Éµk×NÜþDi?_on|<o²Ü?ùÉOBÃ8Pééé	3/½ôÒ¬pO=õ¬¬BÍ+¡a[·n-ÏlÛ¶-ÌÔ9=YîY:N&×®]ã±±±03SëëëW¬XqöìÙC=òÈ#&W­ZEÑÚµkß~ûíIí3a>¬®[·nhh¨<_ùqÜS¿éÔ[¬óÔÎ;CºBÃø7Þã0s§¬µ´´Çãø±Á±cÇ&mV>ëML9Ã¬N½È*0O9r$äjûöíaÏçÃ8ÌÜ)«¡¾çÎÇ¯½öÚÈÈHóÈx'|2~üx<y2ÛÚÚ*o¾÷îñññø;¦R©©ßeÚo:í­@VùèæÍÑm×¯_aæNyvo7éäòG©¼ùØØØî¹JV§½È*0O=÷Üs!WárÇUW<)«Édrí¼×%U`ÿSMù¥ÝYguÝºua|ìØ±i~(Ü­7nÜÏJedjÛ¥KÊ'£££³ÎêáÃ'­ÿëN@.]º4¾ÚÞÞ.« «°Ä'6mºÓ)æLÆA___SSSÈó%KvïÞõêÕ»2Ü$ÉDQ´qãFYYYYYdddUUUU@V@V@VYYYYdÿ&mË-#ñIEND®B`


T-TEST GROUPS=Vimentin(1 2)
  /MISSING=ANALYSIS
  /VARIABLES=年龄
  /CRITERIA=CI(.95).


T 檢定


附註	
已建立輸出	15-MAY-2019 18:59:45	
備註		
輸入	作用中資料集	数据集2	
	過濾器	<無>	
	粗細	<無>	
	分割檔案	<無>	
	工作資料檔案中的 N 列	76	
遺漏值處理	遺漏的定義	使用者定義的遺漏值會被視為遺漏。	
	已使用觀察值	每一個分析的統計資量是根據觀察值，該觀察值對於該分析中的任何變數沒有遺漏或超出範圍的資料	
語法	T-TEST GROUPS=Vimentin(1 2)
  /MISSING=ANALYSIS
  /VARIABLES=年龄
  /CRITERIA=CI(.95).	
資源	處理器時間	00:00:00.00	
	經歷時間	00:00:00.01	


群組統計資料	
	Vimentin	N	平均數	標準偏差	標準錯誤平均值	
年龄	1.0	6	47.167	7.8337	3.1981	
	2.0	38	49.447	14.7129	2.3868	


獨立樣本檢定	
	Levene 的變異數相等測試	針對平均值是否相等的 t 測試	
	F	顯著性	T	df	顯著性 （雙尾）	
						
年龄	採用相等變異數	2.430	.127	-.369	42	.714	
	不採用相等變異數			-.572	11.633	.579	

獨立樣本檢定	
	針對平均值是否相等的 t 測試	
	平均差異	標準誤差	95% 差異數的信賴區間	
			下限	上限	
年龄	採用相等變異數	-2.2807	6.1816	-14.7556	10.1942	
	不採用相等變異數	-2.2807	3.9905	-11.0058	6.4444	

CROSSTABS
  /TABLES=Vimentin BY 性别
  /FORMAT=AVALUE TABLES
  /STATISTICS=CHISQ CORR KAPPA
  /CELLS=COUNT EXPECTED COLUMN
  /COUNT ROUND CELL
  /METHOD=EXACT TIMER(5).


交叉表


附註	
已建立輸出	15-MAY-2019 19:00:32	
備註		
輸入	作用中資料集	数据集2	
	過濾器	<無>	
	粗細	<無>	
	分割檔案	<無>	
	工作資料檔案中的 N 列	76	
遺漏值處理	遺漏的定義	使用者定義的遺漏值會被視為遺漏。	
	已使用觀察值	每一個表格的統計資料都以每一個表格中，所有變數指定範圍中具有有效資料的所有觀察值為基礎。	
語法	CROSSTABS
  /TABLES=Vimentin BY 性别
  /FORMAT=AVALUE TABLES
  /STATISTICS=CHISQ CORR KAPPA
  /CELLS=COUNT EXPECTED COLUMN
  /COUNT ROUND CELL
  /METHOD=EXACT TIMER(5).	
資源	處理器時間	00:00:00.05	
	經歷時間	00:00:00.07	
	要求的維度	2	
	可用的資料格	131029	
	精確統計資料的時間	0:00:00.00	


警告	
CORR 統計資料只可用於數值資料。	


觀察值處理摘要	
	觀察值	
	有效	遺漏	總計	
	N	百分比	N	百分比	N	百分比	
Vimentin  * 性别	44	57.9%	32	42.1%	76	100.0%	


Vimentin *性别 交叉列表	
	性别	總計	
	男	女		
Vimentin	1.0	計數	4	2	6	
		預期計數	3.4	2.6	6.0	
		性别 內的 %	16.0%	10.5%	13.6%	
	2.0	計數	21	17	38	
		預期計數	21.6	16.4	38.0	
		性别 內的 %	84.0%	89.5%	86.4%	
總計	計數	25	19	44	
	預期計數	25.0	19.0	44.0	
	性别 內的 %	100.0%	100.0%	100.0%	


卡方測試	
	數值	df	漸近顯著性 （2 端）	精確顯著性（2 端）	精確顯著性（1 端）	
皮爾森 (Pearson) 卡方	.275a	1	.600	.684	.475	
持續更正b	.007	1	.936			
概似比	.281	1	.596	.684	.475	
費雪 (Fisher) 確切檢定				.684	.475	
有效觀察值個數	44					

a. 2 資料格 (50.0%) 預期計數小於 5。預期的計數下限為 2.59。	
b. 只針對 2x2 表格進行計算	


對稱的測量a	
	數值	
合約的測量	卡帕 (Kappa)	.b	
有效觀察值個數	44	

a. 相關性統計資料只可用於數值資料。	
b. 無法計算卡帕 (Kappa) 統計資料。它需要雙向表格，其中的變數具有相同的類型。	
