# Supplementary material for: High-order radiomics features based on T2 FLAIR MRI predict multiple glioma immunohistochemical features: A more precise and personalized gliomas management
Source: PLoS One. 2020 Jan 22;15(1):e0227703. doi: 10.1371/journal.pone.0227703 (PMC6975558; doi:10.1371/journal.pone.0227703)
Supplement: Attachment — Submitted filename: response to the view letter12-19.docx [file pone.0227703.s022.docx]

Dear Editors:

Thank you very much for your view letter.

Thank you for the question: “We note that you have also added your ethics statement to the "Data Availability Statement" section of the on-line submission form.

In your Data Availability statement, you have not specified where the minimal data set underlying the results described in your manuscript can be found. PLOS defines a study's minimal data set as the underlying data used to reach the conclusions drawn in the manuscript and any additional data required to replicate the reported study findings in their entirety. All PLOS journals require that the minimal data set be made fully available. For more information about our data policy, please see <http://journals.plos.org/plosone/s/data-availability>.

Upon re-submitting your revised manuscript, please upload your study’s minimal underlying data set as either Supporting Information files or to a stable, public repository and include the relevant URLs, DOIs, or accession numbers within your revised cover letter. For a list of acceptable repositories, please see http://journals.plos.org/plosone/s/data-availability#loc-recommended-repositories. Any potentially identifying patient information must be fully anonymized.

Important: If there are ethical or legal restrictions to sharing your data publicly, please explain these restrictions in detail. Please see our guidelines for more information on what we consider unacceptable restrictions to publicly sharing data: http://journals.plos.org/plosone/s/data-availability#loc-unacceptable-data-access-restrictions. Note that it is not acceptable for the authors to be the sole named individuals responsible for ensuring data access.

We will update your Data Availability statement to reflect the information you provide in your cover letter.”

Answer:

We had uploaded our study’s minimal underlying data set as Supporting Information file named as “ supporting information”, in this compressed package file, it consists of S_Fig, S_formula, S_Table and statistical analysis. S1_Table includes all the patients’ information and any potentially identifying patient information was fully anonymized. S2_Table contains a total of 396 features were obtained from each case. Profiles of the selected four feature clusters can be found in S3-S6_Tables. Statistical analysis consists of Ki-67, vimentin, S-100 and CD34 four immunohistochemical statistical analysis data. We also had uploaded the “textureParamters” file as Supporting Information, which is AK Software manual applied in our research. In addition, there are not ethical or legal restrictions to sharing our data publicly.

Thank you very much and best regards.

Yours sincerely,

Jing Li.

Corresponding author:

Name: Huaijun Liu

E-mail: huaijunliu2@126.com.
